# Csp^3^–Csp^2^ Coupling of Isonitriles and (Hetero)arenes through a Photoredox-Catalyzed Double Decyanation Process

**DOI:** 10.1021/acscatal.4c06269

**Published:** 2024-11-08

**Authors:** María Martín, Rafael Martín Romero, Chiara Portolani, Mariola Tortosa

**Affiliations:** † Organic Chemistry Department and Center for Innovation in Advanced Chemistry (ORFEO−CINQA), Universidad Autónoma de Madrid (UAM), 28049 Madrid, Spain; ‡ Institute for Advanced Research in Chemical Sciences (IAdChem), Universidad Autónoma de Madrid, Madrid 28049, Spain; § Department of Industrial Chemistry “Toso Montanari”, Alma Mater Studiorum−University of Bologna, via P. Gobetti 85, 40129 Bologna, Italy

**Keywords:** Deamination, Isonitriles, Photoredox Catalysis, C−N Cleavage, Pyridines

## Abstract

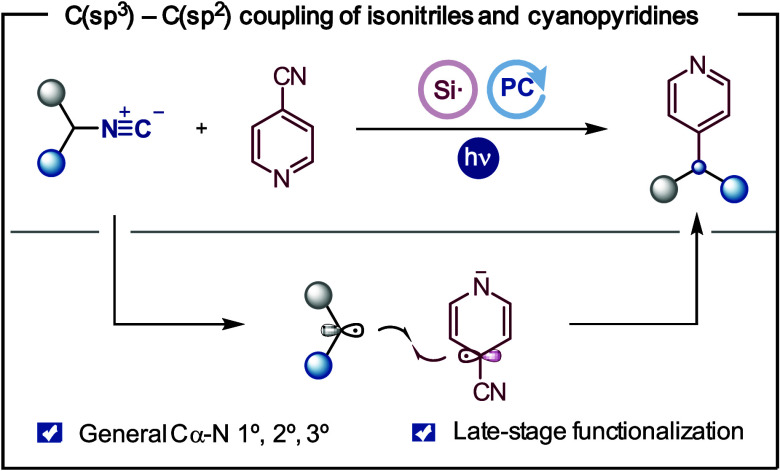

Herein, we demonstrate the ability of isonitriles to be used as alkyl radical precursors in a photoredox-catalyzed transformation involving selective C–N cleavage and Csp^3^–Csp^2^ bond formation. This protocol allows for the preparation of functionalized heteroarenes from readily available isonitriles through a decyanation process. The reaction is general for primary, secondary, and tertiary substrates, including amino acid derivatives and druglike molecules.

Isonitriles are one of the most intriguing functional groups in synthetic chemistry.^1−3^ Their chameleonic electronic properties allow them to act as nucleophiles, electrophiles, carbenes, and radical acceptors. They can be easily prepared in one or two steps mostly from primary amines,^4,5^ but also from alkenes^6^ and tertiary alcohols,^7^ which are three of the most abundant functional groups in chemistry (Scheme 1). Moreover, isonitriles are present in hundreds of secondary metabolites with a growing interest in medicinal chemistry.^8−10^ The reactivity of the multiple N≡C bond in isonitriles has been extensively studied through the Ugi and Passerini multicomponent reactions,^11,12^ metal coordination,^13^ biorthogonal [4 + 1] cycloaddition,^14^ polymerization reactions,^15^ and *N*-formyl amide formation for the synthesis of complex peptides.^16^ In contrast, the selective cleavage and functionalization of the single C–N bond in isonitriles is mostly limited to the formal hydrodeamination reaction (Scheme 1).^17−19^ Despite their availability from feedstock material, the use of isonitriles in catalytic C–N cleavage/C–C bond formation remains underexplored.^20^ We recently disclosed that isonitriles can undergo a mild and general formal hydrodeamination reaction promoted by visible light irradiation in the presence of a silyl radical precursor.^21,22^ With the aim of further expanding the use of isonitriles in reactions involving C–N cleavage, we identified the photoredox-catalyzed coupling with cyanopyridines as a synthetically attractive transformation. Cyanopyridines have been widely used in photoredox catalysis to functionalize C–C, C–H, C–B, and C–O bonds.^23−34^ However, their use to promote the heteroarylation of C–N bonds is currently limited to benzylic substrates (Scheme 1).^35^ Herein, we show that isonitriles are capable of overcoming these limitations through a photoredox catalytic cycle, providing a general Csp^3^–Csp^2^ coupling with cyanopyridines through a double decyanation process.^36^

**Scheme 1:**
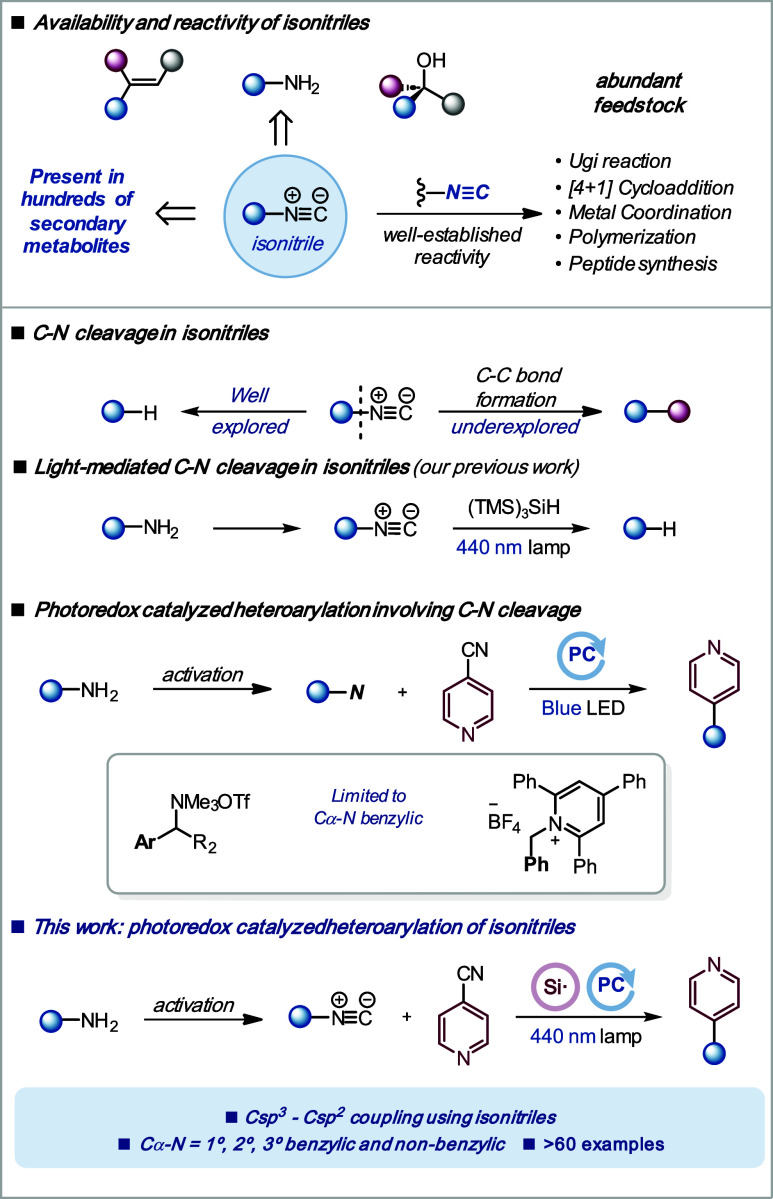
Availability and Reactivity of Isonitriles and Photoredox-Catalyzed C–N Heteroarylation

We hypothesized that upon visible light irradiation, a photocatalyst could induce the formation of a silyl radical through a single-electron oxidation (Scheme 2).^37−41^ The silyl radical could be added to the isonitrile to form an imidoyl radical that would generate a carbon-centered radical upon β-fragmentation. The reduced photocatalyst could then reduce a cyanopyridine to provide an aryl radical anion that could react with the alkyl radical through radical–radical coupling to afford the desired product. The products would be functionalized pyridines, which are the second most common nitrogen-containing heterocycles present in small molecule drugs.^42^

**Scheme 2:**
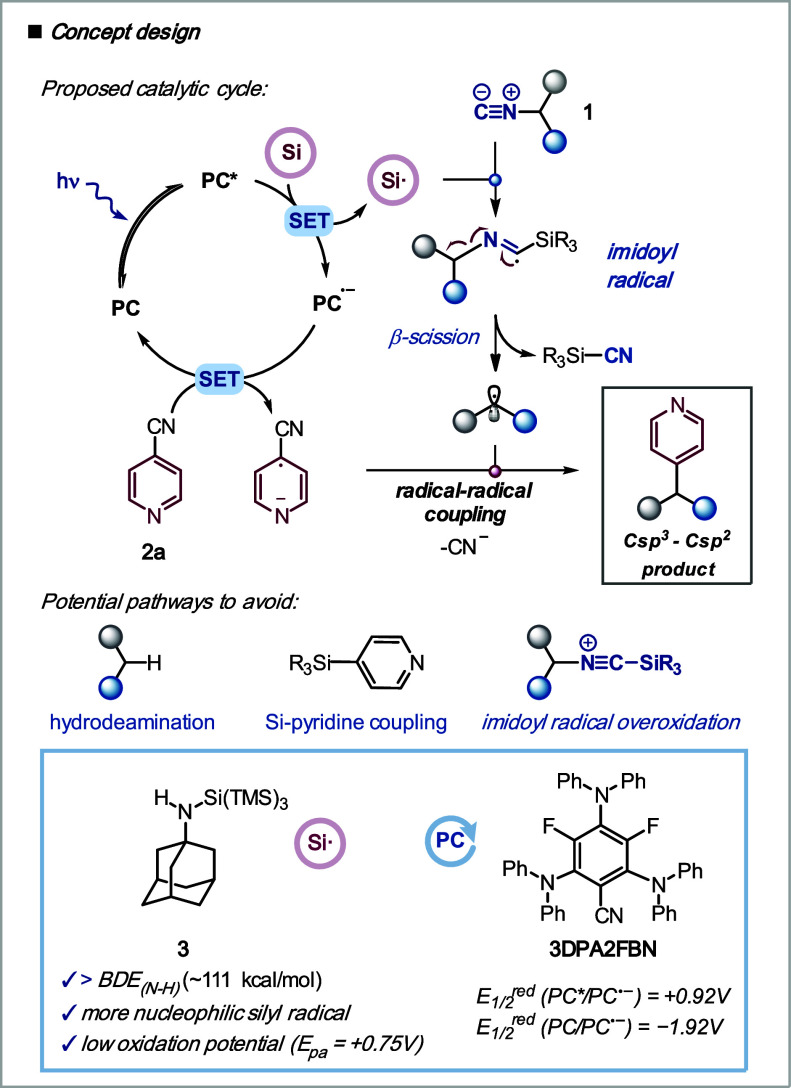
Plan Design

We recognized from the outset that the choice of the silyl radical precursor would be key to avoid three undesired potential pathways: the hydrodeamination, the silicon–pyridine coupling, and the oxidation of the imidoyl radical prior to β-fragmentation. We identified *N*-adamantyl amino-supersilane reagent **3** (Si–NHAd), introduced by MacMillan et al. to promote halide abstractions in the cross-electrophile coupling of unactivated chlorides,^43^ as the reagent of choice. The bond dissociation energy of the Si–H bond of a silyl amide (∼111 kcal/mol)^44^ is significantly higher than that of supersilane (TMS)_3_SiH (79 kcal/mol),^45^ which would suppress the dehydrodeamination pathway. Additionally, the increased nucleophilic character of the amino silyl radical generated compared to that of supersilane could minimize the formation of the silicon–pyridine coupling product. Importantly, this reagent shows much lower oxidation potentials [*E*_pa_(Si–NHAd/Si–NHAd^+•^) = +0.75 V vs SCE in 10:1 DMA/H_2_O]^46^ than supersilane [*E*_p_(Si–H/Si–H^+•^) = +1.67 V vs Ag/AgCl, 3 M, KCl]^47^ or supersilanol [*E*_p_(Si–OH/Si–OH^+•^) = +1.54 V vs SCE in CH_3_CN],^39^ which could prevent the overoxidation of the imidoyl radical.^48^ For the photocatalyst, we identified the organocatalyst 3DPA2FBN^49^ as a suitable catalyst capable of oxidizing the amino-supersilane reagent [*E*_1/2_^red^(PC*/**PC**^**•–**^) = +0.92 V vs SCE in CH_3_CN] and reducing [*E*_1/2_^red^(**PC**/ **PC**^**•–**^) = −1.92 V vs SCE in CH_3_CN]^49^ the 4-cyanopyridine **2a** (*E*_1/2_^red^ = −1.75 V vs SCE in CH_3_CN).^50^

Based on these mechanistic considerations, isonitrile **1a** and 4-cyanopyridine (**2a**) were chosen as model substrates. After optimization of different parameters, optimal conditions were found involving silane **3** and photocatalyst 3DPA2FBN (5 mol %) in acetone under blue light irradiation. With these conditions, pyridine **4a** was obtained in 84% isolated yield without detecting any hydrodeamination product (Table 1, entry 1). Other more commonly used silyl radical sources (Table 1, entries 2 and 3) proved to be less effective. Control experiments revealed that blue light, photocatalyst 3DPA2FBN, and silyl derivative **3** were all individually necessary (Table 1, entries 4–6). The presence of oxygen in the reaction mixture led to a decreased 20% yield (Table 1, entry 7). The reaction was scaled up to the use of 1 g of isonitrile **1a** with good reproducibility (Table 1, entry 8). Moreover, detailed studies, including Stern–Volmer quenching, quantum yield determination, and detection of R_3_SiCN species, supported the catalytic cycle proposed in Scheme 2.^51^

**Table 1:**
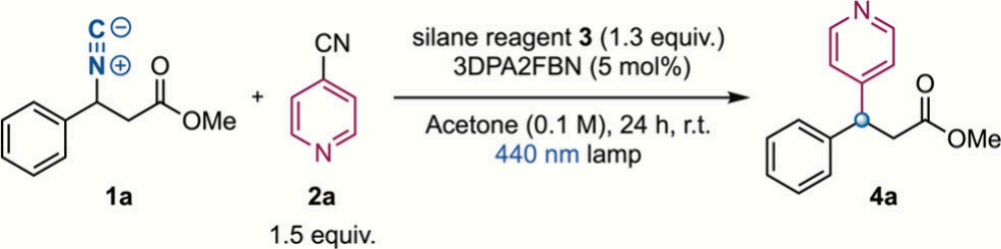
Optimization of the Reaction Conditions^a^

entry	deviation from conditions	yield (%)
1	none	85 (84)^b^
2	TMS_3_SiH instead of **3**	24
3	TMS_3_SiOH instead of **3**	47
4	no light	0
5	no 3DPA2FBN	0
6	no silane	0
7	open air	20
8^c^	1 g scale	73^b^

a All reactions were performed on a 0.10 mmol scale. Yields determined by ^1^H NMR yield using 1,3,5-trimethoxybenzene as an internal standard.

b Isolated yield. See the Supporting Information for experimental details.

c Reaction time = 48 h.

With the optimal conditions in hand, we first explored the scope of the decyanative arylation with benzylic derivatives (Scheme 3). We were pleased to find that the optimized conditions worked efficiently for a wide array of Cα-primary (**4l**-**4w**), secondary (**4a**-**4i**, **4k**), and tertiary (**4j**) isonitriles.

**Scheme 3:**
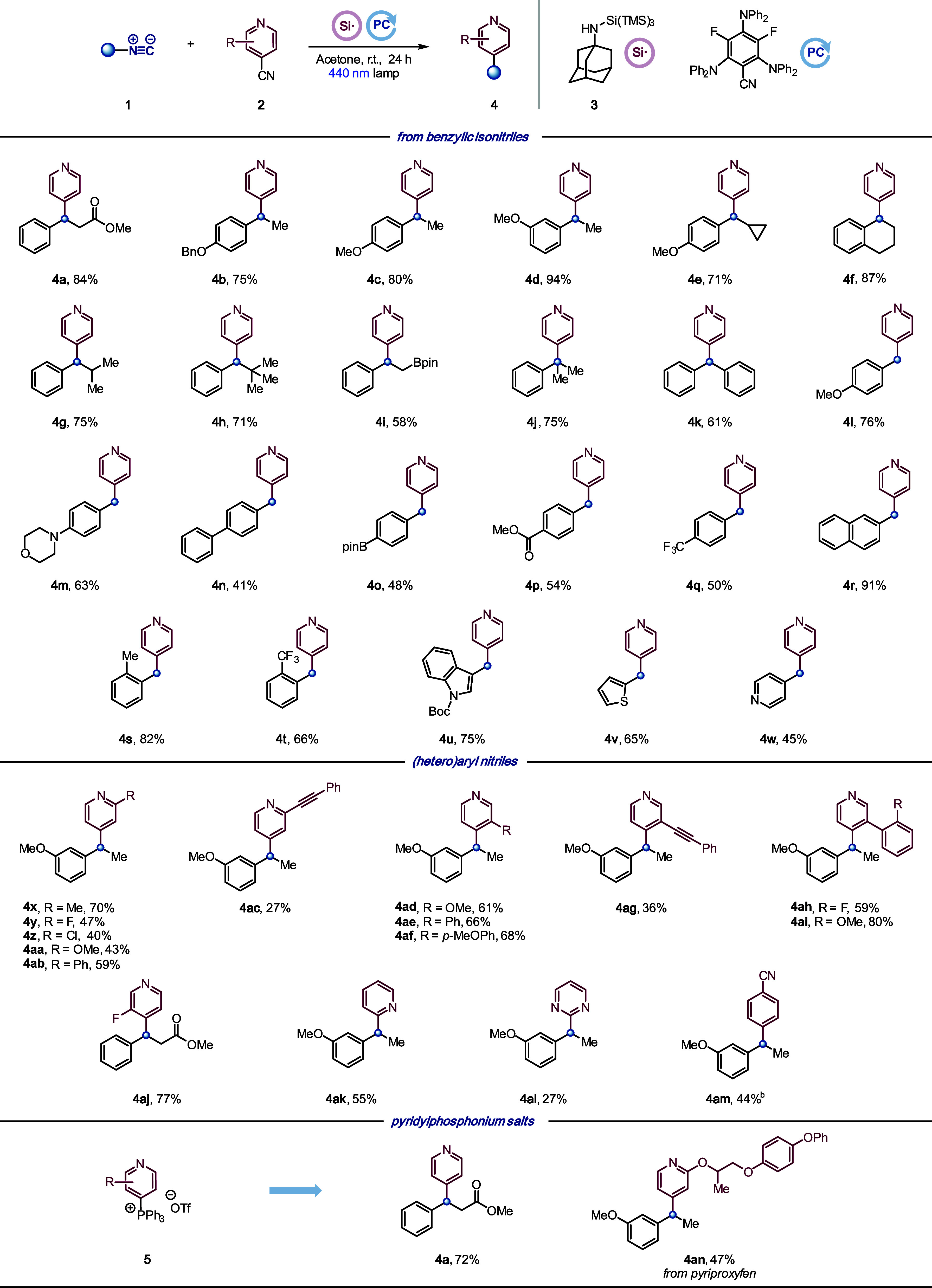
Substrate Scope: Benzylic Isonitriles All yields are isolated. Reaction conditions: **1** (0.20 mmol), **2** (0.30 mmol), aminosilane reagent **3** (0.26 mmol), and **PC** (5 mol %) were irradiated by blue Kessil lamp (440 nm) in acetone (0.1 M) at rt for 24 h. See the Supporting Information for details.

The reaction scope revealed as a general trend that electron-donating substituents on the aromatic ring afforded the corresponding products in higher yields than those bearing electron-withdrawing groups. The steric hindrance in the alpha-position of the alkyl radical (**4g**, **4h**) or ortho substituents (**4f**, **4s**, **4t**) on the aryl ring did not affect the yield. The presence of the morpholine moiety (**4m**), which is often not compatible with other photocatalytic conditions due to oxidative degradation in the alpha-position of the tertiary amine, was not affected under our reaction conditions, thereby highlighting the broad functional group compatibility. Isonitriles bearing an alkyl or aromatic boronic ester were also successfully used, which provided a handle for further functionalization (**4i**, **4o**). Moreover, indol (**4u**), thiophenyl (**4v**), and pyridyl (**4w**)-derived isonitriles afforded the heteroarylation products in moderate to good yields.

We then applied the optimized conditions to different 4-cyanopyridines to obtain the corresponding 2,4-disubstituted (**4x**–**4ac**) and 3,4-disubstituted (**4ad**–**4aj**) products. We were also pleased to find that the reaction took place with 2-cyanopyridine, 2-cyanopyrimidine, and 1,4-dicyanobenzene as coupling partners to provide compounds **4ak**–**4am** in moderate yields. Moreover, we briefly explored the possibility of using phosphonium salts **5** instead of cyanopyridines as coupling partners.^52^ We observed that model substrate **1a** provided the coupling product **4a** with good efficiency. Conveniently, the use of phosphonium salts **5** provides access to more complex pyridines, such as pyriproxyfen derivative **4an**.

More challenging aliphatic isonitriles were also suitable substrates (Scheme 4). As before, the reaction was general for Cα-primary (**4bg**–**4bi**), secondary (**4ao**–**4aq**, **4bf**) and tertiary (**4ar**–**4ax**, **4be**) isonitriles, including an isonitrile with a Cα-heteroatom (**4ay**). Isonitriles derived from phenylalanine, lysine, methionine, and unprotected tyrosine and tryptophan afforded the corresponding α-pyridyl esters (**4az**–**4bd**). Finally, the selective C–N cleavage was tested with different isonitriles prepared from bioactive molecules, showcasing the potential to apply the method in late-stage functionalization (**4be**–**4bi**).

**Scheme 4:**
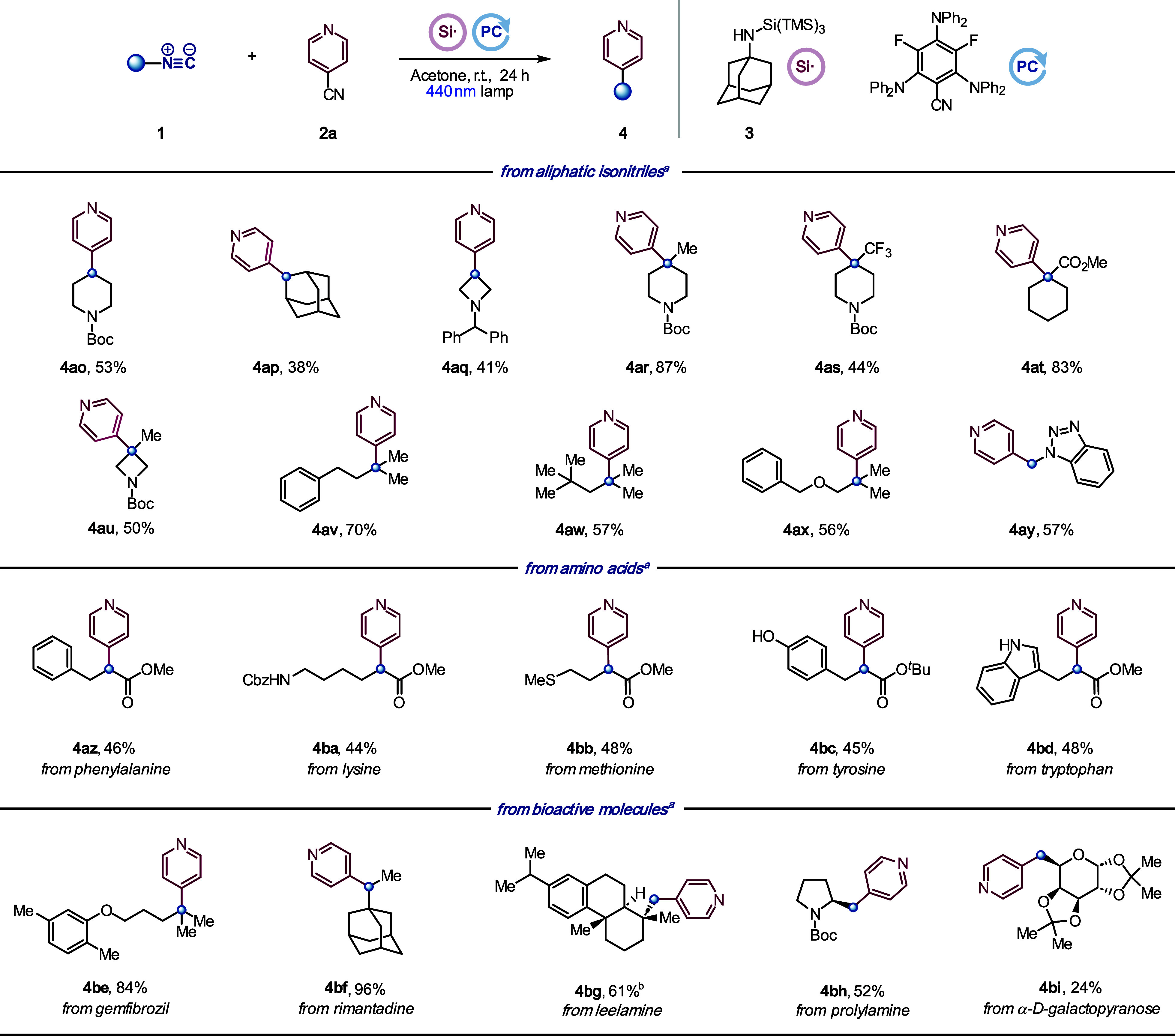
Substrate Scope: Aliphatic Isonitriles, Amino Acid Derivatives, and Bioactive Molecules All yields are isolated. Reaction conditions: **1** (0.20 mmol), **2** (0.30 mmol), aminosilane reagent **3** (0.26 mmol), and **PC** (5 mol %) were irradiated by blue Kessil lamp (440 nm) in acetone (0.1 M) at rt for 24 h. Reaction time = 48 h.

To further prove the synthetic potential of the method, we prepared a bisisonitrile to study the double heteroarylation (Scheme 5A). Interestingly, adjusting the equivalents of the cyanopyridine, we could obtain the double or the monoarylation products **4bj** and **4bk** selectively. A second sequential heteroarylation from **4bk** provided product **4bl** with two different pyridine rings.

**Scheme 5:**
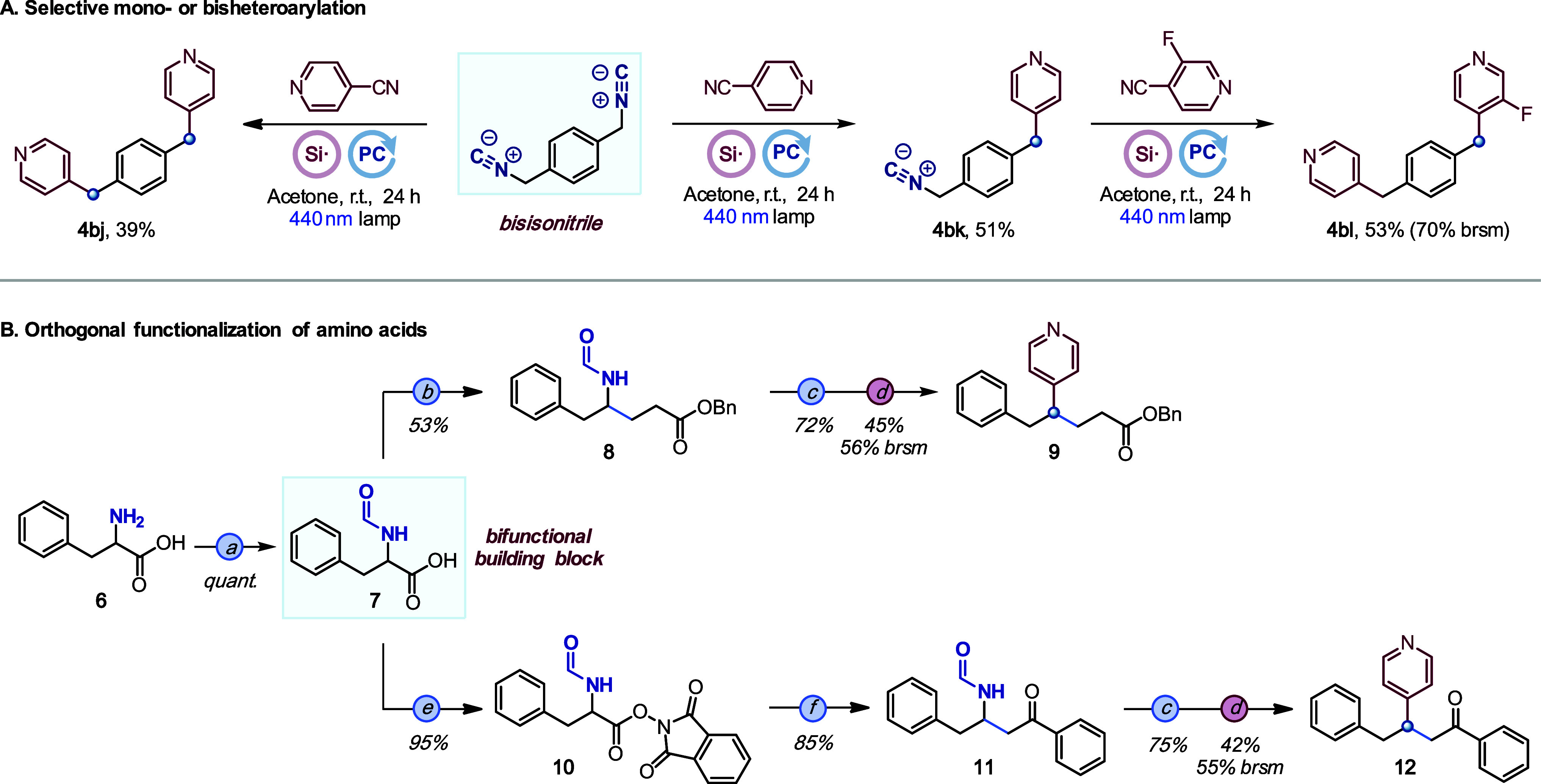
Heteroarylation of Bisisonitriles and Orthogonal Functionalization of Amino Acids All yields are isolated. (a) Formic acid, DMF (reflux). (b) Benzyl acrylate, K_2_HPO_4_, {Ir[dF(CF_3_)ppy]_2_(dtbbpy)}PF_6_, DMF, 440 nm lamp. (c) POCl_3_, Et_3_N, DCM. (d) **2a**, aminosilane reagent **3**, PC, acetone, 440 nm lamp. (e) *N*-Hydroxyphthalimide, DMAP, EDC·HCl, DCM. (f) Trimethyl[(1-phenylvinyl)oxy]silane, PPh_3_, NaI, MeCN, 457 nm lamp. See the Supporting Information for details.

One interesting feature of the use of isonitriles as amine derivatives in this transformation is their ability to promote C–N cleavage in amino acid derivatives, which provides an opportunity for orthogonal functionalization of the C–C and C–N bonds (Scheme 5B). We have illustrated this idea with phenyl alanine derivative **7** in which the formamide serves as a precursor for the isonitrile and as a protecting group for the selective functionalization of the carboxylic acid moiety. Indeed, the decarboxylative alkylation of formamide **7** under photoredox-catalyzed conditions^53^ provided compound **8** in 53% yield. Then, the isonitrile moiety was unmasked by dehydration of formamide **8** and further subjected to our heteroarylation conditions to yield pyridine derivative **9**. Following a similar strategy, redox-active ester **10** was prepared from formamide **7** and used in an alkylation with a silyl enol ether^54^ followed by a dehydration–heteroarylation sequence to provide ketone **12**.

In summary, we have developed the first protocol that uses isonitriles as alkyl radical precursors in a photoredox-catalyzed transformation involving selective C–N cleavage and subsequent C–C bond formation. This transformation allows for the interconversion of readily available isonitriles into functionalized pyridines under mild conditions in the presence of an organic photocatalyst and a silyl amine. Mechanistic studies suggest a catalytic cycle with reductive quenching involved. Importantly, the reaction can be used to functionalize primary, secondary, and tertiary substrates both benzylic and nonbenzylic, thereby expanding significantly the current structural scope. Moreover, we envision that this study will enable the development of further photocatalyzed transformations using isonitriles to selectively cleave and functionalize C–N bonds.

## Supporting Information Available

Experimental procedures, mechanistic studies, and characterization data (PDF)
