# Supplementary material for: Csp3–Csp2 Coupling of Isonitriles and (Hetero)arenes through a Photoredox-Catalyzed Double Decyanation Process
Source: ACS Catal. 2024 Nov 8;14(23):17286–92. doi: 10.1021/acscatal.4c06269 (PMC11629295; doi:10.1021/acscatal.4c06269)

Supporting Information

Csp^3 – Csp^2 Coupling of Isonitriles and (Hetero)arenes through a Photoredox-Catalyzed Double Decyanation Process

María Martín,[a] R. Martín Romero,[a] Chiara Portolani,[a,c] Mariola Tortosa[a,b]*

[a]*Organic Chemistry Department and Center for Innovation in Advanced Chemistry (ORFEO-CINQA), Universidad Autónoma de Madrid, Madrid 28049, Spain.*

[b]*Institute for Advanced Research in Chemical Sciences (IAdChem), Universidad Autónoma de Madrid, Madrid 28049, Spain.*

[c]*Department of Industrial Chemistry "Toso Montanari", Alma Mater Studiorum-University of Bologna, via P. Gobetti 85, 40129 Bologna, Italy.*

*To whom correspondence should be addressed. E-mail: mariola.tortosa@uam.es

TABLE OF CONTENT

1. General Considerations 3
2. General Procedures for the Synthesis of Starting Materials 4
  A. Synthesis of Isonitriles 4
    A. 1. Synthesis of Primary Amines 5
    A. 2. General Procedure A for Isonitriles Synthesis from Primary Amines 9
    A. 3. General Procedure B for Isonitriles Synthesis from Primary Amines 9
    A. 4. General Procedure C for Isonitriles Synthesis from Aldehydes 10
    A. 5. Synthesis of 4-(isocyanomethyl)pyridine, 1x 11
    A. 6. Characterization Data of Isonitriles 11
  B. Synthesis of Substituted (Hetero)aryl Nitriles and Pyridylphosphonium Salts 17
    B. 1. General Procedure D for the Synthesis of Cyano-Pyridines 2j, 2l and 2m 17
    B. 2. General Procedure E for the Synthesis of Cyano-Pyridines 2g and 2k 19
  C. Synthesis of 3DPA2FBN (PC) 21
  D. Synthesis of $TMS_3SiNHAd$ (3) 21
3. Optimization of the Reaction Conditions 22
  A. General Conditions for Optimization 22
4. Photocatalyzed Deaminative Arylation 25
  A. Experimental Set-up 25
  B. General Procedure F 25
  C. Characterization Data 26
  D. Gram-Scale Experiment 55
5. Orthogonal Functionalization of Amino Acids 56
6. Competition Studies: XAT vs Heteroarylation and Decarboxylation vs Heteroarylation 61
7. Mechanistic Studies 62
  A. Stern-Volmer Quenching Studies 62
    B. 1. Stern-Volmer Quenching Studies for 3DPA2FBN 62
    B. 2. Stern-Volmer Quenching Studies for $Ir[(ppy)_2(dtbbpy)]PF_6$ 65
  B. Proposed Mechanism for the Deaminative Arylation 68
  C. Quantum Yield Measurement 69
  D. TEMPO Radical-Trapping Experiment 73
  E. Detection of R_3SiCN 73
8. References 76
9. NMR spectra 80

1. General Considerations

1.1 General: For light irradiation, a Kessil PR160L-blue LED lamp (max 45 W High Luminous DEX 2100 LED, λ_{max} = 440 nm) were placed 5 cm away from the reaction vials. NMR spectra (^{1}H, ^{13}C, ^{11}B, ^{19}F) were obtained at 298 K using 300 and 500 MHz spectrometers. All chemical shifts in NMR experiments are reported as ppm downfield from TMS. The following calibrations were used: $CDCl_3$ δ = 7.26 and 77.2 ppm, DMSO-d_6 δ = 2.50 and 39.5 ppm, CD_2Cl_2 δ = 5.32 and 53.8 ppm, C_6D_6 δ = 7.16 and 128.1 ppm, $C_2D_2Cl_4$ δ = 5.91 and 74.2 ppm. Reactions were monitored by ^{1}H NMR, and/or TLC on silica gel plates (60 Å porosity, 250 μm thickness). TLC analysis was performed using hexanes/EtOAc as the eluent and visualized using phosphomolybdic acid, ninhydrin, *p*-anisaldehyde stain, and/or UV light. Chromatography purification was accomplished using a silica gel 40-63 μm. High Resolution Mass Spectrometry (HRMS) were registered in a spectrometer Bruker maXis IITM (Q-TOF) or a GCT Agilent Technologies 6890 N using Electronic Impact (EI^{+}) techniques at 70 eV. Melting points (ºC) were determined in a Stuart™ melting point SMP3 apparatus in open capillary tubes, and are uncorrected. UV/vis studies and fluorescence measurements were measured in a 1 cm quartz cuvette using a JASCO V-660 UV/vis spectrophotometer and a JASCO FP-8600 spectrofluorometer, respectively.

1.2 Chemicals: Deuterated NMR solvents were purchased and used as received. Dry acetone, DMA, DMF, DMSO, THF and CH_2Cl_2 were obtained from Acros Organics or Merck, and used as received. MeCN was purchased and dried *via* a solvent delivery system. Photoredox-catalyzed reactions were performed using 4 mL Screw Neck Vial (clear glass, 45 x 14.7 mm) with screw cap 13 mm black Sil/PTFE septa. All the reagents were purchased from commercial suppliers and used as received.

2. General Procedures for the Synthesis of Starting Materials

A. Synthesis of Isonitriles

Isonitriles (***Figure S1***) were prepared according to literature procedures[1-10] (**1a**, **1b**, **1k**, **1y-1ab**, **1ad**, **1ae**, **1aj-1an**, **1ap**: ref. 1; **1c**: ref. 2; **1f**: ref. 3; **1i**, **1o**: ref. 4; **1j**, **1l**, **1m**, **1aq**: ref. 5; **1n**: ref. 6; **1r**, **1s**: ref. 7; **1u**: ref. 8; **1v**: ref. 9; **1as**: ref. 10), purchased from suppliers, or synthesized as indicated below.

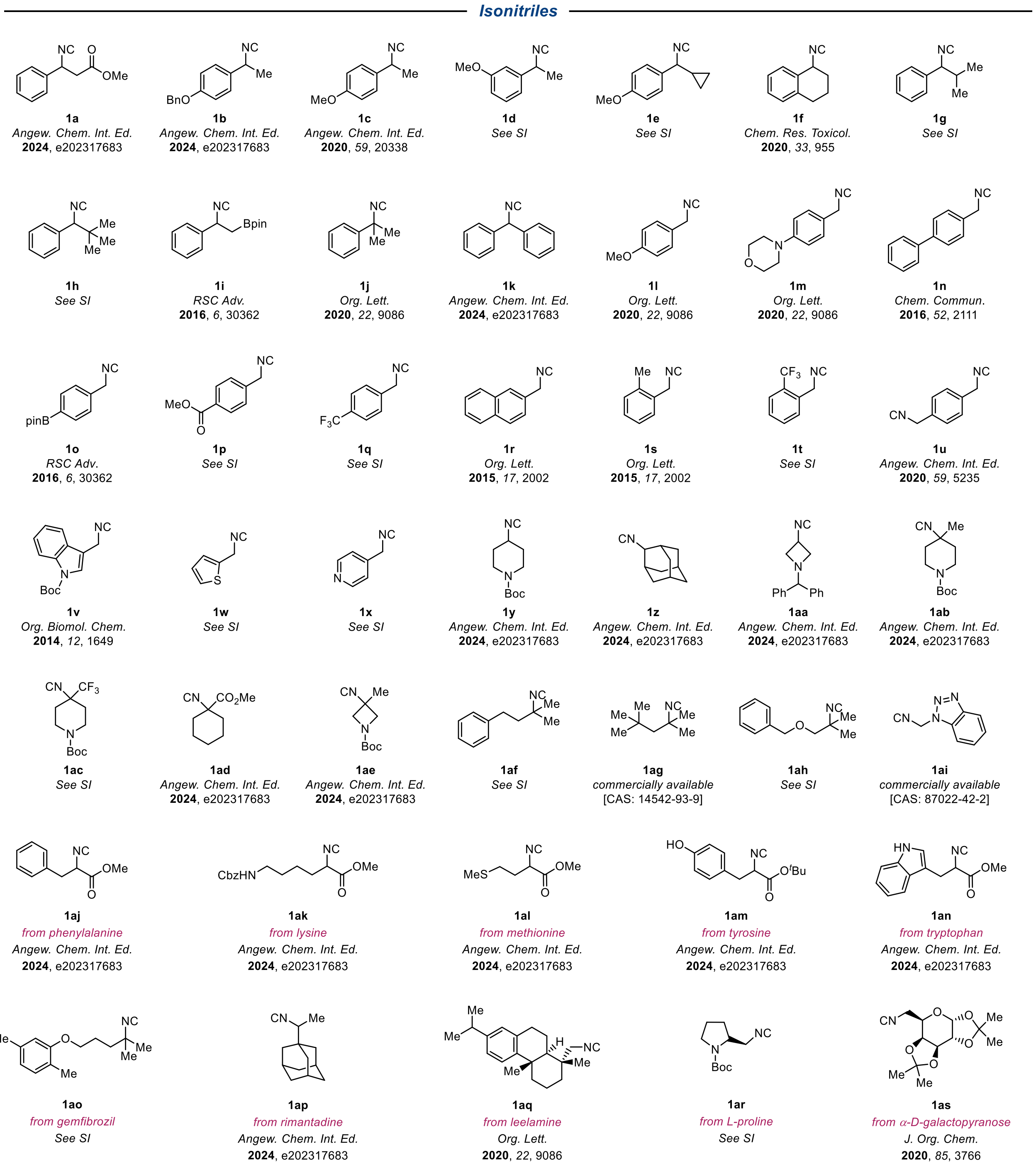

Figure S1: Synthesis of starting isonitriles.

A. 1. Synthesis of Primary Amines

A. 1. 1. Synthesis of cyclopropyl(4-methoxyphenyl)methanamine, SI-4

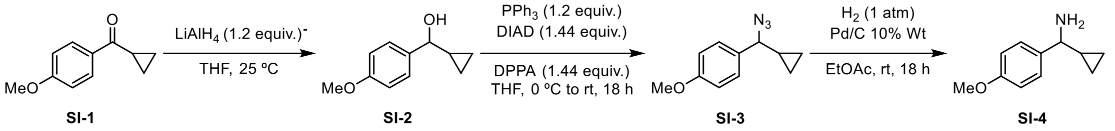

Step 1: To a solution of cyclopropyl(4-methoxyphenyl)methanone **SI-1** (344.2 mg, 1.95 mmol, 1.00 equiv.) in THF (1.4 mL), $LiAlH_4$ (1.18 mL, 2.34 mmol, 1.2 equiv.) was added dropwise at 0 °C. After addition, the reaction mixture was slowly warmed to r.t. and allowed to stir overnight. The mixture was quenched with NaOH (1 M) dropwise, filtrate though a plug of $MgSO_4$ and the solvent was removed under reduced pressure. The crude was purified by flash column chromatography (SiO_2; 0 – 40% EtOAc in cyclohexane) to afford pure product **SI-2** as a colorless oil (283.9 mg, 1.59 mmol) in 82% yield. Spectroscopic data were in agreement with those described in the literature.[11]

Step 2: In a flamed and dried Schlenk flask under argon, triphenylphosphine (501.4 mg, 1.91 mmol, 1.2 equiv.), **SI-2** (283.9 mg, 1.59 mmol, 1.0 equiv.) and THF (6.4 mL) were added. At 0 °C, diisopropyl azodicarboxylate (463.8 mg, 2.29 mmol, 1.44 equiv.) was added. After 5 minutes, diphenyl phosphorazidate (631.2 mg, 2.29 mmol, 1.44 equiv.) was added and the final mixture was stirred at r.t. overnight. The crude reaction was concentrated and purified by flash column chromatography (SiO_2; 0 – 3% EtOAc in cyclohexane) to afford pure product **SI-3** as a colorless oil (202.9 mg, 1.00 mmol) in 63% yield.

^{1}H NMR (300 MHz, $CDCl_3$): δ 7.32 – 7.27 (m, 2H), 6.94 – 6.88 (m, 2H), 3.83 (d, J = 8.3 Hz, 1H), 3.82 (s, 3H), 1.36 – 1.21 (m, 1H), 0.81 – 0.67 (m, 1H), 0.63 – 0.49 (m, 2H), 0.38 – 0.25 (m, 1H). *Spectrum*

^{13}C NMR (75 MHz, $CDCl_3$): δ 159.4, 131.9, 128.2, 114.0, 69.4, 55.3, 16.4, 4.4, 2.6. *Spectrum*

HRMS (ESI+): calculated for $C_{11}H_{13}N_3NaO$ $[M+Na]^+$: 226.0951; found: 226.0950.

Step 3: In a round bottom flask palladium on carbon (20.0 mg, 0.02 mmol, 10% w/w, 0.02 equiv. of palladium) was added followed by a solution of **SI-3** (200.0 mg, 0.98 mmol, 1.0 equiv.) on EtOAc (4.9 mL). Finally, the reaction mixture was purged with hydrogen, and it was stirred overnight under hydrogen atmosphere (1 atm). Upon reaction completion, the mixture was filtrate through a plug of celite and concentrate to obtain the desired pure amine **SI-4** in 99% yield.

^{1}H NMR (300 MHz, $CDCl_3$): δ 7.32 (d, J = 8.6 Hz, 2H), 6.88 (d, J = 8.7 Hz, 2H), 3.80 (s, 3H), 3.17 (d, J = 8.6 Hz, 1H), 1.57 (br s, 2H), 1.15 – 1.01 (m, 1H), 0.64 – 0.53 (m, 1H), 0.51 – 0.41 (m, 1H), 0.35 – 0.19 (m, 2H). *Spectrum*

^{13}C NMR (75 MHz, $CDCl_3$): δ 158.5, 137.9, 127.4, 113.6, 60.1, 55.2, 19.9, 3.9, 3.0. *Spectrum*

HRMS (ESI+): calculated for $C_{11}H_{16}NO$ $[M+H]^+$: 178.1226; found: 178.1221.

A. 1. 2. Synthesis of *tert*-butyl 4-amino-4-(trifluoromethyl)piperidine-1-carboxylate, SI-8

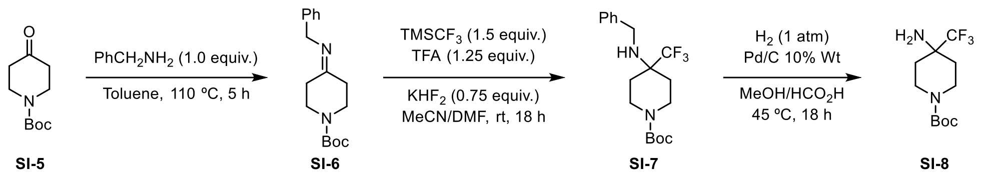

Step 1: *According to the literature procedure,*[12] *tert*-butyl 4-oxopiperidine-1-carboxylate **SI-5** (2.0 g, 10.0 mmol, 1.0 equiv.) and benzylamine (1.1 mL, 10.0 mmol, 1.0 equiv.) were stirred in toluene (50.0 mL) for 5 h under reflux in a round-bottom flask with an attached Dean-Stark trap. The reaction mixture was cooled to r.t., and the solvent was removed under reduced pressure to afford the corresponding **SI-6** product as a yellow oil that was directly used in the next step without further purification.

Step 2: *According to the literature procedure,*[12] in a round bottom flask, **SI-6** (2.88 g, 10.0 mmol, 1.0 equiv.), potassium hydrogenfluoride (585.7 mg, 7.50 mmol, 0.75 equiv.) were stirred in a mixture of MeCN (20.0 mL) and DMF (2.3 mL) under nitrogen atmosphere. The mixture was cooled to 0 ºC and TFA (0.96 mL, 12.50 mmol, 1.25 equiv.) was added dropwise. After 5 minutes, $TMSCF_3$ (2.2 mL, 15.0 mmol, 1.5 equiv.) was added and the reaction mixture was warmed to r.t. and stirred overnight. The resulting mixture was quenched with saturated Na_2CO_3. The aqueous layer was extracted with 1:1 hexane/Et_2O (x3). The combined organic layers were dried over anhydrous $MgSO_4$ and concentrated under reduced pressure. The crude was purified by flash column chromatography (SiO_2; 0 – 10% EtOAc in cyclohexane) to afford pure product **SI-7** as a colorless oil (1.1 g, 3.0 mmol) in 30% yield. Spectroscopic data were in agreement with those described in the literature.[12]

Step 3: *According to a modified literature procedure,*[12] in a round bottom flask palladium on carbon (200.0 mg, 0.19 mmol, 10% w/w, 0.06 equiv. of palladium) was added followed by a solution of **SI-7** (1.1 g, 3.01 mmol, 1.0 equiv.) in a mixture of MeOH (18.4 mL) and HCO_2H (0.5 mL). Finally, the reaction mixture was purged with hydrogen, and it was stirred at 45 ºC overnight under hydrogen atmosphere (1 atm). Upon reaction completion, the mixture was filtrate through a plug of celite, washed with MeOH and the solvent was removed under reduced pressure.

Afterwards, K_2CO_3 (sat.) was added, the aqueous layers were extracted with DCM (x3) and the combined organic layers were dried over anhydrous Na_2SO_4. Then, the solvent was removed in vacuo to obtain the desired pure amine **SI-8** in 92% yield. Spectroscopic data were in agreement with those described in the literature.[12]

A. 1. 3. Synthesis of 2-methyl-4-phenylbutan-2-amine, SI-12

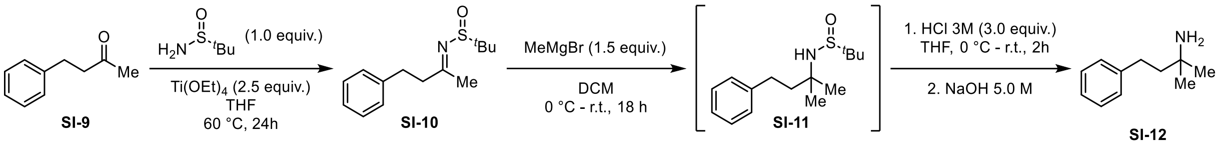

Step 1: *According to a modified literature procedure*,[13] to a round bottom flask with stirring bar, 4-phenylbutan-2-one **SI-9** (3.00 mL, 20.0 mmol, 1.0 equiv.), 2-methylpropane-2-sulfinamide (2.42 g, 20.0 mmol, 1.0 equiv.), titanium ethoxide (10.3 mL, 50.0 mmol, 2.5 equiv.) and THF (38.0 mL) were added successively under argon atmosphere. After heating at 60 °C for 24 h, the mixture was allowed to cool to r.t. and quenched with brine. The slurry was left stirring for 30 min at r.t. and the insoluble materials were removed by filtration through a pad of Celite. The resulting solution was concentrated under reduced pressure. The crude was purified by flash column chromatography (SiO_2; 10 – 80% EtOAc in cyclohexane) to afford pure product **SI-10** as a colorless oil (1.98 g, 7.92 mmol) in 39% yield. Spectroscopic data were in agreement with those described in the literature.[14]

Step 2: *According to a modified literature procedure*,[13] to an oven dried Schlenk flask equipped with magnetic stir bar under argon was added **SI-10** (1.98 g, 7.92 mmol, 1 equiv.) and dry DCM (16.0 mL). Then, methylmagnesium bromide (4.0 mL, 3.0 M solution in Et_2O, 11.88 mmol, 1.5 equiv.) was added dropwise at 0 °C and the mixture was allowed to warm to r.t. and stirred 18 h. The reaction was quenched at 0 °C with the dropwise addition of sat. NH_4Cl (aq), then extracted with DCM. The combined organic layers were dried over anhydrous $MgSO_4$ and concentrated under reduced pressure to afford **SI-11**.

Step 3: *According to a modified literature procedure*,[13] the residue **SI-11** was dissolved in THF, and the mixture was cooled to 0 °C. HCl 3.0 M was added dropwise and the mixture was stirred for 2 h. The solvent was then removed in vacuo and the suspension in water was washed with Et_2O. The aqueous phase was basified to pH = 12 with 5.0 M NaOH and extracted with DCM. The combined organic layers were washed with saturated $NaHCO_3$ (aq), dried over anhydrous $MgSO_4$ and concentrated under reduced pressure to obtain the desired pure amine **SI-12** in 31% yield. Spectroscopic data were in agreement with those described in the literature.[15]

A. 1. 4. Synthesis of 1-(benzyloxy)-2-methylpropan-2-amine, SI-14

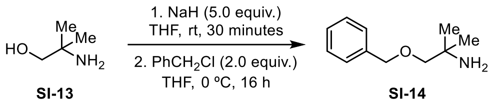

According to a modified literature procedure,[16] a solution of 2-amino-2-methylpropan-1-ol **SI-13** (1.34 g, 15.0 mmol, 1.0 equiv.) on THF (42.5 mL) was added dropwise to a slurry of sodium hydride dispersion in mineral oil (3.0 g, 75.0 mmol, 60% w/v, 5.0 equiv.) in THF (37.5 mL). The mixture was stirred at r.t. for 30 minutes and then cooled to 0 ºC. Then, (chloromethyl)benzene (3.8 mL, 30.0 mmol, 2.0 equiv.) was added dropwise. After stirring for 16 hours, the reaction mixture was poured into ice/water and extracted with EtOAc. The combined organic layers were dried over anhydrous $MgSO_4$ and concentrated under reduced pressure. The crude was purified by flash column chromatography (SiO_2; 0 – 10% MeOH in DCM) to afford pure product **SI-14** as a colorless oil (664.6 mg, 3.7 mmol) in 25% yield.

A. 1. 5. Synthesis of 5-(2,5-dimethylphenoxy)-2-methylpentan-2-amine, SI-17

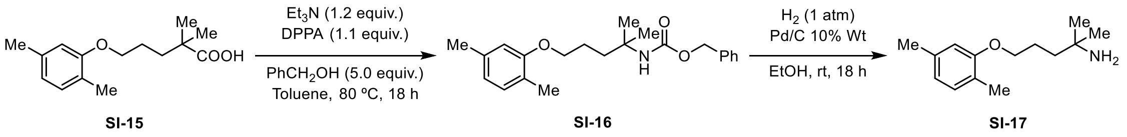

Step 1: *According to the literature procedure,*[17] gemfibrozil **SI-15** (1.25 g, 5.00 mmol, 1.0 equiv.), triethylamine (0.84 mL, 6.00 mmol, 1.2 equiv.) and diphenyl phosphorazidate (1.51 g, 5.50 mmol, 1.1 equiv.) were stirred in toluene (50.0 mL) for 1 h at r.t. After this time, phenylmethanol (2.6 mL, 25.0 mmol, 5.0 equiv.) was added and the reaction mixture was stirred at 80 ºC for 18 h. The crude reaction was concentrated and purify by flash column chromatography (SiO_2; 0 – 10% EtOAc in cyclohexane) to afford pure product **SI-16** as a slightly yellow oil (1.12 g, 3.15 mmol) in 63% yield.

^{1}H NMR (300 MHz, $CDCl_3$): δ 7.42 – 7.31 (m, 5H), 7.04 (d, J = 7.4 Hz, 1H), 6.70 (d, J = 7.6 Hz, 1H), 6.65 (s, 1H), 5.09 (s, 2H), 4.78 (br s, 1H), 3.96 (t, J = 5.8 Hz, 2H), 2.35 (s, 3H), 2.22 (s, 3H), 1.91 – 1.78 (m, 4H), 1.37 (s, 6H). *Spectrum*

^{13}C NMR (75 MHz, $CDCl_3$): δ 157.0, 154.7, 136.8, 136.5, 130.4, 128.6, 128.13, 128.11, 123.6, 120.8, 112.1, 68.0, 66.1, 52.8, 36.9, 27.2, 24.4, 21.5, 15.9. *Spectrum*

Step 2: *According to the literature procedure,*[17] in a round bottom flask palladium on carbon (299.5 mg, 0.28 mmol, 10% w/w, 0.10 equiv. of palladium) was added followed by a solution of **SI-16** (1.0 g, 2.81 mmol, 1.0 equiv.) on EtOH (20.0 mL). Finally, the reaction mixture was purged

with hydrogen, and it was stirred overnight under hydrogen atmosphere (1 atm). Upon reaction completion, the mixture was filtrate through a plug of celite and concentrate to obtain the desired pure amine **SI-17** in 89% yield.

^{1}H NMR (300 MHz, $CDCl_3$): δ 6.99 (d, *J* = 7.5 Hz, 1H), 6.65 (d, *J* = 7.5 Hz, 1H), 6.60 (s, 1H), 4.10 (br s, 2H), 3.94 (t, *J* = 6.2 Hz, 2H), 2.30 (s, 3H), 2.17 (s, 3H), 1.93 – 1.81 (m, 2H), 1.70 – 1.60 (m, 2H), 1.24 (s, 6H). Spectrum

^{13}C NMR (75 MHz, $CDCl_3$): δ 156.9, 136.4, 130.3, 123.6, 120.7, 112.0, 68.0, 50.6, 40.3, 29.1, 24.6, 21.4, 15.8. Spectrum

HRMS (ESI+): calculated for $C_{14}H_{24}NO$ $[M+H]^+$: 222.1852; found: 222.1857.

A. 2. General Procedure A for Isonitriles Synthesis from Primary Amines

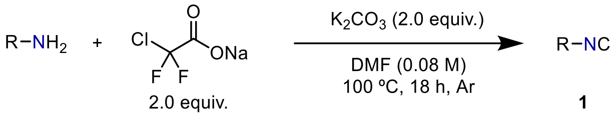

According to the literature procedure,[18] in an oven-dried Schlenk tube equipped with a magnetic stir bar were added the primary amine (1.0 equiv.), sodium chlorodifluoroacetate (2.0 equiv.) and K_2CO_3 (2.0 equiv.). The Schlenk tube was evacuated and refilled with argon before dry DMF (0.08 M) was added via a syringe. The reaction mixture was stirred under an argon atmosphere at 100 ºC overnight. After the completion of the reaction, the mixture was then allowed to cool to r.t. and extracted with EtOAc. The combined organic layers were washed with brine (x3), dried over anhydrous $MgSO_4$ and concentrated to dryness under reduced pressure. The crude product was purified by flash column chromatography on silica gel to afford pure product **1**.

A. 3. General Procedure B for Isonitriles Synthesis from Primary Amines

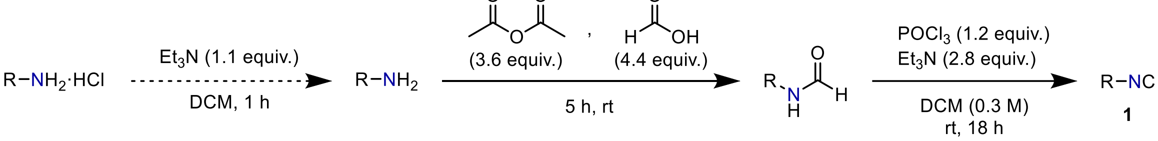

According to the literature procedure,[19] triethylamine (1.1 equiv.) was added to a solution of amine hydrochloride (1.0 equiv.) in DCM. The mixture was stirred under argon for 1 h. The white solid was filtered off and the organic layer was washed with water and was dried with Na_2SO_4. The solvent was removed in vacuo to give the free amine as a coloured oil. This step is

only necessary if starting from the ammonium salt. Acetic anhydride (3.6 equiv.) and formic acid (4.4 equiv.) were stirred for 2 h at 55 °C to prepare formic acetic anhydride. The free amine was cooled to 0 °C and dissolved in a large excess of formic acetic anhydride. The mixture was stirred for 15 min to 0 °C and then, the reaction was allowed to warm to r.t. and stirred a further 5 h. The solvents were removed in vacuo and the resulting oil was partitioned between water and DCM. The aqueous layers were extracted with DCM (x3) and the combined organic layers were dried with anhydrous Na_2SO_4. Then, the solvent was removed in vacuo to afford the corresponding formamide that was directly used in the next step without further purification.

Then, $POCl_3$ (1.2 equiv.) was added dropwise to a solution of the formamide (1.0 equiv.) in Et_3N (2.8 equiv.) and DCM (0.3 M) at –25 ºC. The reaction mixture was stirred for 12 h at r.t., and the resulting red mixture was quenched with $NaHCO_3$. The aqueous layer was extracted with DCM (x3), the combined organic layers were dried over anhydrous $MgSO_4$ and concentrated under reduced pressure. The crude was purified by flash column chromatography on silica gel to afford pure product **1**.

A. 4. General Procedure C for Isonitriles Synthesis from Aldehydes

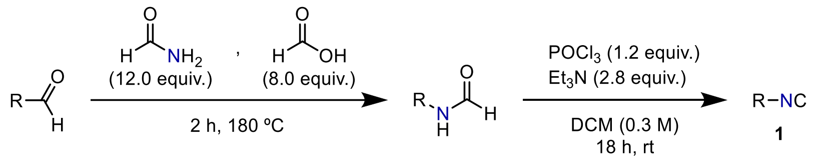

According to the literature procedure,[20] in a flask equipped with a magnetic stir bar were added the corresponding carbonyl compound (1.0 equiv.), formamide (12.0 equiv.) and formic acid (8.0 equiv.). The flask was equipped with a condenser and the reaction mixture was stirred at 180 ºC for 2 hours. The reaction was cooled down and water was added. The mixture was extracted with DCM (x3) and the combined organic layers were dried with anhydrous Na_2SO_4. Then, the solvent was removed in vacuo to afford the corresponding formamide that was directly used in the next step without further purification.

Then,[19] $POCl_3$ (1.2 equiv.) was added dropwise to a solution of the formamide (1.0 equiv.) in Et_3N (2.8 equiv.) and DCM (0.3 M) at –25 ºC. The reaction mixture was stirred for 12 h at r.t., and the resulting red mixture was quenched with $NaHCO_3$. The aqueous layer was extracted with DCM (x3), the combined organic layers were dried over anhydrous $MgSO_4$ and concentrated under reduced pressure. The crude was purified by flash column chromatography on silica gel to afford pure product **1**.

A. 5. Synthesis of 4-(isocyanomethyl)pyridine, 1x

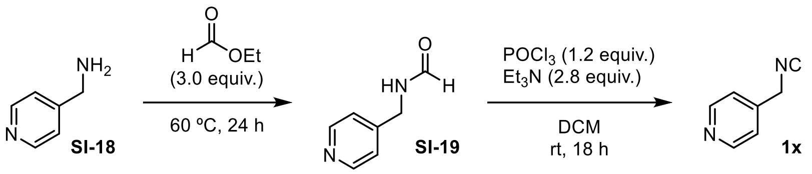

Step 1: *According to the literature procedure*,[21] pyridine-4-methanamine **SI-18** (1.1 g, 10.0 mmol, 1.0 equiv.) and ethyl formate (2.4 mL, 30.0 mmol, 3.0 equiv.) were stirred at 60 ºC for 24 h under nitrogen atmosphere. The reaction mixture was cooled to r.t., and the solvent was removed under reduced pressure to afford the corresponding **SI-19** product as a brown oil that was directly used in the next step without further purification.

Step 2: *According to the literature procedure*,[19] $POCl_3$ (1.2 equiv.) was added dropwise to a solution of the **SI-19** (1.0 equiv.) in Et_3N (2.8 equiv.) and DCM (0.3 M) at –78 ºC. The reaction mixture was stirred for 18 h at r.t., and the resulting mixture was quenched with $NaHCO_3$. The aqueous layer was extracted with DCM (x3), the combined organic layers were dried over anhydrous $MgSO_4$ and concentrated under reduced pressure. The crude was purified by flash column chromatography (SiO_2; 0 – 10% *i*PrOH in DCM) to afford pure product **1x** (121.0 mg, 1.02 mmol) in 10% yield as a yellow oil. $\mathbf{R_f}$ = 0.30 (9:1 DCM/MeOH).

^{1}H NMR (300 MHz, $CDCl_3$): δ 8.67 (d, J = 5.9 Hz, 2H), 7.30 (d, J = 5.3 Hz, 2H), 4.69 (s, 2H). *Spectrum*

^{13}C NMR (75 MHz, $CDCl_3$): δ 160.0 (t, J = 4.8 Hz, -N**C**), 150.6, 141.1, 121.2, 44.7 (t, J = 7.7 Hz, **C**-NC). *Spectrum*

HRMS (ESI+): calculated for $C_7H_7N_2$ $[M+H]^+$: 119.0604; found: 119.0607.

A. 6. Characterization Data of Isonitriles

1-(1-Isocyanoethyl)-3-methoxybenzene (1d)

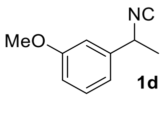

Prepared following the general procedure A, the title compound (174.3 mg, 1.08 mmol) was obtained in 82% yield as a yellowish oil, after purification by flash column chromatography (SiO_2; 0 – 5% EtOAc in cyclohexane). $\mathbf{R_f}$ = 0.30 (9.5:0.5 cyclohexane/EtOAc).

^{1}H NMR (300 MHz, $CDCl_3$): δ 7.31 (t, *J* = 7.9 Hz, 1H), 6.95 – 6.85 (m, 3H), 4.83 – 4.76 (m, 1H), 3.83 (s, 3H), 1.68 (dt, *J* = 6.9, 2.2 Hz, 3H). Spectrum

Spectroscopic data were in agreement with those described in the literature.[22]

1-(Cyclopropyl(isocyano)methyl)-4-methoxybenzene (1e)

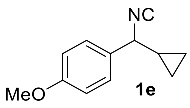

1e

Prepared following the general procedure A, the title compound (83.3 mg, 0.45 mmol) was obtained in 45% yield as a yellowish oil from amine **SI-4**, after purification by flash column chromatography (SiO_2; 0 – 5% EtOAc in cyclohexane). $\mathbf{R_f}$ = 0.41 (9:1 cyclohexane/EtOAc).

^{1}H NMR (300 MHz, $CDCl_3$): δ 7.34 – 7.29 (m, 2H), 6.93 – 6.90 (m, 2H), 4.26 (d, *J* = 7.4 Hz, 1H), 3.82 (s, 3H), 1.35 – 1.30 (m, 1H), 0.73 – 0.62 (m, 2H), 0.59 – 0.55 (m, 1H), 0.49 – 0.45 (m, 1H). Spectrum

^{13}C NMR (75 MHz, $CDCl_3$): δ 159.7, 156.3 (t, *J* = 4.6 Hz, -N**C**), 129.5, 127.4, 114.2, 61.6 (t, *J* = 6.1 Hz, **C**-NC), 55.4, 18.1, 4.0, 3.4. Spectrum

HRMS (ESI+): calculated for $C_{12}H_{13}NO$ $[M]^+$: 187.0992; found: 187.0996.

(1-Isocyano-2-methylpropyl)benzene (1g)

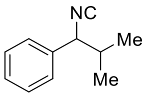

1g

Prepared following the general procedure B, the title compound (244.0 mg, 1.53 mmol) was obtained in 64% yield as an orange oil, after purification by flash column chromatography (SiO_2; 0 – 5% EtOAc in cyclohexane). $\mathbf{R_f}$ = 0.62 (4:1 cyclohexane/EtOAc).

^{1}H NMR (300 MHz, $CDCl_3$): δ 7.42 – 7.29 (m, 5H), 4.58 – 4.55 (m, 1H), 2.13 – 2.03 (m, 1H), 1.04 (d, *J* = 6.8 Hz, 3H), 0.95 (d, *J* = 6.8 Hz, 3H). Spectrum

^{13}C NMR (75 MHz, $CDCl_3$): δ 157.3 (t, *J* = 5.1 Hz, -N**C**), 136.4, 128.7, 128.2, 126.5, 65.3 (t, *J* = 6.2 Hz, **C**-NC), 35.1, 19.6, 16.9. Spectrum

HRMS (ESI+): calculated for $C_{11}H_{13}NNa$ $[M+Na]^+$: 182.0940; found: 182.0934.

(1-Isocyano-2,2-dimethylpropyl)benzene (1h)

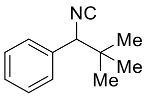

1h

Prepared following the general procedure A, the title compound (108.4 mg, 0.63 mmol) was obtained in 51% yield as a yellowish oil, after purification by flash

column chromatography (SiO_2; 0 – 5% EtOAc in cyclohexane). **R$_f$** = 0.33 (9.5:0.5 cyclohexane/EtOAc).

^{1}H NMR (300 MHz, $CDCl_3$): δ 7.38 – 7.32 (m, 3H), 7.30 – 7.28 (m, 2H), 4.41 – 4.40 (m, 1H), 1.01 (s, 9H). Spectrum

^{13}C NMR (75 MHz, $CDCl_3$): δ 156.9 (t, *J* = 4.9 Hz, -N**C**), 134.9, 128.4, 128.14, 128.13, 69.3 (t, *J* = 6.1 Hz, -NC), 35.8, 26.1. Spectrum

HRMS (ESI+): calculated for $C_{12}H_{15}NNa$ $[M+Na]^+$: 196.1097; found: 196.1094.

Methyl 4-(isocyanomethyl)benzoate (1p)

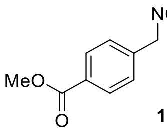

1p

Prepared following the general procedure B, the title compound (531.4 mg, 3.03 mmol) was obtained in 69% yield as a yellow oil, after purification by flash column chromatography (SiO_2; 0 – 20% EtOAc in cyclohexane). **R$_f$** = 0.25 (4:1 cyclohexane/EtOAc).

^{1}H NMR (300 MHz, $CDCl_3$): δ 7.97 (d, *J* = 8.6 Hz, 2H), 7.34 (d, *J* = 8.7 Hz, 2H), 4.65 (s, 2H), 3.84 (s, 3H). Spectrum

^{13}C NMR (75 MHz, $CDCl_3$): δ 166.2, 158.5 (t, *J* = 4.9 Hz, -N**C**), 137.0, 130.04, 129.98, 126.3, 52.1, 45.1 (t, *J* = 7.8 Hz, **C**-NC). Spectrum

HRMS (ESI+): calculated for $C_{10}H_9NNaO_2$ [M+Na]: 198.0525; found: 198.0523.

1-(Isocyanomethyl)-4-(trifluoromethyl)benzene (1q)

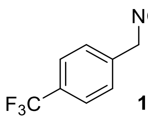

1q

Prepared following the general procedure C, the title compound (132.0 mg, 0.71 mmol) was obtained in 38% yield as a yellow oil, after purification by flash column chromatography (SiO_2; 0 – 15% EtOAc in cyclohexane). **R$_f$** = 0.32 (4:1 cyclohexane/EtOAc).

^{1}H NMR (300 MHz, $CDCl_3$): δ 7.67 (d, *J* = 8.2 Hz, 2H), 7.43 (d, *J* = 8.3 Hz, 2H), 4.71 (s, 2H). Spectrum

Spectroscopic data were in agreement with those described in the literature.[23]

1-(Isocyanomethyl)-2-(trifluoromethyl)benzene (1t)

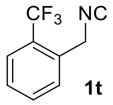

1t

Prepared following the general procedure C, the title compound (102.9 mg, 0.56 mmol) was obtained in 36% yield as a yellow oil, after purification by flash column chromatography (SiO_2; 0 – 10% EtOAc in cyclohexane). **R$_f$** = 0.54 (4:1 cyclohexane/EtOAc).

^{1}H NMR (300 MHz, $CDCl_3$): δ 7.77 – 7.64 (m, 3H), 7.49 (t, *J* = 8.0 Hz, 1H), 4.88 (s, 2H). *Spectrum*

Spectroscopic data were in agreement with those described in the literature.[24]

2-(Isocyanomethyl)thiophene (1w)

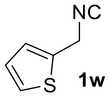

1w

Prepared following the general procedure B, the title compound (924.1 mg, 7.50 mmol) was obtained in 42% yield as a yellow oil, after purification by flash column chromatography (SiO_2; 0 – 15% EtOAc in cyclohexane). **R$_f$** = 0.31 (9:1 DCM/MeOH).

^{1}H NMR (300 MHz, $CDCl_3$): δ 7.32 (dd, *J* = 5.1, 1.2 Hz, 1H), 7.11 – 7.05 (m, 1H), 7.00 (dd, *J* = 5.1, 3.9 Hz, 1H), 4.79 (s, 2H). *Spectrum*

^{13}C NMR (75 MHz, $CDCl_3$): δ 158.0 (t, *J* = 4.8 Hz, -N**C**), 137.4, 127.2, 127.0, 126.4, 40.7 (t, *J* = 7.3 Hz, **C**-NC). *Spectrum*

Spectroscopic data were in agreement with those described in the literature.[25]

***tert*-Butyl 4-isocyano-4-(trifluoromethyl)piperidine-1-carboxylate (1ac)**

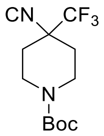

1ac

Prepared following the general procedure B, the title compound (121.0 mg, 1.02 mmol) was obtained in 10% yield as a yellow oil from amine **SI-8**, after purification by flash column chromatography (SiO_2; 0 – 10% EtOAc in cyclohexane). **R$_f$** = 0.30 (9:1 DCM/MeOH).

^{1}H NMR (300 MHz, $CDCl_3$): δ 4.23 (bs, 2H), 3.04 (t, *J* = 12.9 Hz, 2H), 2.05 – 1.92 (m, 2H), 1.80 (dt, *J* = 8.8, 4.1 Hz, 2H), 1.47 (s, 9H). *Spectrum*

^{13}C NMR (75 MHz, $CDCl_3$): δ 163.8, 154.1, 123.2 (q, *J* = 282.6 Hz), 80.6, 62.6 (q, *J* = 30.5 Hz), 38.4 (bs), 29.6, 28.3. *Spectrum*

^{19}F NMR (470 MHz, $CDCl_3$): δ -81.8. *Spectrum*

HRMS (ESI+): calculated for $C_{12}H_{18}F_3N_2O_2$ $[M+H]^+$: 279.1315; found: 279.1317.

m.p.: 58 – 60 ºC.

(3-Isocyano-3-methylbutyl)benzene (1af)

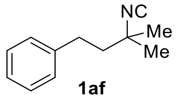

1af

Prepared following the general procedure A, the title compound (168.1 mg, 0.97 mmol) was obtained in 39% yield as a yellow solid from amine **SI-12**, after purification by flash column chromatography (SiO_2; 0 – 3% EtOAc in cyclohexane). $\mathbf{R_f}$ = 0.59 (9:1 cyclohexane/EtOAc).

^{1}H NMR (300 MHz, $CDCl_3$): δ 7.40 – 7.28 (m, 5H), 2.89 – 2.83 (m, 2H), 1.97 – 1.90 (m, 2H), 1.54 (t, J = 1.9 Hz, 6H). Spectrum

Spectroscopic data were in agreement with those described in the literature.[26]

((2-Isocyano-2-methylpropoxy)methyl)benzene (1ah)

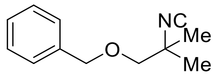

1ah

Prepared following the general procedure A, the title compound (302.2 mg, 1.63 mmol) was obtained in 33% yield as a yellow oil from amine **SI-14**, after purification by flash column chromatography (SiO_2; 0 – 5% EtOAc in cyclohexane). $\mathbf{R_f}$ = 0.44 (9:1 cyclohexane/EtOAc).

^{1}H NMR (300 MHz, $CDCl_3$): δ 7.42 – 7.27 (m, 5H), 4.62 (s, 2H), 3.40 (t, J = 1.7 Hz, 2H), 1.43 (t, J = 2.0 Hz, 6H). Spectrum

^{13}C NMR (75 MHz, $CDCl_3$): δ 154.5 (t, J = 4.5 Hz, -**NC**), 137.8, 128.6, 128.0, 127.7, 76.7, 73.7, 57.4 (t, J = 5.6 Hz, **C**-NC), 26.2. Spectrum

HRMS (ESI+): calculated for $C_{12}H_{16}NO$ $[M+H]^+$: 190.1226; found: 190.1224.

2-((4-Isocyano-4-methylpentyl)oxy)-1,4-dimethylbenzene (1ao)

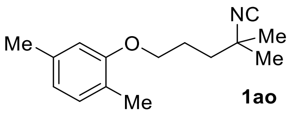

1ao

Prepared following the general procedure A, the title compound (154.7 mg, 0.67 mmol) was obtained in 56% yield as a yellow solid from amine **SI-17**, after purification by flash column chromatography (SiO_2; 0 – 5% EtOAc in cyclohexane). $\mathbf{R_f}$ = 0.59 (9:1 cyclohexane/EtOAc).

^{1}H NMR (300 MHz, $CDCl_3$): δ 7.01 (d, J = 7.4 Hz, 1H), 6.67 (d, J = 7.5 Hz, 1H), 6.62 (s, 1H), 3.99 (t, J = 5.9 Hz, 2H), 2.31 (s, 3H), 2.18 (s, 3H), 2.05 – 1.92 (m, 2H), 1.86 – 1.75 (m, 2H), 1.48 – 1.43 (m, 6H). Spectrum

^{13}**C NMR** (75 MHz, $CDCl_3$): δ 156.8, 153.7 (t, *J* = 4.4 Hz, -N**C**), 136.5, 130.4, 123.5, 120.9, 112.0, 67.2, 57.2 (t, *J* = 5.0 Hz, **C**-NC), 39.2, 29.0, 24.6, 21.4, 15.8. *Spectrum*

HRMS (ESI+): calculated for $C_{15}H_{21}NO$ $[M]^+$: 231.1618; found: 231.1619.

m.p.: 39 – 40 °C.

***tert*-Butyl (S)-2-(isocyanomethyl)pyrrolidine-1-carboxylate (1ar)**

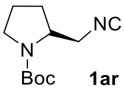

1ar

Prepared following the general procedure B, the title compound (86.9 mg, 0.41 mmol) was obtained in 55% yield as a yellow oil, after purification by flash column chromatography (SiO_2; 0 – 10% EtOAc in cyclohexane). $\mathbf{R_f}$ = 0.30 (4:1 cyclohexane/EtOAc).

^{1}**H NMR** (300 MHz, $CDCl_3$): δ 3.88 (br s, 1H), 3.66 – 3.31 (m, 4H), 2.08 – 1.75 (m, 4H), 1.41 (s, 9H). *Spectrum*

Spectroscopic data were in agreement with those described in the literature.[27]

B. Synthesis of Substituted (Hetero)aryl Nitriles and Pyridylphosphonium Salts

(Hetero)aryl nitriles and pyridylphosphonium salts (***Figure S2***) were prepared according to literature procedures[28-32] (**2f**: ref. 28; **2h**: ref. 29; **2i**: ref. 30; **5a**: ref. 31; **5b**: ref. 32), purchased from suppliers, or synthesized as indicated below.

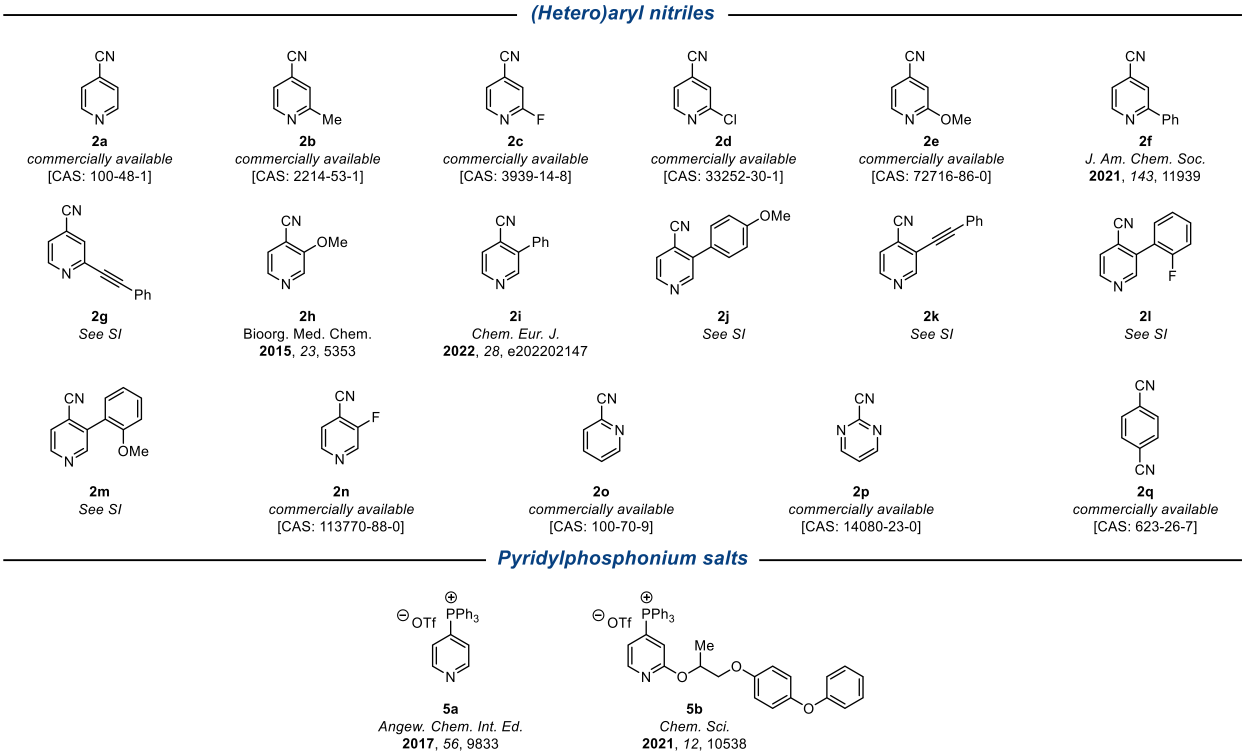

Figure S2: Synthesis of starting (hetero)aryl nitriles and pyridylphosphonium salts.

B. 1. General Procedure D for the Synthesis of Cyano-Pyridines 2j, 2l and 2m

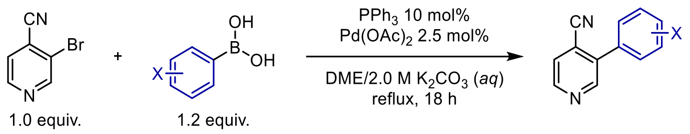

Following a modified procedure of a reported protocol.[33] A flame dried Schlenk flask equipped with a magnetic stir bar, was charged with 3-bromoisonicotinonitrile (1.30 mmol, 1.0 equiv.), boronic acid (1.60 mmol, 1.2 equiv.) and triphenylphosphine (0.13 mmol, 0.10 equiv.). In a vial, K_2CO_3 (4.30 mmol, 3.4 equiv.) was diluted in 2.2 mL water to create a 2.0 M solution, which was sparged with Ar for 30 minutes. The K_2CO_3 (*aq.*) solution was added to the reaction mixture. In a glovebox, $Pd(OAc)_2$ (32.5 µmol, 0.025 equiv.) was dissolved in 2.2 mL 1,2-dimethoxyethane. The $Pd(OAc)_2$ solution was removed from the glovebox and added to the reaction mixture. The reaction was stirred at reflux under argon atmosphere for 18 h. The resulting reaction mixture was

extracted with EtOAc, the combined organic layers were dried over anhydrous Na_2SO_4 and concentrated under reduced pressure. The crude was purified by flash column chromatography on silica gel to afford the corresponding pure product.

3-(4-Methoxyphenyl)isonicotinonitrile (2j)

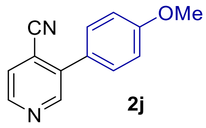

2j

The title compound (193.8 mg, 0.92 mmol) was obtained in 73% yield as a white solid, after purification by flash column chromatography (SiO_2; 0 – 40% EtOAc in cyclohexane). **R_f** = 0.22 (3:1 cyclohexane/EtOAc).

^{1}H NMR (300 MHz, $CDCl_3$): δ 8.84 (br s, 1H), 8.70 (d, *J* = 5.1 Hz, 1H), 7.59 (d, *J* = 5.1 Hz, 1H), 7.54 (d, *J* = 8.0 Hz, 2H), 7.07 (d, *J* = 7.9 Hz, 2H), 3.88 (s, 3H). Spectrum

Spectroscopic data were in agreement with those described in the literature.[34]

3-(2-Fluorophenyl)isonicotinonitrile (2l)

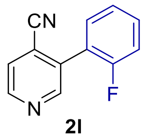

2l

The title compound (130.8 mg, 0.66 mmol) was obtained in 71% yield as a white solid, after purification by flash column chromatography (SiO_2; 0 – 40% EtOAc in cyclohexane). **R_f** = 0.27 (3:1 cyclohexane/EtOAc).

^{1}H NMR (500 MHz, $CDCl_3$): δ 8.84 (br s, 1H), 8.80 (d, *J* = 5.0 Hz, 1H), 7.65 (d, *J* = 5.0 Hz, 1H), 7.51 (dd, *J* = 8.3, 7.2 Hz, 1H), 7.44 (td, *J* = 7.5, 1.8 Hz, 1H), 7.32 (td, *J* = 7.5, 1.6 Hz, 1H), 7.29 – 7.25 (m, 1H). Spectrum

Spectroscopic data were in agreement with those described in the literature.[35]

3-(2-Methoxyphenyl)isonicotinonitrile (2m)

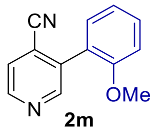

2m

The title compound (168.1 mg, 0.80 mmol) was obtained in 86% yield as a white solid, after purification by flash column chromatography (SiO_2; 0 – 40% EtOAc in cyclohexane). **R_f** = 0.29 (3:1 cyclohexane/EtOAc).

^{1}H NMR (500 MHz, $CDCl_3$): δ 8.78 (br s, 1H), 8.72 (d, *J* = 5.1 Hz, 1H), 7.59 (dd, *J* = 5.0, 0.9 Hz, 1H), 7.47 (ddd, *J* = 8.4, 7.5, 1.7 Hz, 1H), 7.29 (dd, *J* = 7.5, 1.7 Hz, 1H), 7.10 (td, *J* = 7.5, 1.0 Hz, 1H), 7.06 (dd, *J* = 8.4, 1.0 Hz, 1H), 3.85 (s, 3H). Spectrum

^{13}C NMR (75 MHz, $CDCl_3$): δ 156.8, 151.9, 148.6, 136.2, 131.4, 131.0, 125.4, 123.6, 121.2, 116.5, 111.5, 55.5. Spectrum

HRMS (ESI+): calculated for $C_{13}H_{11}N_2O$ $[M+H]^+$: 211.0866; found: 211.0867.

m.p.: 89 – 90 ºC.

B. 2. General Procedure E for the Synthesis of Cyano-Pyridines 2g and 2k

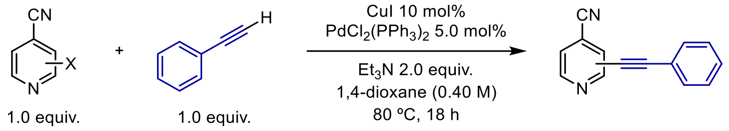

In a flame dried Schlenk flask equipped with a magnetic stir bar, cuprous iodide (0.15 mmol, 10 mol%) was added in the glovebox. Then, the flask was removed from the glovebox and $PdCl_2(PPh_3)_2$ (75.0 µmmol, 5 mol%), halocyanopyridine (1.50 mmol, 1.0 equiv.), 1,4-dioxane (0.40 M), ethynylbenzene (1.50 mmol, 1.0 equiv.) and finally triethylamine (3.00 mmol, 2.0 equiv.) were added in that order. The reaction was stirred at 80 ºC under argon atmosphere for 18 h. Upon completation, the resulting reaction mixture was filtrated though celite, washed with Et_2O and concentrated under reduced pressure. The crude was purified by flash column chromatography on silica gel to afford the corresponding pure product.

2-(Phenylethynyl)isonicotinonitrile (2g)

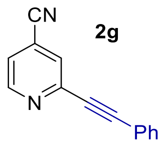

2g

The general procedure was followed with 2-chloroisonicotinonitrile as the halocyanopyridine reactant. The title compound (275.9 mg, 1.35 mmol) was obtained in 90% yield as a brown solid, after purification by flash column chromatography (SiO_2; 0 – 40% EtOAc in cyclohexane). $\mathbf{R_f}$ = 0.18 (4:1 cyclohexane/EtOAc).

^{1}H NMR (500 MHz, $CDCl_3$): δ 8.79 (dd, J = 5.0, 0.9 Hz, 1H), 7.74 (dd, J = 1.5, 0.9 Hz, 1H), 7.63 – 7.59 (m, 2H), 7.46 (dd, J = 5.1, 1.5 Hz, 1H), 7.43 – 7.37 (m, 3H). Spectrum

^{13}C NMR (125 MHz, $CDCl_3$): δ 151.2, 145.0, 132.4, 129.9, 128.7, 128.6, 123.9, 121.4, 121.1, 116.0, 92.4, 87.1. Spectrum

HRMS (ESI+): calculated for $C_{14}H_9N_2$ $[M+H]^+$: 205.0760; found: 205.0757.

m.p.: 88 – 89 ºC.

3-(Phenylethynyl)isonicotinonitrile (2k)

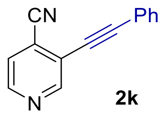

2k

The general procedure was followed with 3-bromoisonicotinonitrile as the halocyanopyridine reactant. The title compound (300.1 mg, 1.47 mmol) was

obtained in 98% yield as a brown solid, after purification by flash column chromatography (SiO_2; 0 – 40% EtOAc in cyclohexane). $\mathbf{R_f}$ = 0.15 (4:1 cyclohexane/EtOAc).

^{1}H NMR (500 MHz, $CDCl_3$): δ 8.94 (br s, 1H), 8.70 (br s, 1H), 7.65 – 7.63 (m, 2H), 7.55 (d, *J* = 4.9 Hz, 1H), 7.45 – 7.39 (m, 3H). *Spectrum*

^{13}C NMR (125 MHz, $CDCl_3$): δ 152.9, 148.6, 132.3, 130.0, 128.7, 125.2, 122.5, 121.5, 115.5, 99.7, 82.7. *Spectrum*

HRMS (ESI+): calculated for $C_{14}H_9N_2$ $[M+H]^+$: 205.0760; found: 205.0755.

m.p.: 64 – 65 ºC

C. Synthesis of 3DPA2FBN (PC)

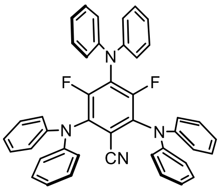

3DPA2FBN

Following a modified procedure of a reported protocol.[36] In a flame dried Schlenk flask equipped with a magnetic stir bar, diphenylamine (3.22 g, 19.0 mmol, 3.8 equiv.) was dissolved in dry THF (80.0 mL) under inert atmosphere. The mixture was cold down to -78 ºC and *n*-BuLi (7.60 mL, 2.5 molar, 19.0 mmol, 3.8 equiv.) was added. The suspension was stirred for 1 hour at -78 ºC. Finally, 2,3,4,5,6-pentafluorobenzonitrile (965 mg, 5.0 mmol, 1.00 equiv.) was added at that temperature and the resulting mixture was stirred overnight allowing it to reach r.t. in the acetone bath. The reaction mixture was quenched by the addition of NH_4Cl (sat.). THF was removed under reduced pressure and DCM was added. The organic phase was washed with water, dried over Na_2SO_4 and the solvent was removed under reduced pressure. Purification via trituration with hot EtOAc, cold down and filtration gave the title compound as a yellow solid (2.55 g, 4.0 mmol, 80% yield) with characterization data in accordance with the literature.[36]

^{1}H NMR (500 MHz, DCM-*d*$_2$): δ 7.29 – 7.23 (m, 12H), 7.08 – 7.02 (m, 6H), 7.00 – 6.96 (m, 12H). Spectrum

D. Synthesis of TMS_3SiNHAd (3)

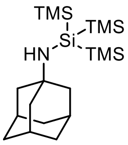

TMS_3SiNHAd

Following a modified procedure of a reported protocol.[37] To a flame dried Schlenk flask containing anhydrous DCM, was added trifluoromethanesulfonic acid (1.39 mL, 15.8 mmol, 1.05 equiv.). Dropwise addition of 1,1,1,3,3,3-hexamethyl-2-(trimethylsilyl)trisilane (4.63 mL, 15.0 mmol, 1.0 equiv.) at 0 ºC was accompanied by evolution of hydrogen gas. The reaction mixture was stirred at r.t. for 1 h, whereupon 1-adamantylamine (2.38 g, 15.8 mmol, 1.05 equiv.) and *N,N*-diisopropylethylamine (3.92 mL, 22.5 mmol, 1.5 equiv.) were added at 0 ºC. The resulting solution was stirred for 12 h at r.t. The solvent was removed under reduced pressure, and the resulting solid was redisolved in hexanes. The mixture was filtered, and the filtrate concentrated in vacuo. Recrystallization by redissolving the crude residue in the minimum amount of hot DCM and adding MeOH afforded the desired product as a white solid (4.95 g, 12.5 mmol, 83% yield) with characterization data in accordance with the literature.[37]

^{1}H NMR (300 MHz, C_6D_6): δ 1.97 (br s, 3H), 1.60 – 1.57 (m, 6H), 1.55 – 1.51 (m, 6H), 0.31 (s, 27H). Spectrum

3. Optimization of the Reaction Conditions

A. General Conditions for Optimization

An oven-dried 4 mL vial equipped with a magnetic stir bar was charged with isonitrile **1a** (0.10 mmol, 1.0 equiv.), isonicotinonitrile **2a**, tris-trimethylsilyl derivative and a photocatalyst. The vial was capped and evacuated three times *via* an inlet needle, purged with argon, and finally it was charged with dry degassed solvent. The reaction mixture was irradiated 24 h with a Kessil PR160L-blue LED lamp (max 45 W High Luminous DEX 2100 LED, λ_{max} = 440 nm). Upon completion, all the volatiles were removed under reduced pressure. Yields were determined by ^{1}H NMR integration versus 1,3,5-trimethoxybenzene as an internal standard.

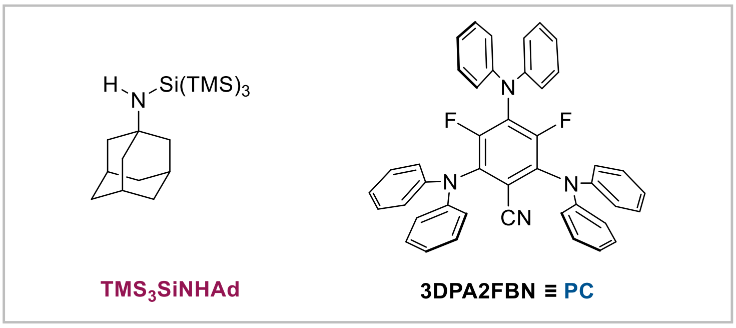

Table S1. Evaluation of silane source

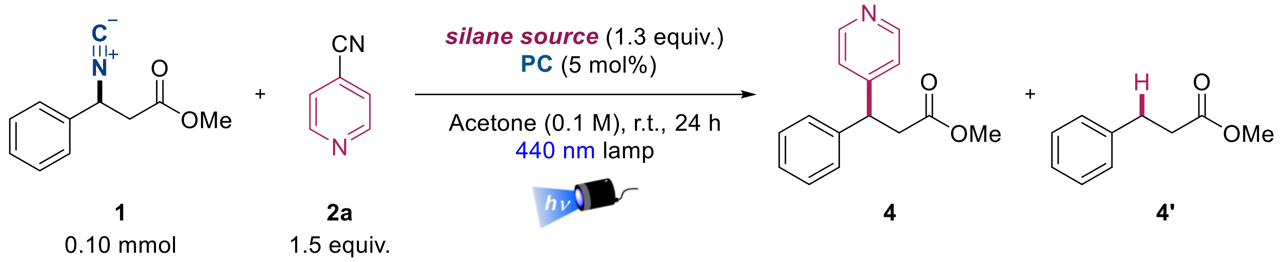

entry	silane source	4 yield (%)	4' yield (%)
1	TMS_3SiH	24	5
2	TMS_3SiOH	47	0
3	$TMS_3SiNHAd$	85	0

Table S2. Evaluation of solvents

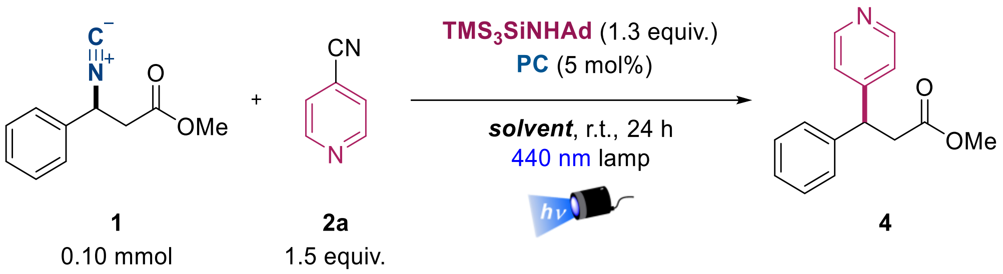

entry	solvent	molarity (M)	yield (%)
1	Acetone	0.1	85
2	Acetone	0.05	88
3	DCE	0.05	22
4	DMSO	0.05	65
5	THF	0.05	29
6	MeOH	0.05	53
7	CH_3CN	0.05	81
8[a]	Acetone	0.1	92 (84)[b]

[a] 0.20 mmol scale. [b] Isolated yield

Table S3. Evaluation of photocatalysts

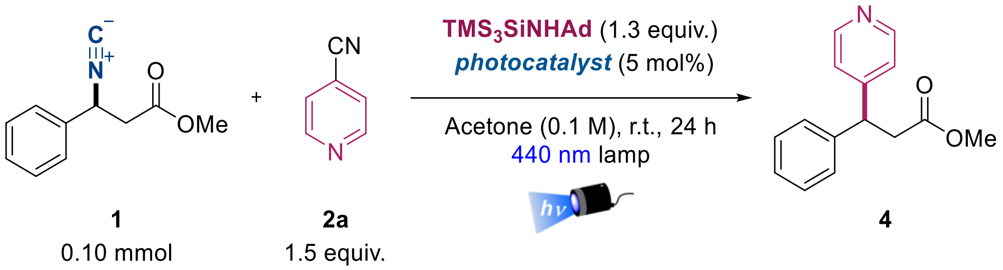

entry	PC	yield (%)
1	3DPA2FBN	85
2	5CzBN	72
3	$Ir(ppy)_3$	28
4	$Ir[(ppy)_2(dtbbpy)]PF_6$	69
5	4CzIPN	62

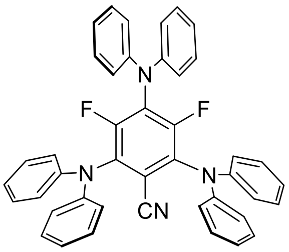

3DPA2FBN ≡ PC

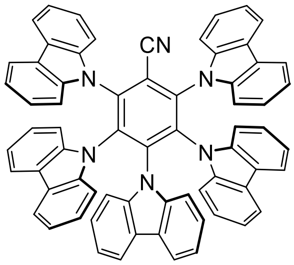

5CzBN

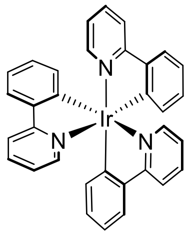

Ir(ppy)$_3$

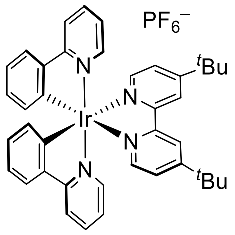

Ir[(ppy)$_2$(dtbbpy)]PF$_6$

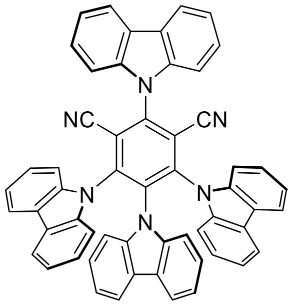

4CzIPN

Table S4. Evaluation of mol% of **PC** and 4-cyanopyridine (**2a**) equivalents

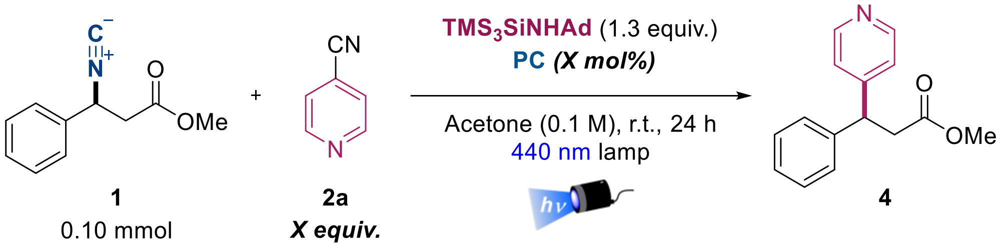

entry	mol% PC1	equiv. of 2a	yield (%)
1	5	1.5	85
2	1	1.5	70
3	5	3.0	78

Table S5. Control experiments

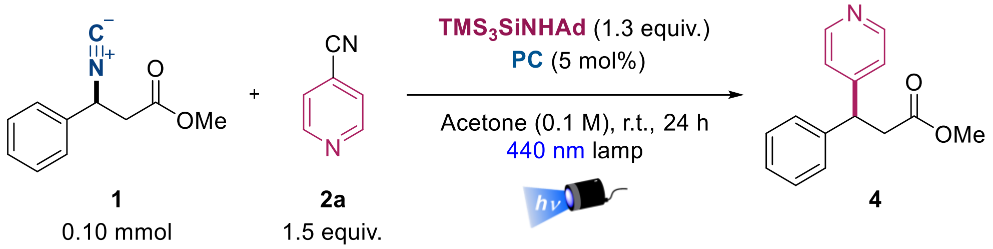

entry	deviation	yield (%)
1	none	85
2	no light	0
3	no **PC**	0
4	no silane	0
5	open air	20

4. Photocatalyzed Deaminative Arylation

A. Experimental Set-up

All photoredox reactions were performed with a Kessil PR160L-blue LED lamp (max 45 W High Luminous DEX 2100 LED, λ_{max} = 440 nm). The lamp was placed 5.0 cm away from the reaction vials, while a fan was used to cool down the system. A typical reaction set-up is shown below.

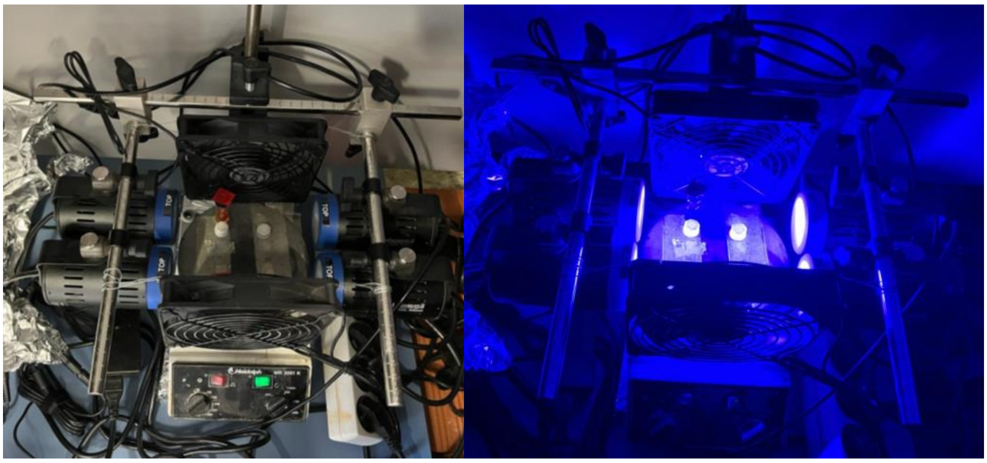

***Figure S3*:** Reaction set-up for the deaminative arylation.

B. General Procedure F

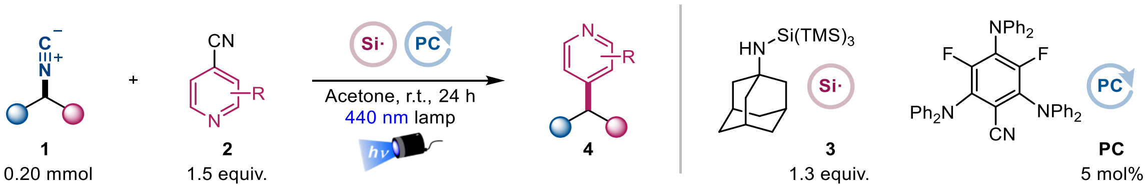

To an oven-dried 4 mL vial equipped with a magnetic stir bar was added isonitrile **1** (0.20 mmol, 1.0 equiv.), cyanopyridine **2** (0.30 mmol, 1.5 equiv.), $TMS_3SiNHAd$ **3** (0.26 mmol, 1.3 equiv.) and 3DPA2FBN **PC** (10.0 µmol, 0.05 equiv.). The vial was capped and evacuated three times *via* an inlet needle then purged with argon, and charged with dry degassed acetone (2.0 mL, 0.1 M), unless otherwise specified. The reaction mixture was irradiated 24 h with a Kessil PR160L-blue LED lamp (max 45 W High Luminous DEX 2100 LED, λ_{max} = 440 nm) as described in the "Experimental set-up" section. Upon completion, all the volatiles were removed under reduced pressure, and the crude mixture was subjected to purification by column chromatography.

Slightly modified conditions (equiv. and reaction time) were used for compounds **4u**, **4v** and **4ao**. For compound **4ap**, we observed better results using acetonitrile as solvent and sodium acetate as an additive. The set up of the reaction was otherwise maintained. See below for details. When using pyridylphosphonium salts, the conditions are the following: isonitrile **1** (0.20 mmol, 1.0 equiv.), pyridylphosphonium salt **5** (0.24 mmol, 1.2 equiv.), $TMS_3SiNHAd$ **3** (0.40 mmol, 2.0 equiv.) and 3DPA2FBN **PC** (10.0 µmol, 0.05 equiv.) in acetone (2.0 mL, 0.1 M) as solvent.

C. Characterization Data

Methyl 3-phenyl-3-(pyridine-4-yl)propanoate (4a)

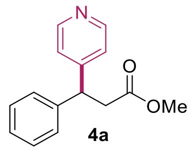

Prepared following the general procedure F using isonitrile **1a** (37.8 mg, 0.20 mmol, 1.0 equiv.), isonicotinonitrile **2a** (31.2 mg, 0.30 mmol, 1.5 equiv.), $(TMS)_3SiNHAdam$ **3** (103.5 mg, 0.26 mmol, 1.3 equiv.) and 3DPA2FBN **PC** (6.4 mg, 10.0 µmol, 5 mol%). The title compound (40.6 mg, 0.17 mmol) was obtained in 84% yield as a brown oil, after purification by flash column chromatography (SiO_2; 0 – 40% EtOAc in cyclohexane). Starting from pyridylphosphonium salt **5a** (117.1 mg, 0.24 mmol, 1.2 equiv.), the title compound (34.9 mg, 0.15 mmol) was obtained in 72% yield. $\mathbf{R_f}$ = 0.43 (1:1 cyclohexane/EtOAc).

^{1}H NMR (500 MHz, $CDCl_3$): δ 8.49 (d, J = 6.2 Hz, 2H), 7.34 – 7.27 (m, 2H), 7.25 – 7.17 (m, 3H), 7.17 – 7.12 (m, 2H), 4.53 (t, J = 7.9 Hz, 1H), 3.59 (s, 3H), 3.06 (dd, J = 8.0, 2.0 Hz, 2H). Spectrum

Spectroscopic data were in agreement with those described in the literature.[38]

4-(1-(4-(Benzyloxy)phenyl)ethyl)pyridine (4b)

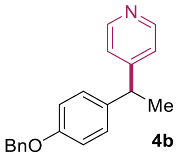

Prepared following the general procedure F using isonitrile **1b** (47.5 mg, 0.20 mmol, 1.0 equiv.), isonicotinonitrile **2a** (31.2 mg, 0.30 mmol, 1.5 equiv.), $(TMS)_3SiNHAdam$ **3** (103.5 mg, 0.26 mmol, 1.3 equiv.) and 3DPA2FBN **PC** (6.4 mg, 10.0 µmol, 5 mol%). The title compound (43.2 mg, 0.15 mmol) was obtained in 75% yield as a yellow solid, after purification by flash column chromatography (SiO_2; 0 – 40% EtOAc in cyclohexane). $\mathbf{R_f}$ = 0.17 (2:1 cyclohexane/EtOAc).

^{1}H NMR (300 MHz, $CDCl_3$): δ 8.49 (d, J = 6.0 Hz, 2H), 7.46 – 7.29 (m, 5H), 7.15 – 7.08 (m, 4H), 6.93 (d, J = 8.6 Hz, 2H), 5.04 (s, 2H), 4.07 (q, J = 7.2 Hz, 1H), 1.61 (d, J = 7.3 Hz, 3H). Spectrum

^{13}C NMR (75 MHz, $CDCl_3$): δ 157.5, 155.5, 149.8, 137.0, 136.8, 128.6, 128.0, 127.5, 122.9, 114.9, 70.1, 43.5, 21.3. Spectrum

HRMS (ESI+): calculated for $C_{20}H_{20}NO$ $[M+H]^+$: 290.1539; found: 290.1536.

m.p.: 74 – 75 °C.

4-(1-(4-Methoxyphenyl)ethyl)pyridine (4c)

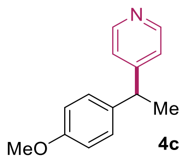

Prepared following the general procedure F using isonitrile **1c** (32.2 mg, 0.20 mmol, 1.0 equiv.), isonicotinonitrile **2a** (31.2 mg, 0.30 mmol, 1.5 equiv.), $(TMS)_3$SiNHAdam **3** (103.5 mg, 0.26 mmol, 1.3 equiv.) and 3DPA2FBN **PC** (6.4 mg, 10.0 µmol, 5 mol%). The title compound (34.2 mg, 0.16 mmol) was obtained in 80% yield as a colorless oil, after purification by flash column chromatography (SiO_2; 0 – 40% EtOAc in cyclohexane). $\mathbf{R_f}$ = 0.21 (2:1 cyclohexane/EtOAc).

^{1}H NMR (300 MHz, $CDCl_3$): δ 8.48 (d, J = 4.2 Hz, 2H), 7.15 – 7.04 (m, 4H), 6.87 – 6.80 (m, 2H), 4.07 (q, J = 7.3 Hz, 1H), 3.79 (s, 3H), 1.61 (d, J = 7.2 Hz, 3H). *Spectrum*

Spectroscopic data were in agreement with those described in the literature.[39]

4-(1-(3-Methoxyphenyl)ethyl)pyridine (4d)

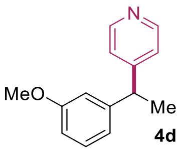

Prepared following the general procedure F using isonitrile **1d** (32.2 mg, 0.20 mmol, 1.0 equiv.), isonicotinonitrile **2a** (31.2 mg, 0.30 mmol, 1.5 equiv.), $(TMS)_3$SiNHAdam **3** (103.5 mg, 0.26 mmol, 1.3 equiv.) and 3DPA2FBN **PC** (6.4 mg, 10.0 µmol, 5 mol%). The title compound (40.3 mg, 0.19 mmol) was obtained in 94% yield as a yellowish oil, after purification by flash column chromatography (SiO_2; 0 – 40% EtOAc in cyclohexane). $\mathbf{R_f}$ = 0.19 (2:1 cyclohexane/EtOAc).

^{1}H NMR (300 MHz, $CDCl_3$): δ 8.49 (d, J = 6.2 Hz, 2H), 7.23 (t, J = 7.8 Hz, 1H), 7.13 (d, J = 6.1 Hz, 2H), 6.82 – 6.71 (m, 3H), 4.08 (q, J = 7.2 Hz, 1H), 3.77 (s, 3H), 1.62 (d, J = 7.2 Hz, 3H). *Spectrum*

Spectroscopic data were in agreement with those described in the literature.[39]

4-(Cyclopropyl(4-methoxyphenyl)methyl)pyridine (4e)

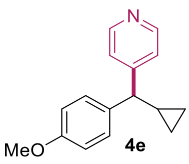

Prepared following the general procedure F using isonitrile **1e** (37.4 mg, 0.20 mmol, 1.0 equiv.), isonicotinonitrile **2a** (31.2 mg, 0.30 mmol, 1.5 equiv.), $(TMS)_3$SiNHAdam **3** (103.5 mg, 0.26 mmol, 1.3 equiv.) and 3DPA2FBN **PC** (6.4 mg, 10.0 µmol, 5 mol%). The title compound (34.1 mg, 0.14 mmol) was obtained in 71% yield as a yellowish oil, after purification by flash column chromatography (SiO_2; 0 – 40% EtOAc in cyclohexane). $\mathbf{R_f}$ = 0.36 (1:1 cyclohexane/EtOAc).

^{1}H NMR (300 MHz, $CDCl_3$): δ 8.49 (d, *J* = 5.6 Hz, 2H), 7.18 (d, *J* = 6.1 Hz, 2H), 7.13 (d, *J* = 8.6 Hz, 2H), 6.84 (d, *J* = 8.6 Hz, 2H), 3.79 (s, 3H), 3.13 (d, *J* = 9.6 Hz, 1H), 1.38 – 1.27 (m, 1H), 0.72 – 0.64 (m, 2H), 0.33 – 0.25 (m, 2H). Spectrum

^{13}C NMR (75 MHz, $CDCl_3$): δ 158.4, 154.4, 149.8, 135.6, 129.3, 123.6, 114.0, 55.3, 54.4, 16.4, 5.5, 5.3. Spectrum

HRMS (APCI+): calculated for $C_{16}H_{18}NO$ $[M+H]^+$: 240.1386; found: 240.1378.

4-(1,2,3,4-Tetrahydronaphthalen-1-yl)pyridine (4f)

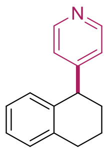

4f

Prepared following the general procedure F using isonitrile **1f** (31.4 mg, 0.20 mmol, 1.0 equiv.), isonicotinonitrile **2a** (31.2 mg, 0.30 mmol, 1.5 equiv.), $(TMS)_3SiNHAdam$ **3** (103.5 mg, 0.26 mmol, 1.3 equiv.) and 3DPA2FBN **PC** (6.4 mg, 10.0 µmol, 5 mol%). The title compound (36.5 mg, 0.17 mmol) was obtained in 87% yield as a yellow oil, after purification by flash column chromatography (SiO_2; 0 – 40% EtOAc in cyclohexane). $\mathbf{R_f}$ = 0.15 (2:1 cyclohexane/EtOAc).

^{1}H NMR (300 MHz, $CDCl_3$): δ 8.49 (d, *J* = 6.0 Hz, 2H), 7.16 (d, *J* = 4.3 Hz, 2H), 7.09 – 7.03 (m, 1H), 7.02 (d, *J* = 6.1 Hz, 2H), 6.79 (d, *J* = 7.6 Hz, 1H), 4.12 (t, *J* = 6.3 Hz, 1H), 3.09 – 2.63 (m, 2H), 2.25 – 2.09 (m, 1H), 1.92 – 1.69 (m, 3H). Spectrum

Spectroscopic data were in agreement with those described in the literature.[40]

4-(2-Methyl-1-phenylpropyl)pyridine (4g)

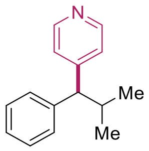

4g

Prepared following the general procedure F using isonitrile **1g** (31.8 mg, 0.20 mmol, 1.0 equiv.), isonicotinonitrile **2a** (31.2 mg, 0.30 mmol, 1.5 equiv.), $(TMS)_3SiNHAdam$ **3** (103.5 mg, 0.26 mmol, 1.3 equiv.) and 3DPA2FBN **PC** (6.4 mg, 10.0 µmol, 5 mol%). The title compound (32.0 mg, 0.15 mmol) was obtained in 75% yield as a yellowish oil, after purification by flash column chromatography (SiO_2; 0 – 40% EtOAc in cyclohexane). $\mathbf{R_f}$ = 0.41 (1:1 cyclohexane/EtOAc).

^{1}H NMR (300 MHz, $CDCl_3$): δ 8.47 (d, *J* = 6.1 Hz, 2H), 7.32 – 7.14 (m, 7H), 3.39 (d, *J* = 10.8 Hz, 1H), 2.57 – 2.40 (m, 1H), 0.88 (d, *J* = 6.5 Hz, 6H). Spectrum

Spectroscopic data were in agreement with those described in the literature.[39]

4-(2,2-Dimethyl-1-phenylpropyl)pyridine (4h)

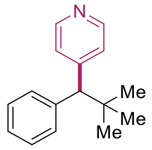

4h

Prepared following the general procedure F using isonitrile **1h** (34.7 mg, 0.20 mmol, 1.0 equiv.), isonicotinonitrile **2a** (31.2 mg, 0.30 mmol, 1.5 equiv.), $(TMS)_3SiNHAdam$ **3** (103.5 mg, 0.26 mmol, 1.3 equiv.) and 3DPA2FBN **PC** (6.4 mg, 10.0 µmol, 5 mol%). The title compound (32.0 mg, 0.14 mmol) was obtained in 71% yield as a colorless oil, after purification by flash column chromatography (SiO_2; 0 – 40% EtOAc in cyclohexane). $\mathbf{R_f}$ = 0.41 (1:1 cyclohexane/EtOAc).

^{1}H NMR (300 MHz, $CDCl_3$): δ 8.53 (d, J = 5.1 Hz, 2H), 7.46 – 7.25 (m, 7H), 3.71 (s, 1H), 1.07 (s, 9H). Spectrum

^{13}C NMR (75 MHz, $CDCl_3$): δ 152.0, 149.6, 141.5, 129.9, 128.3, 126.8, 125.3, 64.0, 35.2, 29.2. Spectrum

HRMS (ESI+): calculated for $C_{16}H_{20}N$ $[M+H]^+$: 226.1590; found: 226.1588.

4-(1-Phenyl-2-(4,4,5,5-tetramethyl-1,3,2-dioxaborolan-2-yl)ethyl)pyridine (4i)

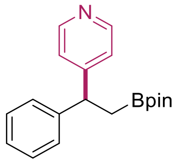

4i

Prepared following the general procedure F using isonitrile **1i** (51.4 mg, 0.20 mmol, 1.0 equiv.), isonicotinonitrile **2a** (31.2 mg, 0.30 mmol, 1.5 equiv.), $(TMS)_3SiNHAdam$ **3** (103.5 mg, 0.26 mmol, 1.3 equiv.) and 3DPA2FBN **PC** (6.4 mg, 10.0 µmol, 5 mol%). The title compound (35.8 mg, 0.12 mmol) was obtained in 58% yield as a colorless oil, after purification by flash column chromatography (SiO_2; 0 – 30% EtOAc in cyclohexane). $\mathbf{R_f}$ = 0.33 (3:1 cyclohexane/EtOAc).

^{1}H NMR (300 MHz, $CDCl_3$): δ 8.46 (d, J = 6.2 Hz, 2H), 7.29 – 7.26 (m, 2H), 7.24 – 7.17 (m, 5H), 4.25 (t, J = 8.4 Hz, 1H), 1.58 (d, J = 8.4 Hz, 2H), 1.07 (s, 12H). Spectrum

Spectroscopic data were in agreement with those described in the literature.[41]

4-(2-Phenylpropan-2-yl)pyridine (4j)

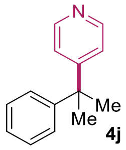

4j

Prepared following the general procedure F using isonitrile **1j** (29.0 mg, 0.20 mmol, 1.0 equiv.), isonicotinonitrile **2a** (31.2 mg, 0.30 mmol, 1.5 equiv.), $(TMS)_3SiNHAdam$ **3** (103.5 mg, 0.26 mmol, 1.3 equiv.) and 3DPA2FBN **PC** (6.4 mg, 10.0 µmol, 5 mol%). The title compound (29.4 mg, 0.15 mmol) was obtained in 75% yield as a colorless oil, after purification by flash column chromatography (SiO_2; 0 – 40% EtOAc in cyclohexane). $\mathbf{R_f}$ = 0.19 (2:1 cyclohexane/EtOAc).

^{1}**H NMR** (300 MHz, $CDCl_3$): δ 8.48 (d, *J* = 6.3 Hz, 2H), 7.35 – 7.26 (m, 2H), 7.24 – 7.17 (m, 3H), 7.13 (d, *J* = 6.2 Hz, 2H), 1.68 (s, 6H). *Spectrum*

Spectroscopic data were in agreement with those described in the literature.[39]

4-Benzhydrylpyridine (4k)

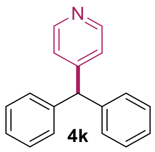

4k

Prepared following the general procedure F using isonitrile **1k** (38.7 mg, 0.20 mmol, 1.0 equiv.), isonicotinonitrile **2a** (31.2 mg, 0.30 mmol, 1.5 equiv.), $(TMS)_3SiNHAdam$ **3** (103.5 mg, 0.26 mmol, 1.3 equiv.) and 3DPA2FBN **PC** (6.4 mg, 10.0 µmol, 5 mol%). The title compound (29.7 mg, 0.12 mmol) was obtained in 61% yield as a yellowish oil, after purification by flash column chromatography (SiO_2; 0 – 40% EtOAc in cyclohexane). $\mathbf{R_f}$ = 0.31 (2:1 cyclohexane/EtOAc).

^{1}**H NMR** (300 MHz, $CDCl_3$): δ 8.55 (d, *J* = 5.1 Hz, 2H), 7.42 – 7.26 (m, 6H), 7.20 – 7.11 (m, 4H), 7.08 (d, *J* = 5.1 Hz, 2H), 5.54 (s, 1H). *Spectrum*

Spectroscopic data were in agreement with those described in the literature.[40]

4-(4-Methoxybenzyl)pyridine (4l)

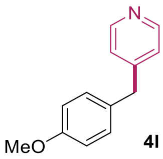

4l

Prepared following the general procedure F using isonitrile **1l** (29.4 mg, 0.20 mmol, 1.0 equiv.), isonicotinonitrile **2a** (31.2 mg, 0.30 mmol, 1.5 equiv.), $(TMS)_3SiNHAdam$ **3** (103.5 mg, 0.26 mmol, 1.3 equiv.) and 3DPA2FBN **PC** (6.4 mg, 10.0 µmol, 5 mol%). The title compound (30.3 mg, 0.15 mmol) was obtained in 76% yield as an orange oil, after purification by flash column chromatography (SiO_2; 0 – 40% EtOAc in cyclohexane). $\mathbf{R_f}$ = 0.15 (1:1 cyclohexane/EtOAc).

^{1}**H NMR** (300 MHz, $CDCl_3$): δ 8.47 (d, *J* = 5.7 Hz, 2H), 7.15 – 7.01 (m, 4H), 6.84 (d, *J* = 8.5 Hz, 2H), 3.89 (s, 2H), 3.78 (s, 3H). *Spectrum*

Spectroscopic data were in agreement with those described in the literature.[42]

4-(4-(Pyridin-4-ylmethyl)phenyl)morpholine (4m)

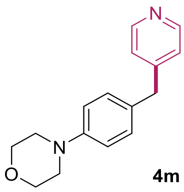

4m

Prepared following the general procedure F using isonitrile **1m** (40.5 mg, 0.20 mmol, 1.0 equiv.), isonicotinonitrile **2a** (31.2 mg, 0.30 mmol, 1.5 equiv.), $(TMS)_3SiNHAdam$ **3** (103.5 mg, 0.26 mmol, 1.3 equiv.) and 3DPA2FBN **PC** (6.4 mg, 10.0 µmol, 5 mol%). The title compound (31.9 mg, 0.13 mmol) was

obtained in 63% yield as an orange oil, after purification by flash column chromatography (SiO_2; 0 – 40% EtOAc in cyclohexane). **R$_f$** = 0.18 (1:1 cyclohexane/EtOAc).

^{1}H NMR (300 MHz, $CDCl_3$): δ 8.47 (d, *J* = 6.0 Hz, 2H), 7.11 – 7.05 (m, 4H), 6.86 (d, *J* = 8.6 Hz, 2H), 3.89 (s, 2H), 3.87 – 3.83 (m, 4H), 3.16 – 3.11 (m, 4H). Spectrum

^{13}C NMR (75 MHz, $CDCl_3$): δ 150.7, 150.1, 149.8, 130.4, 129.9, 124.2, 116.1, 67.0, 49.5, 40.5. Spectrum

HRMS (ESI+): calculated for $C_{16}H_{19}N_2O$ $[M+H]^+$: 255.1492; found: 255.1490.

4-([1,1'-Biphenyl]-4-ylmethyl)pyridine (4n)

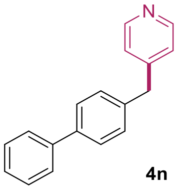
4n

Prepared following the general procedure F using isonitrile **1n** (38.7 mg, 0.20 mmol, 1.0 equiv.), isonicotinonitrile **2a** (31.2 mg, 0.30 mmol, 1.5 equiv.), $(TMS)_3$SiNHAdam **3** (103.5 mg, 0.26 mmol, 1.3 equiv.) and 3DPA2FBN **PC** (6.4 mg, 10.0 µmol, 5 mol%). The title compound (20.3 mg, 0.08 mmol) was obtained in 41% yield as a white solid, after purification by flash column chromatography (SiO_2; 0 – 30% EtOAc in cyclohexane). **R$_f$** = 0.13 (2:1 cyclohexane/EtOAc).

^{1}H NMR (300 MHz, $CDCl_3$): δ 8.52 (d, *J* = 4.6 Hz, 2H), 7.57 (m, 4H), 7.46 – 7.41 (m, 2H), 7.38 – 7.31 (m, 1H), 7.25 (d, *J* = 7.7 Hz, 2H), 7.14 (d, *J* = 5.5 Hz, 2H), 4.01 (s, 2H). Spectrum

Spectroscopic data were in agreement with those described in the literature.[43]

4-[[4-(4,4,5,5-Tetramethyl-1,3,2-dioxaborolan-2-yl)phenyl]methyl]pyridine (4o)

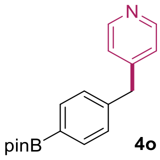
4o

Prepared following the general procedure F using isonitrile **1o** (48.6 mg, 0.20 mmol, 1.0 equiv.), isonicotinonitrile **2a** (31.2 mg, 0.30 mmol, 1.5 equiv.), $(TMS)_3$SiNHAdam **3** (103.5 mg, 0.26 mmol, 1.3 equiv.) and 3DPA2FBN **PC** (6.4 mg, 10.0 µmol, 5 mol%). The title compound (28.4 mg, 0.10 mmol) was obtained in 48% yield as a yellow liquid, after purification by flash column chromatography (SiO_2; 10 – 60% EtOAc in cyclohexane). **R$_f$** = 0.39 (1:1 cyclohexane/EtOAc).

^{1}H NMR (300 MHz, $CDCl_3$): δ 8.49 (br s, 2H), 7.76 (d, *J* = 8.0 Hz, 2H), 7.18 (d, *J* = 8.1 Hz, 2H), 7.11 (d, *J* = 5.5 Hz, 2H), 3.99 (s, 2H), 1.34 (s, 12H). Spectrum

^{13}C NMR (75 MHz, $CDCl_3$): δ 150.1, 149.8, 142.1, 135.4, 128.6, 124.5, 84.0, 41.6, 25.0. Spectrum

^{11}B NMR (160 MHz, $CDCl_3$): δ 30.6. Spectrum

HRMS (ESI+): calculated for $C_{18}H_{23}BNO_2$ $[M+H]^+$: 296.1816; found: 296.1814.

Methyl 4-(pyridine-4-ylmethyl)benzoate (4p)

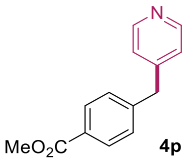

4p

Prepared following the general procedure F using isonitrile **1p** (35.0 mg, 0.20 mmol, 1.0 equiv.), isonicotinonitrile **2a** (31.2 mg, 0.30 mmol, 1.5 equiv.), $(TMS)_3SiNHAdam$ **3** (103.5 mg, 0.26 mmol, 1.3 equiv.) and 3DPA2FBN **PC** (6.4 mg, 10.0 µmol, 5 mol%). The title compound (24.4 mg, 0.11 mmol) was obtained in 54% yield as a yellow oil, after purification by flash column chromatography (SiO_2; 0 – 45% EtOAc in cyclohexane). $\mathbf{R_f}$ = 0.30 (1:1 cyclohexane/EtOAc).

^{1}H NMR (300 MHz, $CDCl_3$): δ 8.52 (br s, 2H), 7.99 (d, J = 8.0 Hz, 2H), 7.25 (d, J = 8.0 Hz, 2H), 7.09 (d, J = 5.0 Hz, 2H), 4.02 (s, 2H), 3.91 (s, 3H). Spectrum

^{13}C NMR (75 MHz, $CDCl_3$): δ 167.0, 150.2, 149.1, 144.3, 130.2, 129.2, 128.9, 124.3, 52.2, 41.3. Spectrum

HRMS (ESI+): calculated for $C_{14}H_{14}NO_2$ $[M+H]^+$: 228.1019; found: 228.1022.

4-(4-(Trifluoromethyl)benzyl)pyridine (4q)

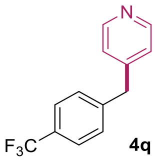

4q

Prepared following the general procedure F using isonitrile **1q** (37.0 mg, 0.20 mmol, 1.0 equiv.), isonicotinonitrile **2a** (31.2 mg, 0.30 mmol, 1.5 equiv.), $(TMS)_3SiNHAdam$ **3** (103.5 mg, 0.26 mmol, 1.3 equiv.) and 3DPA2FBN **PC** (6.4 mg, 10.0 µmol, 5 mol%). The title compound (23.7 mg, 0.10 mmol) was obtained in 50% yield as a brown oil, after purification by flash column chromatography (SiO_2; 0 – 40% EtOAc in cyclohexane). $\mathbf{R_f}$ = 0.41 (1:1 cyclohexane/EtOAc).

^{1}H NMR (300 MHz, $CDCl_3$): δ 8.53 (d, J = 5.8 Hz, 2H), 7.58 (d, J = 8.0 Hz, 2H), 7.29 (d, J = 8.0 Hz, 2H), 7.12 (d, J = 5.9 Hz, 2H), 4.04 (s, 2H). Spectrum

Spectroscopic data were in agreement with those described in the literature.[43]

4-(Naphthalen-2-ylmethyl)pyridine (4r)

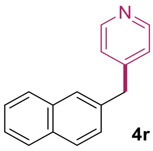

4r

Prepared following the general procedure F using isonitrile **1r** (33.4 mg, 0.20 mmol, 1.0 equiv.), isonicotinonitrile **2a** (31.2 mg, 0.30 mmol, 1.5 equiv.), $(TMS)_3SiNHAdam$ **3** (103.5 mg, 0.26 mmol, 1.3 equiv.) and 3DPA2FBN **PC** (6.4 mg, 10.0 µmol, 5 mol%). The title compound (40.1 mg, 0.18 mmol) was obtained

in 91% yield as a yellowish oil, after purification by flash column chromatography (SiO_2; 0 – 40% Et_2O in pentane). $\mathbf{R_f}$ = 0.33 (1:1 pentane/Et_2O).

^{1}H NMR (300 MHz, $CDCl_3$): δ 8.53 (br s, 2H), 7.84 – 7.76 (m, 3H), 7.64 (s, 1H), 7.51 – 7.43 (m, 2H), 7.30 – 7.26 (m, 1H), 7.17 (d, *J* = 5.0 Hz, 2H), 4.14 (s, 2H). Spectrum

Spectroscopic data were in agreement with those described in the literature.[44]

4-(2-Methylbenzyl)pyridine (4s)

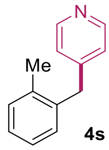

4s

Prepared following the general procedure F using isonitrile **1s** (26.2 mg, 0.20 mmol, 1.0 equiv.), isonicotinonitrile **2a** (31.2 mg, 0.30 mmol, 1.5 equiv.), $(TMS)_3SiNHAdam$ **3** (103.5 mg, 0.26 mmol, 1.3 equiv.) and 3DPA2FBN **PC** (6.4 mg, 10.0 µmol, 5 mol%). The title compound (30.2 mg, 0.17 mmol) was obtained in 82% yield as a brown oil, after purification by flash column chromatography (SiO_2; 0 – 30% EtOAc in cyclohexane). $\mathbf{R_f}$ = 0.15 (4:1 cyclohexane/EtOAc).

^{1}H NMR (300 MHz, $CDCl_3$): δ 8.47 (dd, *J* = 6.1, 1.5 Hz 2H), 7.21 – 7.16 (m, 3H), 7.13 – 7.09 (m, 1H), 7.03 (dd, *J* = 6.1, 1.5 Hz, 2H), 3.98 (s, 2H), 2.21 (s, 3H). Spectrum

Spectroscopic data were in agreement with those described in the literature.[43]

4-(2-(Trifluoromethyl)benzyl)pyridine (4t)

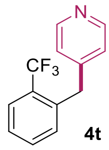

4t

Prepared following the general procedure F using isonitrile **1t** (37.0 mg, 0.20 mmol, 1.0 equiv.), isonicotinonitrile **2a** (31.2 mg, 0.30 mmol, 1.5 equiv.), $(TMS)_3SiNHAdam$ **3** (103.5 mg, 0.26 mmol, 1.3 equiv.) and 3DPA2FBN **PC** (6.4 mg, 10.0 µmol, 5 mol%). The title compound (31.5 mg, 0.13 mmol) was obtained in 66% yield as a brown oil, after purification by flash column chromatography (SiO_2; 0 – 40% EtOAc in cyclohexane). $\mathbf{R_f}$ = 0.29 (1:1 cyclohexane/EtOAc).

^{1}H NMR (300 MHz, $CDCl_3$): δ 8.51 (br s, 2H), 7.70 (d, *J* = 8.3 Hz, 1H), 7.48 (t, *J* = 7.2 Hz, 1H), 7.37 (t, *J* = 7.2 Hz, 1H), 7.19 (d, *J* = 7.7 Hz, 1H), 7.05 (d, *J* = 4.8 Hz, 2H), 4.17 (s, 2H). Spectrum

^{13}C NMR (75 MHz, $CDCl_3$): δ 149.8, 149.3, 137.1, 132.2, 132.1, 129.2 (q, *J* = 30.0 Hz), 127.1, 126.4 (q, *J* = 5.9 Hz), 124.4 (br s), 124.5 (q, *J* = 274.3 Hz), 37.5. Spectrum

^{19}F NMR (470 MHz, $CDCl_3$): δ -59.6. Spectrum

HRMS (ESI+): calculated for $C_{13}H_{11}F_3N$ $[M+H]^+$: 238.0838; found: 238.0839.

tert-Butyl 3-(pyridin-4-ylmethyl)-1H-indole-1-carboxylate (4u)

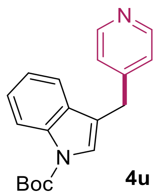

Prepared following the general procedure F using isonitrile **1v** (51.3 mg, 0.20 mmol, 1.0 equiv.), isonicotinonitrile **2a** (31.2 mg, 0.30 mmol, 1.5 equiv.), $(TMS)_3SiNHAdam$ **3** (103.5 mg, 0.26 mmol, 1.3 equiv.) and 3DPA2FBN **PC** (6.4 mg, 10.0 µmol, 5 mol%). The title compound (46.3 mg, 0.15 mmol) was obtained in 75% yield as a brown oil, after purification by flash column chromatography (SiO_2; 0 – 40% EtOAc in cyclohexane). $\mathbf{R_f}$ = 0.31 (1:1 cyclohexane/EtOAc).

^{1}H NMR (300 MHz, $CDCl_3$): δ 8.53 (br s, 2H), 8.13 (d, J = 8.1 Hz, 1H), 7.40 (s, 1H), 7.37 – 7.28 (m, 2H), 7.24 – 7.15 (m, 3H), 4.05 (s, 2H), 1.67 (s, 9H). Spectrum

Spectroscopic data were in agreement with those described in the literature.[45]

4-(Thiophen-2-ylmethyl)pyridine (4v)

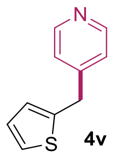

Prepared following the general procedure F using isonitrile **1w** (24.6 mg, 0.20 mmol, 1.0 equiv.), isonicotinonitrile **2a** (31.2 mg, 0.30 mmol, 1.5 equiv.), $(TMS)_3SiNHAdam$ **3** (103.5 mg, 0.26 mmol, 1.3 equiv.) and 3DPA2FBN **PC** (6.4 mg, 10.0 µmol, 5 mol%). The title compound (22.7 mg, 0.13 mmol) was obtained in 65% yield as a yellow oil, after purification by flash column chromatography (SiO_2; 0 – 40% EtOAc in cyclohexane). $\mathbf{R_f}$ = 0.62 (1:1 cyclohexane/EtOAc).

^{1}H NMR (300 MHz, $CDCl_3$): δ 8.53 (d, J = 4.8 Hz, 2H), 7.21 – 7.14 (m, 3H), 6.95 (dd, J = 5.2, 3.4 Hz, 1H), 6.85 – 6.81 (m, 1H), 4.15 (s, 2H). Spectrum

^{13}C NMR (75 MHz, $CDCl_3$): δ 150.0, 149.4, 141.1, 127.2, 126.2, 124.8, 123.9, 35.4. Spectrum

HRMS (ESI+): calculated for $C_{10}H_{10}NS$ $[M+H]^+$: 176.0528; found: 176.0526.

Di(pyridine-4-yl)methane (4w)

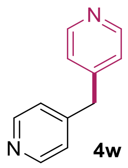

Prepared following the general procedure F using isonitrile **1x** (23.6 mg, 0.20 mmol, 1.0 equiv.), isonicotinonitrile **2a** (31.2 mg, 0.30 mmol, 1.5 equiv.), $(TMS)_3SiNHAdam$ **3** (103.5 mg, 0.26 mmol, 1.3 equiv.) and 3DPA2FBN **PC** (6.4 mg, 10.0 µmol, 5 mol%). The title compound (15.3 mg, 0.09 mmol) was obtained in 45% yield as a yellow oil, after purification by flash column chromatography (SiO_2; 0 – 15% *i*PrOH in CH_2Cl_2). $\mathbf{R_f}$ = 0.58 (9:1 DCM/MeOH).

^{1}H NMR (300 MHz, $CDCl_3$): δ 8.56 – 8.54 (m, 4H), 7.12 – 7.10 (m, 4H), 3.97 (s, 2H). Spectrum

Spectroscopic data were in agreement with those described in the literature.[46]

4-(1-(3-Methoxyphenyl)ethyl)-2-methylpyridine (4x)

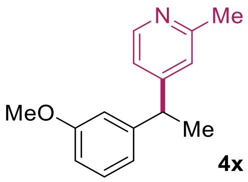

4x

Prepared following the general procedure F using isonitrile **1d** (32.2 mg, 0.20 mmol, 1.0 equiv.), 2-methylisonicotinonitrile **2b** (35.4 mg, 0.30 mmol, 1.5 equiv.), $(TMS)_3SiNHAdam$ **3** (103.5 mg, 0.26 mmol, 1.3 equiv.) and 3DPA2FBN **PC** (6.4 mg, 10.0 µmol, 5 mol%). The title compound (31.9 mg, 0.14 mmol) was obtained in 70% yield as an orange oil, after purification by flash column chromatography (SiO_2; 0 – 50% Et_2O in pentane). $\mathbf{R_f}$ = 0.24 (2:1 pentane/Et_2O).

^{1}H NMR (300 MHz, $CDCl_3$): δ 8.37 (br s, 1H), 7.24 – 7.19 (m, 1H), 6.99 – 6.94 (m, 2H), 6.80 – 6.73 (m, 3H), 4.03 (q, J = 7.2 Hz, 1H), 3.77 (s, 3H), 2.50 (s, 3H), 1.60 (d, J = 7.2 Hz, 3H). Spectrum

^{13}C NMR (75 MHz, $CDCl_3$): δ 159.9, 158.4, 155.4, 149.1, 146.3, 129.7, 122.6, 120.1, 114.0, 111.5, 55.3, 44.3, 24.5, 21.1. Spectrum

HRMS (ESI+): calculated for $C_{15}H_{18}NO$ $[M+H]^+$: 228.1383; found: 228.1380.

2-Fluoro-4-(1-(3-methoxyphenyl)ethyl)pyridine (4y)

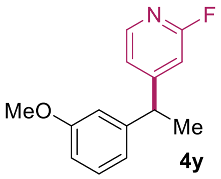

4y

Prepared following the general procedure F using isonitrile **1d** (32.2 mg, 0.20 mmol, 1.0 equiv.), 2-fluoroisonicotinonitrile **2c** (36.6 mg, 0.30 mmol, 1.5 equiv.), $(TMS)_3SiNHAdam$ **3** (103.5 mg, 0.26 mmol, 1.3 equiv.) and 3DPA2FBN **PC** (6.4 mg, 10.0 µmol, 5 mol%). The title compound (21.6 mg, 0.09 mmol) was obtained in 47% yield as a yellowish oil, after purification by flash column chromatography (SiO_2; 0 – 5% EtOAc in cyclohexane). $\mathbf{R_f}$ = 0.43 (6:1 cyclohexane/EtOAc).

^{1}H NMR (300 MHz, $CDCl_3$): δ 8.09 (d, J = 5.2 Hz, 1H), 7.24 (t, J = 7.9 Hz, 1H), 7.02 – 7.01 (m, 1H), 6.80 – 6.76 (m, 3H), 6.73 – 6.72 (m, 1H), 4.11 (q, J = 7.2 Hz, 1H), 3.79 (s, 3H), 1.63 (d, J = 7.2 Hz, 3H). Spectrum

^{13}C NMR (75 MHz, $CDCl_3$): δ 164.3 (d, J = 238.6 Hz), 161.2 (d, J = 7.6 Hz), 160.0, 147.6 (d, J = 15.3 Hz), 145.4, 129.9, 121.0 (d, J = 4.0 Hz), 120.1, 114.0, 111.8, 108.4 (d, J = 37.3 Hz), 55.4, 44.3 (d, J = 2.8 Hz), 21.0. Spectrum

^{19}F NMR (470 MHz, $CDCl_3$): δ -68.5. Spectrum

HRMS (ESI+): calculated for $C_{14}H_{15}FNO$ $[M+H]^+$: 232.1132; found: 232.1123.

2-Chloro-4-(1-(3-methoxyphenyl)ethyl)pyridine (4z)

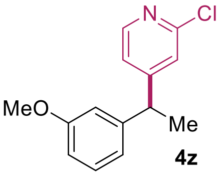

Prepared following the general procedure F using isonitrile **1d** (32.2 mg, 0.20 mmol, 1.0 equiv.), 2-chloroisonicotinonitrile **2d** (41.6 mg, 0.30 mmol, 1.5 equiv.), $(TMS)_3SiNHAdam$ **3** (103.5 mg, 0.26 mmol, 1.3 equiv.) and 3DPA2FBN **PC** (6.4 mg, 10.0 µmol, 5 mol%). The title compound (19.6 mg, 0.08 mmol) was obtained in 40% yield as a colorless oil, after purification by flash column chromatography (SiO_2; 0 – 7% EtOAc in cyclohexane). $\mathbf{R_f}$ = 0.43 (4:1 cyclohexane/EtOAc).

^{1}H NMR (300 MHz, $CDCl_3$): δ 8.26 (d, J = 5.2 Hz, 1H), 7.24 (t, J = 7.9 Hz, 1H), 7.18 – 7.17 (m, 1H), 7.06 – 7.04 (m, 1H), 6.80 – 6.76 (m, 2H), 6.72 – 6.71 (m, 1H), 4.07 (q, J = 7.2 Hz, 1H), 3.79 (s, 3H), 1.62 (d, J = 7.2 Hz, 3H). *Spectrum*

^{13}C NMR (75 MHz, $CDCl_3$): δ 160.0, 158.7, 151.9, 149.7, 145.3, 130.0, 123.4, 122.0, 120.1, 114.0, 111.9, 55.4, 44.2, 21.0. *Spectrum*

HRMS (ESI+): calculated for $C_{14}H_{15}ClNO$ $[M+H]^+$: 248.0837; found: 248.0839.

2-Methoxy-4-(1-(3-methoxyphenyl)ethyl)pyridine (4aa)

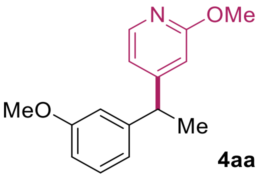

Prepared following the general procedure F using isonitrile **1d** (32.2 mg, 0.20 mmol, 1.0 equiv.), 2-methoxyisonicotinonitrile **2e** (40.2 mg, 0.30 mmol, 1.5 equiv.), $(TMS)_3SiNHAdam$ **3** (103.5 mg, 0.26 mmol, 1.3 equiv.) and 3DPA2FBN **PC** (6.4 mg, 10.0 µmol, 5 mol%). The title compound (20.7 mg, 0.09 mmol) was obtained in 43% yield as a yellow oil, after purification by flash column chromatography (SiO_2; 0 – 20% EtOAc in cyclohexane). $\mathbf{R_f}$ = 0.29 (9:1 cyclohexane/EtOAc).

^{1}H NMR (300 MHz, $CDCl_3$): δ 8.03 (d, J = 5.4 Hz, 1H), 7.22 (dt, J = 7.6, 1.1 Hz, 1H), 6.82 – 6.70 (m, 4H), 6.61 (t, J = 0.7 Hz, 1H), 4.02 (q, J = 7.2 Hz, 1H), 3.91 (s, 3H), 3.78 (s, 3H), 1.60 (d, J = 7.2 Hz, 3H). *Spectrum*

^{13}C NMR (75 MHz, $CDCl_3$): δ 164.7, 159.9, 158.0, 146.8, 146.3, 129.7, 120.2, 116.9, 114.0, 111.6, 109.6, 55.3, 53.5, 44.3, 21.0. *Spectrum*

HRMS (ESI+): calculated for $C_{15}H_{18}NO_2$ $[M+H]^+$: 244.1332; found: 244.1330.

4-(1-(3-Methoxyphenyl)ethyl)-2-phenylpyridine (4ab)

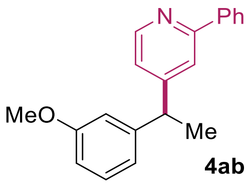

4ab

Prepared following the general procedure F using isonitrile **1d** (32.2 mg, 0.20 mmol, 1.0 equiv.), 2-phenylisonicotinonitrile **2f** (54.1 mg, 0.30 mmol, 1.5 equiv.), $(TMS)_3SiNHAdam$ **3** (103.5 mg, 0.26 mmol, 1.3 equiv.) and 3DPA2FBN **PC** (6.4 mg, 10.0 µmol, 5 mol%). The title compound (34.3 mg, 0.12 mmol) was obtained in 59% yield as a yellow oil, after purification by flash column chromatography (SiO_2; 0 – 50% Et_2O in pentane). $\mathbf{R_f}$ = 0.36 (2:1 pentane/Et_2O).

^{1}H NMR (300 MHz, $CDCl_3$): δ 8.61 (d, J = 5.2 Hz, 1H), 8.00 – 7.92 (m, 2H), 7.62 – 7.58 (m, 1H), 7.52 – 7.38 (m, 3H), 7.27 – 7.21 (m, 1H), 7.14 (dd, J = 5.2, 1.4 Hz, 1H), 7.86 – 7.75 (m, 3H), 4.18 (q, J = 7.2 Hz, 1H), 3.79 (s, 3H), 1.69 (d, J = 7.2 Hz, 3H). *Spectrum*

^{13}C NMR (75 MHz, $CDCl_3$): δ 160.0, 157.8, 155.8, 149.9, 146.3, 139.8, 129.8, 129.0, 128.8, 127.2, 121.6, 120.2, 120.1, 114.1, 111.6, 55.3, 44.6, 21.2. *Spectrum*

HRMS (ESI+): calculated for $C_{20}H_{20}NO$ $[M+H]^+$: 290.1539; found: 290.1535.

4-(1-(3-Methoxyphenyl)ethyl)-2-(phenylethynyl)pyridine (4ac)

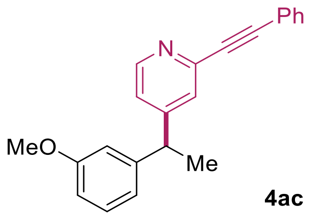

4ac

Prepared following the general procedure F using isonitrile **1d** (32.2 mg, 0.20 mmol, 1.0 equiv.), 2-(phenylethynyl)isonicotinonitrile **2g** (61.3 mg, 0.30 mmol, 1.5 equiv.), $(TMS)_3SiNHAdam$ **3** (103.5 mg, 0.26 mmol, 1.3 equiv.) and 3DPA2FBN **PC** (6.4 mg, 10.0 µmol, 5 mol%). The title compound (22.3 mg, 0.07 mmol) was obtained in 27% yield as a yellow oil, after purification by flash column chromatography (SiO_2; 0 – 40% Et_2O in pentane). $\mathbf{R_f}$ = 0.19 (2:1 pentane/Et_2O).

^{1}H NMR (300 MHz, $CDCl_3$): δ 8.50 (d, J = 5.3 Hz, 1H), 7.63 – 7.56 (m, 2H), 7.40 (br s, 1H), 7.39 – 7.31 (m, 3H), 7.25 (d, J = 8.0 Hz, 1H), 7.10 (dd, J = 5.1, 1.5 Hz, 1H), 6.83 – 6.73 (m, 3H), 4.10 (q, J = 7.2 Hz, 1H), 3.79 (s, 3H), 1.65 (d, J = 7.2 Hz, 3H). *Spectrum*

^{13}C NMR (75 MHz, $CDCl_3$): δ 160.0, 155.6, 150.1, 145.8, 143.5, 132.2, 129.9, 129.1, 128.5, 126.5, 122.5, 122.4, 120.2, 114.0, 111.8, 89.2, 89.0, 55.4, 44.3, 21.0. *Spectrum*

HRMS (ESI+): calculated for $C_{22}H_{20}NO$ $[M+H]^+$: 314.1539; found: 314.1533.

4-(1-(3-Methoxyphenyl)ethyl)-2-methylpyridine (4ad)

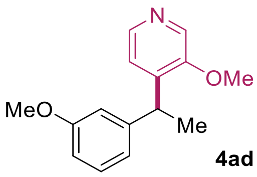

4ad

Prepared following the general procedure F using isonitrile **1d** (32.2 mg, 0.20 mmol, 1.0 equiv.), 3-methoxyisonicotinonitrile **2h** (40.2 mg, 0.30 mmol, 1.5 equiv.), $(TMS)_3SiNHAdam$ **3** (103.5 mg, 0.26 mmol, 1.3 equiv.) and 3DPA2FBN **PC** (6.4 mg, 10.0 µmol, 5 mol%). The title compound (29.6 mg, 0.12 mmol) was obtained in 61% yield as a yellowish oil, after purification by flash column chromatography (SiO_2; 20 – 60% Et_2O in pentane). $\mathbf{R_f}$ = 0.31 (1:2 pentane/Et_2O).

^{1}H NMR (300 MHz, $CDCl_3$): δ 8.20 (br s, 1H), 8.18 (d, J = 4.9 Hz, 1H), 7.23 – 7.17 (m, 1H), 7.05 (d, J = 4.8 Hz, 1H), 6.83 – 6.79 (m, 1H), 6.78 – 6.71 (m, 2H), 4.51 (q, J = 7.2 Hz, 1H), 3.88 (s, 3H), 3.77 (s, 3H), 1.56 (d, J = 7.2 Hz, 3H). *Spectrum*

^{13}C NMR (75 MHz, $CDCl_3$): δ 159.8, 153.3, 146.2, 143.3, 143.0, 133.4, 129.4, 122.2, 120.3, 114.1, 111.3, 56.2, 55.3, 37.2, 20.2. *Spectrum*

HRMS (ESI+): calculated for $C_{15}H_{18}NO_2$ $[M+H]^+$: 244.1332; found: 244.1332.

4-(1-(3-Methoxyphenyl)ethyl)-2-methylpyridine (4ae)

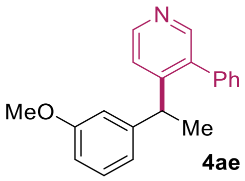

4ae

Prepared following the general procedure F using isonitrile **1d** (32.2 mg, 0.20 mmol, 1.0 equiv.), 3-phenylisonicotinonitrile **2i** (54.1 mg, 0.30 mmol, 1.5 equiv.), $(TMS)_3SiNHAdam$ **3** (103.5 mg, 0.26 mmol, 1.3 equiv.) and 3DPA2FBN **PC** (6.4 mg, 10.0 µmol, 5 mol%). The title compound (38.4 mg, 0.13 mmol) was obtained in 66% yield as a yellow oil, after purification by flash column chromatography (SiO_2; 0 – 40% Et_2O in pentane). $\mathbf{R_f}$ = 0.22 (2:1 pentane/Et_2O).

^{1}H NMR (300 MHz, $CDCl_3$): δ 8.51 (d, J = 5.3 Hz, 1H), 8.43 (br s, 1H), 7.43 – 7.40 (m, 3H), 7.23 (d, J = 5.0 Hz, 3H), 7.15 (t, J = 7.9 Hz, 1H), 6.71 (d, J = 8.3 Hz, 1H), 6.62 (d, J = 7.7 Hz, 1H), 6.57 (br s, 1H), 4.26 (q, J = 7.2 Hz, 1H), 3.74 (s, 3H), 1.53 (d, J = 7.2 Hz, 3H). *Spectrum*

^{13}C NMR (75 MHz, $CDCl_3$): δ 159.7, 152.6, 150.5, 149.0, 146.4, 137.9, 137.4, 129.6, 129.5, 128.5, 127.8, 122.2, 120.0, 113.9, 111.3, 55.2, 40.0, 21.6. *Spectrum*

HRMS (ESI+): calculated for $C_{20}H_{20}NO$ $[M+H]^+$: 290.1539; found: 290.1539.

4-(1-(3-Methoxyphenyl)ethyl)-2-methylpyridine (4af)

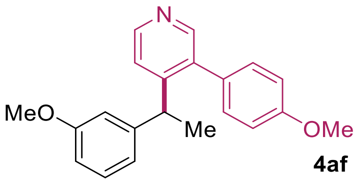

4af

Prepared following the general procedure F using isonitrile **1d** (32.2 mg, 0.20 mmol, 1.0 equiv.), 3-(4-methoxyphenyl)isonicotinonitrile **2j** (63.1 mg, 0.30 mmol, 1.5 equiv.), $(TMS)_3SiNHAdam$ **3** (103.5 mg, 0.26 mmol, 1.3 equiv.) and 3DPA2FBN **PC** (6.4 mg, 10.0 µmol, 5 mol%). The title compound (43.3 mg, 0.14 mmol) was obtained in 68% yield as a yellow oil, after purification by flash column chromatography (SiO_2; 0 – 40% Et_2O in pentane). $\mathbf{R_f}$ = 0.41 (1:1 pentane/Et_2O).

^{1}H NMR (300 MHz, $CDCl_3$): δ 8.49 (d, J = 5.2 Hz, 1H), 8.41 (br s, 1H), 7.20 (d, J = 5.2 Hz, 1H), 7.19 – 7.12 (m, 3H), 6.95 (d, J = 8.7 Hz, 2H), 6.71 (dd, J = 8.2, 2.6 Hz, 1H), 6.65 – 6.61 (m, 1H), 6.59 (m, 1H), 4.28 (q, J = 7.2 Hz, 1H), 3.86 (s, 3H), 3.74 (s, 3H), 1.52 (d, J = 7.2 Hz, 3H). *Spectrum*

^{13}C NMR (75 MHz, $CDCl_3$): δ 159.7, 159.4, 152.9, 150.7, 148.8, 146.5, 137.1, 130.8, 130.2, 129.5, 122.2, 120.1, 114.0, 113.9, 111.3, 55.5, 55.2, 40.1, 21.6. *Spectrum*

HRMS (ESI+): calculated for $C_{21}H_{22}NO_2$ $[M+H]^+$: 320.1645; found: 320.1647.

4-(1-(3-Methoxyphenyl)ethyl)-3-(phenylethynyl)pyridine (4ag)

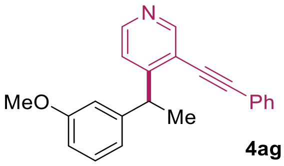

4ag

Prepared following the general procedure F using isonitrile **1d** (32.2 mg, 0.20 mmol, 1.0 equiv.), 3-(phenylethynyl)isonicotinonitrile **2k** (61.3 mg, 0.30 mmol, 1.5 equiv.), $(TMS)_3SiNHAdam$ **3** (103.5 mg, 0.26 mmol, 1.3 equiv.) and 3DPA2FBN **PC** (6.4 mg, 10.0 µmol, 5 mol%). The title compound (22.3 mg, 0.07 mmol) was obtained in 36% yield as a yellow oil, after purification by flash column chromatography (SiO_2; 0 – 40% Et_2O in pentane). $\mathbf{R_f}$ = 0.25 (2:1 pentane/Et_2O).

^{1}H NMR (300 MHz, $CDCl_3$): δ 8.70 (br s, 1H), 8.45 (br s, 1H), 7.55 – 7.35 (m, 2H), 7.40 – 7.35 (m, 3H), 7.23 (d, J = 7.9 Hz, 1H), 7.16 (d, J = 5.2 Hz, 1H), 6.89 – 6.81 (m, 2H), 6.77 (ddd, J = 8.2, 2.5, 0.8 Hz, 1H), 4.69 (q, J = 7.2 Hz, 1H), 3.75 (s, 3H), 1.67 (d, J = 7.2 Hz, 3H). *Spectrum*

^{13}C NMR (75 MHz, $CDCl_3$): δ 159.9, 156.4, 152.9, 149.0, 145.5, 131.8, 129.6, 128.9, 128.6, 122.9, 121.6, 120.3, 120.1, 114.1, 111.7, 96.6, 85.1, 55.3, 42.3, 20.6. *Spectrum*

HRMS (ESI+): calculated for $C_{22}H_{20}NO$ $[M+H]^+$: 314.1539; found: 314.1534.

3-(2-Fluorophenyl)-4-(1-(3-methoxyphenyl)ethyl)pyridine (4ah)

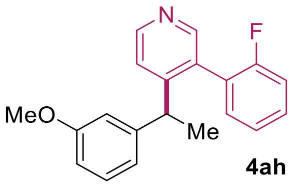

4ah

Prepared following the general procedure F using isonitrile **1d** (32.2 mg, 0.20 mmol, 1.0 equiv.), 3-(2-fluorophenyl)isonicotinonitrile **2l** (59.5 mg, 0.30 mmol, 1.5 equiv.), $(TMS)_3SiNHAdam$ **3** (103.5 mg, 0.26 mmol, 1.3 equiv.) and 3DPA2FBN **PC** (6.4 mg, 10.0 µmol, 5 mol%). The title compound (36.0 mg, 0.12 mmol) was obtained as a mixture of atropoisomers in 59% yield as a yellowish oil, after purification by flash column chromatography (SiO_2; 20 – 40% Et_2O in pentane). $\mathbf{R_f}$ = 0.39 (1:1 pentane/Et_2O).

^{1}H NMR (300 MHz, DMSO-d_6), T = 27 ºC: δ 8.54 (d, J = 5.2 Hz, 1H), 8.34 (s, 1H), 7.56 – 7.18 (m, 5H), 7.15 – 7.09 (m, 1H), 6.71 (d, J = 8.2 Hz, 1H), 6.53 – 6.48 (br s, 2H), 3.98 (br s, 1H), 3.65 (br s, 3H), 1.51 (br s, 3H). Spectrum

^{1}H NMR (300 MHz, DMSO-d_6), T = 80 ºC: δ 8.54 (d, J = 5.2 Hz, 1H), 8.34 (s, 1H), 7.55 – 7.47 (m, 1H), 7.38 (d, J = 5.2 Hz, 1H), 7.34 – 7.28 (m, 3H), 7.13 (t, J = 7.9 Hz, 1H), 6.72 (dd, J = 8.2, 2.9 Hz, 1H), 6.58 (d, J = 7.7 Hz, 1H), 6.51 (t, J = 2.1 Hz, 1H), 4.02 (q, J = 7.2 Hz, 1H), 3.67 (s, 3H), 1.51 (d, J = 7.2 Hz, 3H). Spectrum

^{13}C NMR (75 MHz, DMSO-d_6), T = 80 ºC: δ 159.01, 158.98 (d, J = 243.7 Hz), 152.7, 149.7, 149.1, 145.1, 131.5 (d, J = 3.0 Hz), 130.2, 130.0 (d, J = 8.1 Hz), 128.9, 124.4 (d, J =16.6 Hz), 124.1 (d, J = 3.6 Hz), 121.4, 119.0, 115.2 (d, J = 22.1 Hz), 112.9, 111.4, 54.6, 39.9, 20.1. Spectrum

^{19}F NMR (470 MHz, $CDCl_3$), T = 27 ºC: δ -113.2, -114.1. Spectrum

^{19}F NMR (470 MHz, DMSO-d_6), T = 80 ºC: δ -114.6. Spectrum

HRMS (ESI+): calculated for $C_{20}H_{19}FNO$ $[M+H]^+$: 308.1445; found: 308.1442.

3-(2-Methoxyphenyl)-4-(1-(3-methoxyphenyl)ethyl)pyridine (4ai)

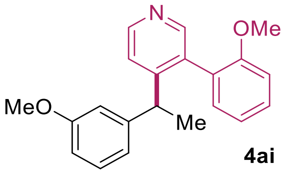

4ai

Prepared following the general procedure F using isonitrile **1d** (32.2 mg, 0.20 mmol, 1.0 equiv.), 3-(2-methoxyphenyl)isonicotinonitrile **2m** (63.1 mg, 0.30 mmol, 1.5 equiv.), $(TMS)_3SiNHAdam$ **3** (103.5 mg, 0.26 mmol, 1.3 equiv.) and 3DPA2FBN **PC** (6.4 mg, 10.0 µmol, 5 mol%). The title compound (51.2 mg, 0.16 mmol) was obtained as a mixture of atropoisomers in 80% yield as an orange oil, after purification by flash column chromatography (SiO_2; 20 – 40% Et_2O in pentane). $\mathbf{R_f}$ = 0.30 (1:1 pentane/Et_2O).

^{1}H NMR (300 MHz, DMSO-d_6), T = 27 ºC: δ 8.46 (d, *J* = 5.2 Hz, 1H), 8.42 (d, *J* = 5.2 Hz, 1H), 8.23 (br s, 2H), 7.47 – 7.37 (m, 3H), 7.24 – 7.20 (m, 1H), 7.18 – 7.06 (m, 6H), 7.03 – 7.01 (m, 2H), 6.75 (dd, *J* = 8.2, 2.6 Hz, 1H), 6.70 – 6.66 (m, 2H), 6.60 (t, *J* = 2.1 Hz, 1H), 6.54 (d, *J* = 7.7 Hz, 1H), 6.45 (t, *J* = 2.1 Hz, 1H), 3.96 (q, *J* = 7.2 Hz, 1H), 3.90 (q, *J* = 7.2 Hz, 1H), 3.78 (s, 3H), 3.69 (s, 3H), 3.63 (s, 3H), 3.58 (s, 3H), 1.53 (d, *J* = 7.2 Hz, 3H), 1.37 (d, *J* = 7.2 Hz, 3H). Spectrum

^{1}H NMR (300 MHz, DMSO-d_6), T = 120 ºC: δ 8.45 (d, *J* = 5.2 Hz, 1H), 8.24 (s, 1H), 7.45 – 7.39 (m, 1H), 7.26 (br s, 1H), 7.15 – 7.01 (m, 4H), 6.71 (dd, *J* = 8.1, 2.6 Hz, 1H), 6.62 (d, *J* = 7.7 Hz, 1H), 6.54 (br s, 1H), 4.00 (q, *J* = 7.2 Hz, 1H), 3.69 (s, 6H), 1.48 (d, *J* = 7.2 Hz, 3H). Spectrum

^{13}C NMR (75 MHz, DMSO-d_6), T = 120 ºC: δ 158.9, 156.3, 152.6, 149.6, 147.9, 133.1, 130.4, 128.9, 128.4, 126.0, 120.8, 119.9, 119.0, 113.0, 111.2, 54.8, 54.5, 39.6, 19.8. Spectrum

HRMS (ESI+): calculated for $C_{21}H_{22}NO_2$ $[M+H]^+$: 320.1645; found: 320.1643.

Methyl 3-(3-fluoropyridin-4-yl)-3-phenylpropanoate (4aj)

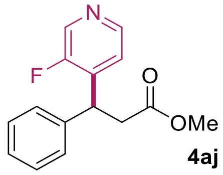

4aj

Prepared following the general procedure F using isonitrile **1a** (37.8 mg, 0.20 mmol, 1.0 equiv.), 3-fluoroisonicotinonitrile **2n** (36.6 mg, 0.30 mmol, 1.5 equiv.), $(TMS)_3SiNHAdam$ **3** (103.5 mg, 0.26 mmol, 1.3 equiv.) and 3DPA2FBN **PC** (6.4 mg, 10.0 µmol, 5 mol%). The title compound (39.8 mg, 0.15 mmol) was obtained in 77% yield as a yellowish oil, after purification by flash column chromatography (SiO_2; 0 – 50% EtOAc in cyclohexane). $\mathbf{R_f}$ = 0.30 (1:1 cyclohexane/EtOAc).

^{1}H NMR (300 MHz, $CDCl_3$): δ 8.39 – 8.33 (m, 2H), 7.35 – 7.26 (m, 2H), 7.26 – 7.18 (m, 4H), 4.84 (t, *J* = 7.9 Hz, 1H), 3.61 (s, 3H), 3.10 (dd, *J* = 7.9, 2.4 Hz, 2H). Spectrum

^{13}C NMR (75 MHz, $CDCl_3$): δ 171.5, 157.8 (d, *J* = 257.1 Hz), 145.9 (d, *J* = 5.1 Hz), 140.5, 139.4 (d, *J* = 11.9 Hz), 138.3 (d, *J* = 25.3 Hz), 129.1, 127.7, 127.5, 123.1, 52.1, 40.2, 38.7. Spectrum

^{19}F NMR (470 MHz, $CDCl_3$): δ -130.8. Spectrum

HRMS (ESI+): calculated for $C_{15}H_{15}FNO_2$ $[M+H]^+$: 260.1081; found: 260.1079.

2-(1-(3-Methoxyphenyl)ethyl)pyridine (4ak)

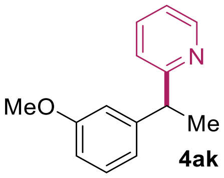

4ak

Prepared following the general procedure F using isonitrile **1d** (32.2 mg, 0.20 mmol, 1.0 equiv.), picolinonitrile **2o** (31.2 mg, 0.30 mmol, 1.5 equiv.), $(TMS)_3SiNHAdam$ **3** (103.5 mg, 0.26 mmol, 1.3 equiv.) and 3DPA2FBN **PC** (6.4 mg, 10.0 µmol, 5 mol%). The title compound (23.4 mg, 0.11 mmol)

was obtained in 55% yield as an orange oil, after purification by flash column chromatography (SiO_2; 0 – 40% EtOAc in cyclohexane). **R$_f$** = 0.42 (2:1 cyclohexane/EtOAc).

^{1}H NMR (300 MHz, $CDCl_3$): δ 8.56 (d, *J* = 5.0 Hz, 1H), 7.56 (td, *J* = 7.6, 1.8 Hz, 1H), 7.22 (d, *J* = 7.9 Hz, 1H), 7.15 – 7.06 (m, 2H), 6.92 – 6.82 (m, 2H), 6.77 – 6.71 (m, 1H), 4.27 (q, *J* = 7.2 Hz, 1H), 3.77 (s, 3H), 1.69 (d, *J* = 7.2 Hz, 3H). *Spectrum*

Spectroscopic data were in agreement with those described in the literature.[47]

2-(1-(3-Methoxyphenyl)ethyl)pyrimidine (4al)

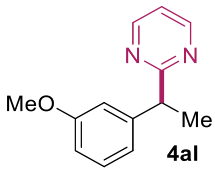

Prepared following the general procedure F using isonitrile **1d** (32.2 mg, 0.20 mmol, 1.0 equiv.), pyrimidine-2-carbonitrile **2p** (31.5 mg, 0.30 mmol, 1.5 equiv.), $(TMS)_3SiNHAdam$ **3** (103.5 mg, 0.26 mmol, 1.3 equiv.) and 3DPA2FBN **PC** (6.4 mg, 10.0 µmol, 5 mol%). After 72h, an additional mixture of pyrimidine-2-carbonitrile (31.5 mg, 0.30 mmol, 1.5 equiv.) and $(TMS)_3SiNHAdam$ **3** (103.5 mg, 0.26 mmol, 1.3 equiv.) was added under nitrogen and the final reaction was stirred under blue light irradiation an additional 72h. The title compound (11.6 mg, 0.05 mmol) was obtained in 27% yield as an orange oil, after purification by flash column chromatography (SiO_2; 0 – 60% EtOAc in cyclohexane). **R$_f$** = 0.34 (1:1 cyclohexane/EtOAc).

^{1}H NMR (300 MHz, $CDCl_3$): δ 8.69 (d, *J* = 4.9 Hz, 2H), 7.21 (t, *J* = 7.9 Hz, 1H), 7.11 (t, *J* = 4.9 Hz, 1H), 6.97 (d, *J* = 7.6 Hz, 1H), 6.94 (t, *J* = 2.1 Hz, 1H), 6.74 (dd, *J* = 8.2, 2.6 Hz, 1H), 4.42 (q, *J* = 7.2 Hz, 1H), 3.78 (s, 3H), 1.74 (d, *J* = 7.2 Hz, 3H). *Spectrum*

^{13}C NMR (75 MHz, $CDCl_3$): δ 173.1, 159.8, 157.3, 145.7, 129.6, 120.2, 118.9, 113.7, 112.1, 55.3, 48.9, 20.4. *Spectrum*

HRMS (ESI+): calculated for $C_{13}H_{15}N_2O$ $[M+H]^+$: 215.1179; found: 215.1181.

2-(1-(3-Methoxyphenyl)ethyl)pyrimidine (4am)

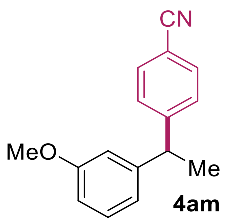

Prepared following the general procedure F using isonitrile **1d** (32.2 mg, 0.20 mmol, 1.0 equiv.), sodium acetate (49.2 mg, 0.60 mmol, 3.0 equiv.), terephthalonitrile **2q** (31.5 mg, 0.30 mmol, 1.5 equiv.), $(TMS)_3SiNHAdam$ **3** (103.5 mg, 0.26 mmol, 1.3 equiv.), 3DPA2FBN **PC** (6.4 mg, 10.0 µmol, 5 mol%) and acetonitrile as solvent (2.0 mL, 0.1 M). A mixture of the title compound and isonitrile **1d** (27.6 mg of a mixture 82:18) was isolated after purification by flash chromatography (SiO_2; 0 – 10% Et_2O in pentane). Isonitrile **1d** was removed by its derivatization by modified Passerini reaction.[48] The **1d** and **4am** mixture was treated with paraformaldehyde (8.0 mg), CH_2Cl_2 (0.5 mL) and propionic acid (4 µL) for 24 h. Upon completion, all volatiles

were removed under reduced pressure. The pure titled compound (21.1 mg, 0.09 mmol) was obtained in 44% yield as a pale-yellow oil, after purification by flash chromatography (SiO_2; 0 – 10% Et_2O in pentane). $\mathbf{R_f}$ = 0.50 (9:1 pentane/Et_2O).

^{1}H NMR (300 MHz, $CDCl_3$): δ 8.56 (d, *J* = 8.4 Hz, 2H), 7.31 (d, *J* = 8.2 Hz, 2H), 7.23 (t, *J* = 8.0 Hz, 1H), 6.79 – 6.69 (m, 3H), 4.16 (q, *J* = 7.2 Hz, 1H), 3.78 (s, 3H), 1.63 (d, *J* = 7.2 Hz, 3H). Spectrum

^{13}C NMR (75 MHz, $CDCl_3$): δ 160.0, 151.9, 146.5, 132.4, 129.8, 128.5, 120.1, 119.1, 114.1, 111.5, 110.2, 55.3, 45.0, 21.5. Spectrum

HRMS (APCI+): calculated for $C_{16}H_{16}NO$ $[M+H]^+$: 238.1226; found: 238.1226.

4-(1-(3-Methoxyphenyl)ethyl)-2-((1-(4-phenoxyphenoxy)propan-2-yl)oxy)pyridine (4an)

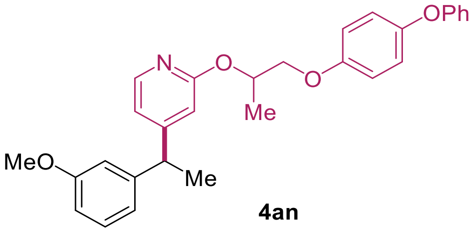

4an

Prepared following the general procedure F using isonitrile **1d** (12.1 mg, 0.075 mmol, 1.0 equiv.), (2-((1-(4-phenoxyphenoxy)propan-2-yl)oxy)pyridin-4-yl)triphenylphosphonium trifluoromethanesulfonate **5b** (65.9 mg, 0.090 mmol, 1.2 equiv.), $(TMS)_3SiNHAdam$ **3** (59.7 mg, 0.15 mmol, 2.0 equiv.) and 3DPA2FBN **PC** (2.4 mg, 3.8 µmol, 5 mol%). The title compound (16.2 mg, 0.036 mmol) was obtained in 47% yield as a yellowish oil as a 1:1 mixture of diasteroisomers, after purification by flash column chromatography (SiO_2; 0 – 5% EtOAc in cyclohexane). $\mathbf{R_f}$ = 0.46 (9:1 cyclohexane/EtOAc).

^{1}H NMR (300 MHz, $CDCl_3$): δ 8.02 (d, *J* = 5.2 Hz, 1H), 7.32 – 7.27 (m, 2H), 7.25 – 7.19 (m, 1H), 7.06 – 7.01 (m, 1H), 6.98 – 6.89 (m, 6H), 6.82 – 6.71 (m, 4H), 6.63 (br s, 1H), 5.61 – 5.51 (m, 1H), 4.17 (dd, *J* = 9.9, 5.2 Hz, 1H), 4.09 – 3.99 (m, 2H), 3.78 (s, 3H), 1.60 (d, *J* = 7.2 Hz, 3H), 1.47 (d, *J* = 6.4 Hz, 3H). Spectrum

^{13}C NMR (75 MHz, $CDCl_3$): δ 163.6, 159.9, 158.7, 155.4, 150.4, 146.5, 146.2, 129.74, 129.70, 122.6, 120.9, 120.2, 117.8, 117.0, 116.0, 114.0, 111.6, 110.3, 71.2, 69.6, 55.3, 44.4, 21.0, 17.1. Spectrum

HRMS (APCI+): calculated for $C_{29}H_{30}NO_4$ $[M+H]^+$: 456.2169; found: 456.2157.

***tert*-Butyl 4-(pyridine-4-yl)piperidine-1-carboxylate (4ao)**

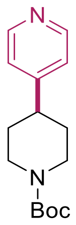

4ao

Prepared following the general procedure F using isonitrile **1y** (42.1 mg, 0.20 mmol, 1.0 equiv.), isonicotinonitrile **2a** (31.2 mg, 0.30 mmol, 1.5 equiv.), $(TMS)_3SiNHAdam$ **3** (103.5 mg, 0.26 mmol, 1.3 equiv.) and 3DPA2FBN **PC** (6.4 mg, 10.0 µmol, 5 mol%). The title compound (27.6 mg, 0.11 mmol) was obtained in 53% yield as a yellow oil, after purification by flash column chromatography (SiO_2; 0 – 40% EtOAc in cyclohexane). $\mathbf{R_f}$ = 0.34 (1:1 cyclohexane/EtOAc).

^{1}H NMR (300 MHz, $CDCl_3$): δ 8.52 (d, J = 6.1 Hz, 2H), 7.27 (d, J = 6.1 Hz, 2H), 4.28 – 4.25 (m, 1H), 2.81 (t, J = 12.8 Hz, 2H), 2.64 (tt, J = 12.2, 3.6 Hz, 1H), 1.85 – 1.81 (m, 2H), 1.69 – 1.58 (m, 3H), 1.48 (s, 9H). *Spectrum*

Spectroscopic data were in agreement with those described in the literature.[49]

4-((1r,3r,5r,7r)-Adamantan-2-yl)pyridine (4ap)

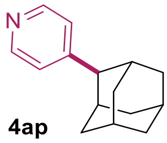

4ap

Prepared following the general procedure F using isonitrile **1z** (32.3 mg, 0.20 mmol, 1.0 equiv.), isonicotinonitrile **2a** (31.2 mg, 0.30 mmol, 1.5 equiv.), $(TMS)_3SiNHAdam$ **3** (103.5 mg, 0.26 mmol, 1.3 equiv.) and 3DPA2FBN **PC** (6.4 mg, 10.0 µmol, 5 mol%). The title compound (16.4 mg, 0.08 mmol) was obtained in 38% yield as a yellow oil, after purification by flash column chromatography (SiO_2; 0 – 60% EtOAc in cyclohexane). $\mathbf{R_f}$ = 0.51 (1:1 cyclohexane/EtOAc).

^{1}H NMR (300 MHz, $CDCl_3$): δ 8.52 (d, J = 5.3 Hz, 2H), 7.27 (d, J = 5.3 Hz, 2H), 2.97 (s, 1H), 2.47 (s, 2H), 2.05 – 1.89 (m, 5H), 1.81 – 1.70 (m, 5H), 1.63 – 1.54 (m, 2H). *Spectrum*

^{13}C NMR (75 MHz, $CDCl_3$): δ 153.9, 149.7, 122.6, 46.6, 39.0, 37.7, 32.1, 30.6, 27.9, 27.7. *Spectrum*

Spectroscopic data were in agreement with those described in the literature.[50]

4-(1-Benzhydrylazetidin-3-yl)pyridine (4aq)

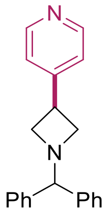

4aq

Prepared following the general procedure F using isonitrile **1aa** (49.7 mg, 0.20 mmol, 1.0 equiv.), isonicotinonitrile **2a** (31.2 mg, 0.30 mmol, 1.5 equiv.), $(TMS)_3SiNHAdam$ **3** (103.5 mg, 0.26 mmol, 1.3 equiv.) and 3DPA2FBN **PC** (6.4 mg, 10.0 µmol, 5 mol%). The title compound (24.6 mg, 0.08 mmol) was obtained in 41% yield as a yellowish oil, after purification by flash column chromatography (SiO_2; 0 – 40% EtOAc in cyclohexane). $\mathbf{R_f}$ = 0.23 (1:1 cyclohexane/EtOAc).

^{1}H NMR (300 MHz, $CDCl_3$): δ 8.54 (d, *J* = 5.1 Hz, 2H), 7.45 – 7. 34 (m, 4H), 7.31 – 7.26 (m, 6H), 7.21 – 7.18 (m, 2H), 4.40 (s, 1H), 3.64 – 3.59 (m, 3H), 3.18 – 3.14 (m, 2H). Spectrum

^{13}C NMR (75 MHz, $CDCl_3$): δ 152.3, 150.0, 142.0, 128.7, 127.6, 127.4, 122.5, 78.3, 59.9, 34.3. Spectrum

HRMS (ESI+): calculated for $C_{21}H_{21}N_2$ $[M+H]^+$: 301.1699; found: 301.1697.

***tert*-Butyl 4-methyl-4-(pyridin-4-yl)piperidine-1-carboxylate (4ar)**

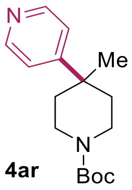

4ar

Prepared following the general procedure F using isonitrile **1ab** (44.9 mg, 0.20 mmol, 1.0 equiv.), isonicotinonitrile **2a** (31.2 mg, 0.30 mmol, 1.5 equiv.), $(TMS)_3SiNHAdam$ **3** (103.5 mg, 0.26 mmol, 1.3 equiv.) and 3DPA2FBN **PC** (6.4 mg, 10.0 µmol, 5 mol%). The title compound (48.7 mg, 0.18 mmol) was obtained in 87% yield as a yellow oil, after purification by flash column chromatography (SiO_2; 0 – 60% EtOAc in cyclohexane). $\mathbf{R_f}$ = 0.28 (1:1 cyclohexane/EtOAc).

^{1}H NMR (300 MHz, $CDCl_3$): δ 8.54 (br s, 2H), 7.22 (d, *J* = 5.4 Hz, 2H), 3.52 – 3.31 (m, 4H), 2.05 – 1.95 (m, 2H), 1.73 – 1.62 (m, 2H), 1.43 (s, 9H), 1.24 (s, 3H). Spectrum

^{13}C NMR (75 MHz, $CDCl_3$): δ 157.5, 154.9, 150.2, 121.3, 79.6, 40.4, 36.7, 36.1, 28.5, 28.2. Spectrum

HRMS (ESI+): calculated for $C_{16}H_{25}N_2O_2$ $[M+H]^+$: 277.1911; found: 277.1905.

***tert*-Butyl 4-(pyridin-4-yl)-4-(trifluoromethyl)piperidine-1-carboxylate (4as)**

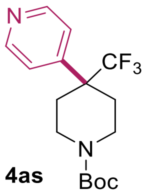

4as

Prepared following the general procedure F using isonitrile **1ac** (55.7 mg, 0.20 mmol, 1.0 equiv.), isonicotinonitrile **2a** (31.2 mg, 0.30 mmol, 1.5 equiv.), $(TMS)_3SiNHAdam$ **3** (103.5 mg, 0.26 mmol, 1.3 equiv.) and 3DPA2FBN **PC** (6.4 mg, 10.0 µmol, 5 mol%). The title compound (28.8 mg, 0.09 mmol) was obtained in 44% yield as a yellow oil, after purification by flash column chromatography (SiO_2; 0 – 60% EtOAc in cyclohexane). $\mathbf{R_f}$ = 0.36 (1:1 cyclohexane/EtOAc).

^{1}H NMR (300 MHz, $CDCl_3$): δ 8.67 (d, *J* = 5.0 Hz, 2H), 7.33 (d, *J* = 5.6 Hz, 2H), 4.03 (d, *J* = 10.7 Hz, 2H), 2.66 (t, *J* = 12.4 Hz, 2H), 2.40 (d, *J* = 12.3 Hz, 2H), 2.13 – 2.98 (m, 2H), 1.42 (s, 9H). Spectrum

^{13}C NMR (75 MHz, $CDCl_3$): δ 154.6, 150.6, 143.4, 126.7 (q, *J* = 282.6 Hz), 124.1, 80.2, 16.8 (q, *J* = 24.9 Hz), 39.1, 28.4, 27.8. Spectrum

^{19}F NMR (470 MHz, $CDCl_3$): δ -76.0. Spectrum

HRMS (ESI+): calculated for $C_{16}H_{21}F_3N_2NaO_2$ $[M+Na]^+$: 353.1447; found: 353.1446.

Methyl 1-(pyridin-4-yl)cyclohexane-1-carboxylate (4at)

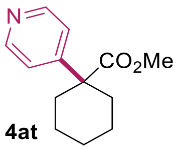

4at

Prepared following the general procedure F using isonitrile **1ad** (33.4 mg, 0.20 mmol, 1.0 equiv.), isonicotinonitrile **2a** (31.2 mg, 0.30 mmol, 1.5 equiv.), $(TMS)_3SiNHAdam$ **3** (103.5 mg, 0.26 mmol, 1.3 equiv.) and 3DPA2FBN **PC** (6.4 mg, 10.0 µmol, 5 mol%). The title compound (36.2 mg, 0.17 mmol) was obtained in 83% yield as an orangish oil, after purification by flash column chromatography (SiO_2; 0 – 40% EtOAc in cyclohexane). $\mathbf{R_f}$ = 0.47 (1:1 cyclohexane/EtOAc).

^{1}H NMR (300 MHz, $CDCl_3$): δ 8.54 (d, J = 6.3 Hz, 2H), 7.28 (d, J = 6.3 Hz, 2H), 3.65 (s, 3H), 2.49 – 2.39 (m, 2H), 1.78 – 1.59 (m, 5H), 1.54 – 1.40 (m, 2H), 1.36 – 1.27 (m, 1H). Spectrum

^{13}C NMR (75 MHz, $CDCl_3$): δ 174.7, 152.7, 150.2, 121.4, 52.4, 51.0, 34.2, 25.5, 23.5. Spectrum

HRMS (ESI+): calculated for $C_{13}H_{18}NO_2$ $[M+H]^+$: 220.1332; found: 220.1336.

***tert*-Butyl 3-methyl-3-(pyridin-4-yl)azetidine-1-carboxylate (4au)**

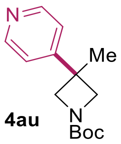

4au

Prepared following the general procedure F using isonitrile **1ae** (39.3 mg, 0.20 mmol, 1.0 equiv.), isonicotinonitrile **2a** (31.2 mg, 0.30 mmol, 1.5 equiv.), $(TMS)_3SiNHAdam$ **3** (103.5 mg, 0.26 mmol, 1.3 equiv.) and 3DPA2FBN **PC** (6.4 mg, 10.0 µmol, 5 mol%). The title compound (24.8 mg, 0.10 mmol) was obtained in 50% yield as a yellow oil, after purification by flash column chromatography (SiO_2; 0 – 40% EtOAc in cyclohexane). $\mathbf{R_f}$ = 0.10 (2:1 cyclohexane/EtOAc).

^{1}H NMR (300 MHz, $CDCl_3$): δ 8.58 (d, J = 5.0 Hz, 2H), 7.15 (d, J = 6.1 Hz, 2H), 4.14 (d, J = 8.3 Hz, 2H), 3.93 (d, J = 8.2 Hz, 2H), 1.62 (s, 3H), 1.45 (s, 9H). Spectrum

^{13}C NMR (75 MHz, $CDCl_3$): δ 156.5, 155.6, 150.2, 120.7, 80.0, 44.7, 37.5, 28.5, 28.1. Spectrum

HRMS (ESI+): calculated for $C_{14}H_{21}N_2O_2$ $[M+H]^+$: 249.1598; found: 249.1591.

4-(2-Methyl-4-phenylbutan-2-yl)pyridine (4av)

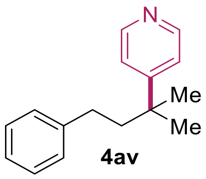
4av

Prepared following the general procedure F using isonitrile **1af** (34.7 mg, 0.20 mmol, 1.0 equiv.), isonicotinonitrile **2a** (31.2 mg, 0.30 mmol, 1.5 equiv.), $(TMS)_3$SiNHAdam **3** (103.5 mg, 0.26 mmol, 1.3 equiv.) and 3DPA2FBN **PC** (6.4 mg, 10.0 µmol, 5 mol%). The title compound (30.2 mg, 0.14 mmol) was obtained in 70% yield as a yellow oil, after purification by flash column chromatography (SiO_2; 0 – 40% EtOAc in cyclohexane). $\mathbf{R_f}$ = 0.37 (4:1 cyclohexane/EtOAc).

^{1}H NMR (300 MHz, $CDCl_3$): δ 8.55 (d, J = 5.5 Hz, 2H), 7.34 – 7.21 (m, 4H), 7.18 – 7.13 (m, 1H), 7.09 – 7.06 (m, 2H), 2.37 – 2.31 (m, 2H), 1.96 – 1.90 (m, 2H), 1.37 (s, 6H). Spectrum

Spectroscopic data were in agreement with those described in the literature.[51]

4-(2,4,4-Trimethylpentan-2-yl)pyridine (4aw)

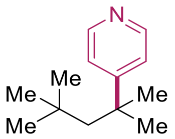
4aw

Prepared following the general procedure F using isonitrile **1ag** (27.8 mg, 0.20 mmol, 1.0 equiv.), isonicotinonitrile **2a** (31.2 mg, 0.30 mmol, 1.5 equiv.), $(TMS)_3$SiNHAdam **3** (103.5 mg, 0.26 mmol, 1.3 equiv.) and 3DPA2FBN **PC** (6.4 mg, 10.0 µmol, 5 mol%). The title compound (21.9 mg, 0.12 mmol) was obtained in 57% yield as a yellow oil, after purification by flash column chromatography (SiO_2; 0 – 40% EtOAc in cyclohexane). $\mathbf{R_f}$ = 0.61 (1:1 cyclohexane/EtOAc).

^{1}H NMR (300 MHz, $CDCl_3$): δ 8.50 (br s, 2H), 7.33 (d, J = 6.1 Hz, 2H), 1.78 (s, 2H), 1.36 (s, 6H), 0.74 (s, 9H). Spectrum

^{13}C NMR (75 MHz, $CDCl_3$): δ 160.7, 148.9, 121.9, 56.4, 38.9, 32.6, 31.9, 30.9. Spectrum

HRMS (ESI+): calculated for $C_{13}H_{22}N$ $[M+H]^+$: 192.1747; found: 192.1741.

4-(1-Benzyloxy)-2-methylpropan-2-yl)pyridine (4ax)

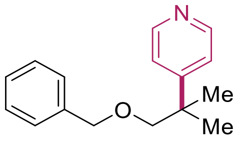
4ax

Prepared following the general procedure F using isonitrile **1ah** (37.9 mg, 0.20 mmol, 1.0 equiv.), isonicotinonitrile **2a** (31.2 mg, 0.30 mmol, 1.5 equiv.), $(TMS)_3$SiNHAdam **3** (103.5 mg, 0.26 mmol, 1.3 equiv.) and 3DPA2FBN **PC** (6.4 mg, 10.0 µmol, 5 mol%). The title compound (26.5 mg, 0.11 mmol) was obtained in 56% yield as a yellow oil, after purification by flash column chromatography (SiO_2; 0 – 40% EtOAc in cyclohexane). $\mathbf{R_f}$ = 0.38 (1:1 cyclohexane/EtOAc).

^{1}H NMR (300 MHz, $CDCl_3$): δ 8.53 (br s, 2H), 7.35 – 7.27 (m, 5H), 7.21 (d, *J* = 7.3 Hz, 2H), 4.47 (s, 2H), 3.46 (s, 2H), 1.34 (s, 6H). Spectrum

^{13}C NMR (75 MHz, $CDCl_3$): δ 156.9, 149.5, 138.4, 128.4, 127.7, 127.5, 121.9, 79.0, 73.4, 39.3, 25.6. Spectrum

HRMS (ESI+): calculated for $C_{16}H_{20}NO$ $[M+H]^+$: 242.1539; found: 242.1537.

1-(Pyridin-4-ylmethyl)-1*H*-benzo[*d*][1,2,3]triazole (4ay)

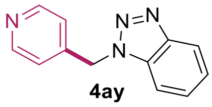

4ay

Prepared following the general procedure F using isonitrile **1ai** (31.6 mg, 0.20 mmol, 1.0 equiv.), isonicotinonitrile **2a** (31.2 mg, 0.30 mmol, 1.5 equiv.), $(TMS)_3SiNHAdam$ **3** (103.5 mg, 0.26 mmol, 1.3 equiv.) and 3DPA2FBN **PC** (6.4 mg, 10.0 µmol, 5 mol%). The title compound (23.9 mg, 0.11 mmol) was obtained in 57% yield as a white solid, after purification by flash column chromatography (SiO_2; 30 – 70% EtOAc in cyclohexane). $\mathbf{R_f}$ = 0.12 (1:2 cyclohexane/EtOAc).

^{1}H NMR (300 MHz, $CDCl_3$): δ 8.61 (br s, 2H), 8.14 – 8.10 (m, 1H), 7.50 – 7.40 (m, 2H), 7.38 – 7.32 (m, 1H), 7.13 (d, *J* = 5.1 Hz, 2H), 5.88 (s, 2H). Spectrum

Spectroscopic data were in agreement with those described in the literature.[52]

Methyl 3-phenyl-2-(pyridine-4-yl)propanoate (4az)

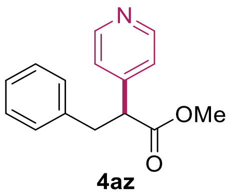

4az

Prepared following the general procedure F using isonitrile **1aj** (37.8 mg, 0.20 mmol, 1.0 equiv.), isonicotinonitrile **2a** (31.2 mg, 0.30 mmol, 1.5 equiv.), $(TMS)_3SiNHAdam$ **3** (103.5 mg, 0.26 mmol, 1.3 equiv.) and 3DPA2FBN **PC** (6.4 mg, 10.0 µmol, 5 mol%). The title compound (22.1 mg, 0.09 mmol) was obtained in 46% yield as a yellow oil, after purification by flash column chromatography (SiO_2; 0 – 40% EtOAc in cyclohexane). $\mathbf{R_f}$ = 0.42 (1:1 cyclohexane/EtOAc).

^{1}H NMR (300 MHz, $CDCl_3$): δ 8.53 (d, *J* = 5.8 Hz, 2H), 7.25 – 7.17 (m, 5H), 7.10 – 7.05 (m, 2H), 3.87 – 3.79 (m, 1H), 3.64 (s, 3H), 3.41 (dd, *J* = 13.6, 8.4 Hz, 1H), 3.02 (dd, *J* = 13.8, 7.3 Hz, 1H). Spectrum

^{13}C NMR (75 MHz, $CDCl_3$): δ 172.7, 150.1, 147.3, 138.1, 129.0, 128.6, 126.8, 123.4, 53.1, 52.4, 39.4. Spectrum

HRMS (ESI+): calculated for $C_{15}H_{16}NO_2$ $[M+H]^+$: 242.1176; found: 242.1171.

Methyl 6-(((benzyloxy)carbonyl)amino)-2-(pyridine-4-yl)hexanoate (4ba)

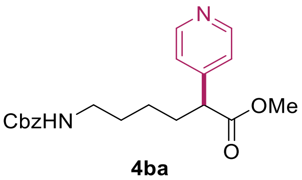

4ba

Prepared following the general procedure F using isonitrile **1ak** (60.9 mg, 0.20 mmol, 1.0 equiv.), isonicotinonitrile **2a** (31.2 mg, 0.30 mmol, 1.5 equiv.), $(TMS)_3SiNHAdam$ **3** (103.5 mg, 0.26 mmol, 1.3 equiv.) and 3DPA2FBN **PC** (6.4 mg, 10.0 µmol, 5 mol%). The title compound (31.2 mg, 0.09 mmol) was obtained in 44% yield as a yellow oil, after purification by flash column chromatography (SiO_2; 5 – 60% EtOAc in cyclohexane). $\mathbf{R_f}$ = 0.25 (1:2 cyclohexane/EtOAc).

^{1}H NMR (300 MHz, $CDCl_3$): δ 8.54 (d, J = 4.4 Hz, 2H), 7.35 – 7.30 (m, 5H), 7.22 (d, J = 4.4 Hz, 2H), 5.08 (s, 2H), 4.78 (br s, 1H), 3.66 (s, 3H), 3.52 (t, J = 7.6 Hz, 1H), 3.19 – 3.12 (m, 2H), 2.12 – 2.03 (m, 1H), 1.79 – 1.74 (m, 1H), 1.54 – 1.48 (m, 2H), 1.33 – 1.28 (m, 2H). *Spectrum*

^{13}C NMR (75 MHz, $CDCl_3$): δ 173.1, 156.5, 149.9, 148.1, 136.7, 128.7, 128.2, 123.4, 66.8, 52.4, 51.1, 40.8, 32.8, 29.8, 24.7. *Spectrum*

HRMS (ESI+): calculated for $C_{20}H_{25}N_2O_4$ $[M+H]^+$: 357.1809; found: 357.1815.

Methyl 6-(((benzyloxy)carbonyl)amino-2-(pyridine-4-yl)hexanoate (4bb)

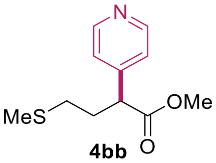

4bb

Prepared following the general procedure F using isonitrile **1al** (60.9 mg, 0.20 mmol, 1.0 equiv.), isonicotinonitrile **2a** (31.2 mg, 0.30 mmol, 1.5 equiv.), $(TMS)_3SiNHAdam$ **3** (103.5 mg, 0.26 mmol, 1.3 equiv.) and 3DPA2FBN **PC** (6.4 mg, 10.0 µmol, 5 mol%). The title compound (21.6 mg, 0.10 mmol) was obtained in 48% yield as an orange oil, after purification by flash column chromatography (SiO_2; 0 – 45% EtOAc in cyclohexane). $\mathbf{R_f}$ = 0.37 (1:1 cyclohexane/EtOAc).

^{1}H NMR (300 MHz, $CDCl_3$): δ 8.56 (d, J = 6.1 Hz, 2H), 7.23 (d, J = 6.1 Hz, 2H), 3.88 – 3.77 (m, 1H), 3.68 (s, 3H), 2.48 – 2.31 (m, 3H), 2.07 (s, 3H), 2.05 – 1.97 (m, 1H). *Spectrum*

^{13}C NMR (75 MHz, $CDCl_3$): δ 172.8, 150.0, 147.6, 123.5, 52.6, 49.4, 32.0, 31.7, 15.4. *Spectrum*

HRMS (ESI+): calculated for $C_{11}H_{16}NO_2S$ $[M+H]^+$: 226.0896; found: 226.0897.

tert-Butyl 3-(4-hydroxyphenyl)-2-(pyridin-4-yl)propanoate (4bc)

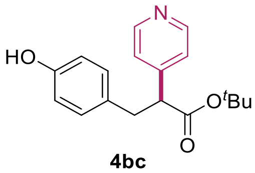

4bc

Prepared following the general procedure F using isonitrile **1am** (49.5 mg, 0.20 mmol, 1.0 equiv.), isonicotinonitrile **2a** (31.2 mg, 0.30 mmol, 1.5 equiv.), $(TMS)_3SiNHAdam$ **3** (103.5 mg, 0.26 mmol, 1.3 equiv.) and 3DPA2FBN **PC** (6.4 mg, 10.0 µmol, 5 mol%). The title compound (26.9 mg, 0.09 mmol) was obtained in 45% yield as a yellow oil, after purification by flash column chromatography (SiO_2; 0 – 50% EtOAc in cyclohexane). $\mathbf{R_f}$ = 0.31 (1:1 cyclohexane/EtOAc).

^{1}H NMR (300 MHz, $CDCl_3$): δ 8.48 (d, J = 5.2 Hz, 2H), 7.22 (d, J = 5.3 Hz, 2H), 6.89 – 6.87 (m, 2H), 6.70 – 6.67 (m, 2H), 3.69 (t, J = 7.8 Hz, 1H), 3.26 (dd, J = 13.8, 7.8 Hz, 1H), 2.90 (dd, J = 13.8, 7.9 Hz, 1H), 1.36 (s, 9H). *Spectrum*

^{13}C NMR (75 MHz, $CDCl_3$): δ 171.4, 155.5, 149.11, 149.08, 130.2, 129.4, 123.9, 115.5, 81.9, 54.5, 38.8, 28.0. *Spectrum*

HRMS (ESI+): calculated for $C_{18}H_{21}NO_3$ $[M+H]^+$: 300.1594; found: 300.1601.

tert-Butyl 3-(4-hydroxyphenyl)-2-(pyridin-4-yl)propanoate (4bd)

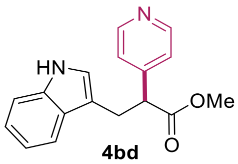

4bd

Prepared following the general procedure F using isonitrile **1an** (45.7 mg, 0.20 mmol, 1.0 equiv.), isonicotinonitrile **2a** (31.2 mg, 0.30 mmol, 1.5 equiv.), $(TMS)_3SiNHAdam$ **3** (103.5 mg, 0.26 mmol, 1.3 equiv.) and 3DPA2FBN **PC** (6.4 mg, 10.0 µmol, 5 mol%). The title compound (27.1 mg, 0.10 mmol) was obtained in 48% yield as a yellow oil, after purification by flash column chromatography (SiO_2; 0 – 30% EtOAc in cyclohexane). $\mathbf{R_f}$ = 0.25 (4:1 cyclohexane/EtOAc).

^{1}H NMR (300 MHz, $CDCl_3$): δ 8.53 (d, J = 5.6 Hz, 2H), 8.05 (br s, 1H), 7.57 (d, J = 7.7 Hz, 1H), 7.34 (d, J = 8.1 Hz, 1H), 7.26 – 7.25 (m, 2H), 7.22 – 7.18 (m, 1H), 7.15 – 7.12 (m, 1H), 6.82 (d, J = 2.3 Hz, 1H), 3.99 (t, J = 7.6 Hz, 1H), 3.65 (s, 3H), 3.59 (dd, J = 14.6, 8.1 Hz, 1H), 3.19 (dd, J = 14.6, 7.3 Hz, 1H). *Spectrum*

^{13}C NMR (75 MHz, $CDCl_3$): δ 173.1, 149.8, 148.3, 136.3, 127.2, 123.5, 122.7, 122.3, 119.7, 118.5, 112.4, 111.4, 52.5, 52.0, 29.3. *Spectrum*

HRMS (ESI+): calculated for $C_{17}H_{17}N_2O_2$ $[M+H]^+$: 281.1285; found: 281.1288.

4-(5-(2,5-Dimethylphenoxy)-2-methylpentan-2-yl)pyridine (4be)

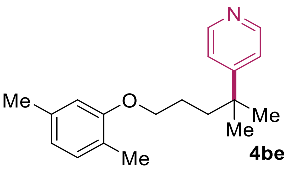

4be

Prepared following the general procedure F using isonitrile **1ao** (46.3 mg, 0.20 mmol, 1.0 equiv.), isonicotinonitrile **2a** (31.2 mg, 0.30 mmol, 1.5 equiv.), $(TMS)_3SiNHAdam$ **3** (103.5 mg, 0.26 mmol, 1.3 equiv.) and 3DPA2FBN **PC** (6.4 mg, 10.0 µmol, 5 mol%). The title compound (47.7 mg, 0.17 mmol) was obtained in 84% yield as a yellow oil, after purification by flash column chromatography (SiO_2; 0 – 40% EtOAc in cyclohexane). $\mathbf{R_f}$ = 0.15 (4:1 cyclohexane/EtOAc).

^{1}H NMR (300 MHz, $CDCl_3$): δ 8.54 (br s, 2H), 7.31 (d, *J* = 5.1 Hz, 2H), 7.00 (d, *J* = 7.6 Hz, 1H), 6.65 (d, *J* = 7.6 Hz, 1H), 6.55 (s, 1H), 3.85 (t, *J* = 6.1 Hz, 2H), 2.29 (s, 3H), 2.17 (s, 3H), 1.88 – 1.80 (m, 2H), 1.61 – 1.49 (m, 2H), 1.35 (s, 6H). Spectrum

Spectroscopic data were in agreement with those described in the literature.[53]

4-(1-((3r,5r,7r)-Adamantan-1-yl)ethyl)pyridine (4bf)

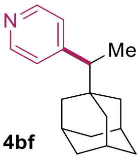

4bf

Prepared following the general procedure F using isonitrile **1ap** (37.9 mg, 0.20 mmol, 1.0 equiv.), isonicotinonitrile **2a** (31.2 mg, 0.30 mmol, 1.5 equiv.), $(TMS)_3SiNHAdam$ **3** (103.5 mg, 0.26 mmol, 1.3 equiv.) and 3DPA2FBN **PC** (6.4 mg, 10.0 µmol, 5 mol%). The title compound (46.1 mg, 0.19 mmol) was obtained in 96% yield as a pale-yellow solid, after purification by flash column chromatography (SiO_2; 0 – 30% EtOAc in cyclohexane). $\mathbf{R_f}$ = 0.43 (1:1 cyclohexane/EtOAc).

^{1}H NMR (300 MHz, $CDCl_3$): δ 8.47 (br s, 2H), 7.04 (d, *J* = 5.8 Hz, 2H), 2.35 (q, *J* = 7.3 Hz, 1H), 1.98 – 1.90 (m, 3H), 1.68 (s, 1H), 1.64 (s, 2H), 1.59 – 1.49 (m, 6H), 1.46 – 1.36 (m, 3H), 1.21 (d, *J* = 7.3 Hz, 3H). Spectrum

^{13}C NMR (75 MHz, $CDCl_3$): δ 153.5, 149.0, 125.0, 50.7, 40.0, 37.1, 35.2, 28.8, 13.7. Spectrum

HRMS (ESI+): calculated for $C_{17}H_{24}N$ $[M+H]^+$: 242.1903; found: 242.1901.

m.p.: 52 – 54 °C.

4-(((1*S*,4a*S*,10a*S*)-7-Isopropyl-1,4a-dimethyl-1,2,3,4,4a,9,10,10a-octahydrophenanthren-1-yl)methyl)pyridine (4bg)

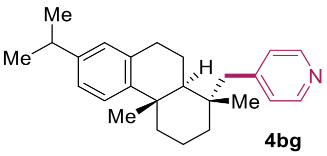

4bg

Prepared following the general procedure F using isonitrile **1aq** (59.1 mg, 0.20 mmol, 1.0 equiv.), isonicotinonitrile **2a** (31.2 mg, 0.30 mmol, 1.5 equiv.), $(TMS)_3SiNHAdam$ **3** (103.5 mg, 0.26 mmol, 1.3 equiv.) and 3DPA2FBN **PC** (6.4 mg, 10.0 µmol, 5 mol%). The reaction mixture was irradiated for 48 h. The title compound (42.1 mg, 0.12 mmol)

was obtained in 61% yield as a yellowish oil, after purification by flash column chromatography (SiO_2; 0 – 25% Et_2O in pentane). **R$_f$** = 0.35 (4:1 cyclohexane/EtOAc).

^{1}H NMR (300 MHz, $CDCl_3$): δ 8.48 (d, *J* = 5.5 Hz, 2H), 7.13 (d, *J* = 8.1 Hz, 1H), 7.10 (d, *J* = 5.7 Hz, 2H), 6.98 (dd, *J* = 8.1, 2.1 Hz, 1H), 6.90 (d, *J* = 2.1 Hz, 1H), 3.00 – 2.95 (m, 1H), 2.93 – 2.85 (m, 1H), 2.85 – 2.79 (m, 1H), 2.63 (s, 2H), 2.26 – 2.22 (m, 1H), 2.08 – 2.03 (m, 1H), 1.89 – 1.81 (m, 1H), 1.73 – 1.67 (m, 1H), 1.63 – 1.58 (m, 1H), 1.35 – 1.27 (m, 4H), 1.23 (s, 6H), 1.21 (s, 3H), 1.02 (s, 3H). Spectrum

^{13}C NMR (75 MHz, $CDCl_3$): δ 149.2, 148.4, 147.4, 145.8, 134.5, 127.0, 126.8, 124.3, 124.1, 49.6, 47.7, 38.4, 38.04, 37.96, 37.7, 33.6, 30.1, 25.7, 24.1, 20.7, 19.5, 18.9. Spectrum

HRMS (ESI+): calculated for $C_{25}H_{34}N$ $[M+H]^+$: 348.2686; found: 348.2694.

***tert*-Butyl (R)-2-(pyridin-4-ylmethyl)pyrrolidine-1-carboxylate (4bh)**

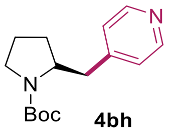

4bh

Prepared following the general procedure F using isonitrile **1ar** (53.9 mg, 0.20 mmol, 1.0 equiv.), isonicotinonitrile **2a** (31.2 mg, 0.30 mmol, 1.5 equiv.), $(TMS)_3SiNHAdam$ **3** (103.5 mg, 0.26 mmol, 1.3 equiv.) and 3DPA2FBN **PC** (6.4 mg, 10.0 µmol, 5 mol%). The title compound (28.3 mg, 0.11 mmol) was obtained as a mixture of rotamers in 52% yield as a yellowish oil, after purification by flash column chromatography (SiO_2; 0 – 55% EtOAc in cyclohexane). **R$_f$** = 0.19 (1:1 cyclohexane/EtOAc).

^{1}H NMR (300 MHz, $C_2D_2Cl_4$), T = 27 ºC: δ 8.42 (br s, 2H), 7.11 – 7.04 (m, 2H), 3.98 – 3.86 (m, 1H), 3.29 – 3.16 (m, 2H), 3.04 – 2.92 (m, 1H), 2.60 – 2.44 (m, 1H), 1.72 – 1.52 (m, 4H), 1.38 (s, 9H). Spectrum

^{1}H NMR (300 MHz, $C_2D_2Cl_4$), T = 90 ºC: δ 8.46 (br s, 2H), 7.09 (br s, 2H), 4.01 – 3.98 (m, 1H), 3.35 (dt, *J* = 10.7, 7.6 Hz, 1H), 3.21 (dd, *J* = 10.7, 7.5 Hz, 1H), 3.02 (dd, *J* = 13.3, 4.0 Hz, 1H), 2.61 (dd, *J* = 13.3, 8.7 Hz, 1H), 1.86 – 1.78 (m, 1H), 1.73 – 1.67 (m, 2H), 1.60 – 1.56 (m, 1H), 1.43 (s, 9H). Spectrum

^{13}C NMR (300 MHz, $C_2D_2Cl_4$), T = 90 ºC: δ 154.5, 149.4, 149.0, 125.2, 79.5, 58.0, 46.8, 40.1, 30.2, 28.9, 23.4. Spectrum

HRMS (ESI+): calculated for $C_{15}H_{23}N_2O_2$ $[M+H]^+$: 263.1754; found: 263.1757.

4-(((3a*R*,5*R*,5a*S*,8a*S*,8b*R*)-2,2,7,7-Tetramethyltetrahydro-5*H*-bis([1,3]dioxolo)[4,5-*b*:4',5'-*d*]pyran-5-yl)methyl)pyridine (4bi)

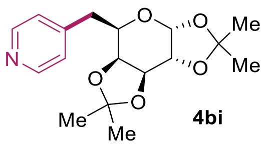

4bi

Prepared following the general procedure F using isonitrile **1as** (53.9 mg, 0.20 mmol, 1.0 equiv.), isonicotinonitrile **2a** (31.2 mg, 0.30 mmol, 1.5 equiv.), $(TMS)_3SiNHAdam$ **3** (103.5 mg, 0.26 mmol, 1.3 equiv.) and 3DPA2FBN **PC** (6.4 mg, 10.0 µmol, 5 mol%). The title compound (15.6 mg, 0.05 mmol) was obtained in 24% yield (57% brsm) as an orange oil, after purification by flash column chromatography (SiO_2; 0 – 60% EtOAc in cyclohexane). $\mathbf{R_f}$ = 0.15 (2:1 cyclohexane/EtOAc).

^{1}H NMR (300 MHz, $CDCl_3$): δ 8.51 (d, *J* = 6.1 Hz, 2H), 7.27 – 7.26 (m, 2H), 5.54 (d, *J* = 5.0 Hz, 1H), 4.60 (dd, *J* = 7.9, 2.4 Hz, 1H), 4.31 (dd, *J* = 5.1, 2.4 Hz, 1H), 4.08 (dd, *J* = 7.9, 1.9 Hz, 1H), 4.02 (ddd, *J* = 7.8, 5.5, 1.8 Hz, 1H), 3.00 (dd, *J* = 14.7, 8.2 Hz, 1H), 2.89 (dd, *J* = 14.7, 5.5 Hz, 1H), 1.50 (s, 3H), 1.48 (s, 3H), 1.36 (s, 3H), 1.32 (s, 3H). *Spectrum*

^{13}C NMR (75 MHz, $CDCl_3$): δ 149.4, 148.2, 124.7, 109.5, 108.7, 96.8, 72.3, 71.0, 70.5, 67.5, 35.8, 26.2, 26.1, 25.0, 24.6. *Spectrum*

HRMS (ESI+): calculated for $C_{17}H_{24}NO_5$ $[M+H]^+$: 322.1649; found: 322.1650.

4-(4-(Isocyanomethyl)benzyl)pyridine (4bj)

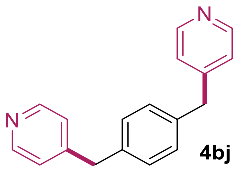

4bj

Prepared following the general procedure F using isonitrile **1u** (31.2 mg, 0.20 mmol, 1.0 equiv.), isonicotinonitrile **2a** (62.5 mg, 0.60 mmol, 3.0 equiv.), $(TMS)_3SiNHAdam$ **3** (207 mg, 0.52 mmol, 2.6 equiv.) and 3DPA2FBN **PC** (6.4 mg, 10.0 µmol, 5 mol%). The title compound (20.4 mg, 0.08 mmol) was obtained in 39% yield as a yellow oil, after purification by flash column chromatography (SiO_2; 0 – 20% MeOH in CH_2Cl_2). $\mathbf{R_f}$ = 0.47 (9:1 CH_2Cl_2/MeOH). 11% yield of monofunctionalized product **4bk** was also isolated from the column chromatography.

^{1}H NMR (300 MHz, $CDCl_3$): δ 8.53 – 7.46 (m, 4H), 7.12 (s, 4H), 7.11 – 7.07 (m, 4H), 3.94 (s, 4H). *Spectrum*

^{13}C NMR (75 MHz, $CDCl_3$): δ 150.1, 150.0, 137.5, 129.5, 124.3, 41.0. *Spectrum*

HRMS (ESI+): calculated for $C_{18}H_{17}N_2$ $[M+H]^+$: 261.1386; found: 261.1385.

4-(4-(Isocyanomethyl)benzyl)pyridine (4bk)

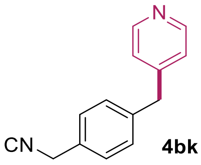

4bk

Prepared following the general procedure F using isonitrile **1u** (38.4 mg, 0.25 mmol, 1.2 equiv.), isonicotinonitrile **2a** (24.0 mg, 0.23 mmol, 1.2 equiv.), $(TMS)_3SiNHAdam$ **3** (79.6 mg, 0.20 mmol, 1.0 equiv.) and 3DPA2FBN **PC** (4.93 mg, 7.7 µmol, 4 mol%). The title compound (21.2 mg, 0.10 mmol) was obtained in 51% yield as a yellow oil, after purification by flash column chromatography (SiO_2; 0 – 20% MeOH in CH_2Cl_2). $\mathbf{R_f}$ = 0.32 (99:1 CH_2Cl_2/MeOH). 30% yield of difunctionalized product **4bj** and 13% of starting isonitrile **1u** were also isolated from the column chromatography.

^{1}H NMR (300 MHz, $CDCl_3$): δ 8.53 – 7.48 (m, 2H), 7.30 (d, J = 8.3 Hz, 2H), 7.22 (d, J = 8.2 Hz, 2H), 7.11 – 7.06 (m, 2H), 4.62 (s, 2H), 3.98 (s, 2H). Spectrum

^{13}C NMR (75 MHz, $CDCl_3$): δ 158 (t, J = 5.2 Hz), 150.1, 149.6, 139.5, 131.0, 129.8, 127.2, 124.2, 45.4, (t, J = 7.1 Hz), 41.0. Spectrum

HRMS (ESI+): calculated for $C_{14}H_{13}N_2$ $[M+H]^+$: 209.1073; found: 209.1071.

3-Fluoro-4-(4-(pyridin-4-ylmethyl)benzyl)pyridine (4bl)

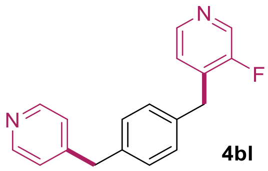

4bl

Prepared following the general procedure F using product **4bk** (17.6 mg, 0.08 mmol, 1.0 equiv.), 3-fluoroisonicotinonitrile **2n** (15.5 mg, 0.15 mmol, 1.5 equiv.), $(TMS)_3SiNHAdam$ **3** (43.7 mg, 0.11 mmol, 1.3 equiv.) and 3DPA2FBN **PC** (2.7 mg, 4.2 µmol, 5 mol%). The title compound (12.5 mg, 0.05 mmol) was obtained in 53% yield (70% brsm) as a yellowish oil, after purification by flash column chromatography (SiO_2; 0 – 5% *i*PrOH in DCM). $\mathbf{R_f}$ = 0.54 (9:1 CH_2Cl_2/MeOH).

^{1}H NMR (300 MHz, $CDCl_3$): δ 8.51 (br s, 2H), 8.41 (br s, 1H), 8.30 (d, J = 4.9 Hz, 1H), 7.17 – 7.15 (m, 4H), 7.13 – 7.11 (m, 2H), 7.06 (dd, J = 6.4, 4.9 Hz, 1H), 3.99 (s, 2H), 3.97 (s, 2H). Spectrum

^{13}C NMR (75 MHz, $CDCl_3$): δ 158.3 (d, J = 254.5 Hz), 151.3, 148.9, 145.9 (d, J = 5.2 Hz), 138.1 (d, J = 24.4 Hz), 137.3, 136.7 (d, J = 13.6 Hz), 136.4, 129.6, 129.5, 125.4, 124.7 (br s), 41.1, 33.9 (d, J = 2.7 Hz). Spectrum

^{19}F NMR (470 MHz, $CDCl_3$): δ -132.4. Spectrum

HRMS (ESI+): calculated for $C_{18}H_{16}FN_2$ $[M+H]^+$: 279.1292; found: 279.1293.

D. Gram-Scale Experiment

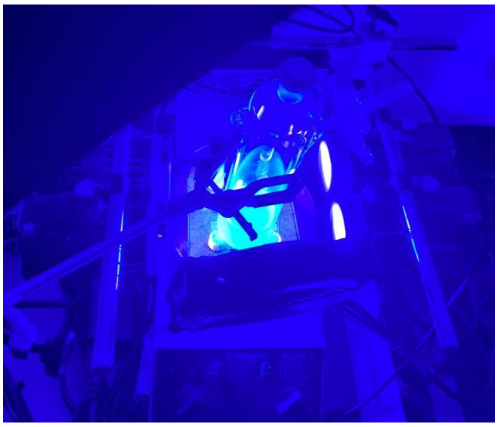

Figure S4. Gram scale synthesis reaction setup of **4a**.

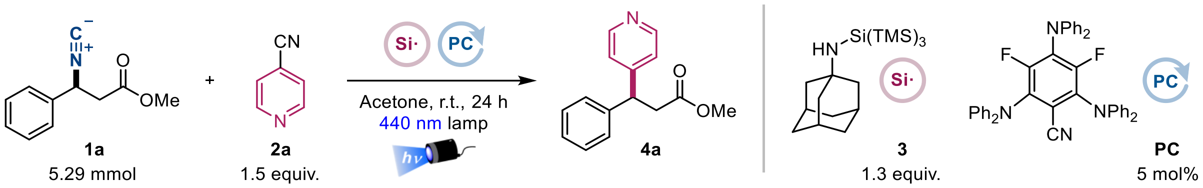

An oven-dried Schlenk flask equipped with a magnetic stir bar was charged with isonitrile **1a** (1.0 g, 5.29 mmol, 1.0 equiv.), isonicotinonitrile **2a** (825 mg, 7.93 mmol, 1.5 equiv.), $TMS_3SiNHAd$ **3** (2.73 g, 6.87 mmol, 1.3 equiv.) and 3DPA2FBN **PC** (169 mg, 0.26 mmol, 0.05 equiv.). The Schlenk flask was evacuated three times, purged with argon, and charged with dry degassed acetone (53 mL, 0.1 M). The reaction mixture was irradiated with two Kessil PR160L-blue LED lamp (max 45 W High Luminous DEX 2100 LED, λ_{max} = 440 nm) as in ***Figure S4*** and it was monitored by ^{1}H NMR. After 24h, a ratio of 1:3 (**1a**:**4a**) was observed. Then, an additional mixture of isonicotinonitrile **2a** (234.2 mg, 2.25 mmol, 0.43 equiv.) and $(TMS)_3SiNHAdam$ **3** (775.9 mg, 1.95 mmol, 0.37 equiv.) was added under nitrogen and the final reaction was stirred under blue light an additional 24h. Upon completion, all the volatiles were removed under reduced pressure, and the crude mixture was subjected to purification by column chromatography (SiO_2; 0 – 40% EtOAc in cyclohexane) to obtain the compound **4a** as a brown oil (0.926 g, 3.84 mmol) in 73% yield.

5. Orthogonal Functionalization of Amino Acids

5.1. Synthesis of formylphenylalanine 7

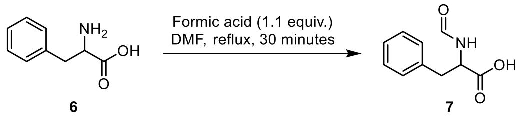

According to a modified literature procedure,[54] a mixture of formic acid (0.30 mL, 7.60 mmol, 1.1 equiv.), DMF (3.6 mL, 2.0 M), and phenylalanine (1.17 g, 7.10 mmol, 1.0 equiv.) was heated to reflux for 1 hour. Evaporation of the solvent and recrystallization from toluene, gave the desired formamide **7** (1.36, 7.10 mmol) in a quantitative yield. Spectroscopic data were in agreement with those described in the literature.

^{1}H NMR (300 MHz, DMSO-d_6): δ 8.26 (d, J = 8.2 Hz, 1H), 7.96 – 7.95 (m, 1H), 7.29 – 7.19 (m, 5H), 4.51 – 4.21 (m, 1H), 3.08 (dd, J = 13.8, 5.0 Hz, 1H), 2.90 – 2.83 (m, 1H). *Spectrum*

5.2. Synthesis of benzyl 5-phenyl-4-(pyridin-4-yl)pentanoate 9

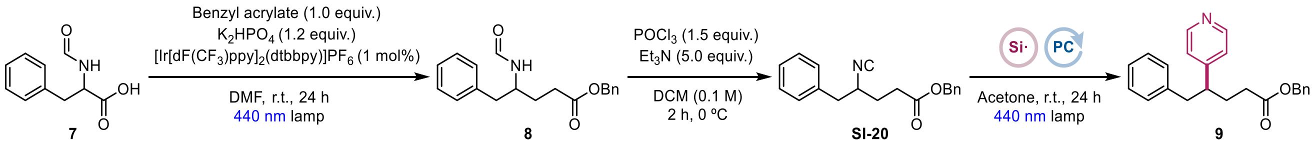

Step 1: *According to a modified literature procedure*,[55] an oven-dried 4 mL vial equipped with a Teflon septum and magnetic stir bar was charged with Ir[dF(CF_3)ppy)]$_2$(dtbbpy)PF_6 (4.5 mg, 4.0 µmol, 0.01 equiv), formylphenylalanine **7** (77.3 mg, 0.40 mmol, 1.0 equiv), benzyl acrylate (61.2 µL, 0.40 mmol, 1.0 equiv), K_2HPO_4 (83.6 mg, 0.24 mmol, 1.2 equiv), and DMF (1.0 mL, 0.40 M). The reaction mixture was degassed by bubbling nitrogen stream for 15 min, then irradiated with a Kessil PR160L-blue LED lamp (max 45 W High Luminous DEX 2100 LED, λ_{max} = 440 nm). After 24h, the reaction mixture was diluted with saturated aqueous $NaHCO_3$ solution. The reaction mixture was stirred for 12 h at r.t., and the resulting red mixture was quenched with $NaHCO_3$. The aqueous layer was extracted with Et_2O (x3), the combined organic layers were dried over anhydrous Na_2SO_4 and concentrated under reduced pressure. The crude was purified by flash column chromatography (SiO_2; 0 – 55% EtOAc in cyclohexane) to afford pure product **8** as a yellowish oil as a mixture of rotamers (65.9 mg, 0.21 mmol) in 53% yield. **R$_f$** = 0.12 (2:1 cyclohexane/EtOAc).

^{1}H NMR (500 MHz, $CDCl_3$): δ 8.03 – 7.62 (m, 1H), 7.37 – 7.28 (m, 7H), 7.24 – 7.21 (m, 1H), 7.18 – 7.10 (m, 2H), 5.70 – 5.49 (m, 1H), 5.13 – 5.07 (m, 2H), 4.32 – 3.54 (m, 1H), 2.88 – 2.66 (m, 2H), 2.49 – 2.36 (m, 2H), 2.02 – 1.89 (m, 1H), 1.79 – 1.69 (m, 1H). *Spectrum*

^{13}C NMR (125 MHz, $CDCl_3$) (only 1 rotamer): δ 173.5, 161.0, 137.3, 135.9, 129.5, 128.72, 128.67, 128.5, 128.4, 126.8, 66.6, 49.1, 41.2, 31.2, 28.9. *Spectrum*

HRMS (APCI): calculated for $C_{19}H_{22}NO_3$ $[M+H]^+$: 312.1594; found: 312.1588.

Step 2: $POCl_3$ (29.6 µL, 0.32 mmol, 1.5 equiv.) in DCM (1.0 mL) was added dropwise to a solution of the **8** (65.9 mg, 0.21 mmol, 1.0 equiv.) in Et_3N (0.15 mL, 1.1 mmol, 5.0 equiv.) and DCM (1.2 mL) at –78 ºC. The reaction mixture was stirred for 2 h at 0 ºC, and the resulting mixture was quenched with $NaHCO_3$. The aqueous layer was extracted with DCM (x3), the combined organic layers were dried over anhydrous $MgSO_4$ and concentrated under reduced pressure. The crude was purified by flash column chromatography (SiO_2; 0 – 8% EtOAc in cyclohexane) to afford pure product **SI-20** (44.7 mg, 0.15 mmol) in 72% yield as a yellowish oil. **R_f** = 0.32 (9:1 cyclohexane/EtOAc).

^{1}H NMR (300 MHz, $CDCl_3$): δ 7.40 – 7.33 (m, 7H), 7.31 – 7.28 (m, 1H), 7.23 – 7.22 (m, 2H), 5.17 – 5.11 (m, 2H), 3.88 – 3.82 (m, 1H), 2.97 – 2.88 (m, 2H), 2.68 – 2.54 (m, 2H), 2.05 – 1.99 (m, 1H), 1.92 – 1.84 (m, 1H). *Spectrum*

^{13}C NMR (75 MHz, $CDCl_3$): δ 172.2, 157.1 (t, J = 4.7 Hz, -N**C**), 135.9, 135.7, 129.3, 128.8, 128.7, 128.5, 128.4, 127.4, 66.7, 55.8 (t, J = 5.6 Hz, **C**-NC), 41.6, 30.4, 29.6. *Spectrum*

HRMS (ESI+): calculated for $C_{19}H_{20}NO_2$ $[M+H]^+$: 294.1489; found: 294.1483.

Step 3: Prepared following the general procedure F using isonitrile **SI-20** (42.6 mg, 0.15 mmol, 1.0 equiv.), isonicotinonitrile **2a** (22.7 mg, 0.22 mmol, 1.5 equiv.), $(TMS)_3SiNHAdam$ **3** (75.1 mg, 0.19 mmol, 1.3 equiv.) and 3DPA2FBN **PC** (4.7 mg, 7.3 µmol, 5 mol%). The desired product **9** (22.5 mg, 0.07 mmol) was obtained in 45% yield (56% brsm) as a yellowish oil, after purification by flash column chromatography (SiO_2; 33 – 60% Et_2O in pentane). **R_f** = 0.16 (2:1 pentane/Et_2O).

^{1}H NMR (300 MHz, $CDCl_3$): δ 8.46 (br s, 2H), 7.38 – 7.29 (m, 5H), 7.22 – 7.14 (m, 3H), 7.00 (d, J = 5.2 Hz, 2H), 6.98 – 6.94 (m, 2H), 5.10 – 5.00 (m, 2H), 2.96 – 2.82 (m, 3H), 2.27 – 1.94 (m, 4H). *Spectrum*

^{13}C NMR (75 MHz, $CDCl_3$): δ 172.9, 153.5, 149.5, 139.0, 135.9, 129.1, 128.7, 128.5, 128.4, 126.5, 123.6, 66.4, 47.0, 42.9, 32.2, 30.1. *Spectrum*

HRMS (APCI+): calculated for $C_{23}H_{24}NO_2$ $[M+H]^+$: 346.1802; found: 346.1796.

5.3. Synthesis of 1,4-diphenyl-3-(pyridin-4-yl)butan-1-one 12

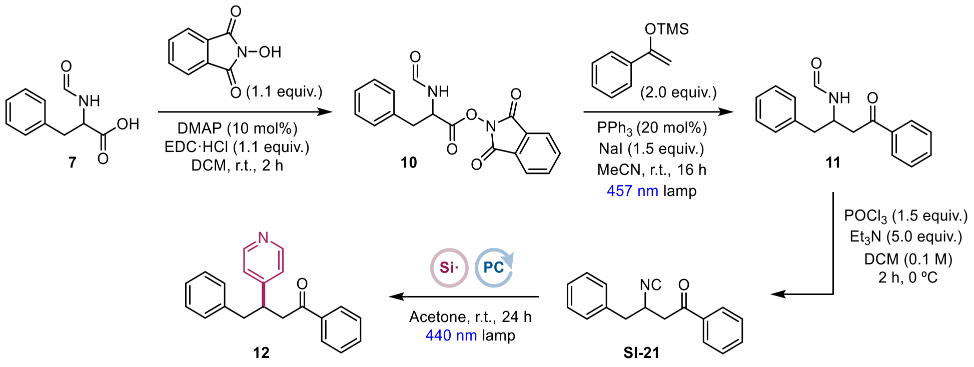

Step 1: A 20 mL glass vial was charged with formylphenylalanine **7** (114.0 mg, 0.59 mmol, 1.0 equiv.), EDC·HCl (124.4 mg, 0.65 mmol, 1.1 equiv.), DMAP (7.2 mg, 0.06 mmol, 0.1 equiv.), N-hydroxyphthalimide (105.9 mg, 0.65 mmol, 1.1 equiv.), followed by DCM (4.5 mL). The resulting solution was stirred at room temperature for 2 h. The organic phase was washed with 1 M HCl (x2), with saturated aqueous $NaHCO_3$ solution and dried over anhydrous Na_2SO_4. The solvent was then removed under reduced pressure affording pure product **10** (189.3 mg, 0.56 mmol) in 95% yield as a yellowish foam.

^{1}H NMR (300 MHz, $CDCl_3$): δ 8.16 (br s, 1H), 7.90 – 7.85 (m, 2H), 7.83 – 7.78 (m, 2H), 7.34 – 7.28 (m, 5H), 6.44 (d, J = 8.4 Hz, 1H), 5.45 – 5.38 (m, 1H), 3.39 (dd, J = 14.3, 5.9 Hz, 1H), 3.31 (dd, J = 14.2, 6.1 Hz, 1H). Spectrum

^{13}C NMR (75 MHz, $CDCl_3$): δ 168.1, 161.5, 160.8, 135.1, 134.3, 129.8, 128.9, 128.8, 127.7, 124.3, 49.9, 37.8. Spectrum

HRMS (APCI): calculated for $C_{18}H_{15}N_2O_5$ $[M+H]^+$: 339.0975; found: 339.0972.

Spectroscopic data were in agreement with those described in the literature.[56]

Step 2: *According to a modified literature procedure*,[57] an oven-dried 4 mL vial equipped with a Teflon septum and magnetic stir bar was charged with redox active ester **10** (46.7 mg, 0.14 mmol, 1.0 equiv.), NaI (31.0 mg, 0.21 mmol, 1.50 equiv.) and PPh_3 (7.2 mg, 0.03 mmol, 0.20 equiv.). The vial was capped and evacuated three times *via* an inlet needle then purged with argon, and charged with dry degassed MeCN (1.4 mL, 0.1 M) and trimethyl((1-phenylvinyl)oxy)silane (56.6 µL, 0.28 mmol, 2.0 equiv.). The reaction mixture was irradiated with a Kessil PR160L-blue LED lamp (max 45 W High Luminous DEX 2100 LED, λ_{max} = 457 nm). After 16h, the reaction mixture was quenched with water. The aqueous layer was extracted with EtOAc (x3), the combined

organic layers were dried over anhydrous Na_2SO_4 and concentrated under reduced pressure. The crude was purified by flash column chromatography (SiO_2; 0 – 60% EtOAc in cyclohexane) to afford pure product **11** as a yellow oil as a mixture of rotamers (31.3 mg, 0.12 mmol) in 85% yield. $\mathbf{R_f}$ = 0.10 (2:1 cyclohexane/EtOAc).

^{1}H NMR (300 MHz, $CDCl_3$): δ 8.10 – 8.06 (m, 1H), 7.92 – 7.85 (m, 2H), 7.70 – 7.54 (m, 1H), 7.48 – 7.42 (m, 2H), 7.31 – 7.27 (m, 1H), 7.25 – 7.16 (m, 4H), 6.52 – 6.42 (m, 1H), 4.73 – 4.20 (m, 1H), 3.34 – 3.25 (m, 1H), 3.17 – 3.06 (m, 2H), 3.02 – 2.87 (m, 1H). *Spectrum*

^{13}C NMR (75 MHz, $CDCl_3$) (only 1 rotamer): δ 199.4, 161.0, 138.0, 136.8, 133.8, 129.4, 128.9, 128.8, 128.2, 126.9, 47.0, 40.4, 39.7. *Spectrum*

HRMS (APCI): calculated for $C_{17}H_{18}NO_2$ $[M+H]^+$: 268.1332; found: 268.1330.

Step 3: $POCl_3$ (35.1 µL, 0.38 mmol, 1.5 equiv.) in DCM (1.0 mL) was added dropwise to a solution of the **11** (67.1 mg, 0.25 mmol, 1.0 equiv.) in Et_3N (0.18 mL, 1.3 mmol, 5.0 equiv.) and DCM (1.5 mL) at –78 ºC. The reaction mixture was stirred for 2 h at 0 ºC, and the resulting mixture was quenched with $NaHCO_3$. The aqueous layer was extracted with DCM (x3), the combined organic layers were dried over anhydrous $MgSO_4$ and concentrated under reduced pressure. The crude was purified by flash column chromatography (SiO_2; 0 – 10% EtOAc in cyclohexane) to afford pure product **SI-21** (46.8 mg, 0.19 mmol) in 75% yield as a colorless solid. $\mathbf{R_f}$ = 0.43 (3:1 cyclohexane/EtOAc).

^{1}H NMR (300 MHz, $CDCl_3$): δ 7.94 – 7.91 (m, 2H), 7.64 – 7.58 (m, 1H), 7.51 – 7.46 (m, 2H), 7.37 – 7.27 (m, 5H), 4.50 (p, *J* = 6.6 Hz, 1H), 3.43 (dd, *J* = 17.6, 6.5 Hz, 1H), 3.24 (dd, *J* = 17.7, 6.6 Hz, 1H), 3.12 – 3.05 (m, 1H), 2.99 (dd, *J* = 13.7, 7.6 Hz, 1H). *Spectrum*

^{13}C NMR (75 MHz, $CDCl_3$): δ 195.4, 157.1 (t, *J* = 4.7 Hz, -N**C**), 136.2, 135.8, 134.0, 129.6, 129.0, 128.9, 128,2, 127.6, 51.7 (t, *J* = 6.5 Hz, **C**-NC), 43.0, 40.9. *Spectrum*

HRMS (APCI): calculated for $C_{17}H_{16}NO$ $[M+H]^+$: 250.1226; found: 250.1222.

m.p.: 75 – 76 ºC.

Step 4: Prepared following the general procedure F using isonitrile **SI-21** (17.0 mg, 0.07 mmol, 1.0 equiv.), isonicotinonitrile **2a** (10.6 mg, 0.10 mmol, 1.5 equiv.), $(TMS)_3SiNHAdam$ **3** (35.3 mg, 0.09 mmol, 1.3 equiv.) and 3DPA2FBN **PC** (2.2 mg, 3.4 µmol, 5 mol%). The desired product **12** (8.6 mg, 0.03 mmol) was obtained in 42% yield (55% brsm) as an orange oil, after purification by flash column chromatography (SiO_2; 0 – 70% EtOAc in cyclohexane). $\mathbf{R_f}$ = 0.29 (1:1 cyclohexane/EtOAc).

^{1}**H NMR** (500 MHz, $CDCl_3$): δ 8.45 (d, *J* = 6.1 Hz, 2H), 7.86 (d, *J* = 6.8 Hz, 2H), 7.55 (t, *J* = 7.5 Hz, 1H), 7.43 (t, *J* = 7.8 Hz, 2H), 7.24 – 7.21 (m, 2H), 7.19 – 7.15 (m, 1H), 7.10 (d, *J* = 6.1 Hz, 2H), 7.07 – 7.05 (m, 2H), 3.69 (p, *J* = 7.2 Hz, 1H), 3.37 (dd, *J* = 17.2, 7.6 Hz, 1H), 3.31 (dd, *J* = 17.3, 6.2 Hz, 1H), 3.02 (dd, *J* = 13.6, 7.1 Hz, 1H), 2.93 (dd, *J* = 13.6, 7.9 Hz, 1H). *Spectrum*

^{13}**C NMR** (125 MHz, $CDCl_3$): δ 198.1, 153.3, 149.9, 138.9, 137.0, 133.4, 129.3, 128.8, 128.5, 128.1, 126.6, 123.3, 43.3, 42.41, 42.41. *Spectrum*

HRMS (APCI): calculated for $C_{21}H_{20}NO$ $[M+H]^+$: 302.1539; found: 302.1533.

6. Competition Studies: XAT vs Heteroarylation and Decarboxylation vs Heteroarylation

To test the feasibility of the heteroarylation reaction in the presence of aryl and alkyl halides and in the presence of carboxylic acids, we run six competition experiments (***Scheme S1***). These experiments showed that the heteroarylation is compatible with the presence of alkyl and aryl chloride (**eq. 3** and **4**) as well as carboxylic acids (**eq. 5** and **6**). However, in the presence of aryl and alkyl bromides (**eq. 1** and **2**) the XAT reaction of the silyl radical competes with the addition of the silyl radical to the isonitrile.

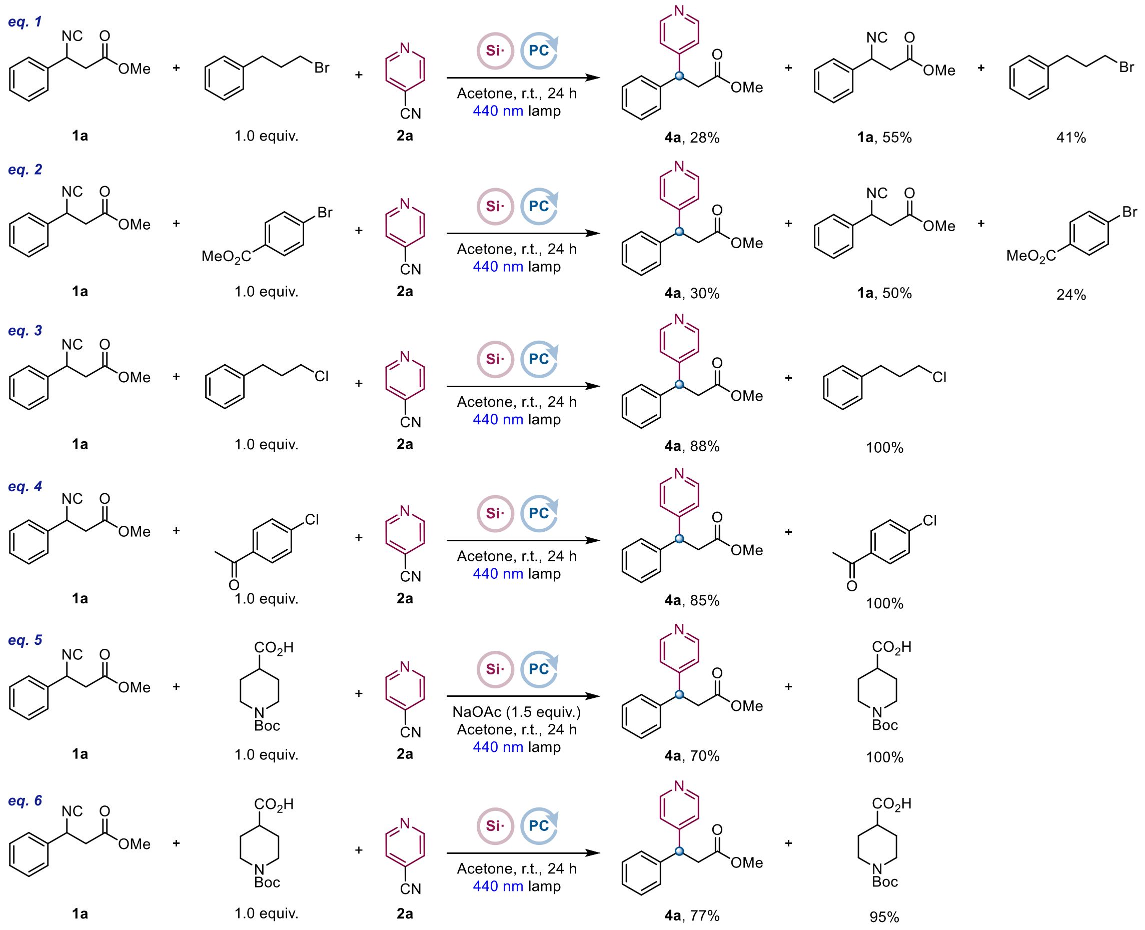

Scheme S1. Competition experiments.

7. Mechanistic Studies

A. Stern-Volmer Quenching Studies

B. 1. Stern-Volmer Quenching Studies for 3DPA2FBN

Stern-Volmer quenching studies were conducted on an JASCO FP-8600 Spectrofluorometer. Samples of **3DPA2FBN**, 4-cyanopyridine **2a** and $TMS_3SiNHAd$ **3**, were prepared as degassed solutions in acetone. The concentration of **3DPA2FBN** was $2.0 \cdot 10^{-4}$ M (solution A). Samples were sealed in a 1 cm quartz cuvette. The sample solution was excited at 440 nm, with emission intensity detected at 482 nm. The emission light was acquired from 450 nm to 650 nm.

*B.1.1 Stern-Volmer quenching studies with isonitrile **1a***

To 2.5 mL of solution A (**3DPA2FBN**), portions of 40 µL of a 0.10 M solution of isonitrile **1a** were added. A fluorescence spectrum of the mixture was recorded after each addition. Results are shown in ***Figure S5***.

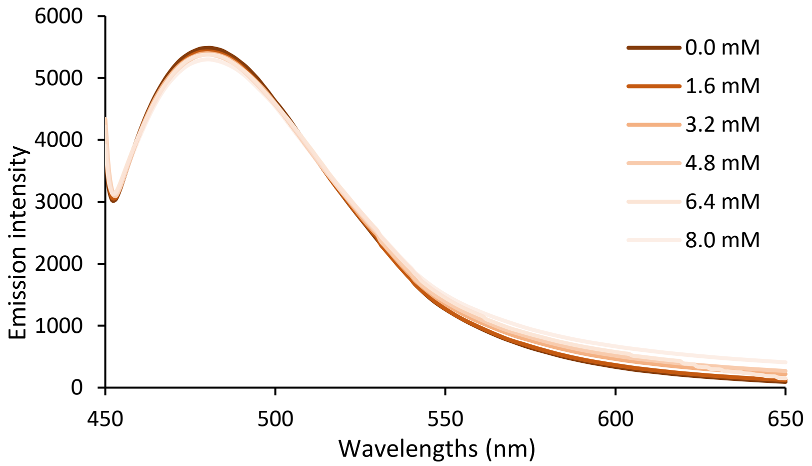

Figure S5. Quenching of the photocatalyst emission ($2.0 \cdot 10^{-4}$ M in acetone) in the presence of increasing amounts of isonitrile **1a**.

The Stern-Volmer plot, reported in ***Figure S6***, shows a linear correlation between the relative intensity I_0/I and the concentration of **1a**. Based on the following equation (*equation S1*), it is possible to calculate the Stern-Volmer constant K_{SV}.

$$\frac{I_0}{I} = K_{SV} \cdot [Q] + 1 \qquad \textit{(equation S1)}$$

Therefore, the K_{SV} for reagent **1a** is 4.2 M^{-1}.

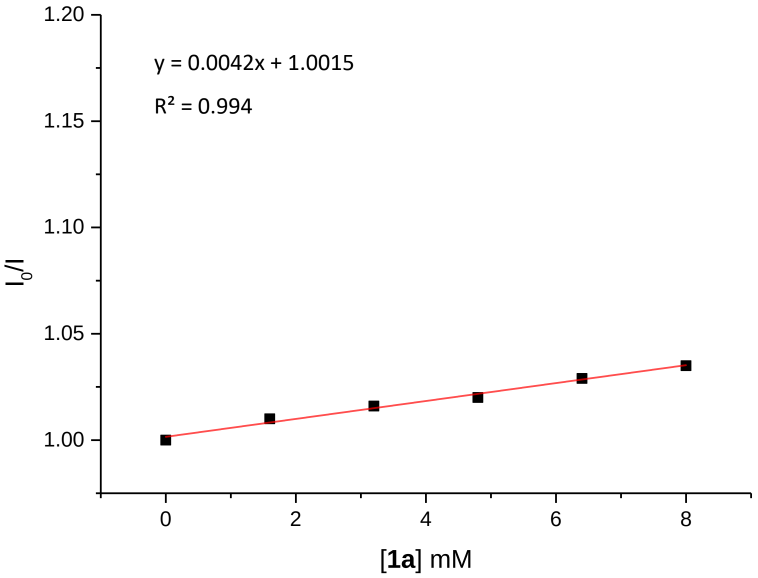

Figure S6. Stern-Volmer quenching plot using isonitrile **1a** as a quencher.

*B.1.2. Stern-Volmer quenching studies with 4-cyanopyridine **2a***

To 2.5 mL of solution A (**3DPA2FBN**), portions of 40 μL of a 0.10 M solution of 4-cyanopyridine **2a** were added. A fluorescence spectrum of the mixture was recorded after each addition. Results are shown in ***Figure S7***.

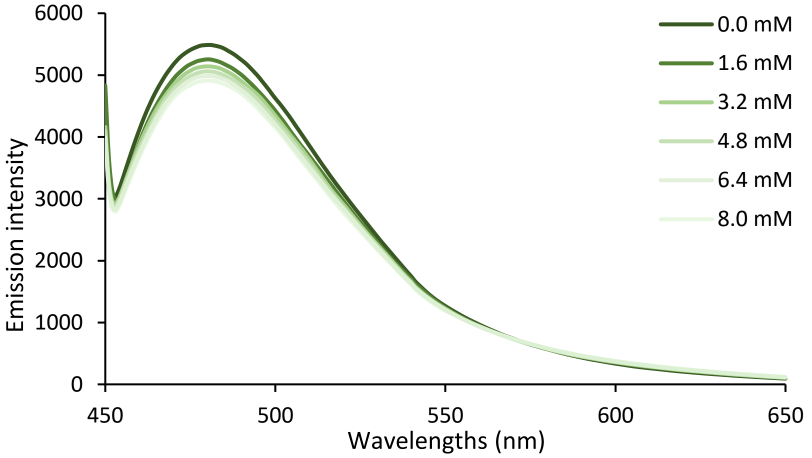

Figure S7. Quenching of the photocatalyst emission ($2.0 \cdot 10^{-4}$ M in acetone) in the presence of increasing amounts of 4-cyanopyridine **2a**.

The Stern-Volmer plot, reported in ***Figure S8***, shows a linear correlation between the relative intensity I_0/I and the concentration of **2a**. Based on the *equation S1*, it is possible to calculate the Stern-Volmer constant K_{SV}.

Therefore, the K_{SV} for reagent **2a** is 13.8 M^{-1}.

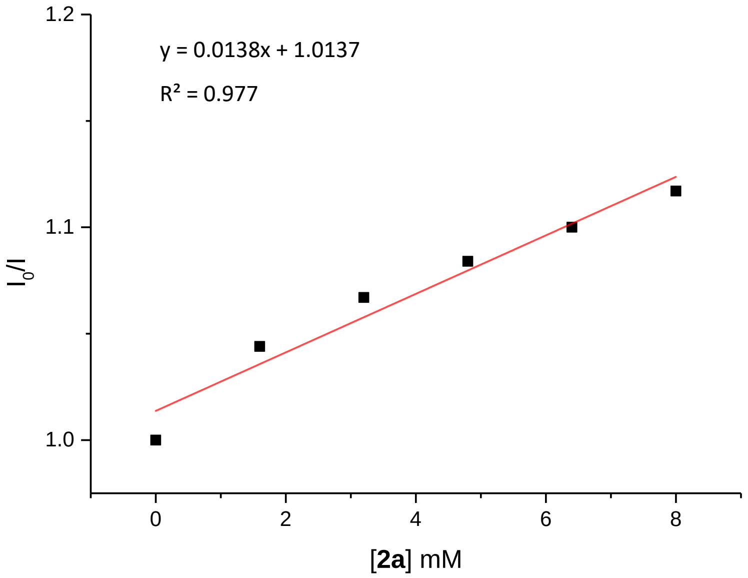

Figure S8. Stern-Volmer quenching plot using 4-cyanopyridine **2a** as a quencher.

*B.1.3. Stern-Volmer quenching studies with $TMS_3SiNHAd$ **3***

To 2.5 mL of solution A (**3DPA2FBN**), portions of 40 μL of a 0.10 M solution of $TMS_3SiNHAd$ **3** were added. A fluorescence spectrum of the mixture was recorded after each addition. Results are shown in ***Figure S9***.

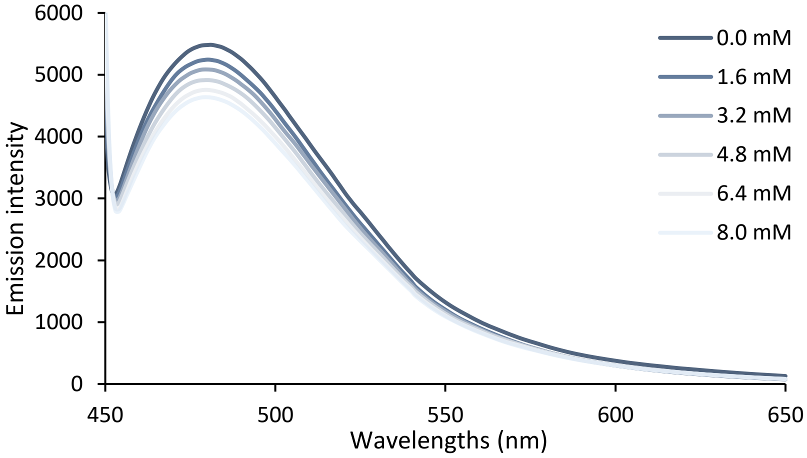

Figure S9. Quenching of the photocatalyst emission ($2.0 \cdot 10^{-4}$ M in acetone) in the presence of increasing amounts of $TMS_3SiNHAd$ **3**.

The Stern-Volmer plot, reported in ***Figure S10***, shows a linear correlation between the relative intensity I_0/I and the concentration of **3**. Based on the *equation S1*, it is possible to calculate the Stern-Volmer constant K_{SV}.

Therefore, the K_{SV} for reagent **3** is 22.8 M^{-1}.

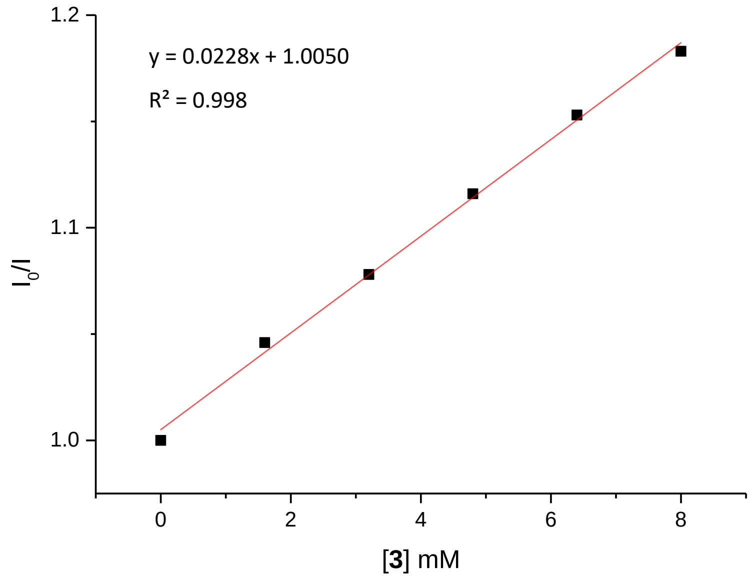

Figure S10. Stern-Volmer quenching plot using $TMS_3SiNHAd$ **3** as a quencher.

*B.1.4. Stern-Volmer quenching plot of **1a**, **2a** and **3***

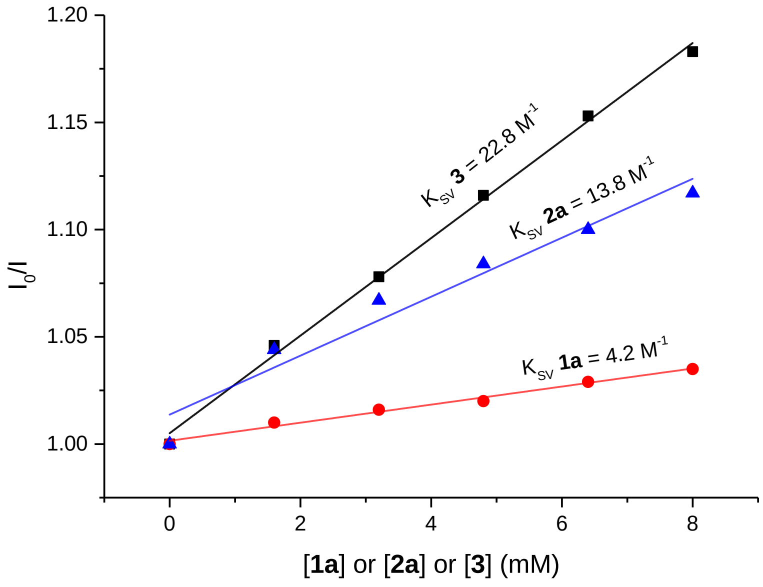

Figure S11. Stern-Volmer quenching plot using **1a**, **2a** and **3** as quenchers.

B. 2. Stern-Volmer Quenching Studies for Ir[(ppy)$_2$(dtbbpy)]PF$_6$

Stern-Volmer quenching studies were conducted on an JASCO FP-8600 Spectrofluorometer. Samples of **Ir[(ppy)$_2$(dtbbpy)]PF$_6$**, 4-cyanopyridine **2a** and $TMS_3SiNHAd$ **3**, were prepared as degassed solutions in acetone. The concentration of **Ir[(ppy)$_2$(dtbbpy)]PF$_6$** was $1.0 \cdot 10^{-5}$ M (solution B). Samples were sealed in a 1 cm quartz cuvette. The sample solution was excited at

440 nm, with emission intensity detected at 575 nm. The emission light was acquired from 500 nm to 800 nm.

*B.2.1. Stern-Volmer quenching studies with 4-cyanopyridine **2a***

To 2.5 mL of solution B (**Ir[(ppy)$_2$(dtbbpy)]PF$_6$**), portions of 10 μL of a 1.25 M solution of 4-cyanopyridine **2a** were added. A fluorescence spectrum of the mixture was recorded after each addition. Results are shown in ***Figure S12***.

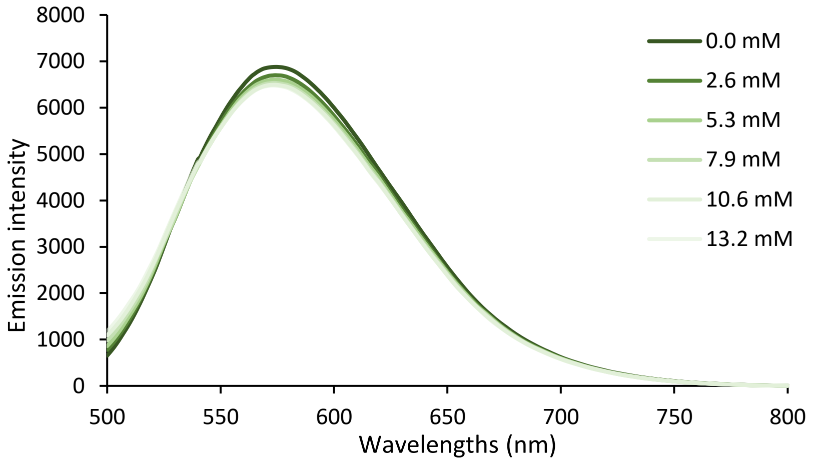

Figure S12. Quenching of the photocatalyst emission ($1.0 \cdot 10^{-5}$ M in acetone) in the presence of increasing amounts of 4-cyanopyridine **2a**.

The Stern-Volmer plot, reported in ***Figure S13***, shows a linear correlation between the relative intensity I_0/I and the concentration of **2a**. Based on the *equation S1*, it is possible to calculate the Stern-Volmer constant K_{SV}.

Therefore, the K_{SV} for reagent **2a** is 4.5 M^{-1}.

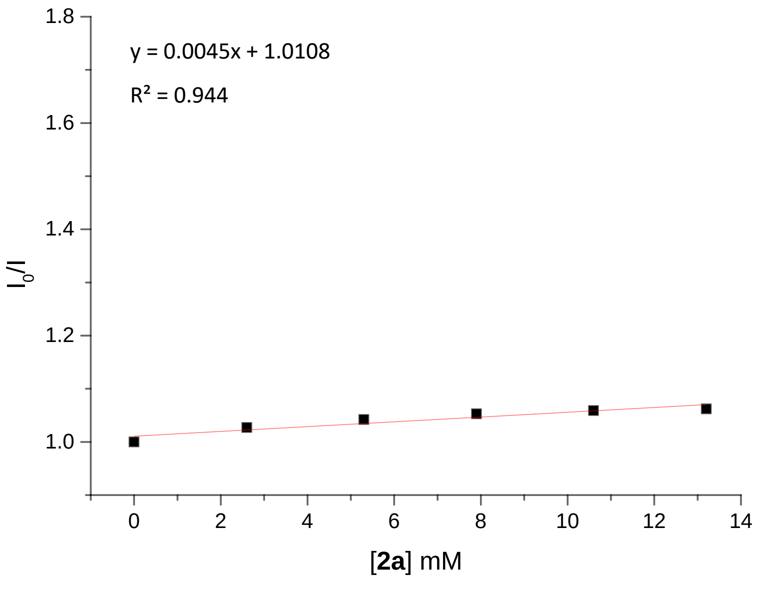

Figure S13. Stern-Volmer quenching plot using 4-cyanopyridine **2a** as a quencher.

*B.2.2. Stern-Volmer quenching studies with $TMS_3SiNHAd$ **3***

To 2.5 mL of solution B (**Ir[(ppy)$_2$(dtbbpy)]PF$_6$**), portions of 40 µL of a 0.165 M solution of $TMS_3SiNHAd$ **3** were added. A fluorescence spectrum of the mixture was recorded after each addition. Results are shown in ***Figure S14***.

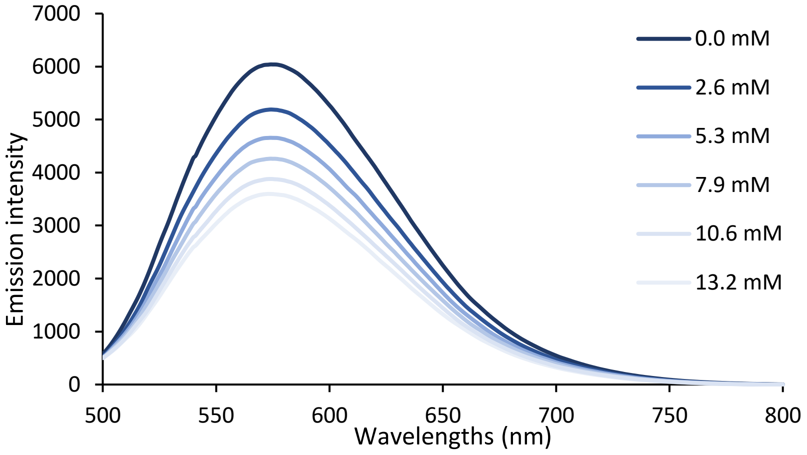

Figure S14. Quenching of the photocatalyst emission ($1.0 \cdot 10^{-5}$ M in acetone) in the presence of increasing amounts of $TMS_3SiNHAd$ **3**.

The Stern-Volmer plot, reported in ***Figure S15***, shows a linear correlation between the relative intensity I_0/I and the concentration of **3**. Based on the *equation S1*, it is possible to calculate the Stern-Volmer constant K_{SV}.

Therefore, the K_{SV} for reagent **3** is 50.7 M^{-1}.

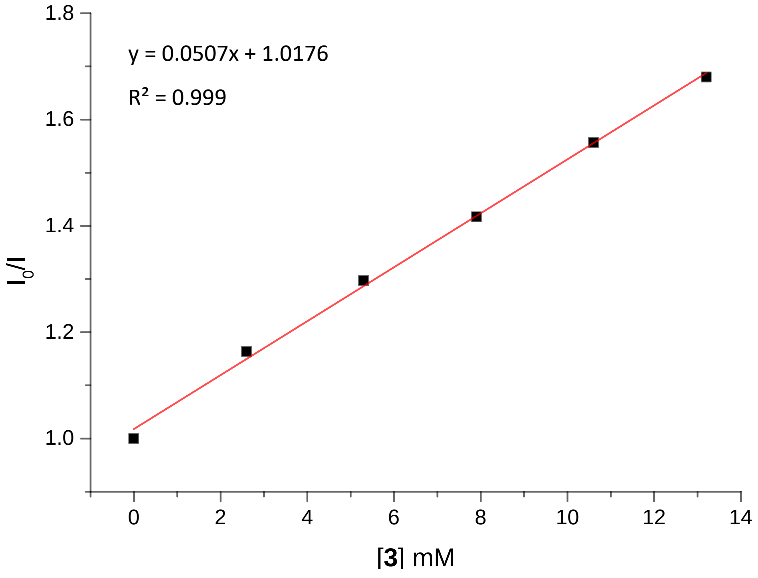

Figure S15. Stern-Volmer quenching plot using $TMS_3SiNHAd$ **3** as a quencher.

B. Proposed Mechanism for the Deaminative Arylation

Although an oxidative quenching cannot be completely ruled out, based on the better quenching of 1-adamantyl aminosilane reagent **3** compared to 4-cyanopyridine **2a** (***Figure S11***), and better matching of the redox potential, we proposed that a reductive quenching is operative for this catalytic cycle (***Figure S16***). Moreover, the Stern-Volmer experiments with Ir[(ppy)$_2$(dtbbpy)]PF_6, also able to catalyze the transformation, reinforces the reductive quenching mechanism.

Upon irradiation with visible light, the excited photocatalyst [3DPA2FBN]* is generated ($E_{1/2}^{red}$ (**PC*/PC**$^{\cdot-}$) = +0.92 V vs. SCE in CH_3CN)[58], which can oxidize the 1-adamantyl aminosilane reagent **3** (E_{pa} = +0.75 V vs. Ag/Ag^+ in 10:1 DMA/H_2O)[59] to furnish the reduced catalyst [3DPA2FBN]$^{\cdot-}$ and the N-centered radical **I**[60] (see *section 5. B.* for Stern-Volmer quenching studies). The succeeding radical aza-Brook rearrangement would provide the α-amino silicon-centered radical **II** whose addition to isonitrile **1** triggers the generation of an imidoyl radical intermediate **III**. A subsequent β–scission liberates the alkyl radical **IV**.[61] On the other hand, the resultant [3DPA2FBN]$^{\cdot-}$ is a powerful reductant ($E_{1/2}^{red}$ (**PC/PC***$^{\cdot-}$) = –1.92 V vs. SCE in CH_3CN)[58] capable of undergoing a SET reduction event with 4-cyanopyridine **2a** ($E_{1/2}^{red}$ = –1.75 V vs. SCE in CH_3CN)[62], generating pyridyl radical anion species **V**, as well as regenerating the ground-state organic photocatalyst and thereby completing the photoredox cycle. Finally, a radical-radical coupling reaction between **IV** and **V**, followed by elimination of CN^- from **VI** would then form the desired product **4**.

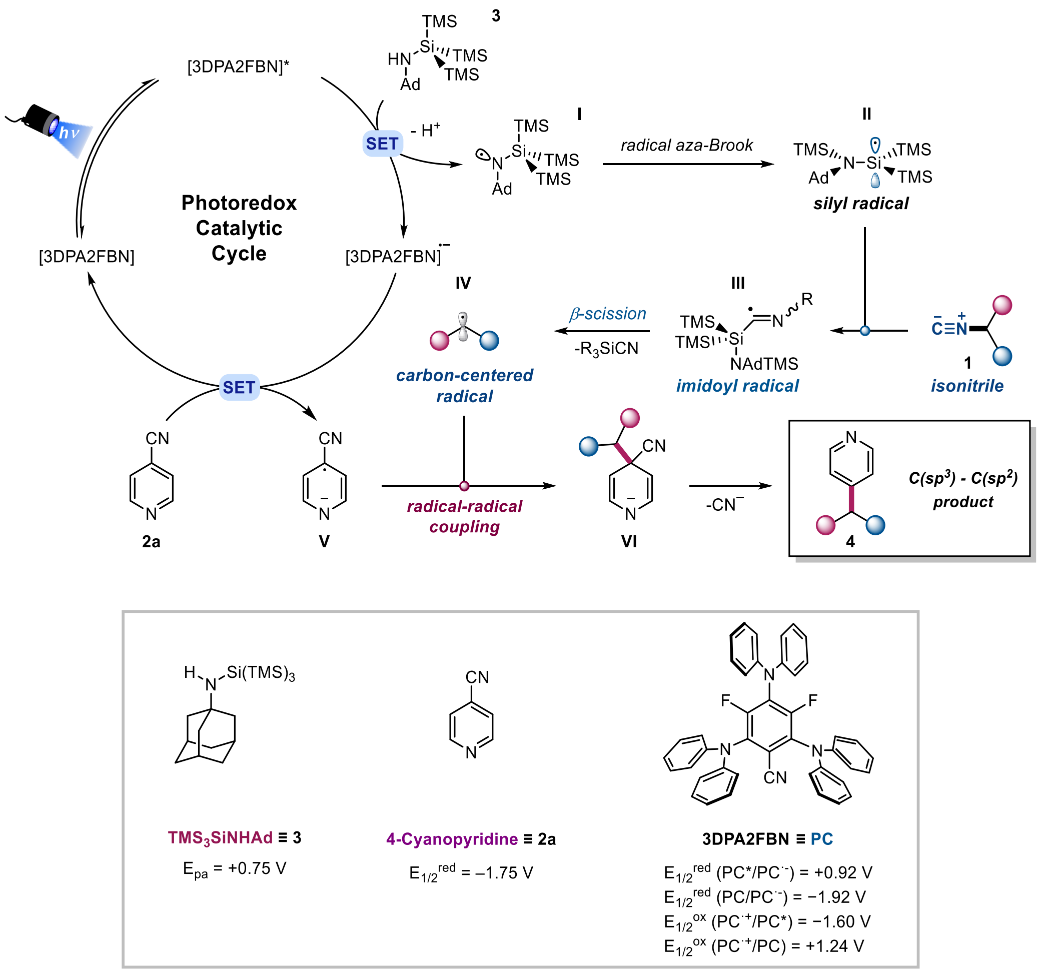

Figure S16. Plausible mechanism for the deaminative arylation.

C. Quantum Yield Measurement

The quantum yield of the reaction was determined using the procedure reported previously.[63] The quantum yield of a reaction is defined as:

$$\phi = \frac{mol\ of\ product\ formed}{F \cdot t \cdot [1 - 10^{-A(\lambda)}]} \qquad \textit{(equation S2)}$$

where ϕ is the quantum yield of the reaction, t is the time of the reaction(s), $[1\text{-}10^{-A(\lambda)}]$ is the ratio of absorbed photons by the solution and F is the photon flux calculated by standard ferrioxalate actinometry.

A ferrioxalate actinometry solution was prepared by following the Hammond variation of the Hatchard and Parker procedure outlined in Handbook of Photochemistry.[64] This method is based on the decomposition of ferric ions to ferrous ions, which are complexed by 1,10-phenanthroline and monitored by UV/Vis absorbance at 510 nm. The moles of iron-phenanthroline complex formed are related to moles of photons absorbed.

Experimental: The following solutions were prepared in the dark (flasks were wrapped in aluminum foil) and stored in the dark at r.t.:

- *Potassium ferrioxalate solution:* 294.8 mg of potassium ferrioxalate and 139 µL of sulfuric acid (96 %) were added to a 50 mL volumetric flask and filled with water (MilliQ grade).
- *Phenanthroline solution:* 0.2 % by weight of 1,10-phenanthroline in water was prepared in a 50 mL volumetric flask.
- *Buffer solution:* 2.47 g of NaOAc and 0.5 mL of sulfuric acid (96 %) were added to a 50 mL volumetric flask and filled with water (MilliQ grade).
- *Reaction solution:* Following the **GP-F**, isonitrile **1a** (37.8 mg, 0.20 mmol), isonicotinonitrile **2a** (31.2 mg, 0.30 mmol), $TMS_3SiNHAd$ **3** (103.5 mg, 0.26 mmol), 3DPA2FBN **PC** (6.4 mg, 10.0 µmol) in acetone (2.0 mL) were used.

Procedure:

1. A 4 mL glass vial was charged with 1 mL of the potassium ferrioxalate solution and placed 5 cm away from the 440 nm Kessil lamp. To monitor the decomposition reaction, this step was performed five different times varying only the irradiation time for each sample: 0, 2, 4, 6 and 10 seconds. All were irradiated at 25% intensity of the 440 nm LED Kessil lamp.
2. After irradiation, all the actinometer solution was transferred to a 10 mL volumetric flask. To this flask, 0.5 mL of the phenanthroline solution and 2 mL of buffer solution were added and filled with water (MilliQ grade).
3. The reaction was stirred in the dark for 1 hour to allow the ferrous ions to coordinate completely to the phenanthroline.
4. The UV-Vis spectra of actinometry samples were recorded for each time interval (***Figure S17***). The absorbance of the actinometry solution was monitored at 510 nm.

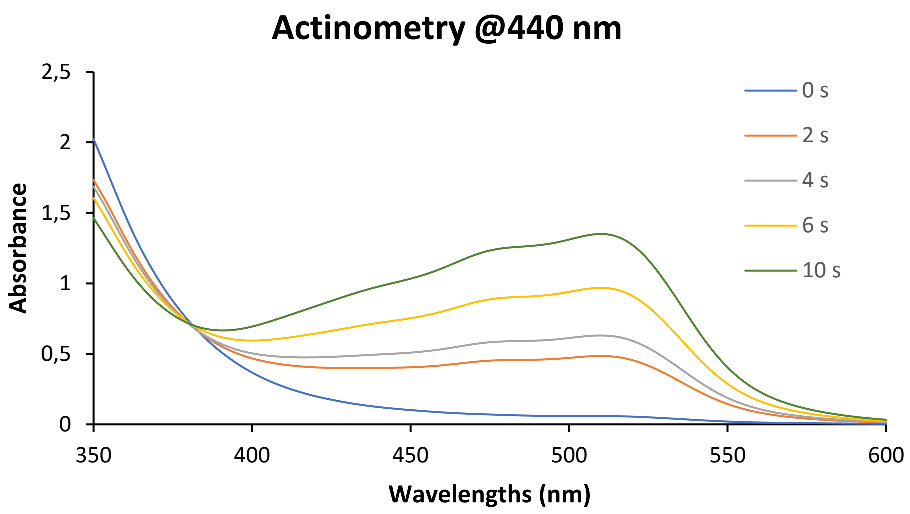

Figure S17: UV-Vis spectra of actinometry samples irradiated with 440 nm Kessil during different time periods.

5. The moles of Fe^{2+} formed (N) is determined using Lambert-Beer's Law (*equation S3*).

$$mmol\ Fe^{2+} = \frac{V_1 \cdot V_3 \cdot \Delta A_{(510\ nm)}}{10^3 \cdot V_2 \cdot l \cdot \varepsilon_{(510\ nm)}} \qquad \textit{(equation S3)}$$

where V_1 is the irradiated volume (1 mL), V_2 is the aliquot of the irradiated solution taken for the determination of the ferrous ions (1 mL), V_3 is the final volume after complexation with phenanthroline (10 mL), l is the optical path-length of the irradiation cell (1 cm), $\Delta A_{(510\ nm)}$ the optical difference in absorbance between the irradiated solution and the one taken in the dark, $\varepsilon_{(510\ nm)}$ is the molar extinction coefficient of the complex $Fe(phen)_3^{2+}$ (11100 L mol^{-1} cm^{-1}).

6. The moles of Fe^{2+} formed (N) are plotted as a function of time (t). The slope of this line (dN/dt) was correlated to the moles of incident photons by unit of time (F), according to the following *equation S4*.

$$\Phi Fe^{2+} = \frac{dN/dt}{F \cdot [1 - 10^{-A(\lambda)}]} \qquad \textit{(equation S4)}$$

where dN/dt is the rate of change of this property, the quantum yield ΦFe^{2+} at 456 nm is 0.9,[64] $[1 - 10^{-A(\lambda)}]$ is the ratio of absorbed photons by the solution, and A(λ) is the absorbance of the ferrioxalate solution at the wavelength used to carry out the experiments (440 nm). The absorbance of this solution at 440 nm is 0.059. Therefore, F, which is the photon flux, was determined to be $9.88 \cdot 10^{-7}$.

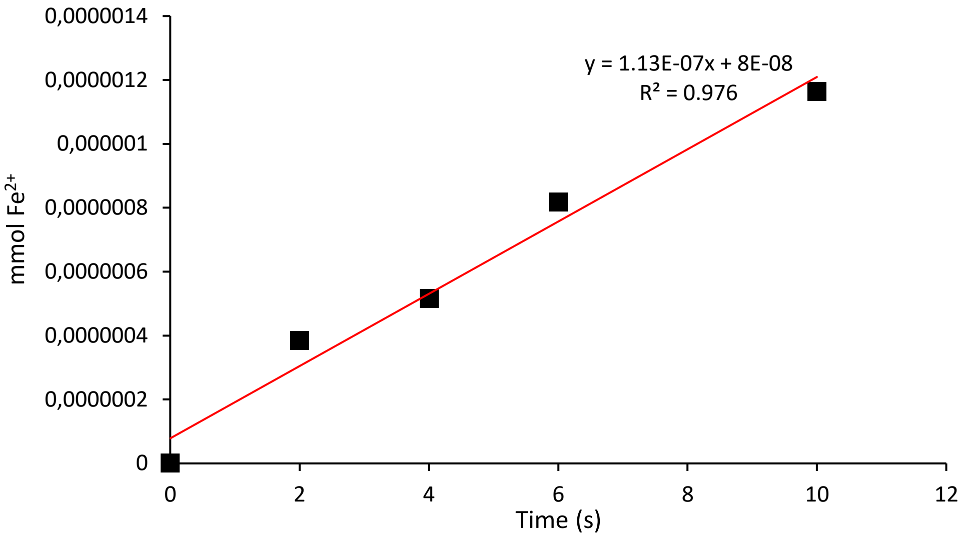

***Figure S18*:** Moles of Fe^{2+} formed after irradiation with 440 nm Kessil as a function of time.

7. The reaction solution was irradiated using the same system described above for the actinometry experiment. The moles of product formed were determined by ^{1}H-NMR analysis using 1,3,5-trimethoxybenzene as internal standard at different intervals of time. The moles of product per unit of time are related to the number of photons absorbed by the use of *equation S2*.

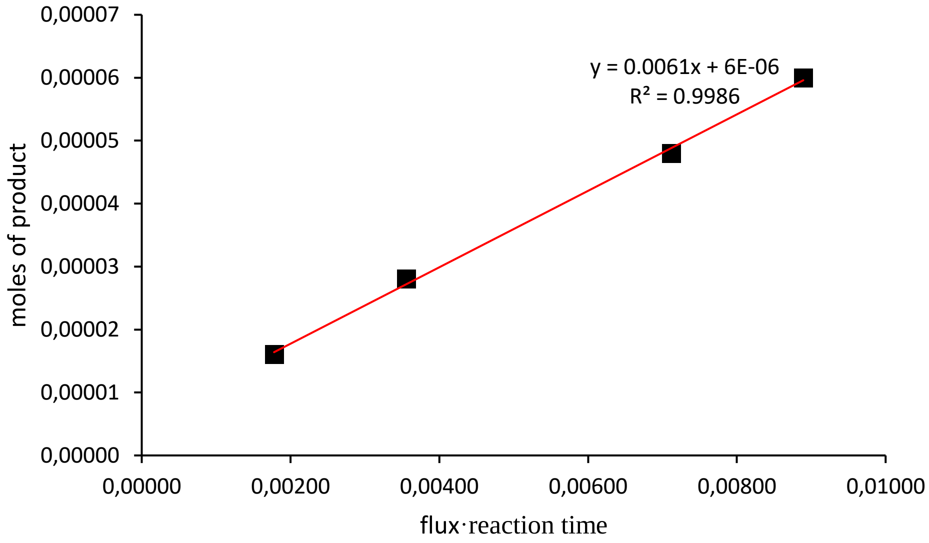

***Figure S19*:** Moles of product **4a** formed related to the moles of incident photons.

In agreement with this, if we plot (***Figure S19***) the moles of product formed (**4a**) vs. the moles of incident photons (F · t), the slope is equal to: $\Phi\cdot[1\text{-}10^{-A(440\text{ nm})}]$, where Φ is the quantum yield to determine and A(440 nm) is the absorption of the reaction under study. An absorbance of 1.26 was determined for the model reaction mixture. **The quantum yield (Φ) of the photochemical transformation was measured to be 0.01.**

D. TEMPO Radical-Trapping Experiment

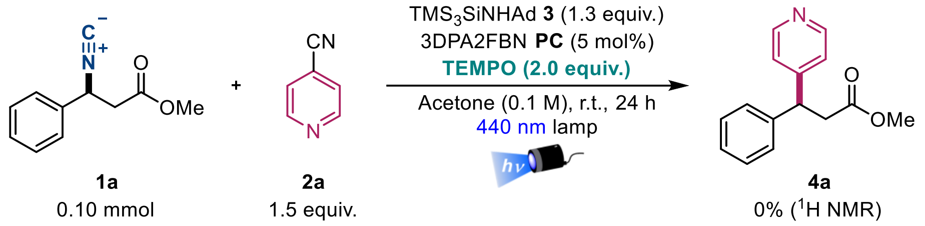

According to the **GP-F**, isonitrile **1a** (18.9 mg, 0.10 mmol, 1.0 equiv.), isonicotinonitrile **2a** (15.6 mg, 0.15 mmol, 1.5 equiv.), TMS$_3$SiNHAd **3** (51.7 mg, 0.13 mmol, 1.3 equiv.), 3DPA2FBN **PC** (3.2 mg, 5.0 µmol, 0.05 equiv.) and 2,2,6,6-tetramethylpiperidine1-1-oxyl (**TEMPO**, 31.3 mg, 0.20 mmol, 2.0 equiv.) in acetone (1.0 mL) were used. After 24 h, all the volatiles were removed under reduced pressure, and the crude mixture was analyzed by ^{1}H NMR using 1,3,5-trimethoxybenzene as internal standard. ^{1}H NMR analysis of the resulting crude showed no conversion to the arylated product and full recovery of the unreacted starting material.

E. Detection of R$_3$SiCN

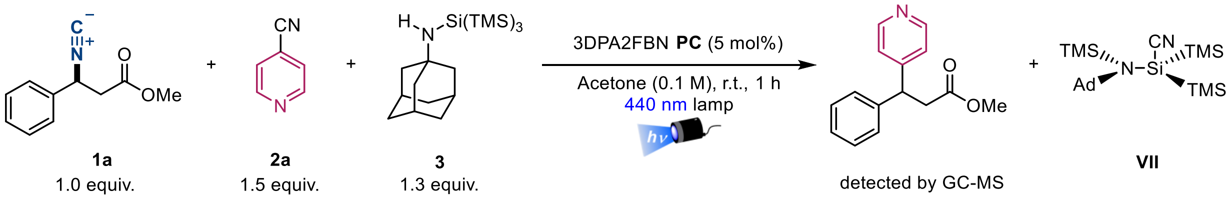

We thoroughly tried to detect the silylamine **VII** through GC-MS. However, our attempts were unsuccessful probably due to the instability of the silylamine **VII**. We could however identify the fragment **VIII** that could be formed through homolytic cleavage of **VII** under the conditions used in the detection method (***Scheme S2***). Moreover, we were able to identify fragment **IX** through an extracted-ion chromatogram.

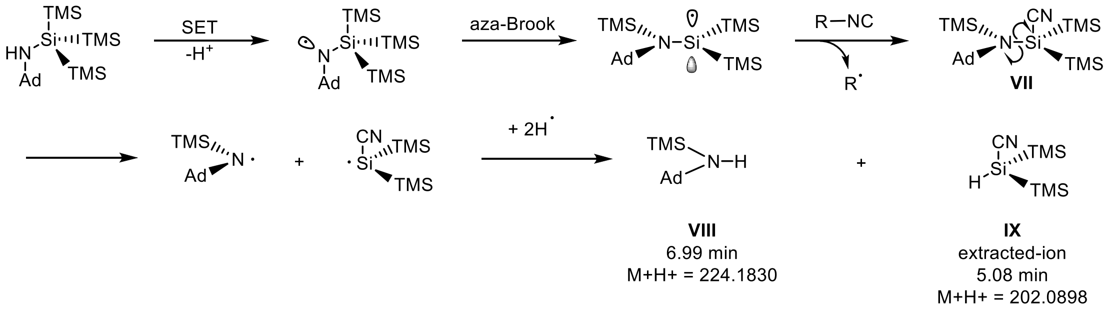

Scheme S2. Possible fragmentation of silylamine **VII**.

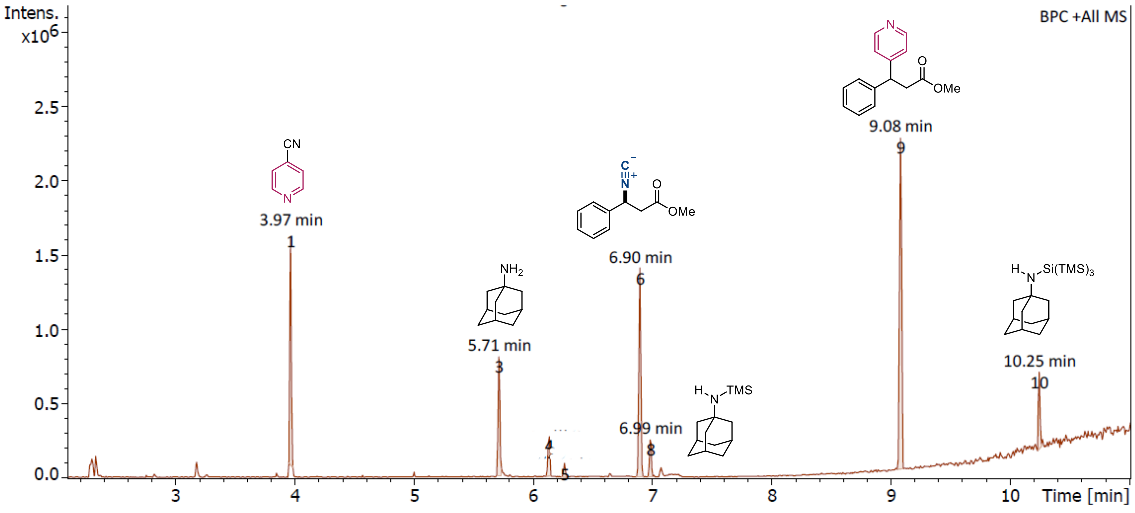

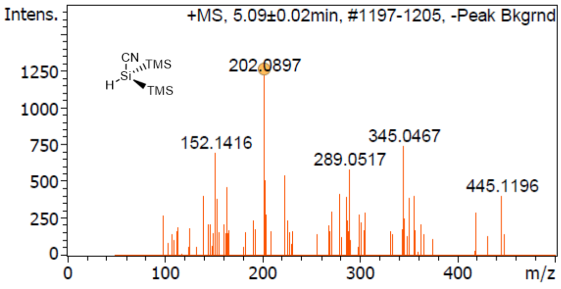

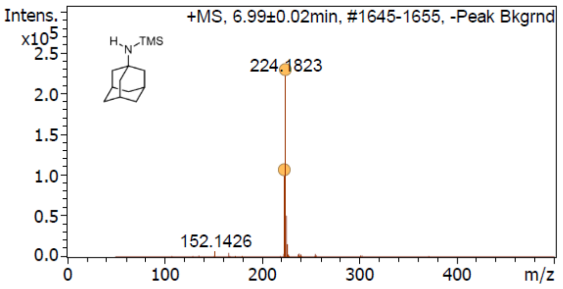

We then tried to identify silane **X** formed when using supersilane as silyl radical precursor.

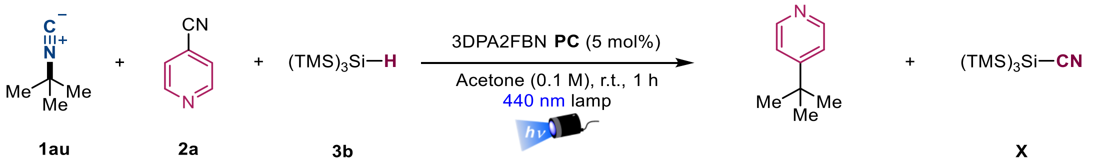

1au 1.0 equiv. | **2a** 1.5 equiv. | **3b** 1.3 equiv. | detected by GC-MS | **X** detected by GC-MS

Irradiating with blue 440 nm Kessil PR160L LED lamp a mixture of *tert*-butyl isocyanide **1au**, isonicotinonitrile **2a,** supersilane **3b** and 3DPA2FBN **PC** in acetone, we were pleased to detect the corresponding $(TMS)_3SiCN$ **X** by GC-MS.

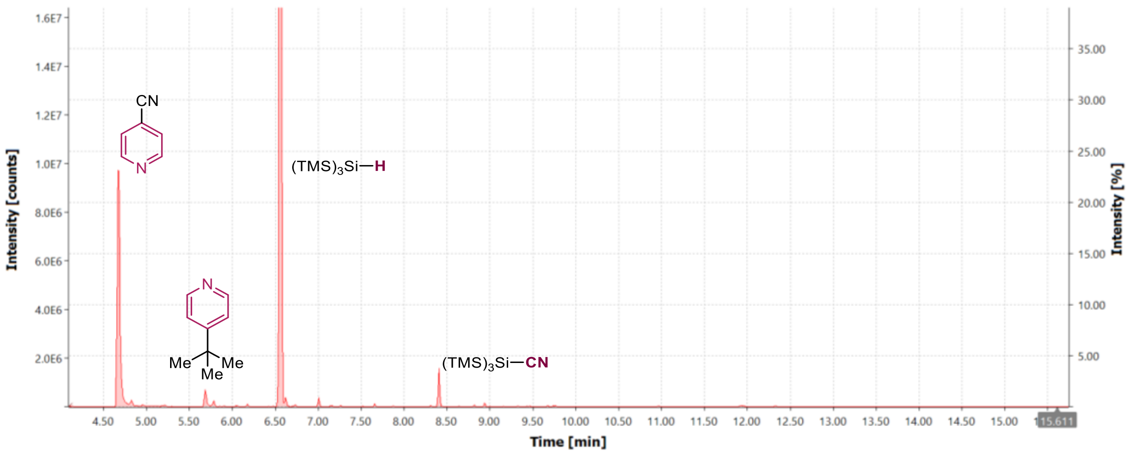

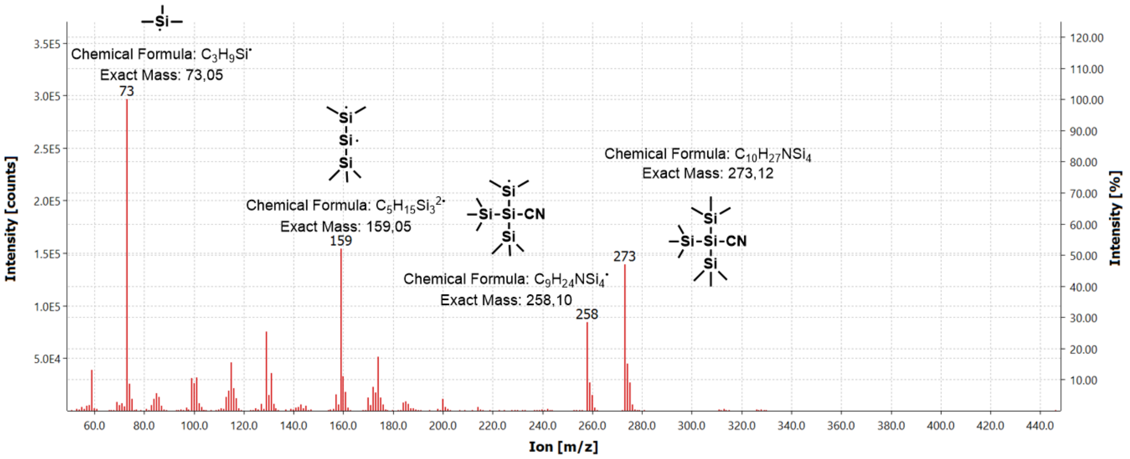
Chemical Formula: C3H9Si•
Exact Mass: 73,05
73
Chemical Formula: C5H15Si3 2•
Exact Mass: 159,05
159
Chemical Formula: C9H24NSi4•
Exact Mass: 258,10
258
Chemical Formula: C10H27NSi4
Exact Mass: 273,12
273
Si
Si-Si-CN
Intensity [counts]
Intensity [%]
Ion [m/z]

8. References

[1] Quirós, I.; Martín, M.; Gomez-Mendoza, M.; Cabrera-Afonso, M. J.; Liras, M.; Fernández, I.; Nóvoa, L.; Tortosa, M. Isonitriles as Alkyl Radical Precursors in Visible Light Mediated Hydro- and Deuterodeamination Reactions. *Angew. Chem. Int. Ed.* **2024**, *63*, e202317683.
[2] Kunig, V. B. K.; Potowski, M.; Akbarzadeh, M.; Arendt, L.; Dormuth, I.; Zhang, R.; Groves, M.; Ottmann, C.; Fried, R.; Dömling, A.; Brunschweiger, A. TEAD-YAP Interaction Inhibitors and MDM2 Binders from DNA-Encoded Indole-Focused Ugi Peptidomimetics. *Angew. Chem. Int. Ed.* **2020**, *59*, 20338-20342.
[3] Galli, U.; Tron, G. C.; Purghè, B.; Grosa, G.; Aprile, S. Metabolic Fate of the Isocyanide Moiety: Are Isocyanides Pharmacophore Groups Neglected by Medicinal Chemists?. *Chem. Res. Toxicol.* **2020**, *33*, 955-966.
[4] Fang, H-P.; Fu, C-C.; Tai, C-K.; Chang, K-H.; Yang, R-H.; Wu, M-J.; Chen, H-C.; Li, C-J.; Huang, S-Q.; Lien, W-H.; Chen, C-H.; Hsieh, C-H.; Wang, B-C.; Cheung, S-F.; Pan, P-S. Synthesis and stability study of isocyano aryl boronate esters and their synthetic applications. *RSC. Adv.* **2016**, *6*, 30362-30371.
[5] Si, Y-X.; Zhu, P-F.; Zhang, S-L. Synthesis of Isocyanides by Reacting Primary Amines with Difluorocarbene. *Org. Lett.* **2020**, *22*, 9086-9090.
[6] Li, Y.; Chao, A.; Fleming, F. F. Isonitrile alkylations: a rapid route to imidazo[1,5-a]pyridines. *Chem. Commun.* **2016**, *52*, 2111-2113.
[7] Neochoritis, C. G.; Stotani, S.; Mishra, B.; Dömling, A. Efficient Isocyanide-less Isocyanide-Based Multicomponent Reactions. *Org. Lett.* **2015**, *17*, 2002-2005.
[8] Ricardo, M. G.; Ali, A. M.; Plewka, J.; Surmiak. E.; Labuzek. B.; Neochoritis, C. G.; Atmaj, J.; Skalniak, L.; Zhang, R.; Holak, T. A.; Groves, M.; Rivera, D. G.; Dömling, A. Multicomponent Peptide Stapling as a Diversity-Driven Tool for the Development of Inhibitors of Protein-Protein Interactions. *Angew. Chem. Int. Ed.* **2020**, *59*, 5235-5241.
[9] Neochoritis, C. G.; Dömling, A. Towards a facile and convenient synthesis of highly functionalized indole derivatives based on multi-component reactions. *Org. Biomol. Chem.* **2014**, *12*, 1649-1651.
[10] Jakas, A.; Višnjevac, A.; Jerić, I. Multicomponent Approach to Homo- and Hetero- Multivalent Glycomimetics Bearing Rare Monosaccharides. *J. Org. Chem.* **2020**, *85*, 3766-3787.
[11] Bietti, M.; Fiorentini, S.; Pérez Pato, I.; Salamone, M. Oxygen Acidity of Ring Methoxylated 1,1-Diarylalkanol Radical Cations Bearing α-Cyclopropyl Groups. The Competition between *O*-Neophyl Shift and C−Cyclopropyl β-Scission in the Intermediate 1,1-Diarylalkoxyl Radicals. *J. Org. Chem.* **2006**, *71*, 3167-3175.
[12] Schäfer, G.; Matthey, C.; Bode, J. W. Facile Synthesis of Sterically Hindered and Electron-Deficient Secondary Amides from Isocyanates. *Angew. Chem. Int. Ed.* **2012**, *51*, 1–4.
[13] Pramanick, P. K.; Zhou, Z.; Hou, Z.; Ao, Y.; Yao, B. Native amine-directed site-selective C(sp^3)-H arylation of primary aliphatic amines with aryl iodides. *Chin. Chem. Lett.* **2020**, *31*, 1327–1331.
[14] Colyer, J. T.; Andersen, N. G.; Tedrow, J. S.; Soukup, T. S.; Faul, M. M. Reversal of Diastereofacial Selectivity in Hydride Reductions of N-*tert*-Butanesulfinyl Imines. *J. Org. Chem.* **2006**, *71*, 6859–6862.
[15] Rueping, M.; Vila, C.; Uria, U. Direct Catalytic Azidation of Allylic Alcohols. *Org. Lett.* **2012**, *14*, 768–771.
[16] Misiura, K.; Kardacka, K.; Kusnierczyk, H. Synthesis, in vitro metabolic studies, and antitumour activity of methyl analogues of ifosfamide. *Arch. Pharm. Pharm. Med. Chem.* **2001**, *334*, 291–294.
[17] Kulagowki, J. J.; Blair, W.; Bull, R.; Chang, C.; Deshmukh, G.; Dyke, H.; Eigenbrot, C.; Ghilardi, N.; Gibbons, P.; Harrison, T.; Hewitt, P.; Liimatta, M.; Hurley, C.; Johnson, A.; Johnson, T.; Kenny, J.; Kohli, P.; Maxey, R.; Mendonca, R.; Mortara, K.; Murray, J.; Narukulla, R.; Shia, S.; Steffek, M.; Ubhayakar, S.; Ultsch, M.; van Abbema, A.; Ward, S.; Waszkowycz, B.; Zak, M. Identification of Imidazo-Pyrrolopyridines as Novel and Potent JAK1 Inhibitors. *J. Med. Chem.* **2012**, *55*, 5901–5921.
[18] Si, Y.X.; Zhu, P.F.; Zhang, S.L. Synthesis of Isocyanides by Reacting Primary Amines with Difluorocarbene. *Org. Lett.* **2020**, *22*, 9086–9090.

[19] Trulli, L.; Sciubba, F.; Fioravanti, S. Chiral *trans*-Carboxylic Trifluoromethyl 2-Imidazolines by a Ag_2O-Catalyzed Mannich-type Reaction. *Tetrahedron*, **2018**, *74*, 572-577.
[20] Neochoritis, C. G.; Stotani, S.; Mishra, B.; Dömling, A. Efficient Isocyanide-less Isocyanide-Based Multicomponent Reactions. *Org. Lett.* **2015***, 17*, 2002 – 2005.
[21] Reckzeh, E.; Karageorgis, G.; Schwalfenberg, M.; Ceballos, J.; Nowacki, J.; Stroet, M.; Binici, A.; Knauer, L.; Brand, S.; Choidas, A.; Strohmann, C.; Ziegler, S.; Waldmann, H. Inhibition of Glucose Transporters and Glutaminase Synergistically Impairs Tumor Cell Growth. *Cell. Chem. Biology* **2019**, *26*, 1214–1228.
[22] Kobayashi, K.; Matsumoto, N.; Matsumoto, K. Synthesis of 3,4-Dihydroisoquinolines by Cyclization of 1-Bromo-2-(2-isocyanoalkyl)benzenes with Butyllithium. *Heterocycles*, **2013**, *87*, 389 – 397.
[23] Leech, M. C.; Petti, A.; Tanbouza, N.; Mastrodonato, A.; Goodall, I. C. A.; Ollevier, T.; Dobbs, A. P.; Lam, K. Anodic Oxidation of Aminotetrazoles: A Mild and Safe Route to Isocyanides. *Org. Lett.* **2021***, 23*, 9371 – 9375.
[24] Patil, P.; Ahmadian-Moghaddam, M.; Dömling, A. Isocyanide 2.0. *Green Chem.* **2020***, 22*, 6902 – 6911.
[25] Leech, M. C.; Petti, A.; Tanbouza, N.; Mastrodonato, A.; Goodall, I. C. A.; Ollevier, T.; Dobbs, A. P.; Lam, K. Anodic Oxidation of Aminotetrazoles: A Mild and Safe Route to Isocyanides. *Org. Lett.* **2021**, *23*, 9371–9375.
[26] Adachi, M.; Miyasaka, T.; Hashimoto, H.; Nishikawa, T. One-Step Transformation of Trichloroacetamide into Isonitrile. *Org. Lett*. **2017**, *19*, 380 – 383.
[27] Chennakrishnareddy, G.; Nagendra, G.; Hemantha, H. P.; Das, U.; Guru Row, T. N.; Sureshbabu, V. V. Isoselenocyanates derived from Boc/Z-amino acids: synthesis, isolation, characterization, and application to the efficient synthesis of unsymmetrical selenoureas and selenoureidopeptidomimetics. *Tetrahedron* **2010**, *66*, 6718 – 6724.
[28] Reidl, T. W.; Bandar, J. S. Lewis Basic Salt-Promoted Organosilane Coupling Reactions with Aromatic Electrophiles. *J. Am. Chem. Soc.* **2021**, *143*, 11939-11945.
[29] Lounsbury, N.; Mateo, G.; Jones, B.; Papaiahgari, S.; Thimmulappa, R. K.; Teijaro, C.; Gordon, J.; Korzekwa, K.; Ye, M.; Allaway, G.; Abou-Gharbia, M.; Biswal, S.; Childers, W. Heterocyclic chalcone activators of nuclear factor (erythroid-derived 2)-like 2 (Nrf2) with improved in vivo efficacy. *Bioorg. Med. Chem.* **2015**, *23*, 5352-5359.
[30] Vaid, T. P.; Cook, M. E.; Scott, J. D.; Carazo, M. B.; Ruchti, J.; Minteer, S. D.; Sigman, M. S.; McNeil, A. J.; Sanford, M. S. Theoretical and Experimental Investigation of Functionalized Cyanopyridines Yield an Anolyte with and Extremely Low Reduction Potential for Nonaqueous Redox Flow Batteries. *Chem. Eur. J.* **2022**, *28*, e202202147.
[31] Zhang, X.; McNally, A. Phosphonium Salts as Pseudohalides: Regioselective Nickel-Catalyzed Cross-Coupling of Complex Pyridines and Diazines. *Angew. Chem. Int. Ed.* **2017**, *56*, 9833–9836.
[32] Greenwood, J. W.; Boyle, B. T.; McNally, A. Pyridylphosphonium salts as alternatives to cyanopyridines in radical-radical coupling reactions. *Chem. Sci.* **2021**, *12*, 10538–10543.
[33] Vaid, T. P.; Cook, M. E.; Scott, J. D.; Carazo, M. B.; Ruchti, J.; Minteer, S. D.; Sigman, M. S.; McNeil, A. J.; Sanford, M. S. Theoretical and Experimental Investigation of Functionalized Cyanopyridines Yield an Anolyte with an Extremely Low Reduction Potential for Nonaqueous Redox Flow Batteries. *Chem. Eur. J.* **2022**, *28*, e202202147.
[34] Becker, M. R.; Knochel, P. Practical Continuous-Flow Trapping Metalations of Functionalized Arenes and Heteroarenes Using TMPLi in the Presence of Mg, Zn, Cu or La Halides. *Angew. Chem. Int. Ed.* **2015**, *54*, 12501 – 12505.
[35] Cailly, T.; Fabis, F.; Rault, S. A new, direct, and efficient synthesis of benzonaphthyridin-5-ones. *Tetrahedron* **2006**, *62*, 5862 – 5867.
[36] Speckmeir, E.; Fischer, T. G.; Zitler, K. A Toolbox Approach To Construct Broadly Applicable Metal-Free Catalysts for Photoredox Chemistry: Deliberate Tuning of Redox Potentials and Importance of Halogens in Donor-Acceptor Cyanoarenes. *J. Am. Chem. Soc*. **2018**, *140*, 15353 – 15365.
[37] Sakai, H. A.; Liu, W.; Le, C.; MacMillan, D. W. C. Cross-Electrophile Coupling of Unactivated Alkyl Chlorides. *J. Am. Chem. Soc*. **2020**, *142*, 11691 – 11697.
[38] Zhang, S.; Li, L.; Li, X.; Zhang, J.; Xu, K.; Li, G.; Findlater, M. Electroreductive 4-Pyridylation of Electron-deficient Alkenes with Assistance of $Ni(acac)_2$. *Org. Lett*. **2020**, *22*, 3570 – 3575.

[39] Wang, Y.; Li, R.; Guan, W.; Li, Y.; Li, X.; Yin, J.; Zhang, G.; Zhang, Q.; Xiong, T.; Zhang, Q. Organoborohydride-catalyzed Chichibabin-type C4-position alkylation of pyridines with alkenes assisted by organoboranes. *Chem. Sci.*, **2020**, *11*, 11554 – 11561.
[40] Gao, L.; Wang, G.; Cao, J.; Chen, H.; Gu, Y.; Liu, X.; Cheng, X.; Ma, J.; Li, S. Lewis Acid-Catalyzed Selective Reductive Decarboxylative Pyridylation of N-Hydroxyphthalimide Esters: Synthesis of Congested Pyridine-Substituted Quaternary Carbons. *ACS Catal.* **2019**, *9*, 10142 – 10151.
[41] Chen, B.; Cao, P.; Yin, X.; Liao, Y.; Jiang, L.; Ye, J.; Wang, M.; Liao, J. Modular Synthesis of Enantioenriched 1,1,2-Triarylethanes by an Enantioselective Arylboration and Cross-Coupling Sequence. *ACS Catal.* **2017**, *7*, 2425 – 2429.
[42] Lima, F.; Kabeshov, M. A.; Tran, D. N.; Battilocchio, C.; Sedelmeier, J.; Sedelmeir, G.; Schenkel, B.; Ley, S. V. Visible Light Activation of Boronic Esters Enables Efficient Photoredox $C(sp^2)$–$C(sp^3)$ Cross-Couplings in Flow. *Angew. Chem.* **2016**, *128*, 14291 – 14295.
[43] Wu, J.; Wang, D.; Chen, X.; Gui, Q.; Li, H.; Tan, Z.; Huang, G.; Wang, G. Synthesis of 4-benzylpyridines via Pd-catalyzed CH3-arylation of 4-picoline. *Org. Biomol. Chem.*, **2017**, *15*, 7509 – 7512.
[44] Lipp, B.; Lipp, A.; Detert, H.; Opatz, T. Light-Induced Alkylation of (Hetero)aromatic Nitriles in a Transition-Metal-Free C–C-Bond Metathesis. *Org. Lett.* **2017**, *19*, 2054 – 2057.
[45] Kolusu, S. R. N.; Nappi, M. Metal-free deoxygenative coupling of alcohol-derived benzoates and pyridines for small molecules and DNA-encoded libraries synthesis. *Chem. Sci.*, **2022**, *13*, 6982 – 6989.
[46] Matsumoto, K.; Kannami, M.; Inokuchi, D.; Kurata, H.; Kawase, T.; Oda, M. Tetrakis(4-pyridyl)methane: Synthesis, Properties, and a Diamondoid Network Structure of Its Silver(I) Complex. *Org. Lett.* **2007**, *9*, 2903 – 2906.
[47] Nakano, Y.; Black, M. J.; Meichan, A. J.; Sandoval, B. A.; Chung, M. M.; Biagasiewicz, K. F.; Zhu T.; Hyster, T. K. Photoenzymatic Hydrogenation of Heteroaromatic Olefins Using 'Ene'-Reductases with Photoredox Catalysts. *Angew. Chem. Int. Ed.* **2020**, *59*, 10484 – 10488.
[48] Sidhoum, M. A.; Dos Santos, A.; El Kaïm, L.; Legras, L. Pyrrolidinone and Piperidinone Isocyanides from Isocyano Esters. *Eur. J. Org. Chem.* **2014**, 4949 – 4952.
[49] Zhang, Z.; He, Q.; Zhang, X; Yang, C. Photoredox-catalysed regioselective synthesis of C-4-alkylated pyridines with N-(acyloxy)phthalimides. *Org. Biomol. Chem.,* **2022**, *20*, 1969 – 1973.
[50] Barton, D. H.; Halley, F.; Ozbalik, N.; Schmitt, M.; Young, E.; Balavoine, G. W. Functionalization of saturated hydrocarbons. 14. Further studies on the mechanism of Gif-type systems. *J. Am. Chem. Soc.* **1989**, *111*, 7144 – 7149.
[51] Lin, Q.; Gong, H.; Wu, F. Ni-Catalyzed Reductive Coupling of Heteroaryl Bromides with Tertiary Alkyl Halides. *Org. Lett.* **2022**, *24*, 8996-9000
[52] Katritzky, A. R.; Zhang, S.; Kurz, T.; Wang, M; Steel, P. J. Regiospecific Synthesis of 4-(2-Oxoalkyl)pyridines. *Org. Lett.* **2001**, *3*, 2807 – 2809.
[53] Wang, H.; Wu, J.; Noble, A.; Aggarwal, V. K. Selective Coupling of 1,2-Bis-Boronic Esters at the more Substituted Site through Visible-Light Activation of Electron Donor-Acceptor Complexes. *Angew. Chem. Int. Ed.* **2022**, *61*, e202202061.
[54] Odriozola, A.; Oiarbide, M.; Palomo, C. Enantioselective Synthesis of Quaternary Δ^4- and Δ^5-Dehydroprolines Based on a Two-Step Formal [3+2] Cycloaddition of α-Aryl and α-Alkyl Isocyano(thio)acetates with Vinyl Ketones. *Chem. Eur. J.* **2017**, *23*, 12758–12762.
[55] Chu, L.; Ohta, C.; Zuo, Z.; MacMillan, D. W. C. Carboxylic Acids as A Traceless Activation Group for Conjugate Additions: A Three-Step Synthesis of (±)-Pregabalin. *J. Am. Chem. Soc.* **2014**, *136*, 10886–10889.
[56] Luo, M.-P.; Gu, Y.-J.; Wang, S.-G. Photocatalytic enantioselective Minisci reaction of β-carbolines and application to natural product synthesis. *Chem. Sci.*, **2023**, *14*, 251–256.
[57] Mu, M.-C.; Shang, R.; Zhao, B.; Wang, B.; Fu, Y. Photocatalytic decarboxylative alkylations mediated by triphenylphosphine and sodium iodide. *Science*, **2019**, *363*, 1429–1434.
[58] Speckmeir, E.; Fischer, T. G.; Zitler, K. A Toolbox Approach To Construct Broadly Applicable Metal-Free Catalysts for Photoredox Chemistry: Deliberate Tuning of Redox Potentials and Importance of Halogens in Donor-Acceptor Cyanoarenes. *J. Am. Chem. Soc.* **2018**, *140*, 15353 – 15365.

[59] Carson II, W. P.; Sarver, P. J.; Goudy, N. S.; MacMillan, D. W. C. Photoredox Catalysis-Enabled Sulfination of Alcohols and Bromides. *J. Am. Chem. Soc*. **2023**, *145*, 20767 – 20774.
[60] For the related radical aza-Brook rearrangement with silyl migration from carbon to nitrogen, see: (a) Harris, J. M.; MacInnes, I.; Walton, J. C.; Maillard, B. 1,2-Migration of the trimethylsilyl group in free radicals. *J. Organomet. Chem.* **1991**, *3*, C25−C28. (b) Harris, J. M.; Walton, J. C.; Maillard, B.; Grelier, S.; Picard, J.-P. Hydrogen abstraction from silylamines; an investigation of the 1,2-migration of the trimethylsilyl group in aminyl radicals. *J. Chem. Soc., Perkin Trans. 2* **1993**, *11*, 2119−2123. (c) Schiesser, C. H.; Styles, M. L. On the radical Brook and related reactions: an ab initio study of some (1,2)- silyl, germyl and stannyl translocations. *J. Chem. Soc., Perkin Trans. 2* **1997**, 2355−2340.
[61] (a) Barton, D. H. R.; Bringmann, G.; Lamotte, G.; Motherwell, W. B.; Motherwell, R. S. H.; Porter, A. E. A. *J. Chem. Soc., Perkin Trans. 1,* **1980**, 2657. (b) Barton, D. H. R.; Bringmann, G.; Lamotte, G.; Motherwell, R. S. H.; Motherwell, W. B. *Tetrahedron Lett.* **1979**, *24*, 2291-2294. (c) Saegusa, T.; Kobayashi, S.; Ito, Y.; Yasuda, N. *J. Am. Chem. Soc.* **1968**, *90*, 4182. (d) Ballestri, M.; Chatgilialoglu, C. *J. Org. Chem.* **1991**, *56*, 678 – 683.
[62] Andrieux, C. P.; Gelis, L.; Medebielle, M.; Pinson, J.; Saveant, J. M. Outer-sphere dissociative electron transfer to organic molecules: a source of radicals or carbanions? Direct and indirect electrochemistry of perfluoroalkyl bromides and iodides. *J. Am. Chem. Soc.* **1990**, *112*, 3509–3520.
[63] a) Cuadros, S.; Rosso, C.; Barison, G.; Costa, P.; Kurbasi, M.; Bonchio, M.; Prato, M.; Filippini, G.; Dell'Amico, L. The Photochemical Activity of a Halogen-Bonded Complex Enables the Microfluidic Light-Driven Alkylation of Phenols. *Org. Lett.* **2022**, *16*, 2961 – 2966. b) Georgiou, E.; Spinnato, D.; Chen, K.; Melchiorre, P.; Muñiz, K. Switchable photocatalysis for the chemodivergent benzylation of 4-cyanopyridines. *Chem. Sci.* **2022**, *13*, 8060 – 8064. c) Piedra, H. F.; Plaza, M. Photochemical halogen-bonding assisted generation of vinyl and sulfur-centered radicals: stereoselective catalyst-free C(sp^2)-S bond forming reactions. *Chem. Sci.* **2023**, *14*, 650 – 657.
[64] Murov, S. L.; *Handbook of Photochemistry*, Marcel Dekker, New York, **1973**.

9. NMR spectra

For known compounds, only ^{1}H NMR spectra is provided. For new compounds, full characterization is provided.

^{1}H-NMR (300 MHz, $CDCl_3$) of compound **SI-3**

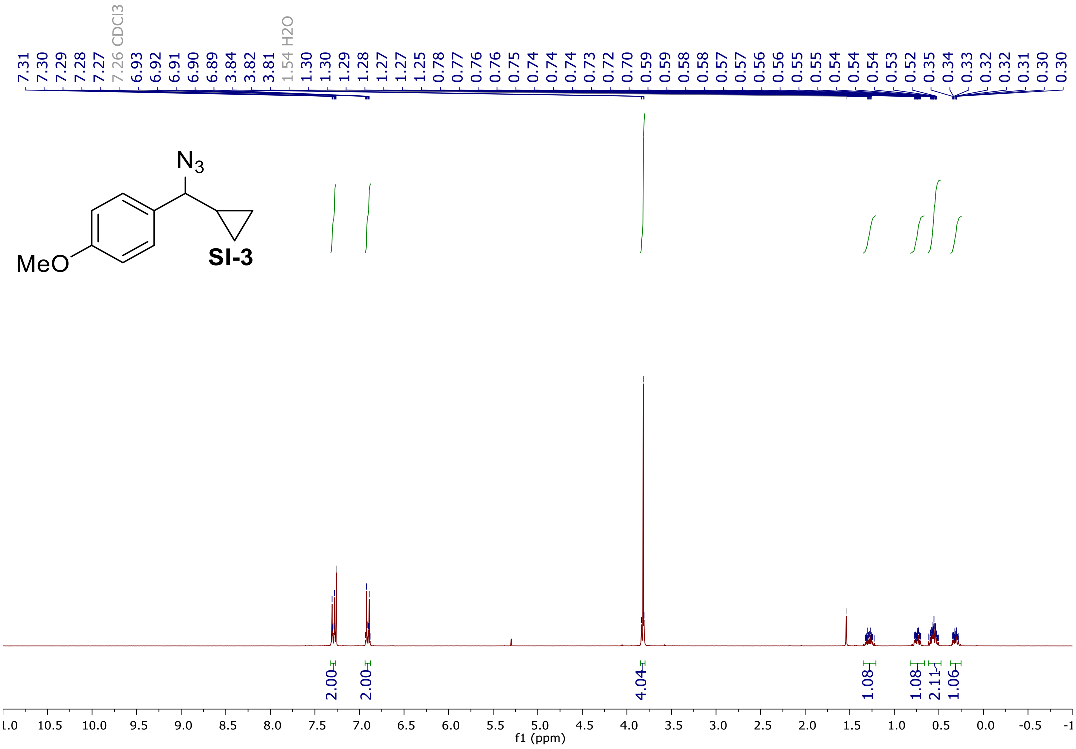

^{13}C-NMR (75 MHz, $CDCl_3$) of compound **SI-3**

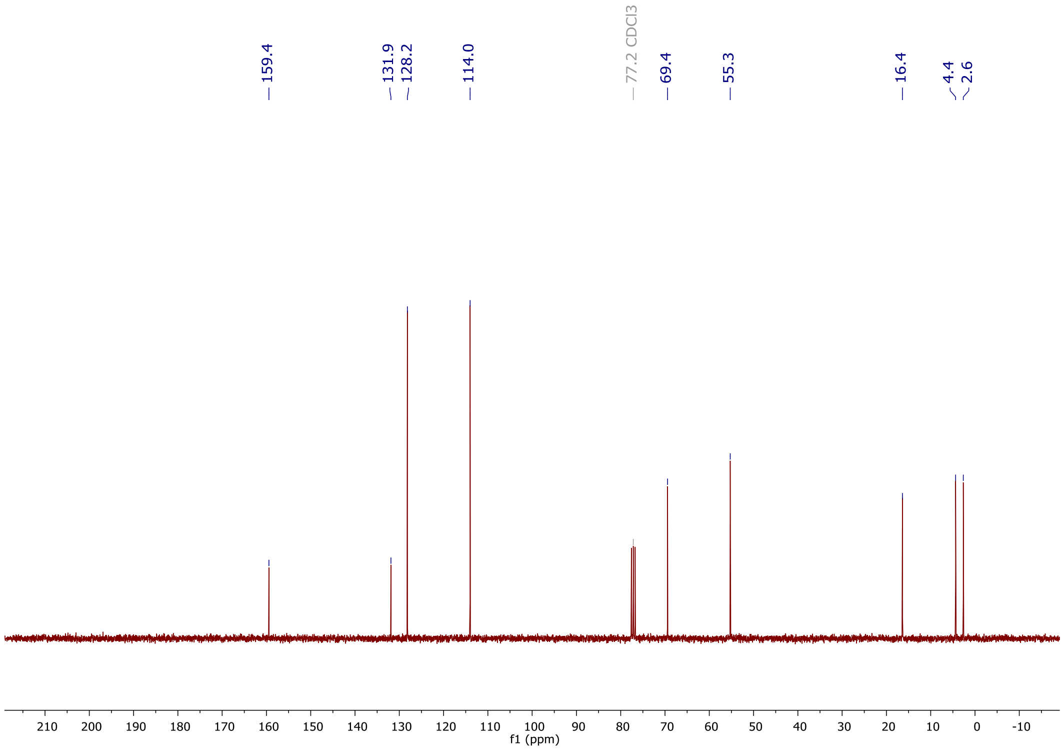

^{1}H-NMR (300 MHz, CDCl3) of compound **SI-4**

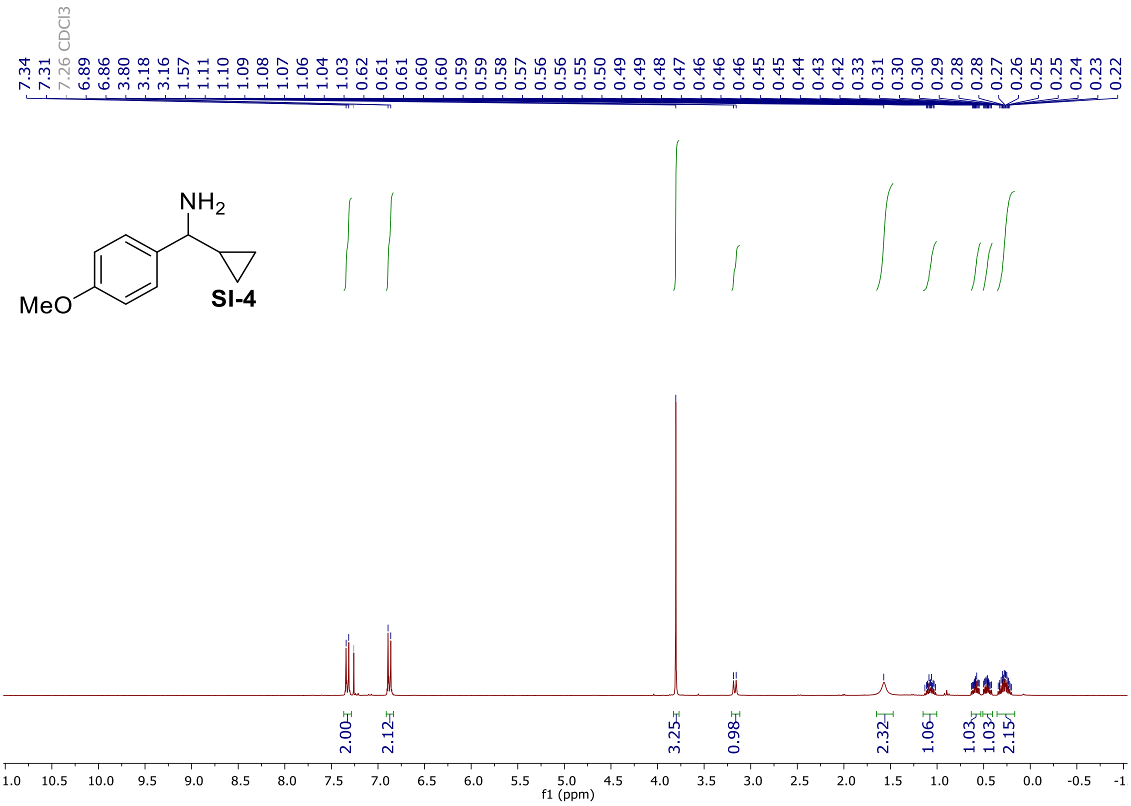

^{13}C-NMR (75 MHz, CDCl3) of compound **SI-4**

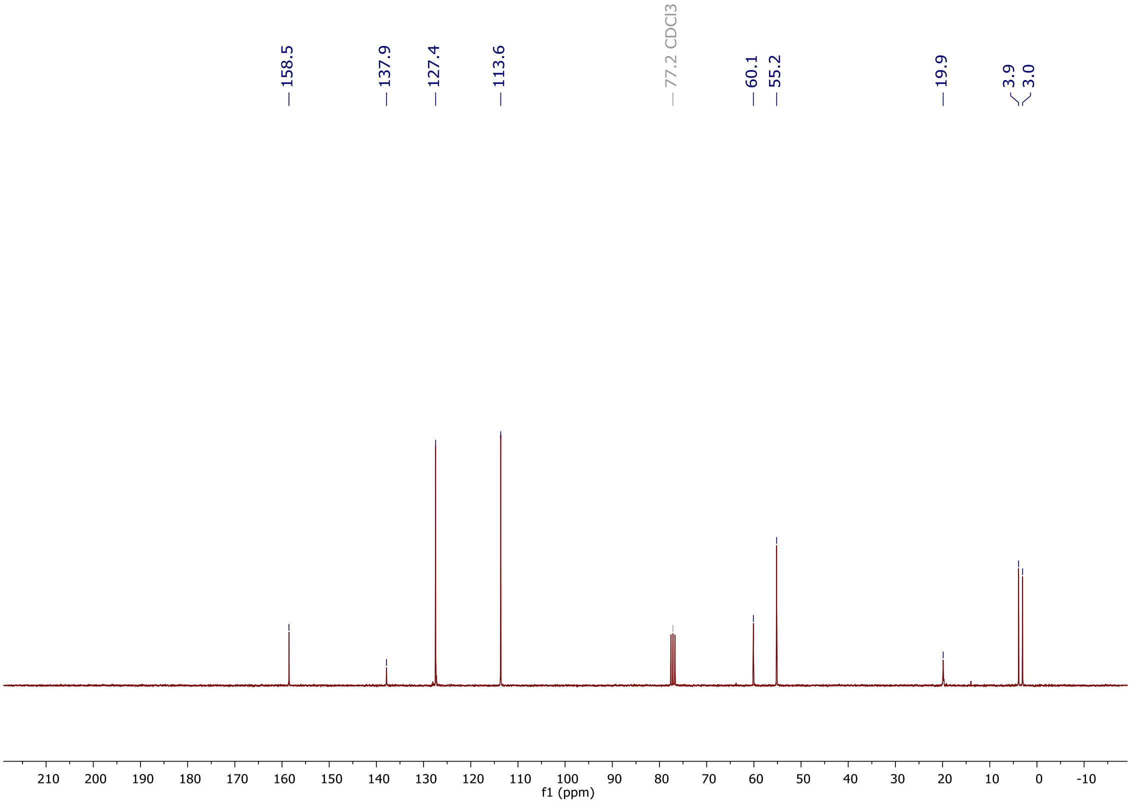

¹H-NMR (300 MHz, $CDCl_3$) of compound **SI-16**

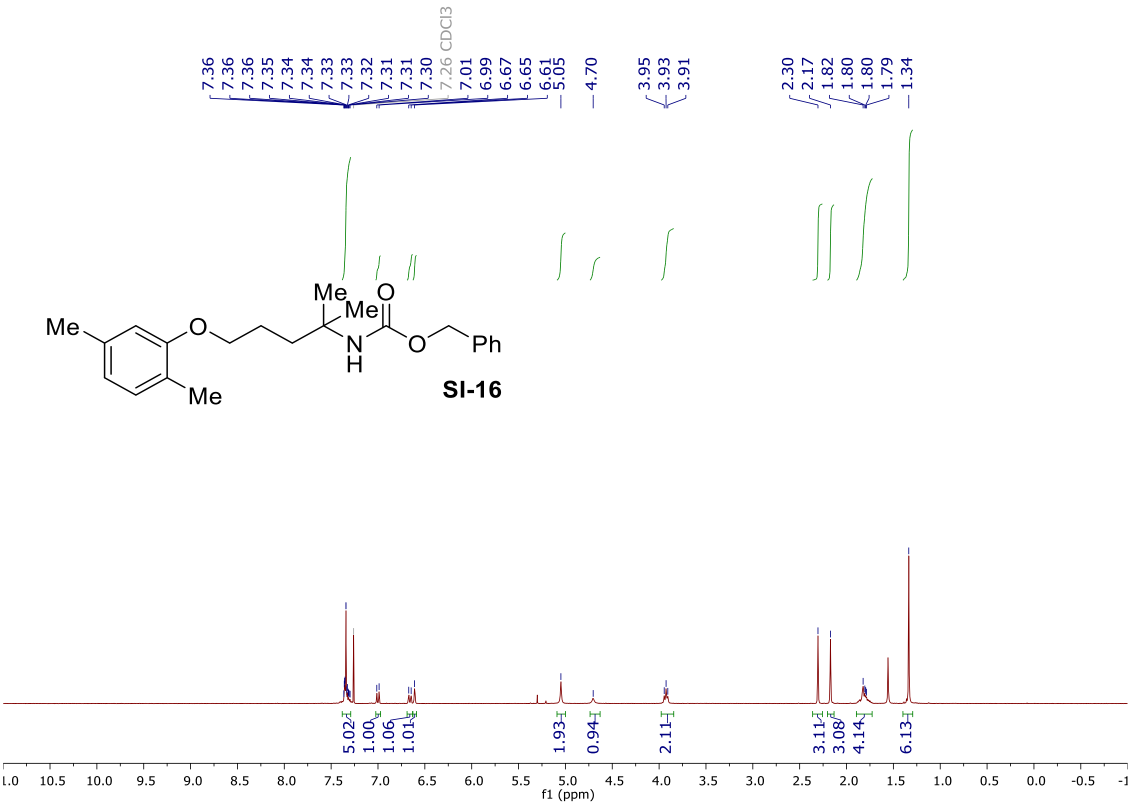

¹³C-NMR (75 MHz, $CDCl_3$) of compound **SI-16**

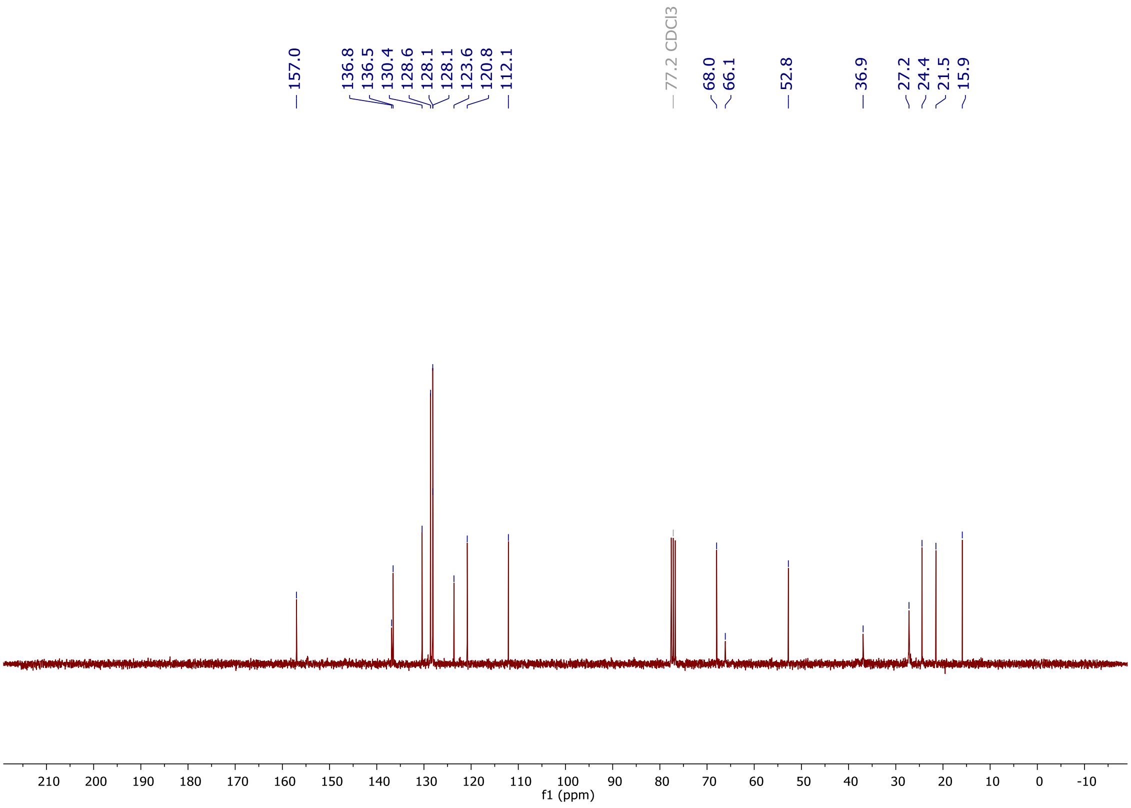

^{1}H-NMR (300 MHz, $CDCl_3$) of compound **SI-17**

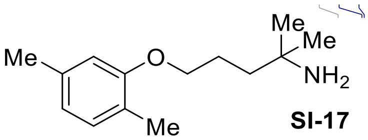

SI-17

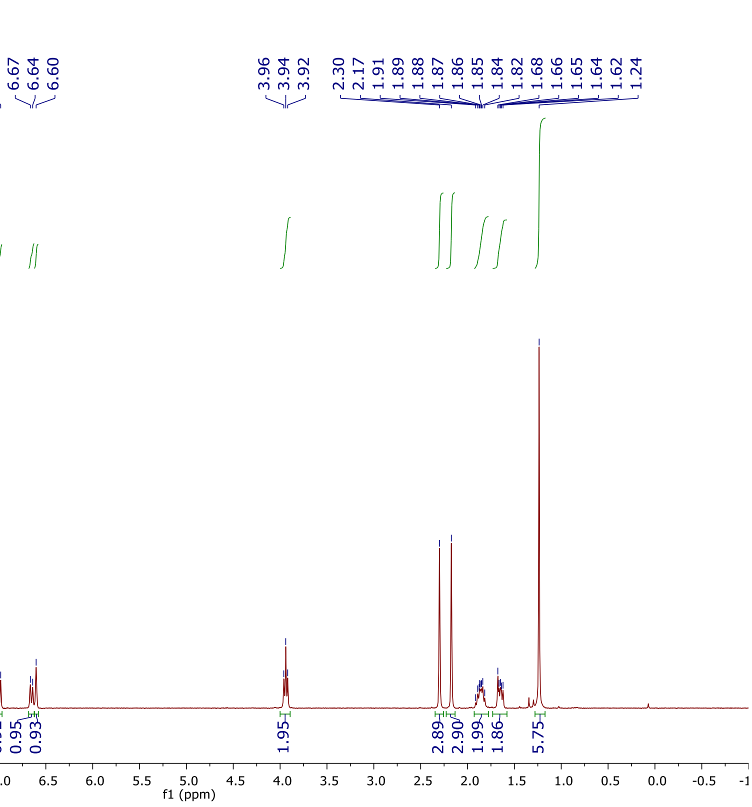

^{13}C-NMR (75 MHz, $CDCl_3$) of compound **SI-17**

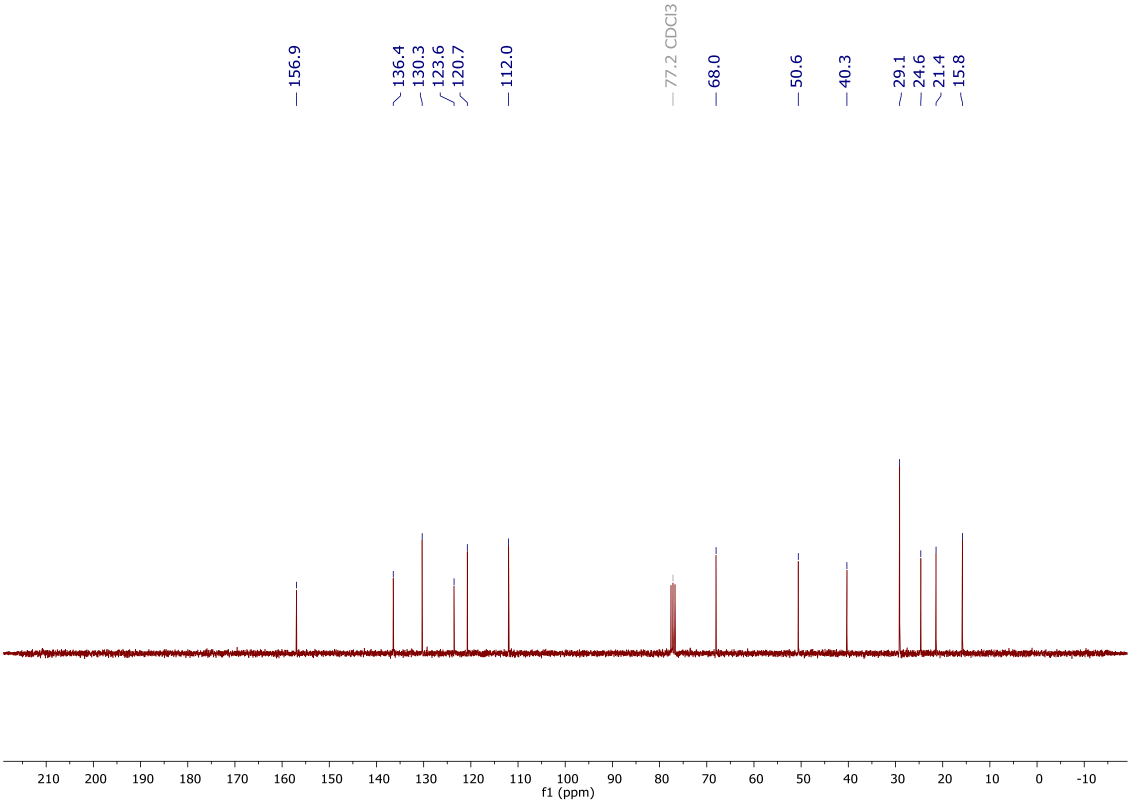

^{1}H-NMR (300 MHz, $CDCl_3$) of compound **1d**

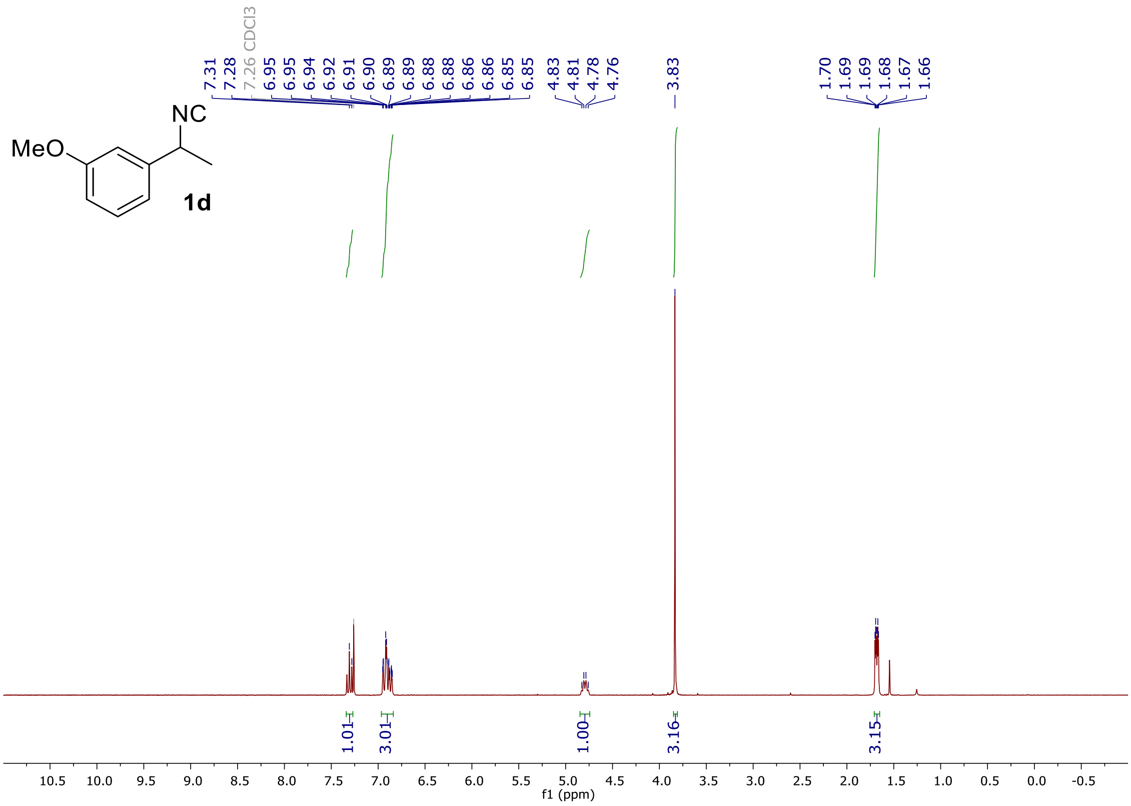

^{1}H-NMR (300 MHz, $CDCl_3$) of compound **1e**

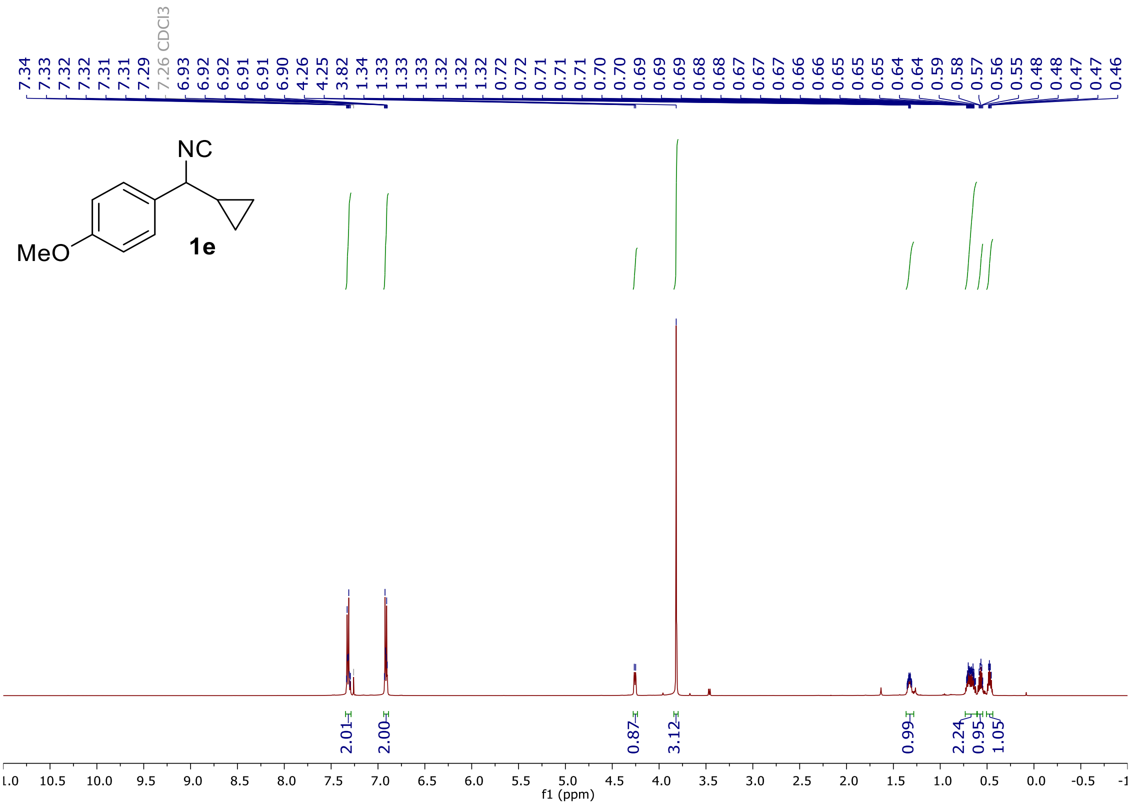

^{13}C-NMR (75 MHz, $CDCl_3$) of compound **1e**

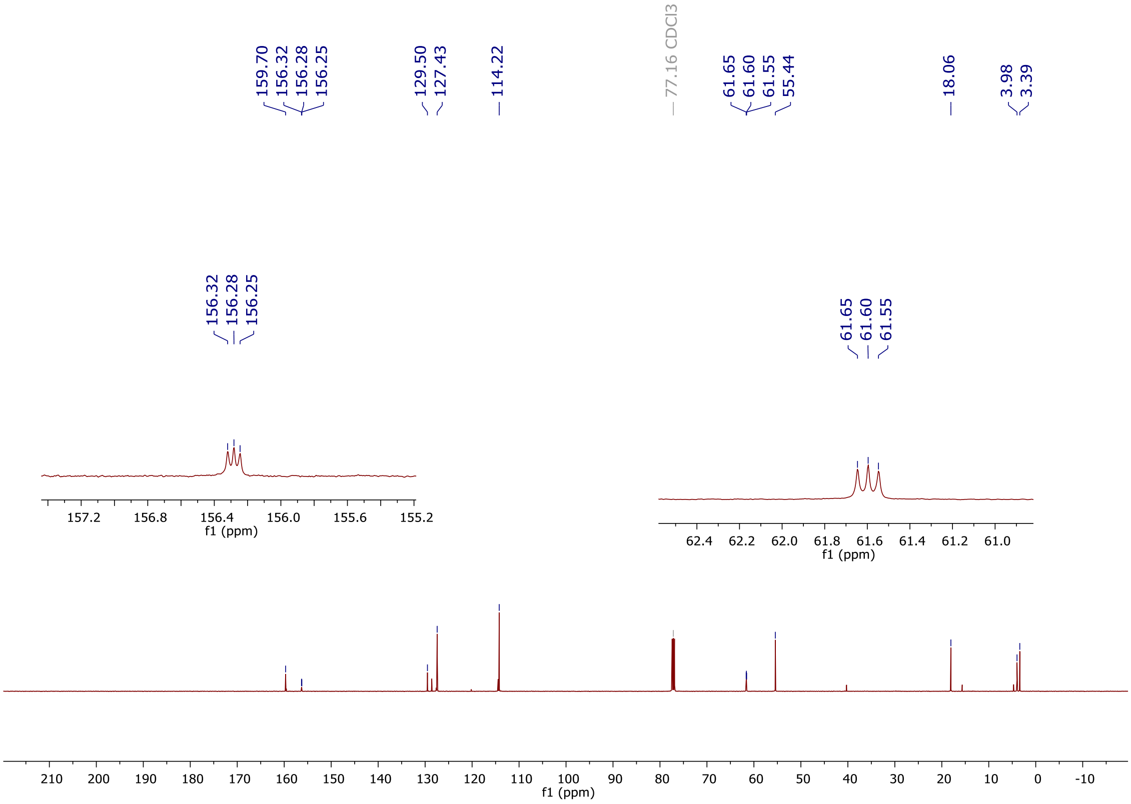

^{1}H-NMR (300 MHz, $CDCl_3$) of compound **1g**

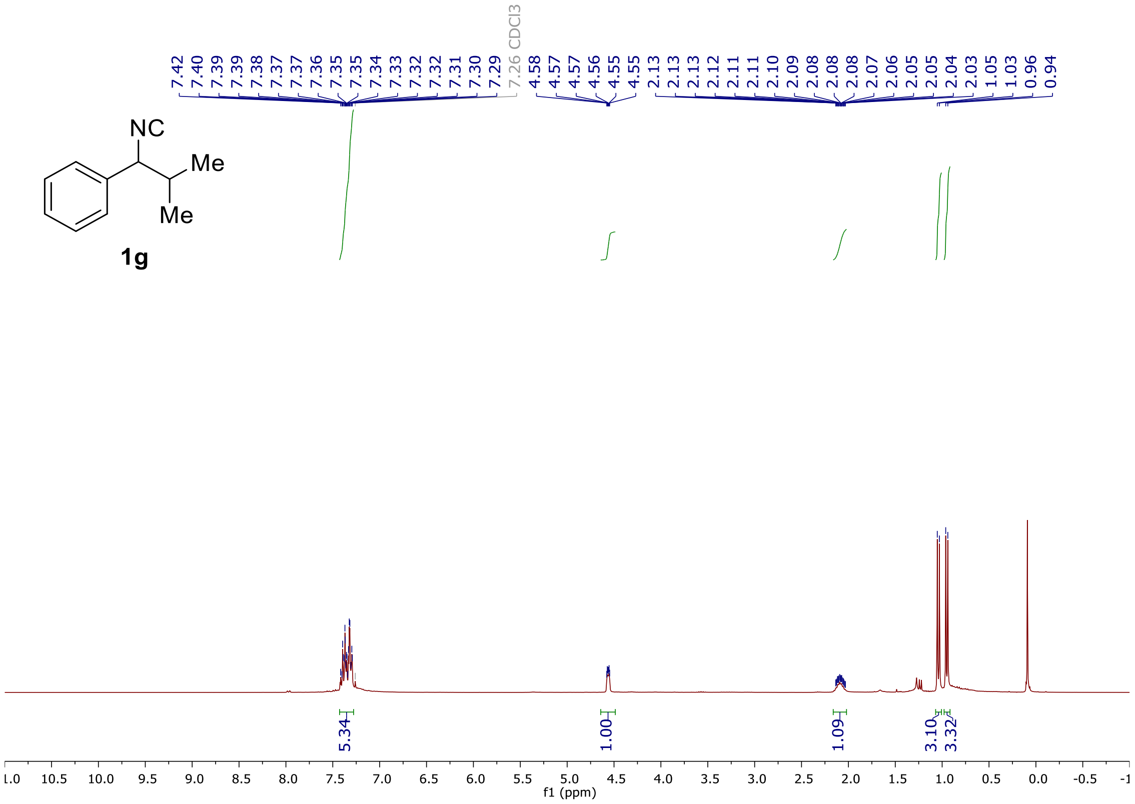

^{13}C-NMR (75 MHz, $CDCl_3$) of compound **1g**

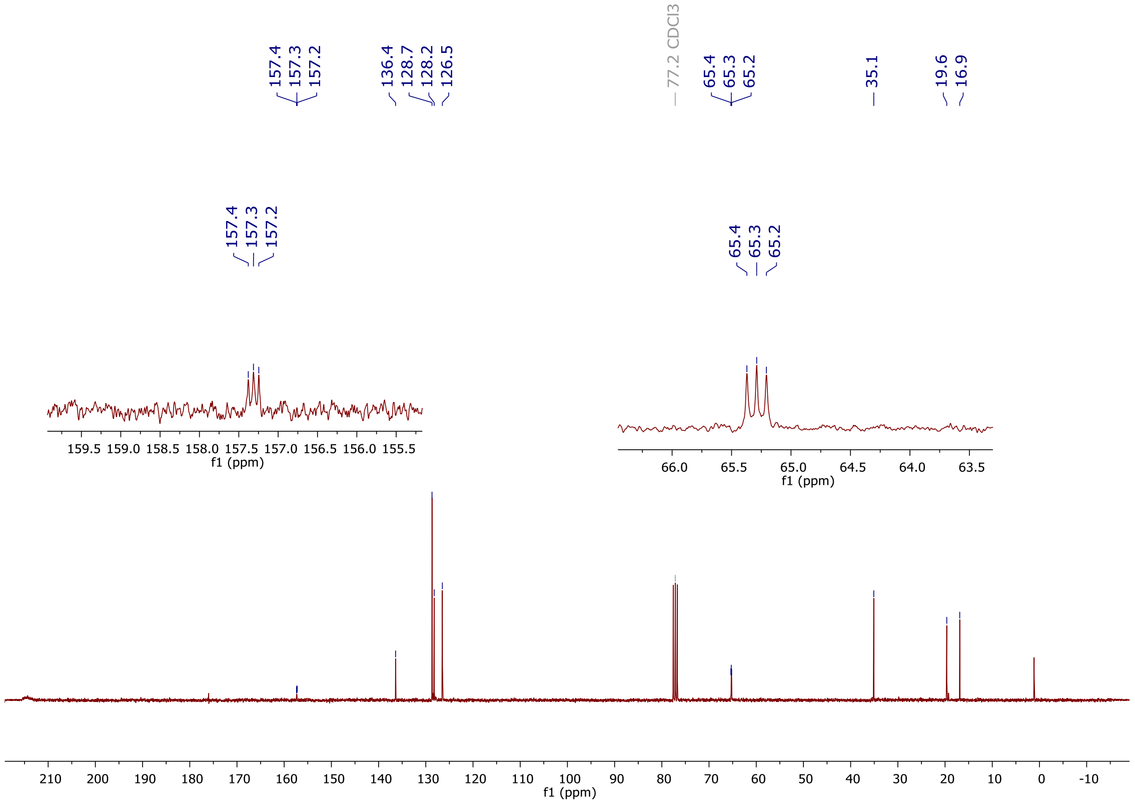

^{1}**H-NMR** (300 MHz, $CDCl_3$) of compound **1h**

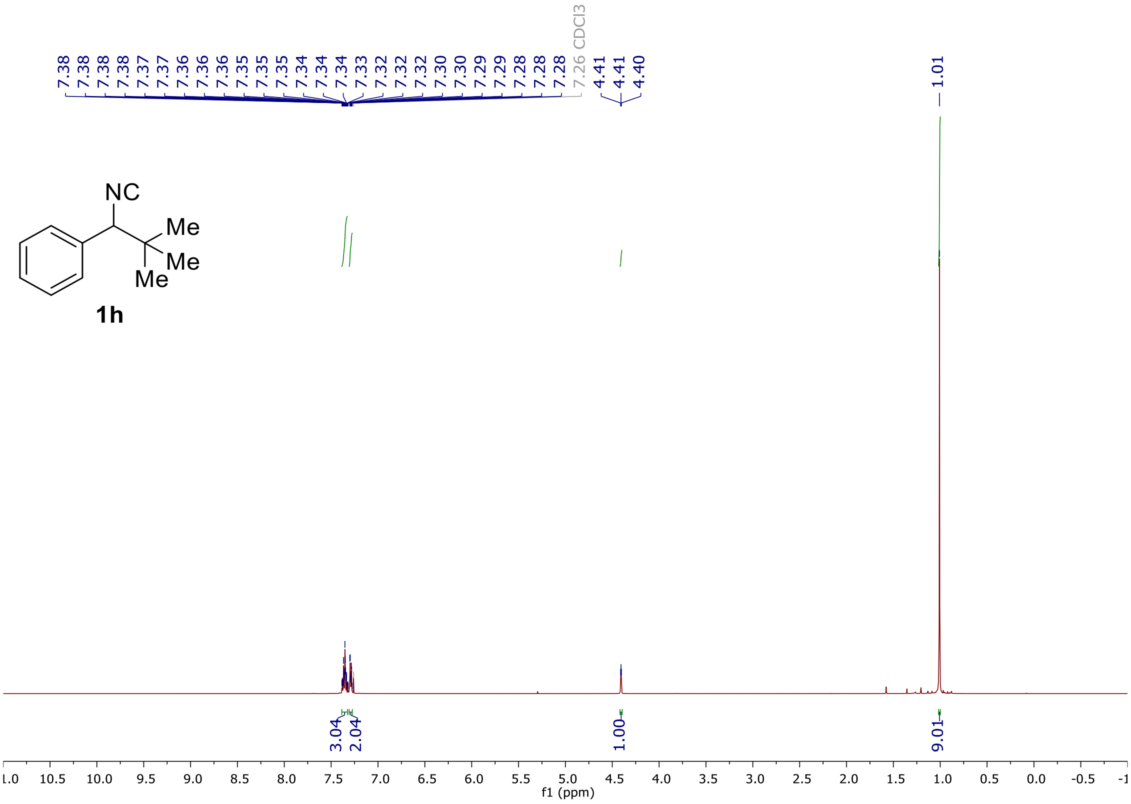

^{13}**C-NMR** (75 MHz, $CDCl_3$) of compound **1h**

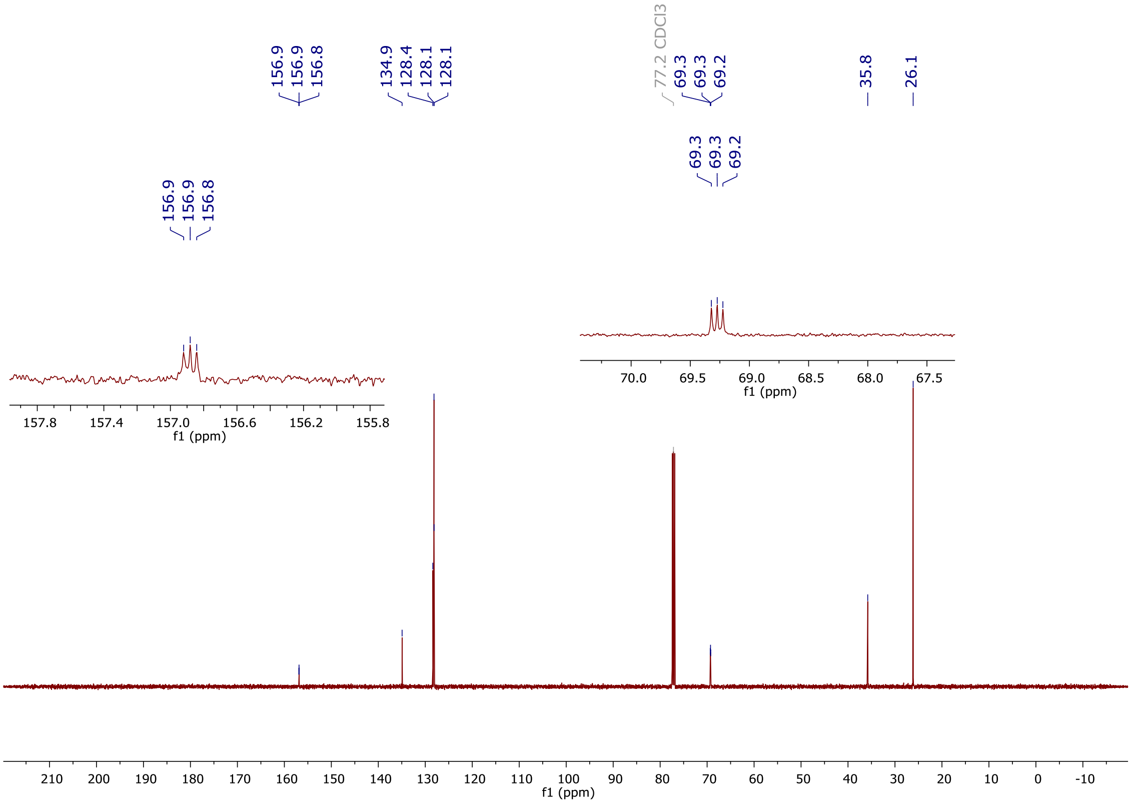

^{1}H-NMR (300 MHz, $CDCl_3$) of compound **1p**

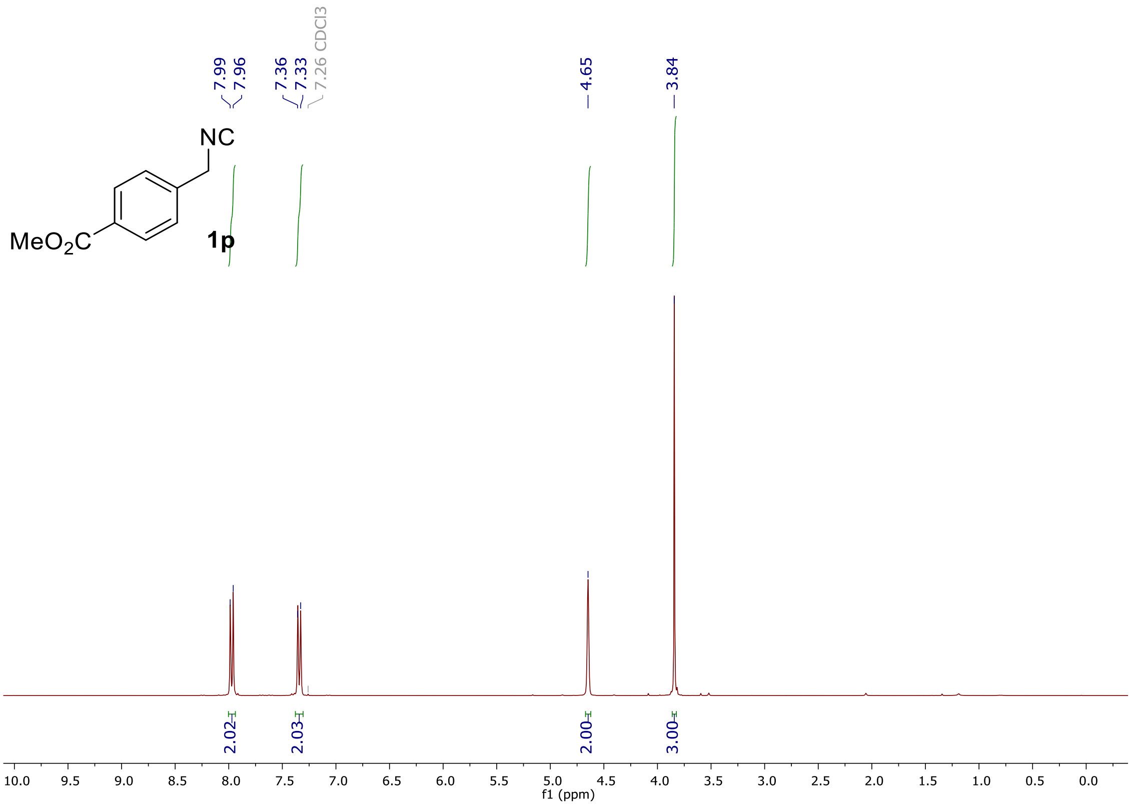

^{13}C-NMR (75 MHz, $CDCl_3$) of compound **1p**

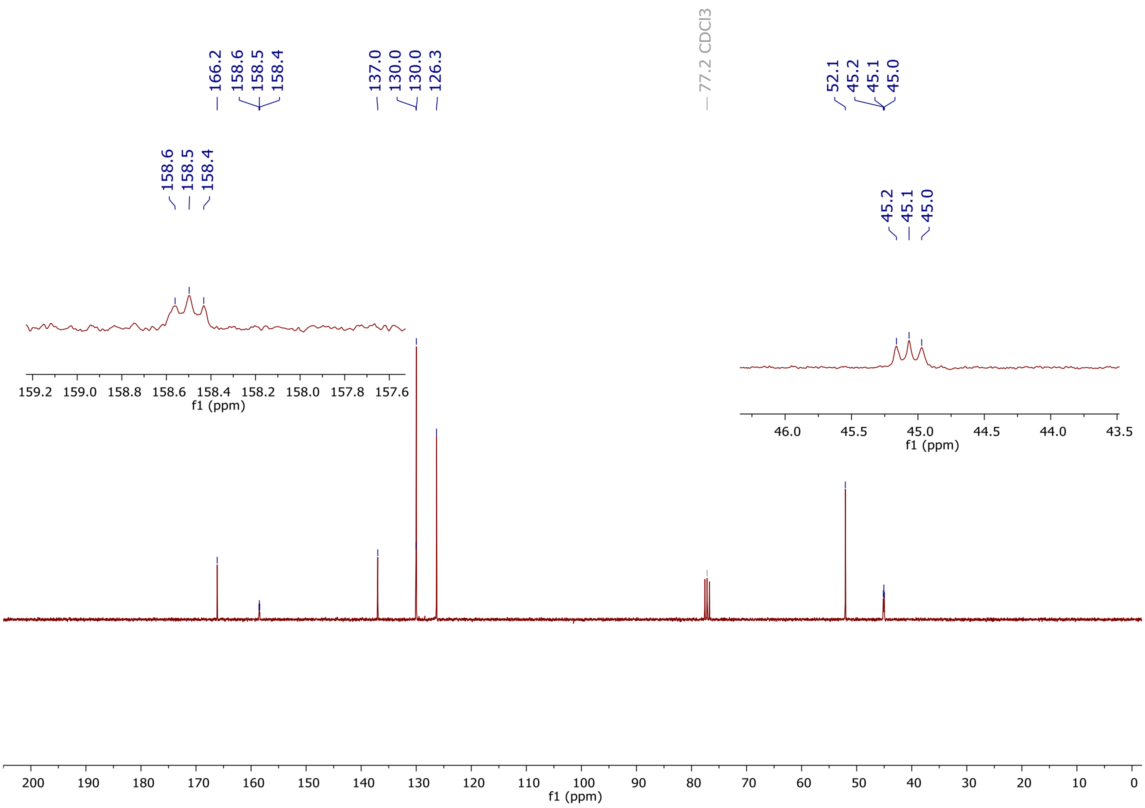

^{1}H-NMR (300 MHz, $CDCl_3$) of compound **1q**

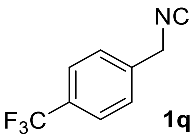
1q

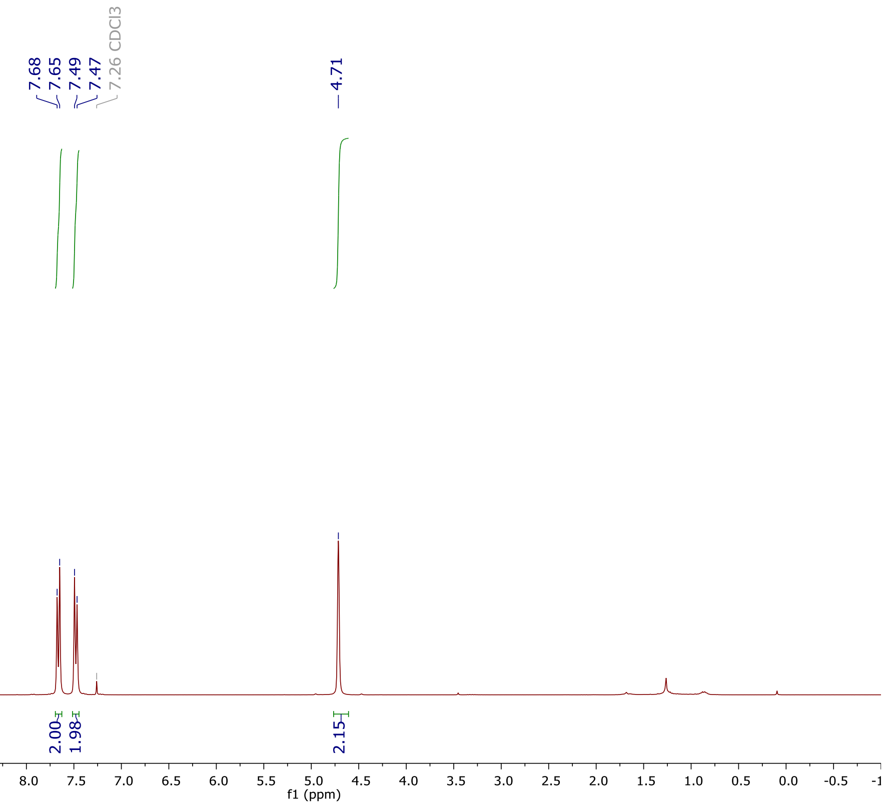

^{1}H-NMR (300 MHz, $CDCl_3$) of compound **1t**

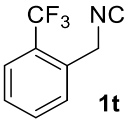

1t

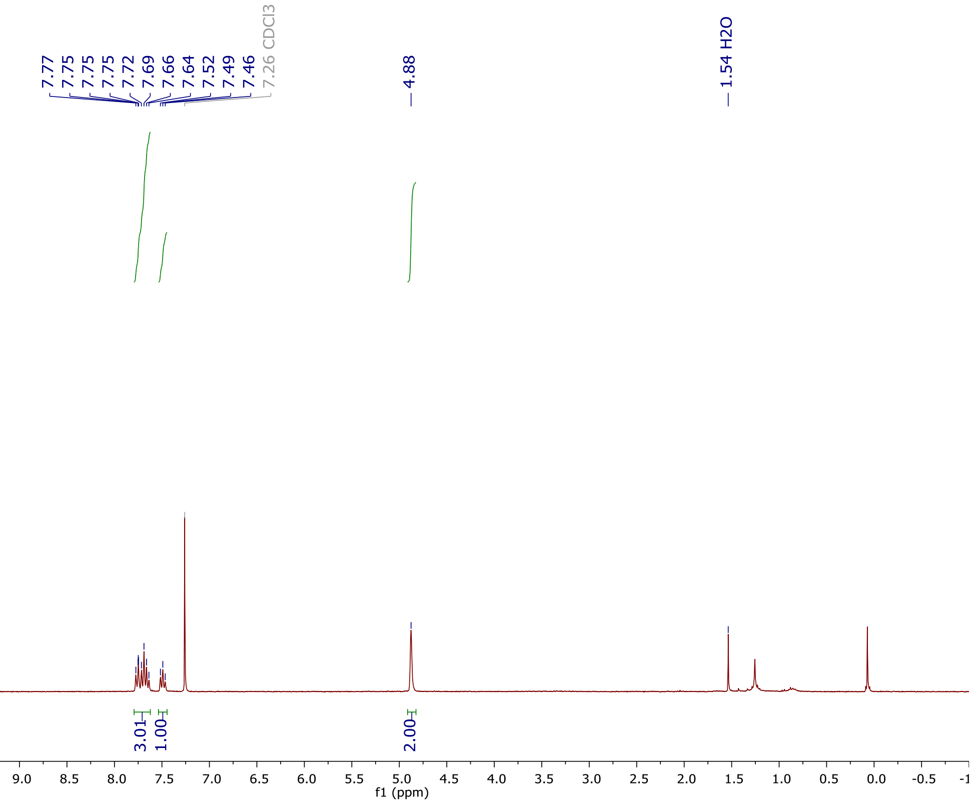

^{1}H-NMR (300 MHz, $CDCl_3$) of compound **1w**

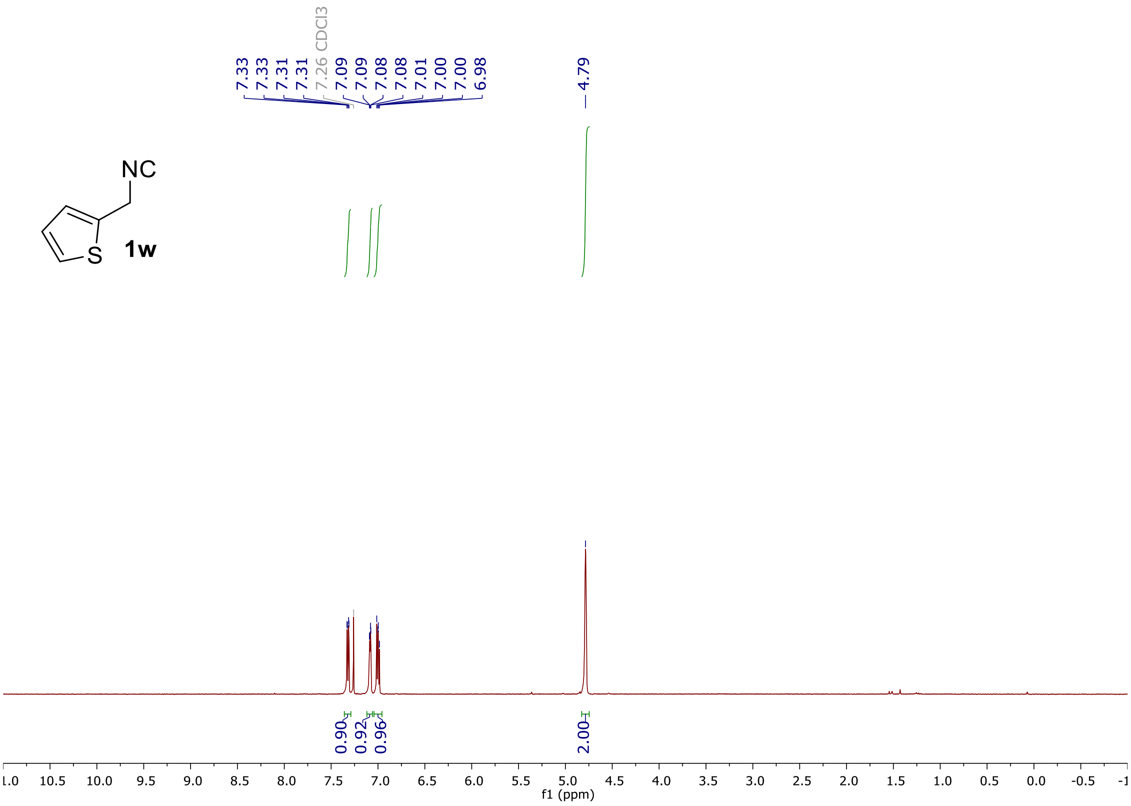

^{13}C-NMR (75 MHz, $CDCl_3$) of compound **1w**

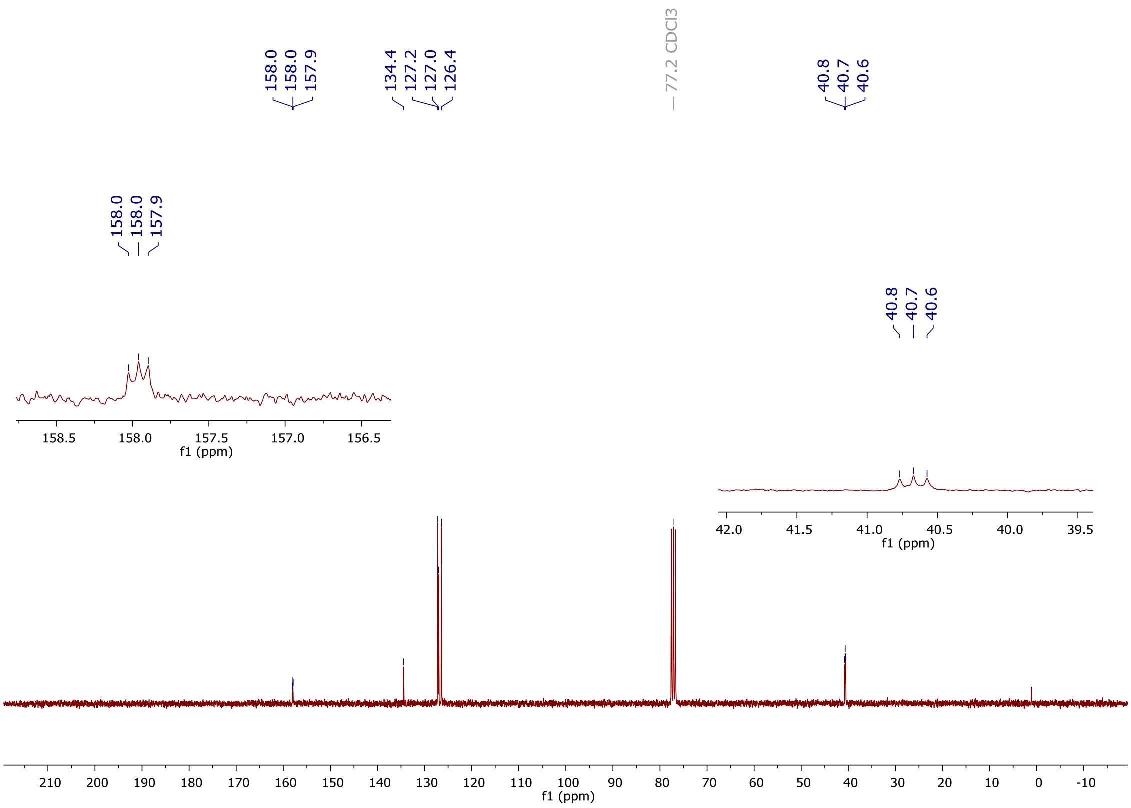

^{1}H-NMR (300 MHz, $CDCl_3$) of compound **1x**

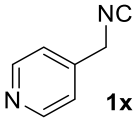

1x

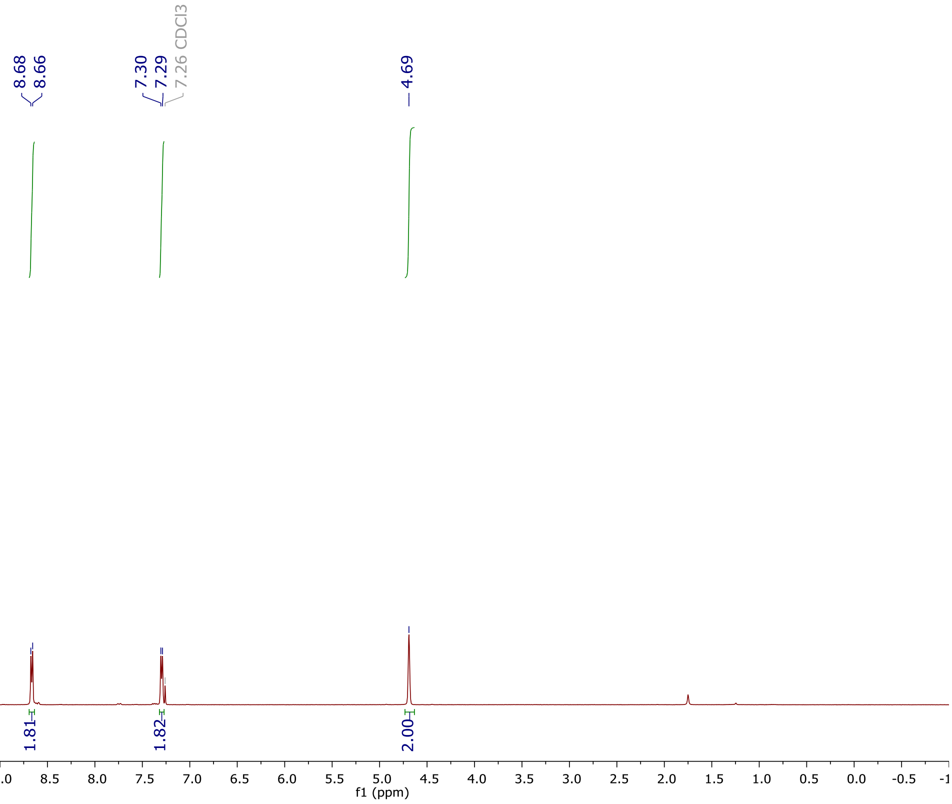

^{13}C-NMR (75 MHz, $CDCl_3$) of compound **1x**

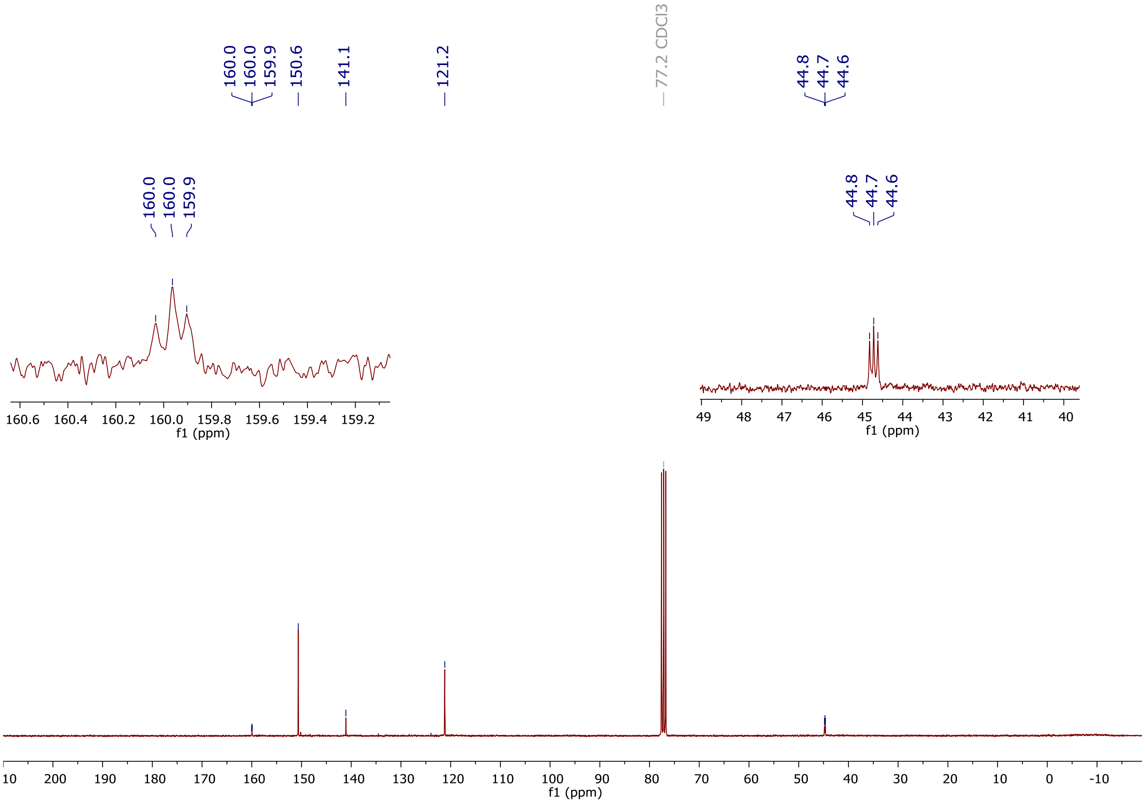

^{1}H-NMR (300 MHz, $CDCl_3$) of compound **1ac**

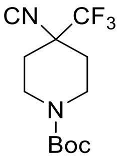

1ac

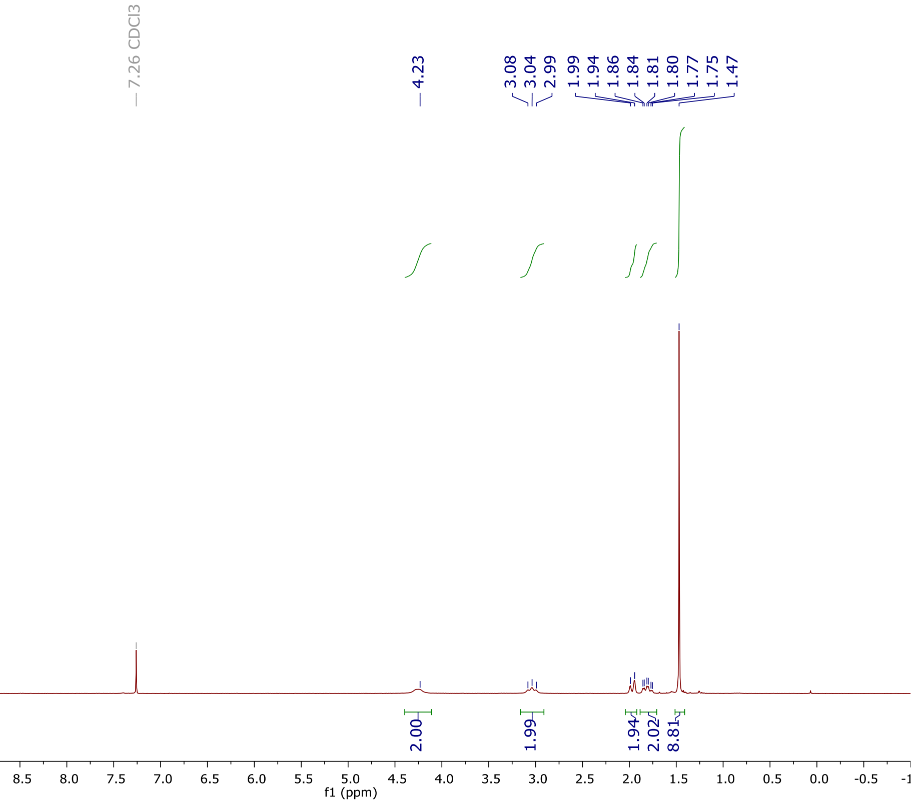

^{13}C-NMR (75 MHz, $CDCl_3$) of compound **1ac**

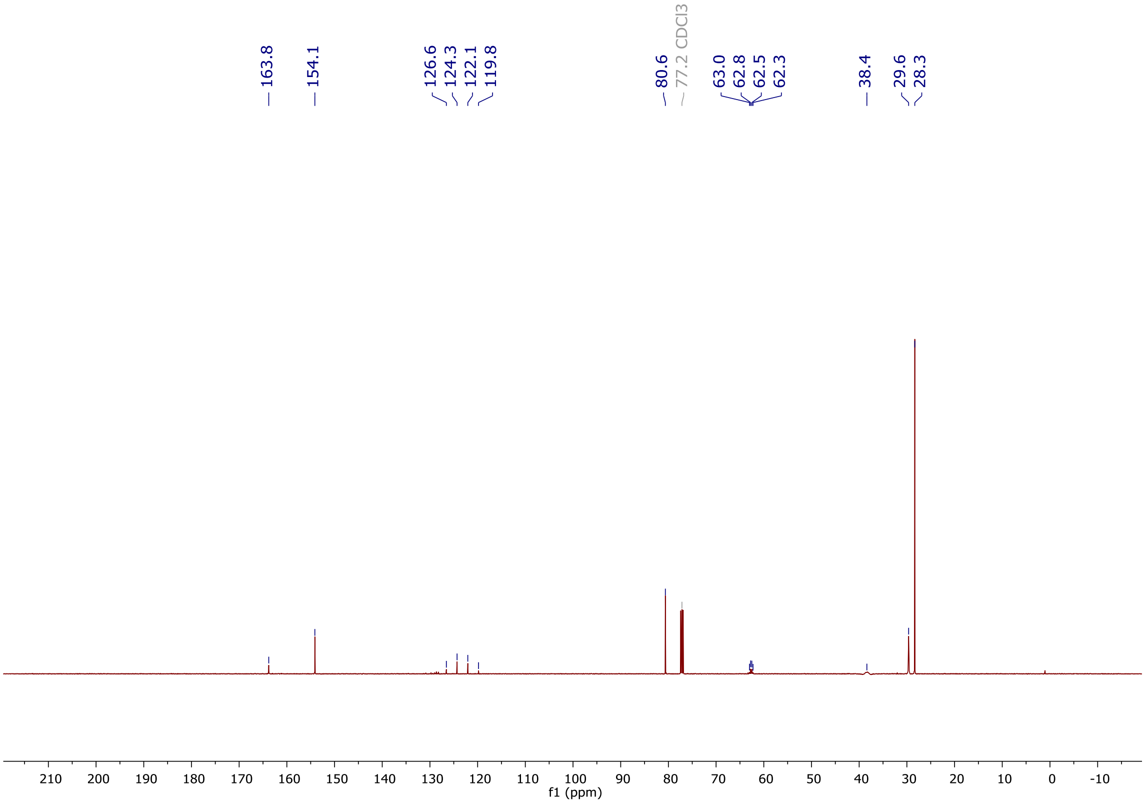

^{19}F-NMR (470 MHz, $CDCl_3$) of compound **1ac**

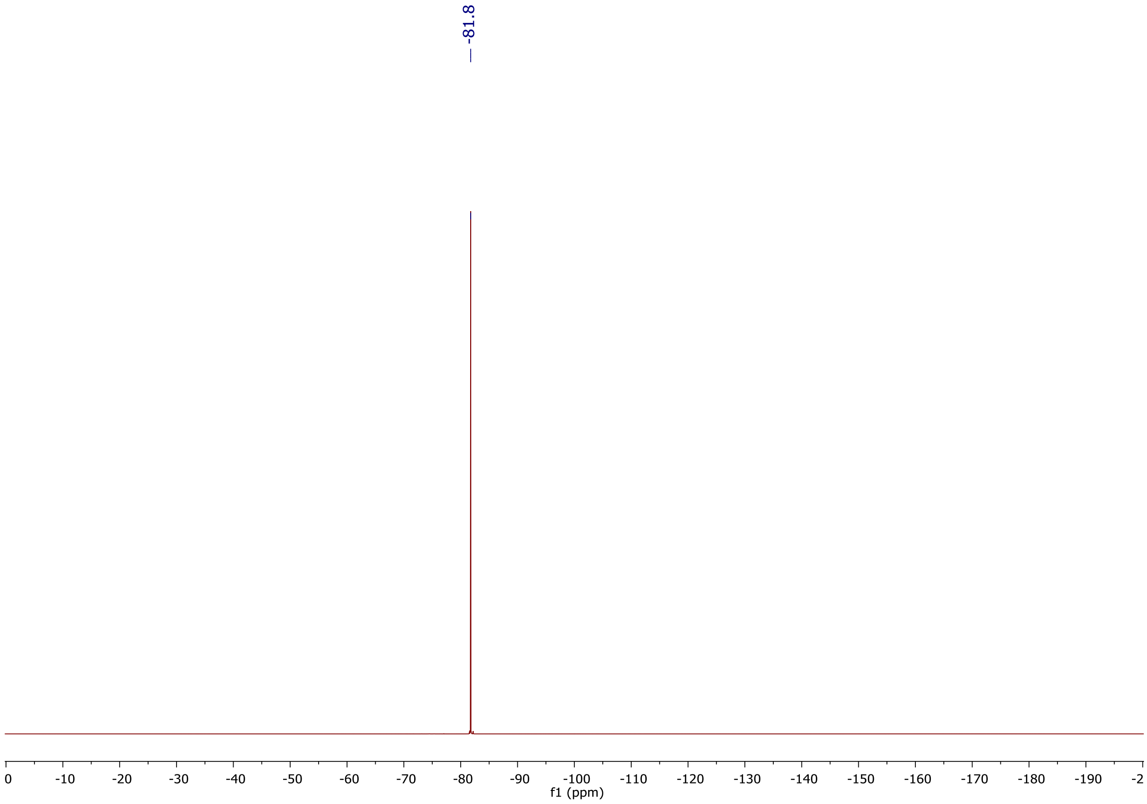

^{1}H-NMR (300 MHz, $CDCl_3$) of compound **1af**

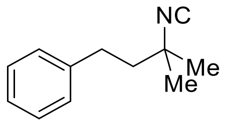

1af

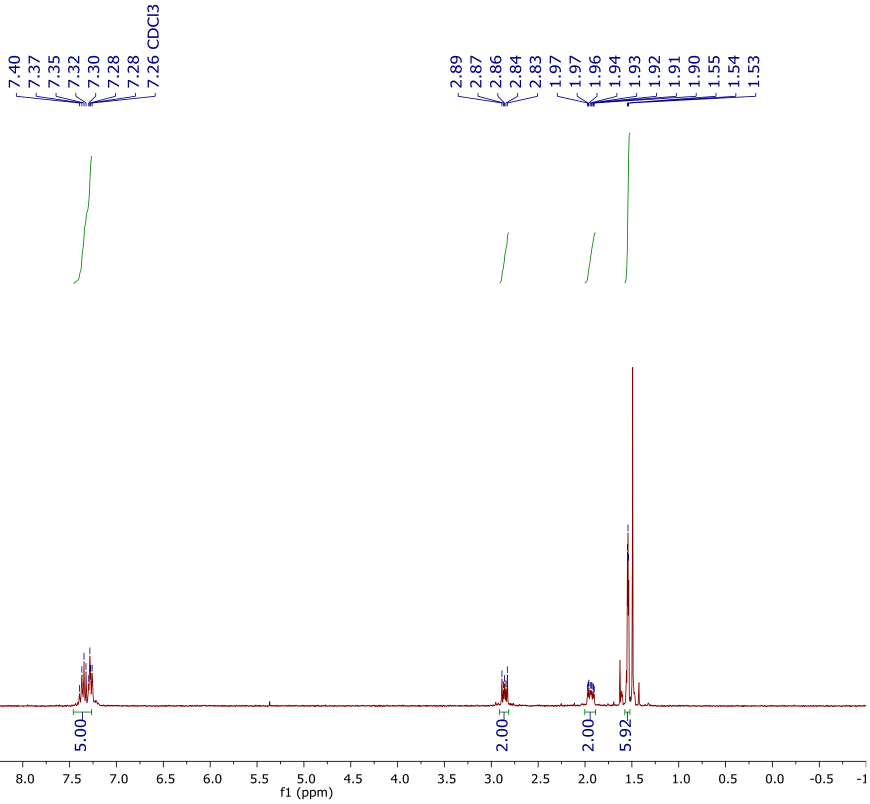

^{1}H-NMR (300 MHz, $CDCl_3$) of compound **1ah**

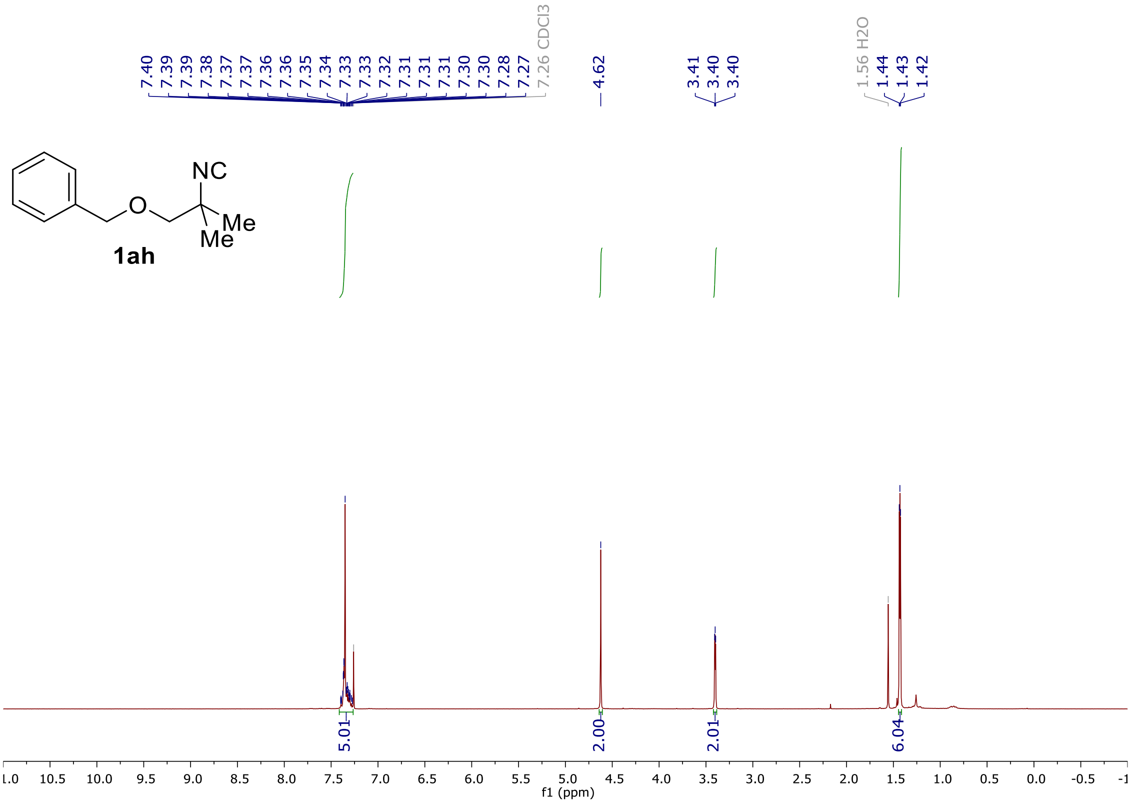

^{13}C-NMR (75 MHz, $CDCl_3$) of compound **1ah**

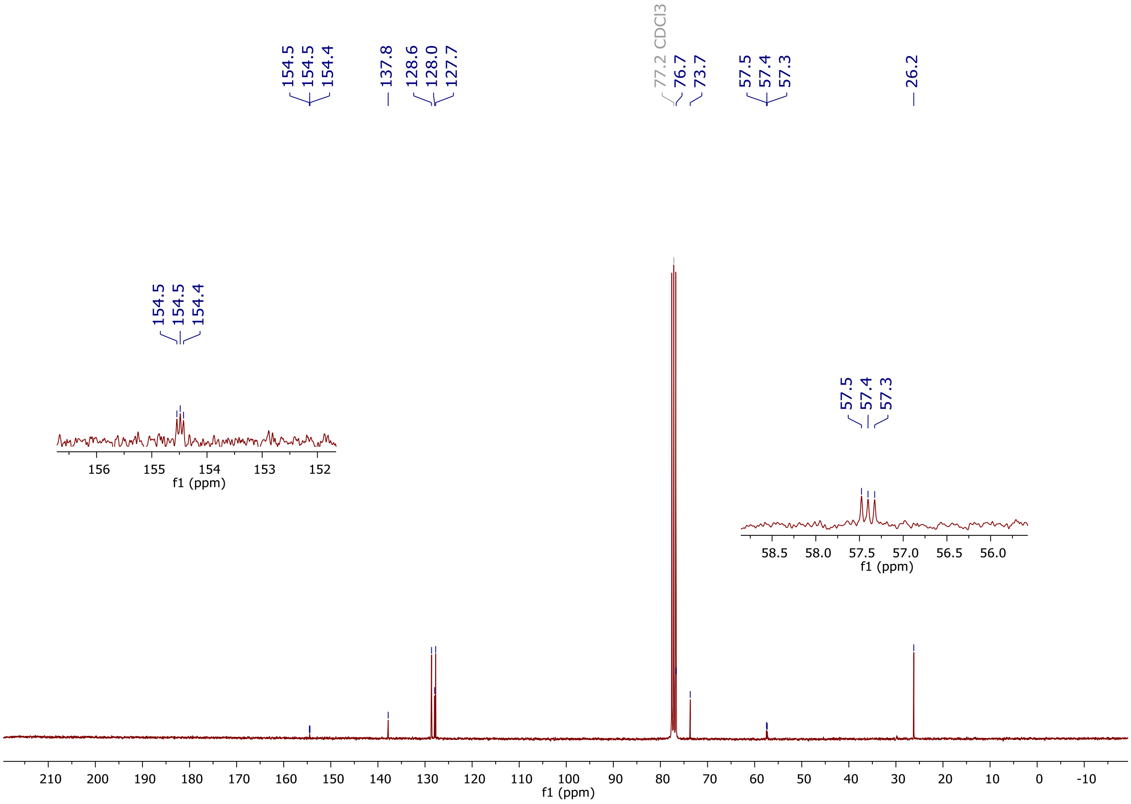

^{1}H-NMR (300 MHz, $CDCl_3$) of compound **1ao**

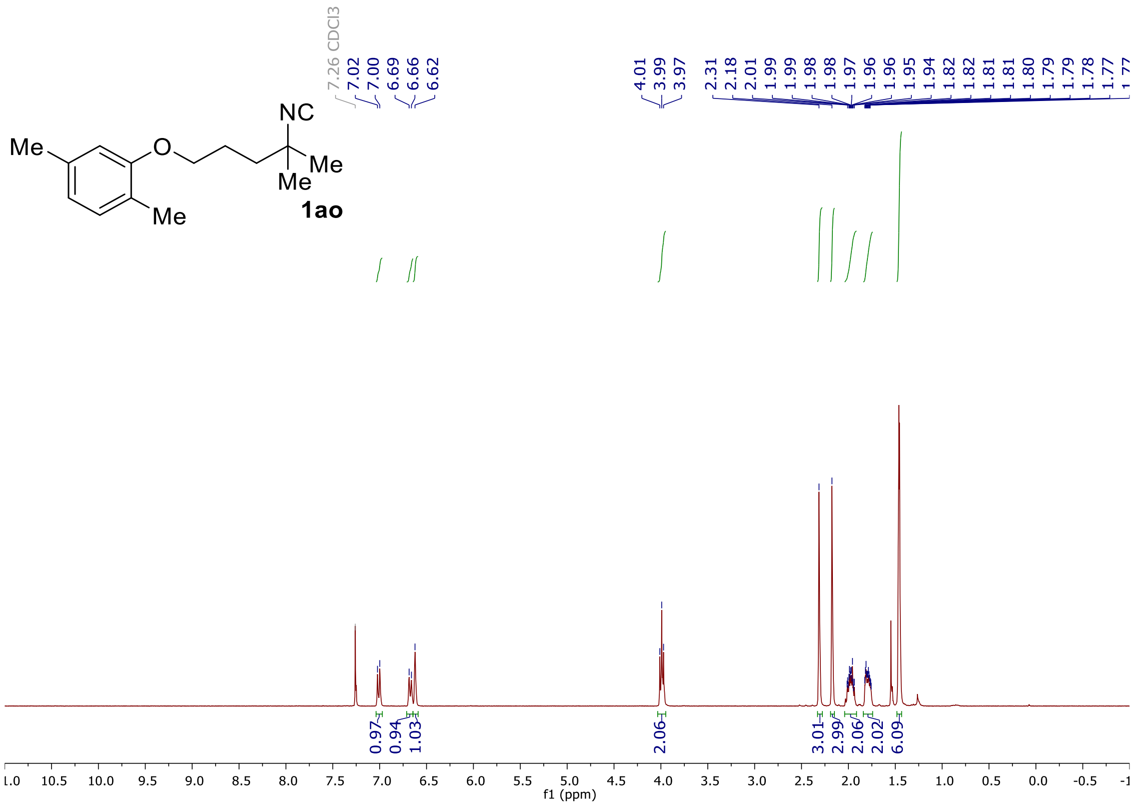

^{13}C-NMR (75 MHz, $CDCl_3$) of compound **1ao**

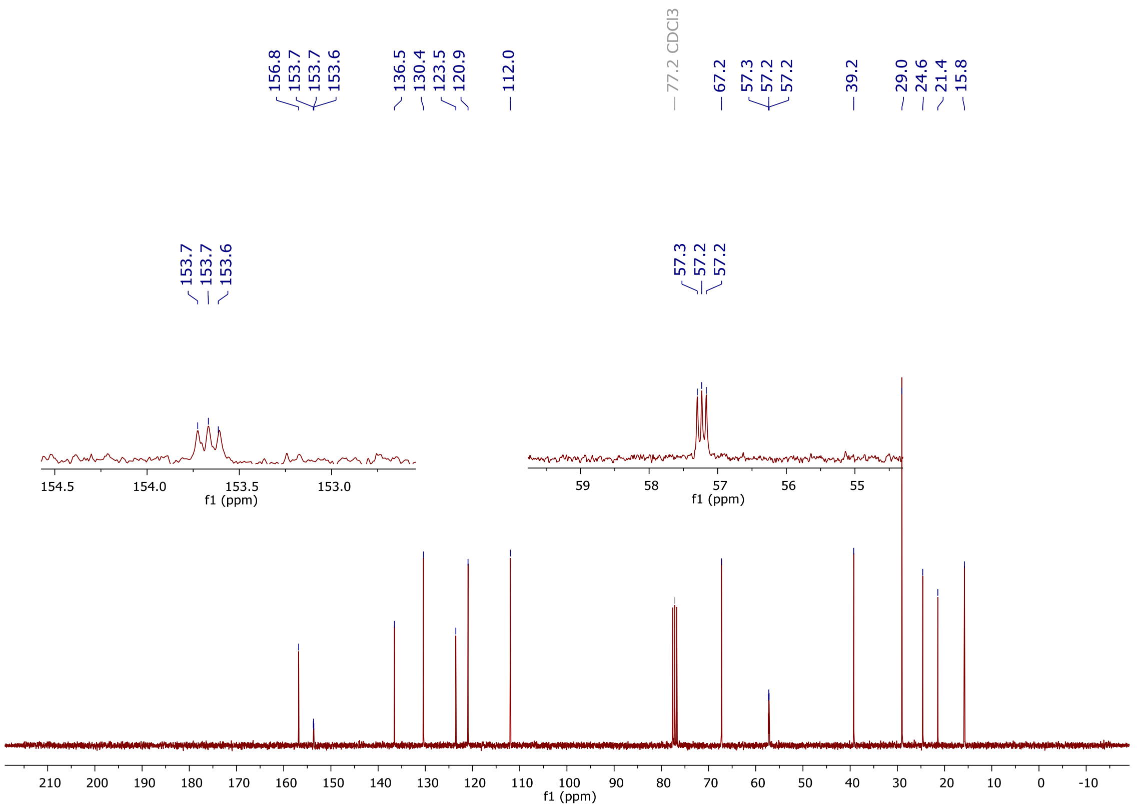

^{1}H-NMR (300 MHz, $CDCl_3$) of compound **1ar**

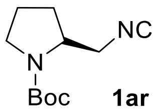

1ar

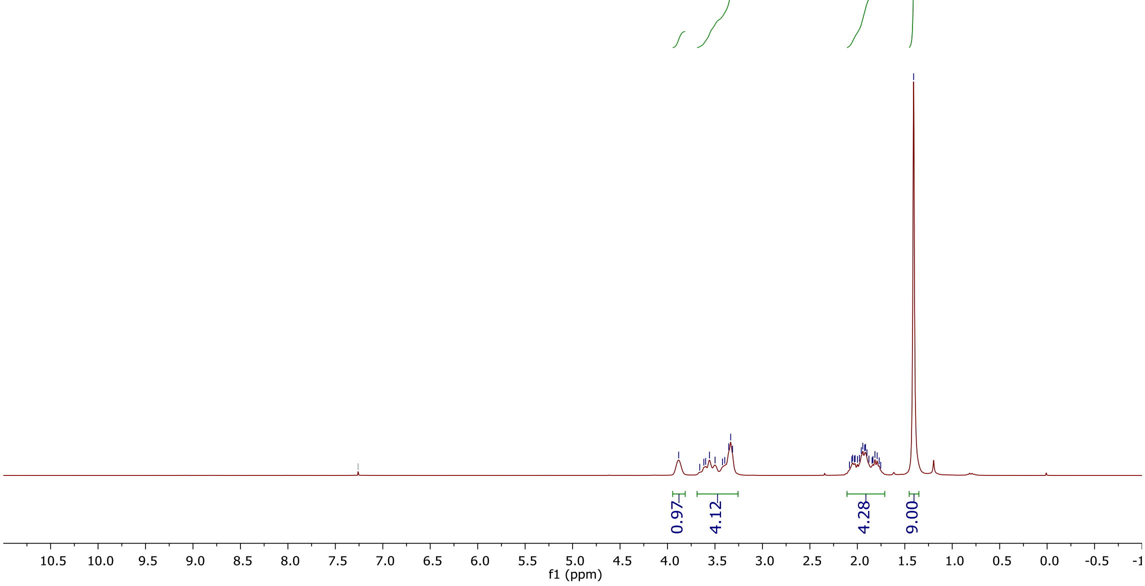

^{1}H-NMR (300 MHz, $CDCl_3$) of compound **2j**

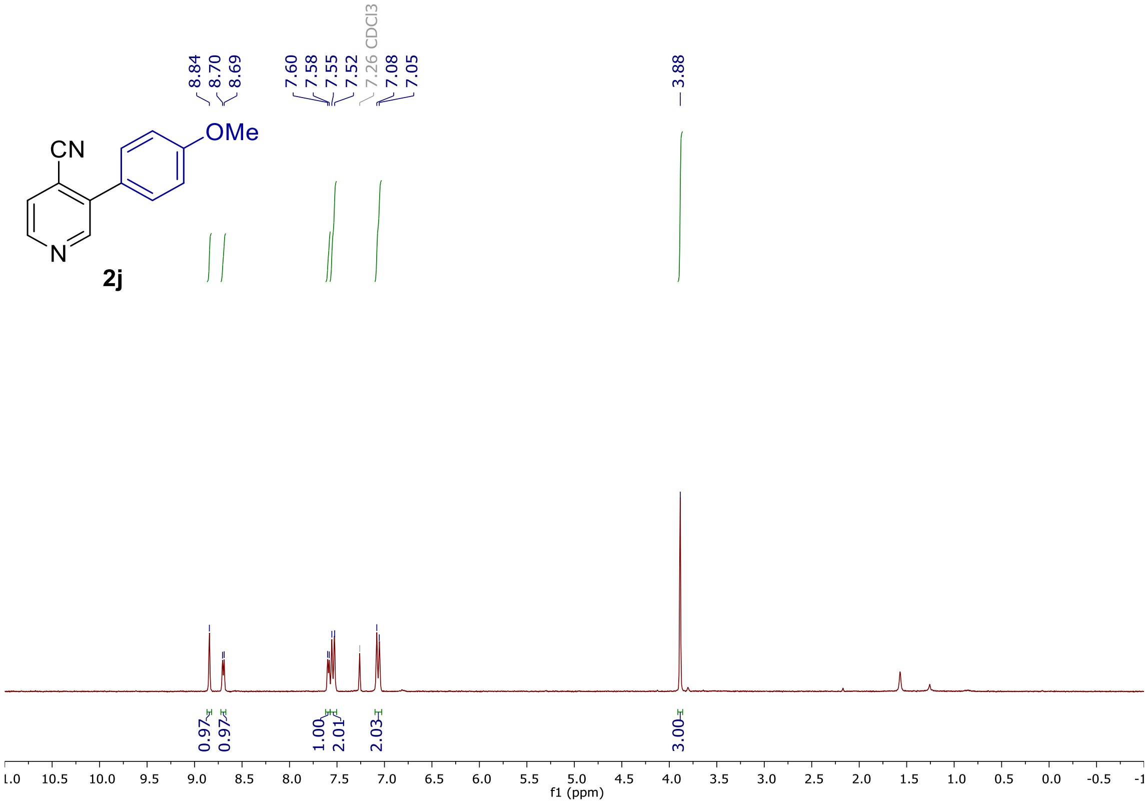

^{1}H-NMR (500 MHz, $CDCl_3$) of compound **2l**

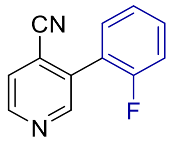

2l

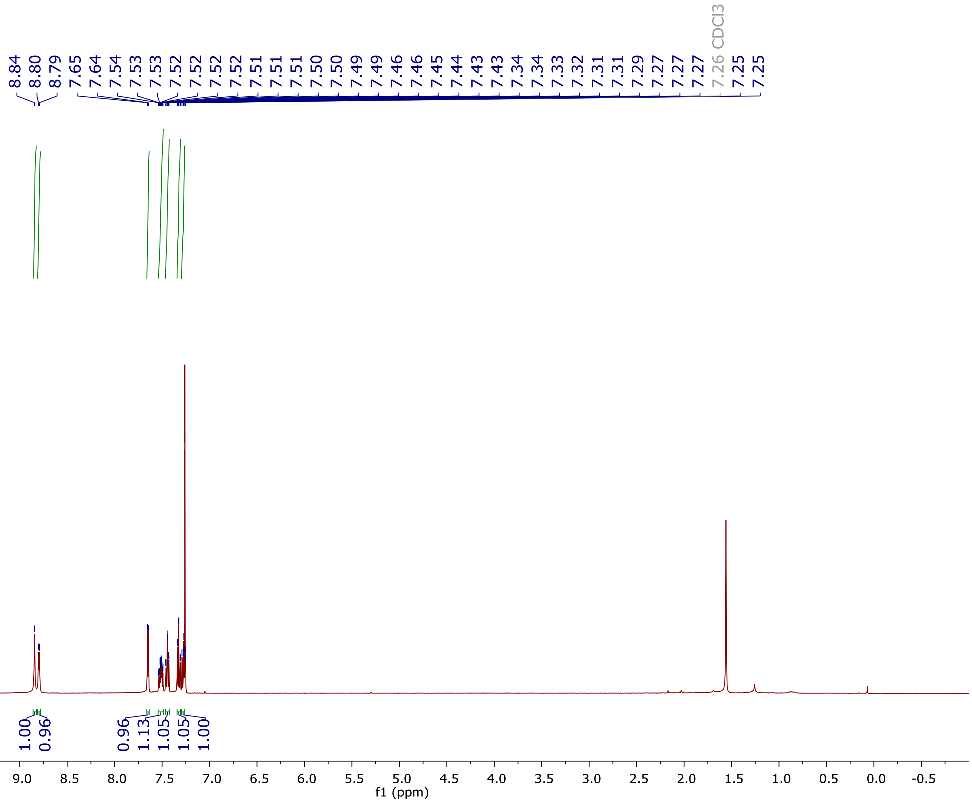

^{1}H-NMR (500 MHz, $CDCl_3$) of compound **2m**

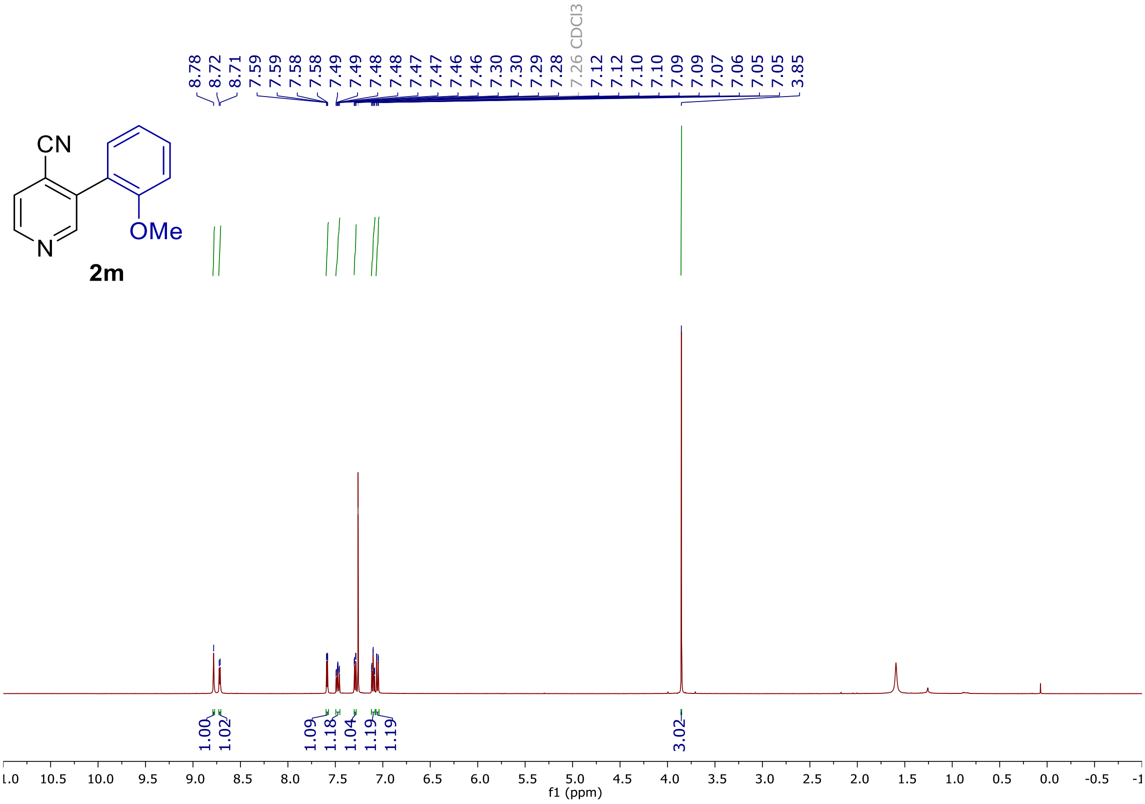

^{13}C-NMR (75 MHz, $CDCl_3$) of compound **2m**

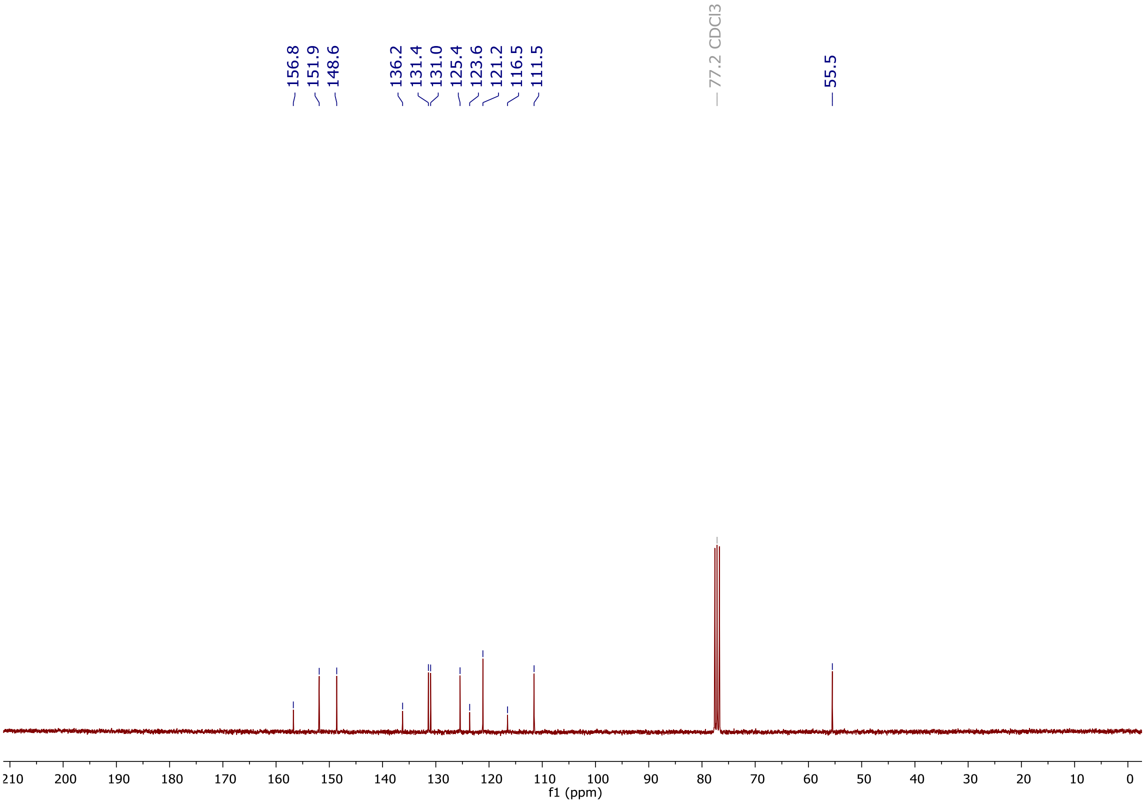

^{1}H-NMR (500 MHz, $CDCl_3$) of compound **2g**

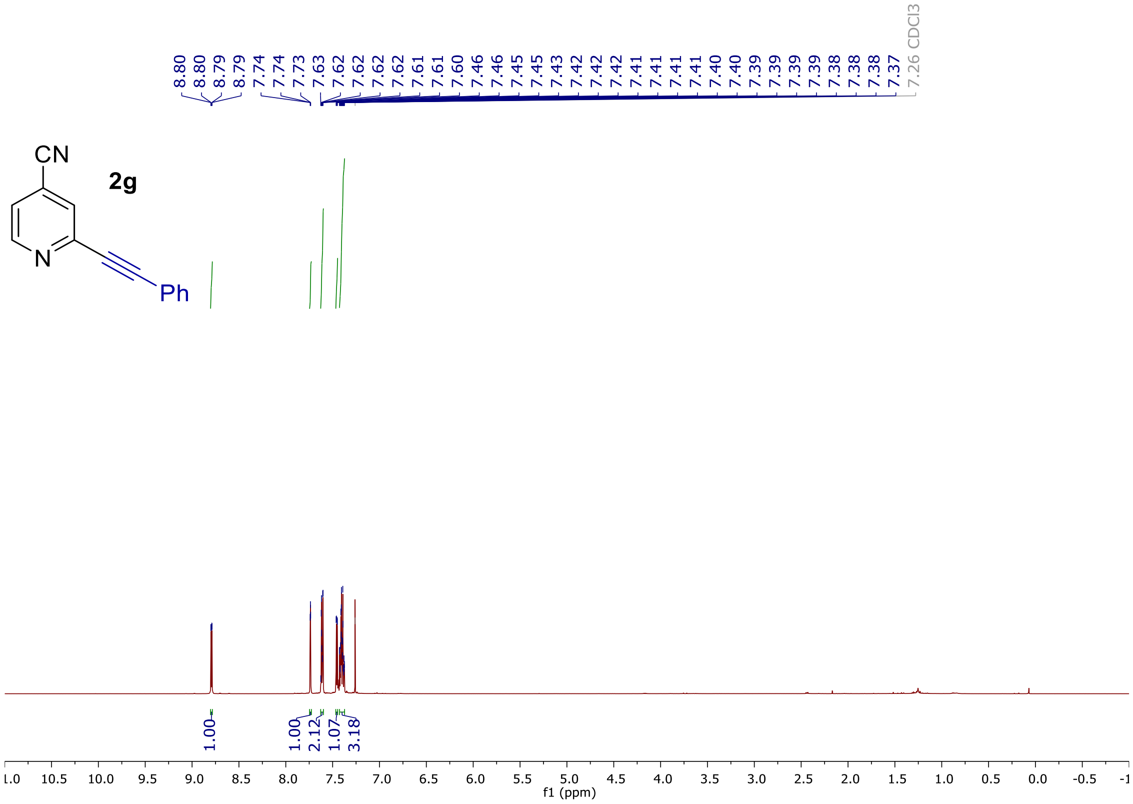

^{13}C-NMR (125 MHz, $CDCl_3$) of compound **2g**

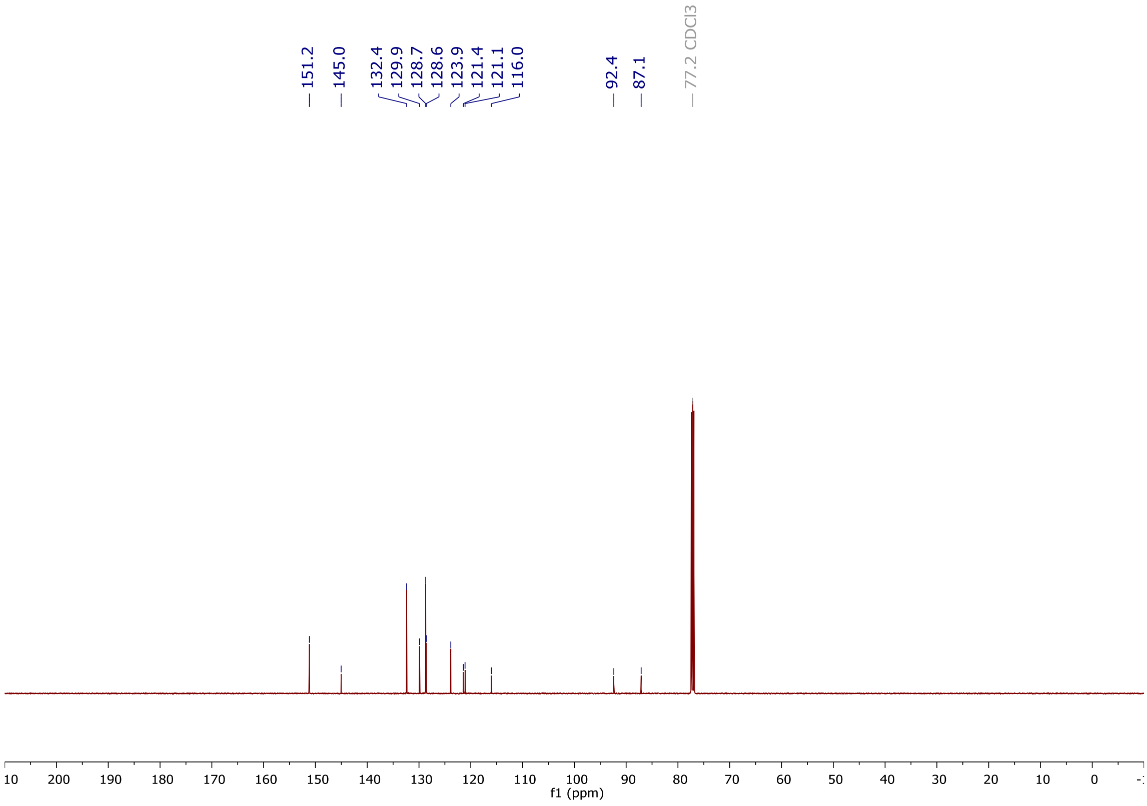

¹H-NMR (500 MHz, $CDCl_3$) of compound **2k**

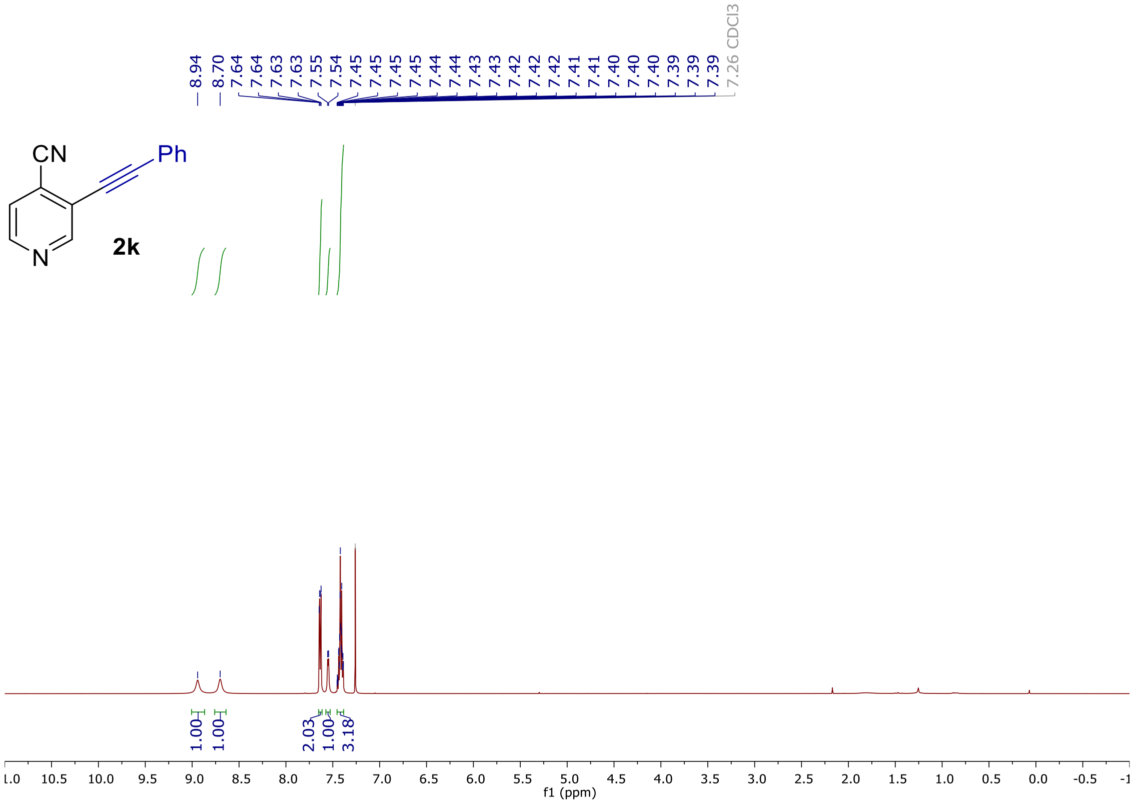

¹³C-NMR (125 MHz, $CDCl_3$) of compound **2k**

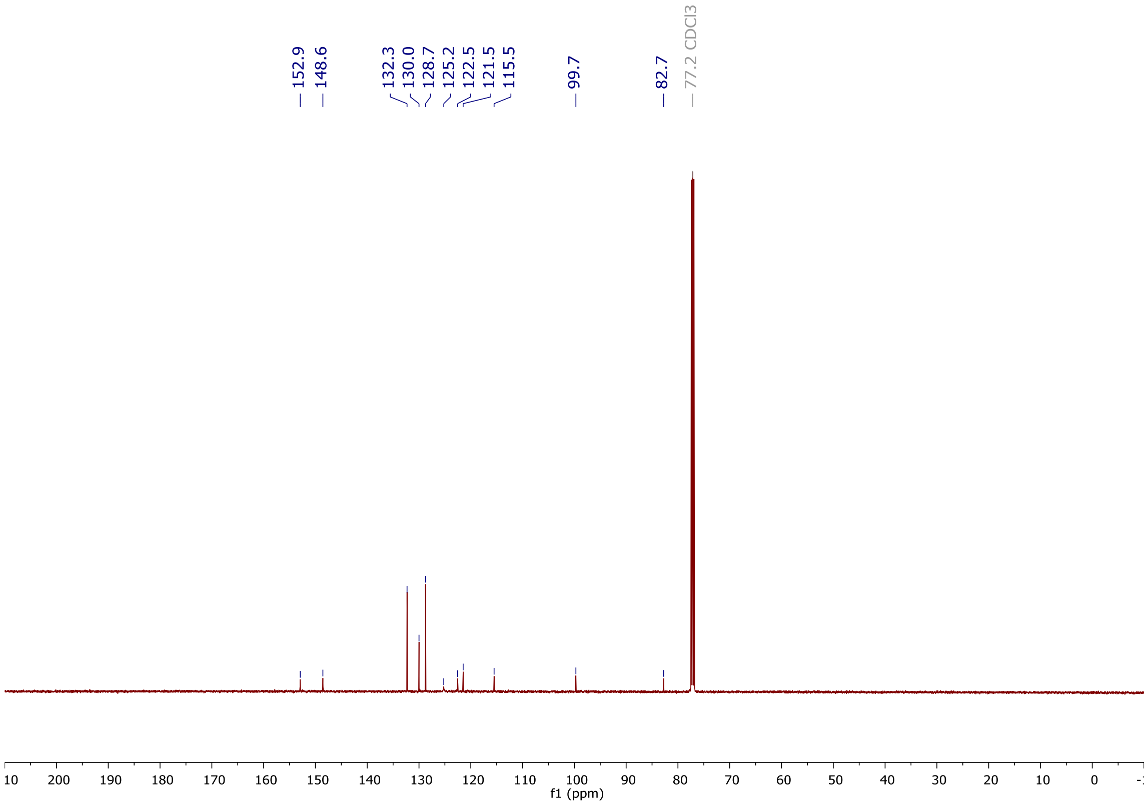

^{1}H-NMR (500 MHz, CD_2Cl_2) of 3DPA2FBN **PC**

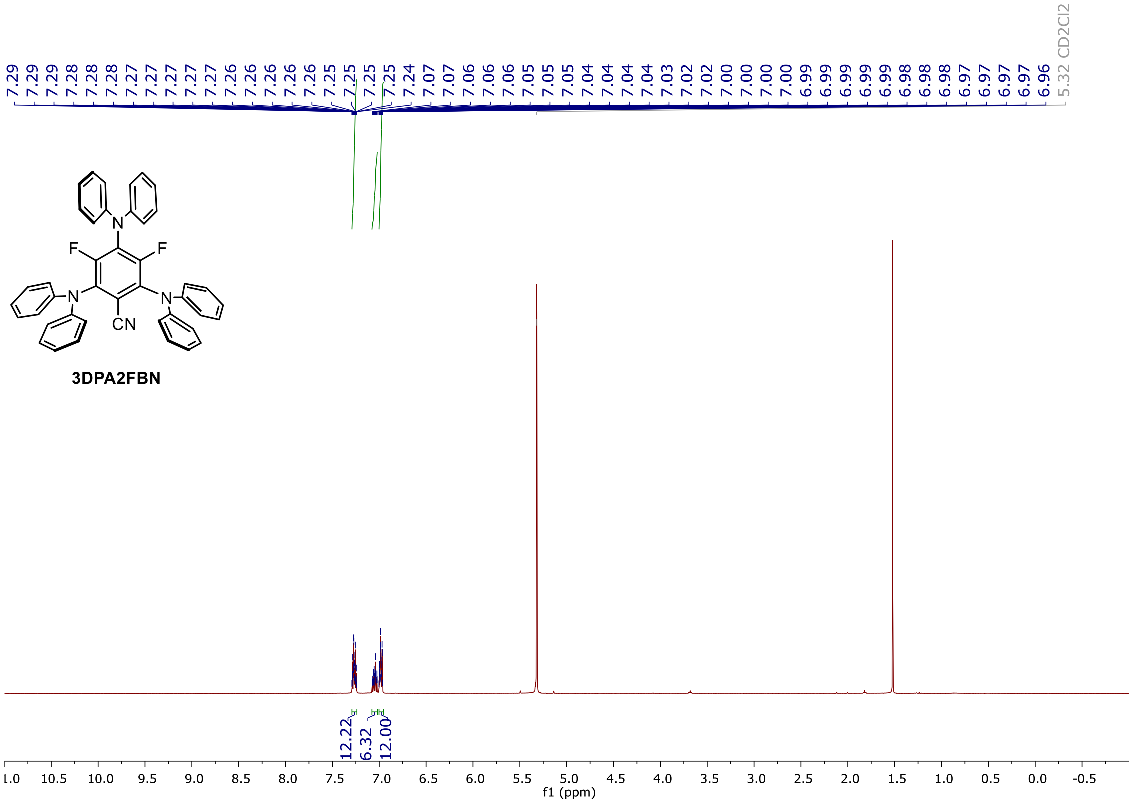

¹H-NMR (300 MHz, C_6D_6) of $TMS_3SiNHAd$ **3**

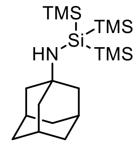

$TMS_3SiNHAd$

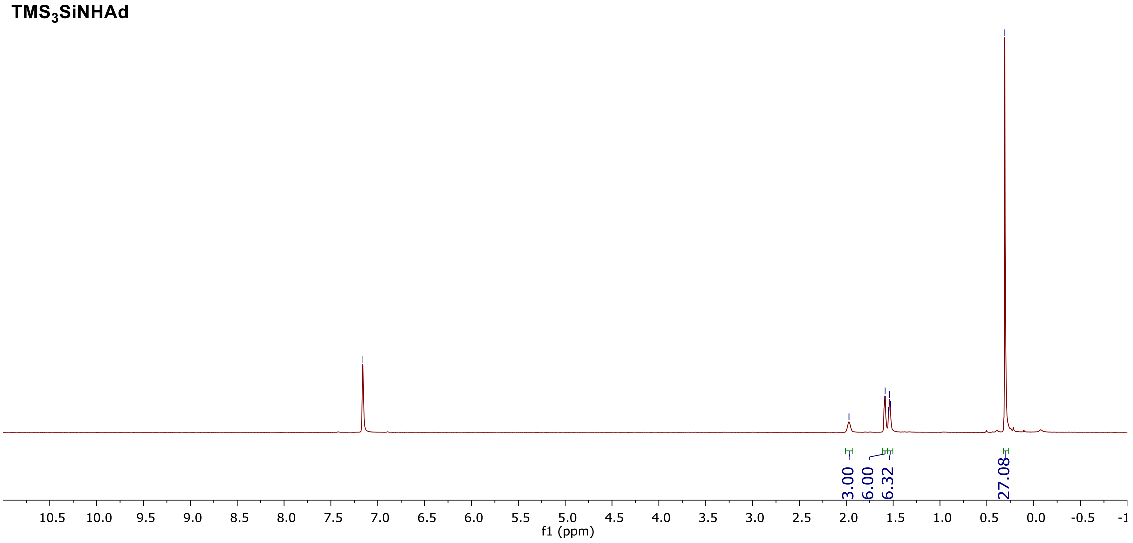

[1]**H-NMR** (500 MHz, $CDCl_3$) of compound **4a**

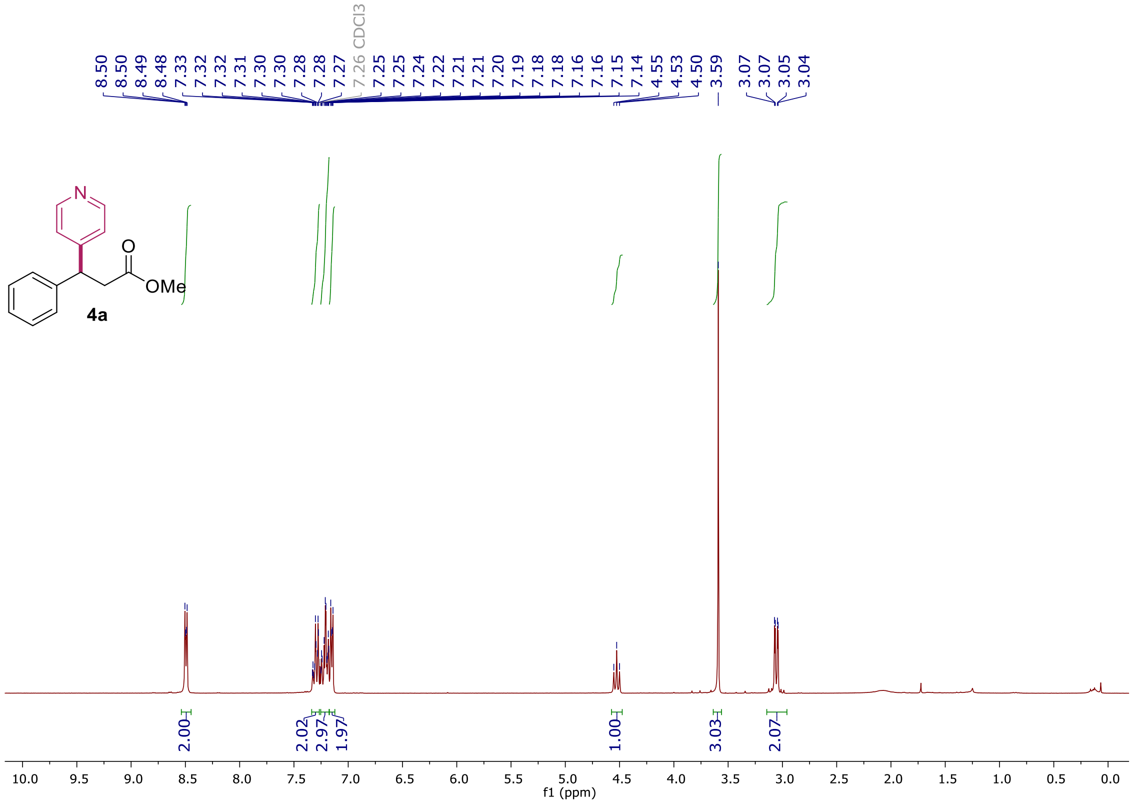

^{1}H-NMR (300 MHz, $CDCl_3$) of compound **4b**

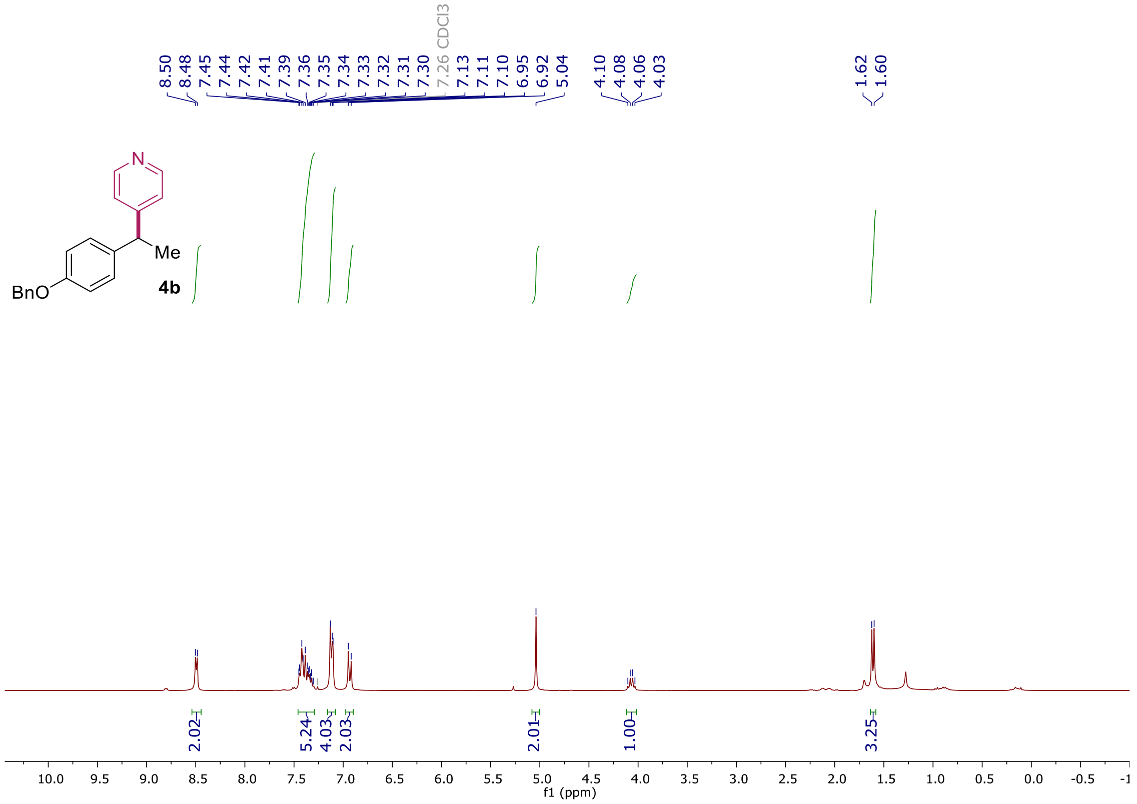

^{13}C-NMR (75 MHz, $CDCl_3$) of compound **4b**

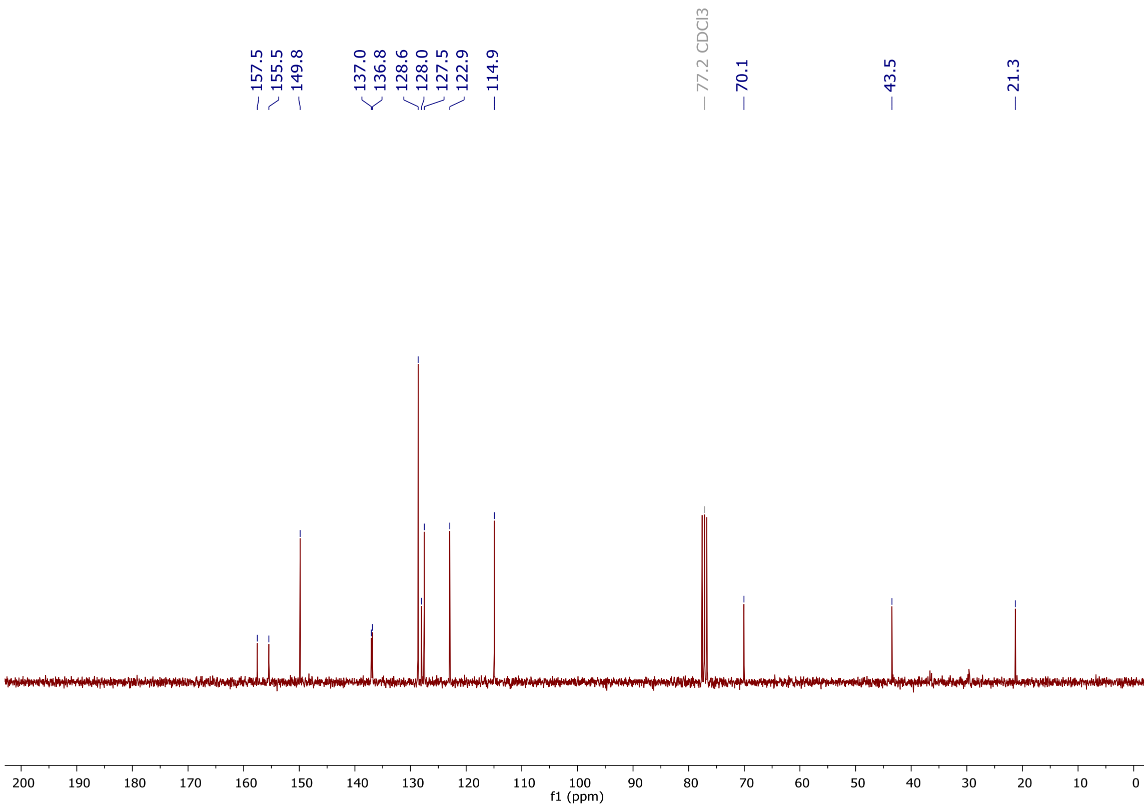

[1]**H-NMR** (300 MHz, $CDCl_3$) of compound **4c**

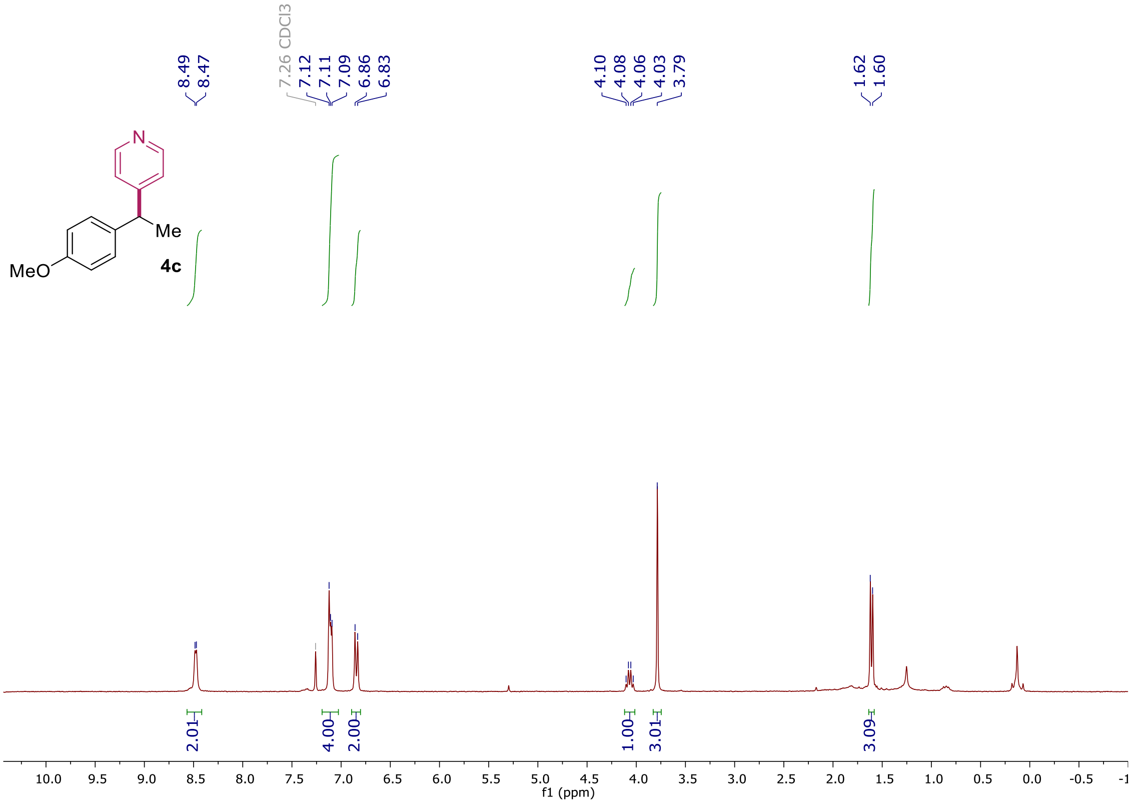

^{1}H-NMR (300 MHz, $CDCl_3$) of compound **4d**

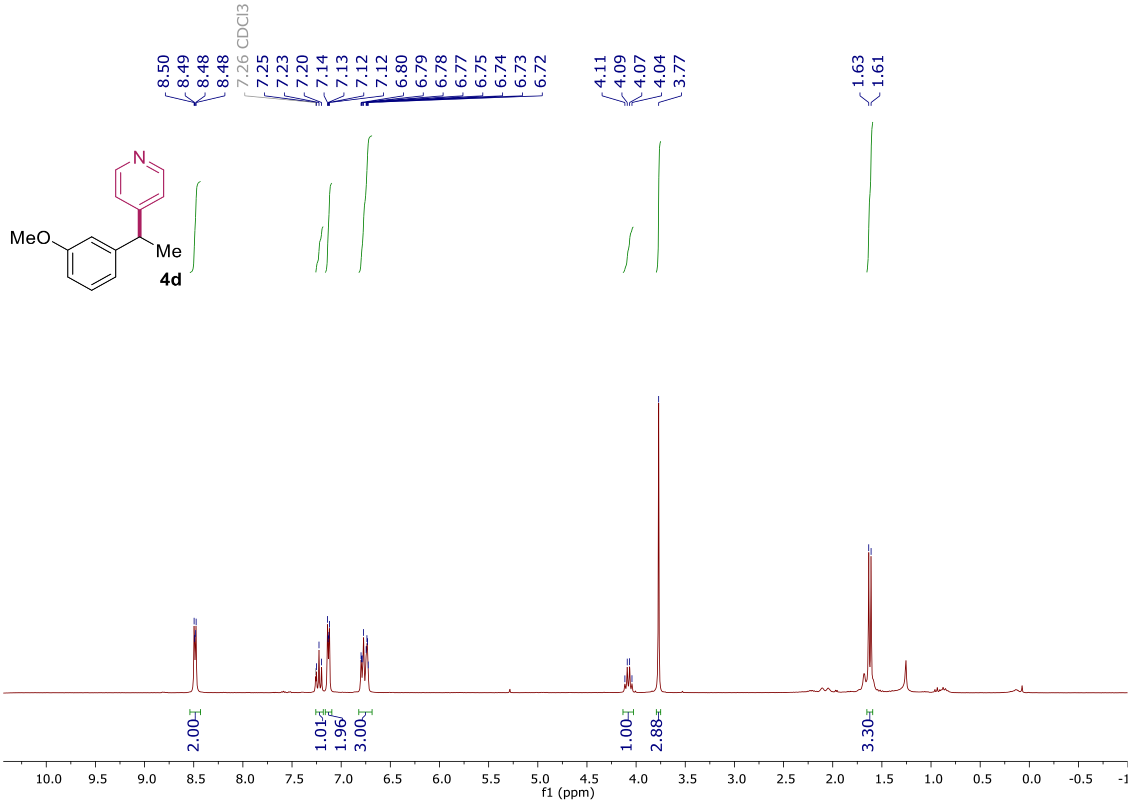

¹H-NMR (300 MHz, $CDCl_3$) of compound **4e**

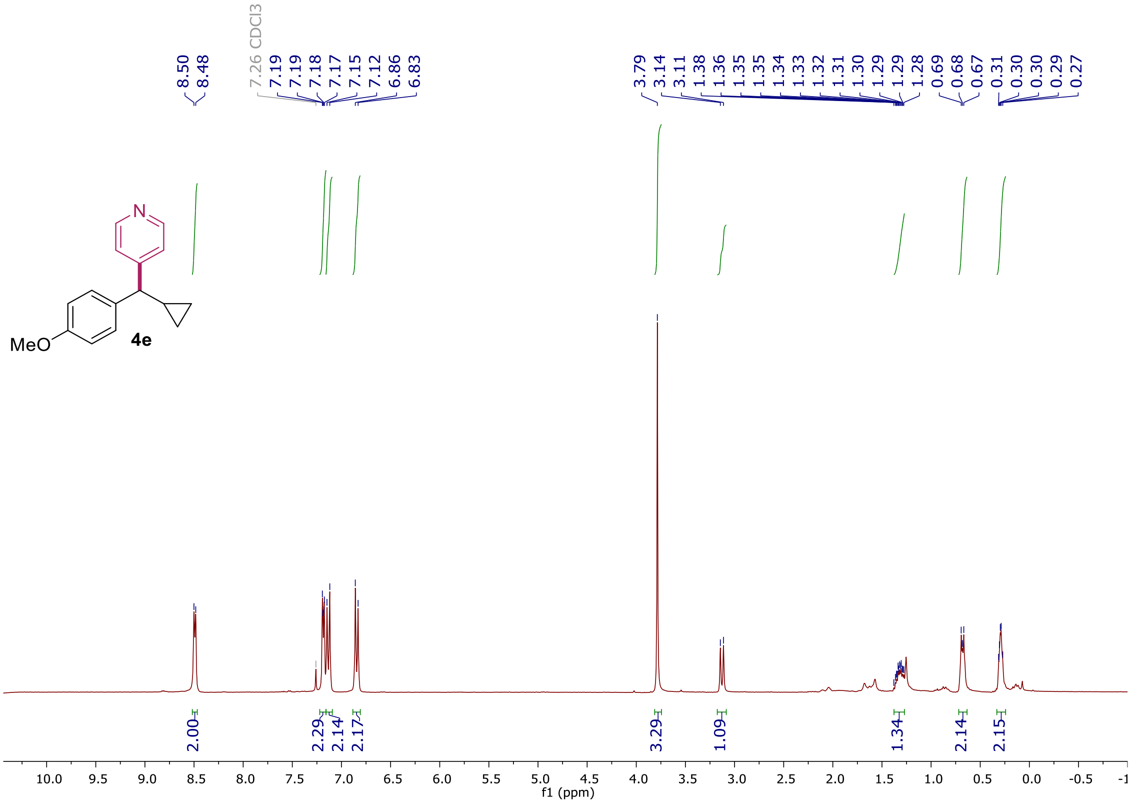

¹³C-NMR (75 MHz, $CDCl_3$) of compound **4e**

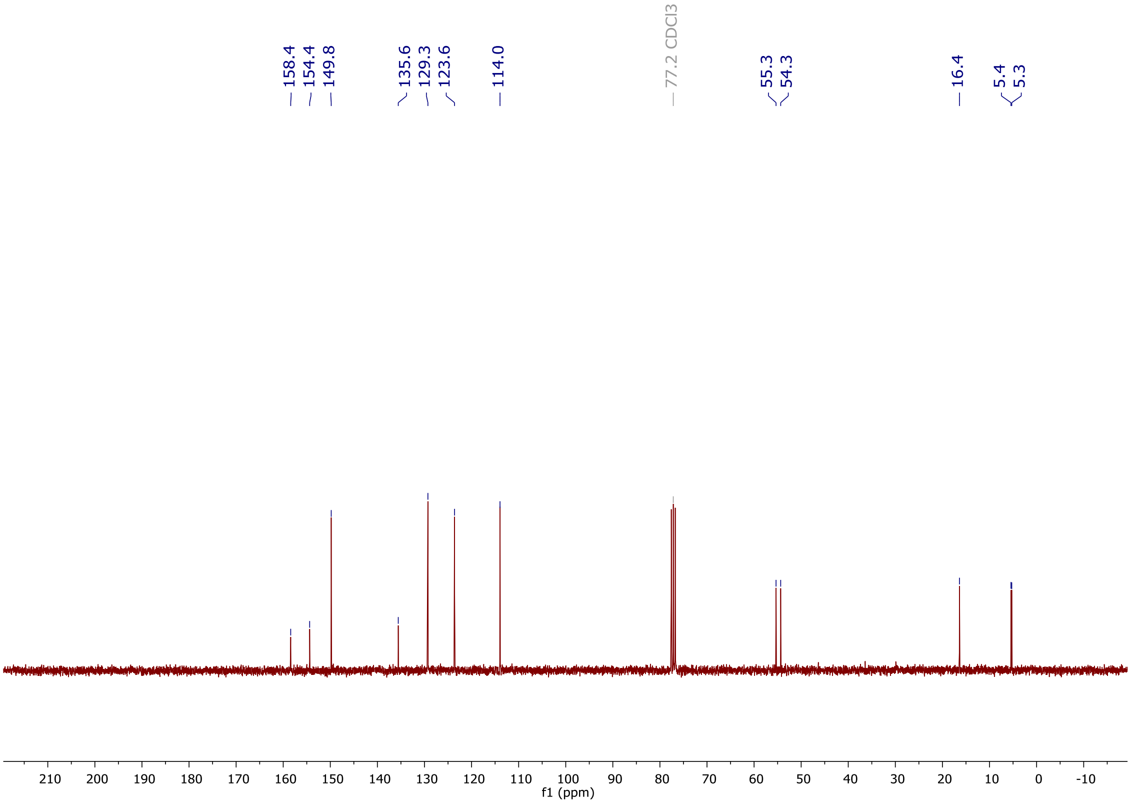

^{1}H-NMR (300 MHz, $CDCl_3$) of compound **4f**

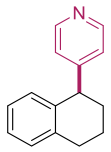

4f

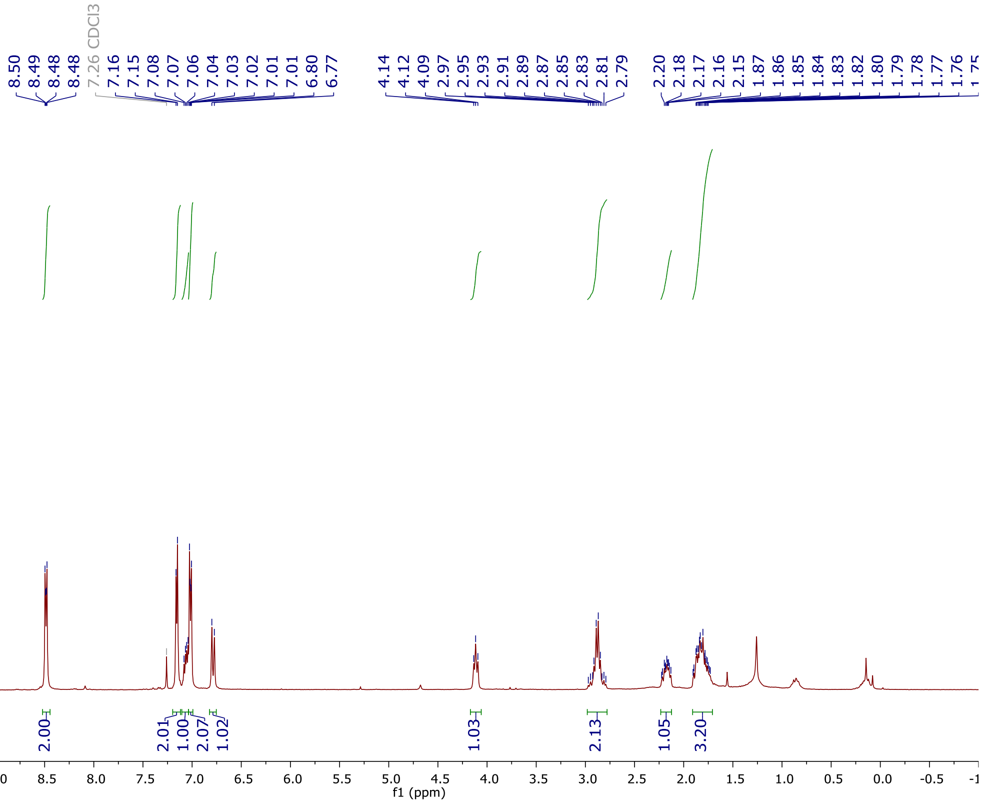

^{1}H-NMR (300 MHz, $CDCl_3$) of compound **4g**

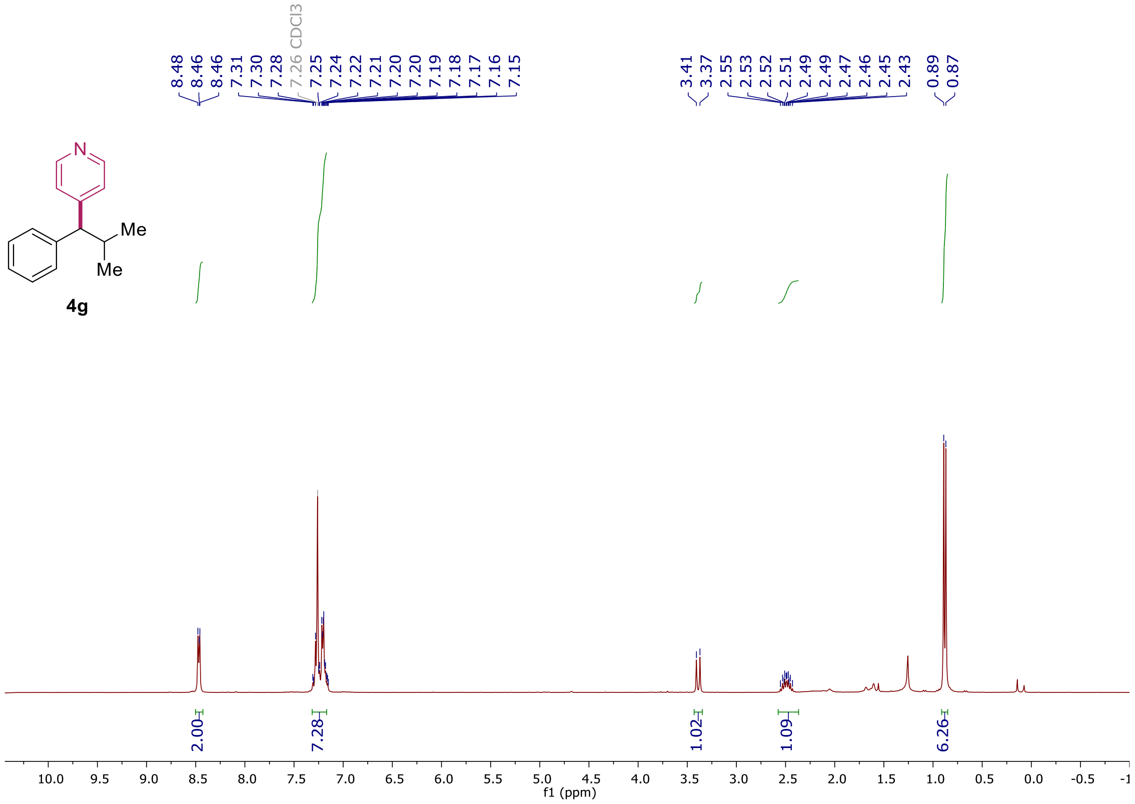

^{1}H-NMR (300 MHz, $CDCl_3$) of compound **4h**

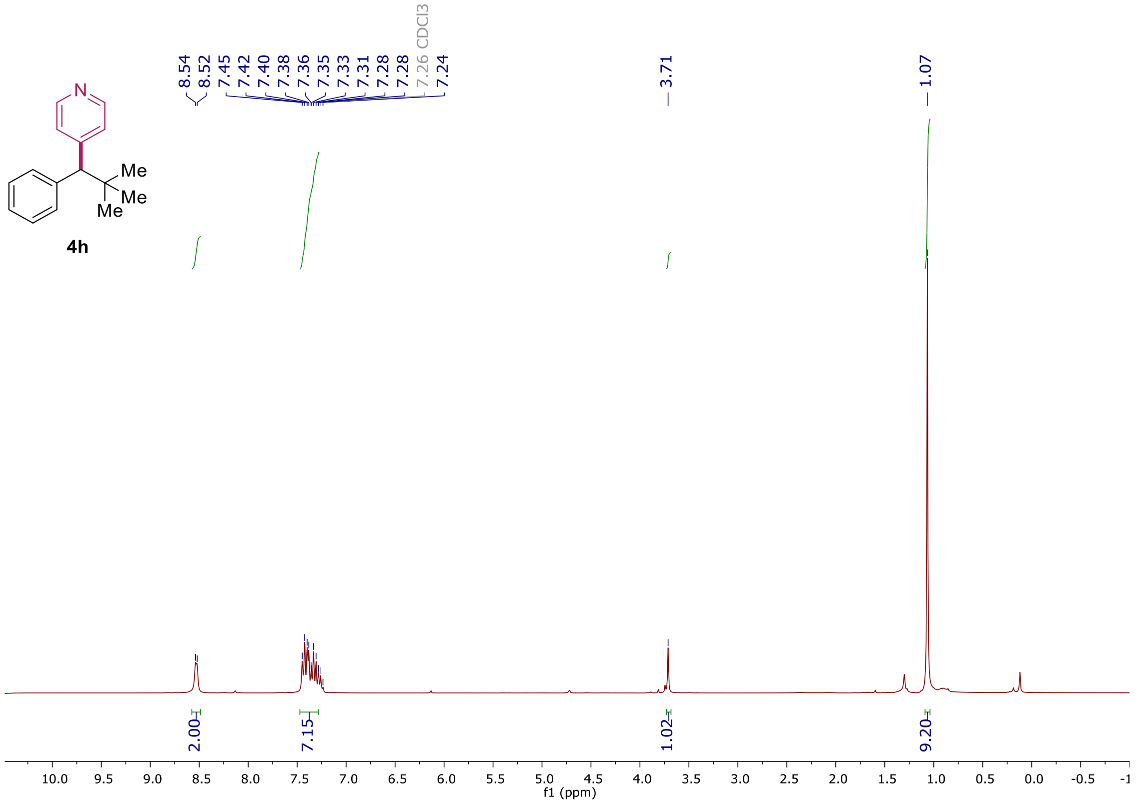

^{13}C-NMR (75 MHz, $CDCl_3$) of compound **4h**

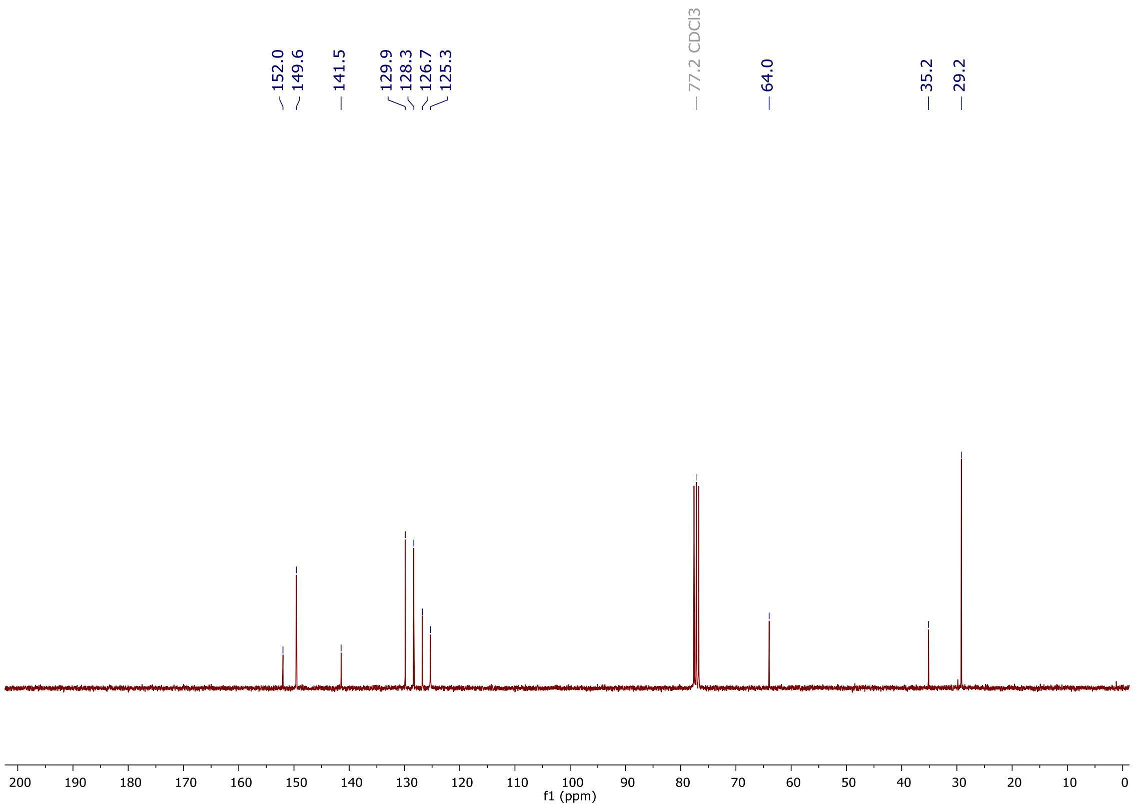

^{1}H-NMR (300 MHz, $CDCl_3$) of compound **4i**

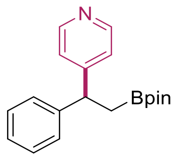

4i

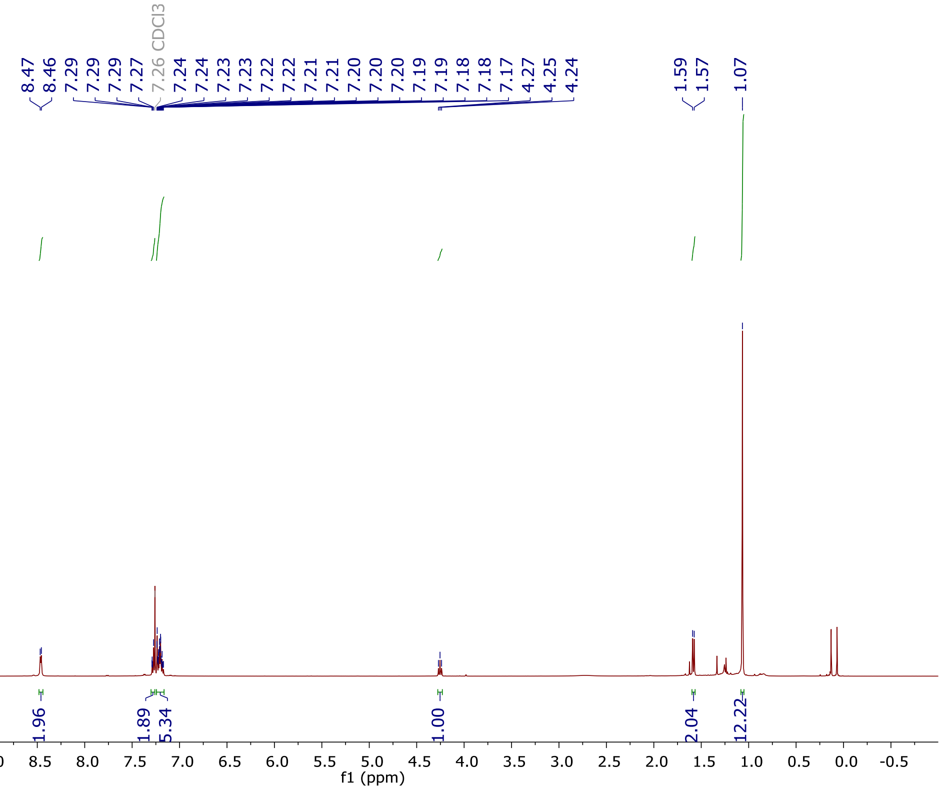

[1]**H-NMR** (300 MHz, $CDCl_3$) of compound **4j**

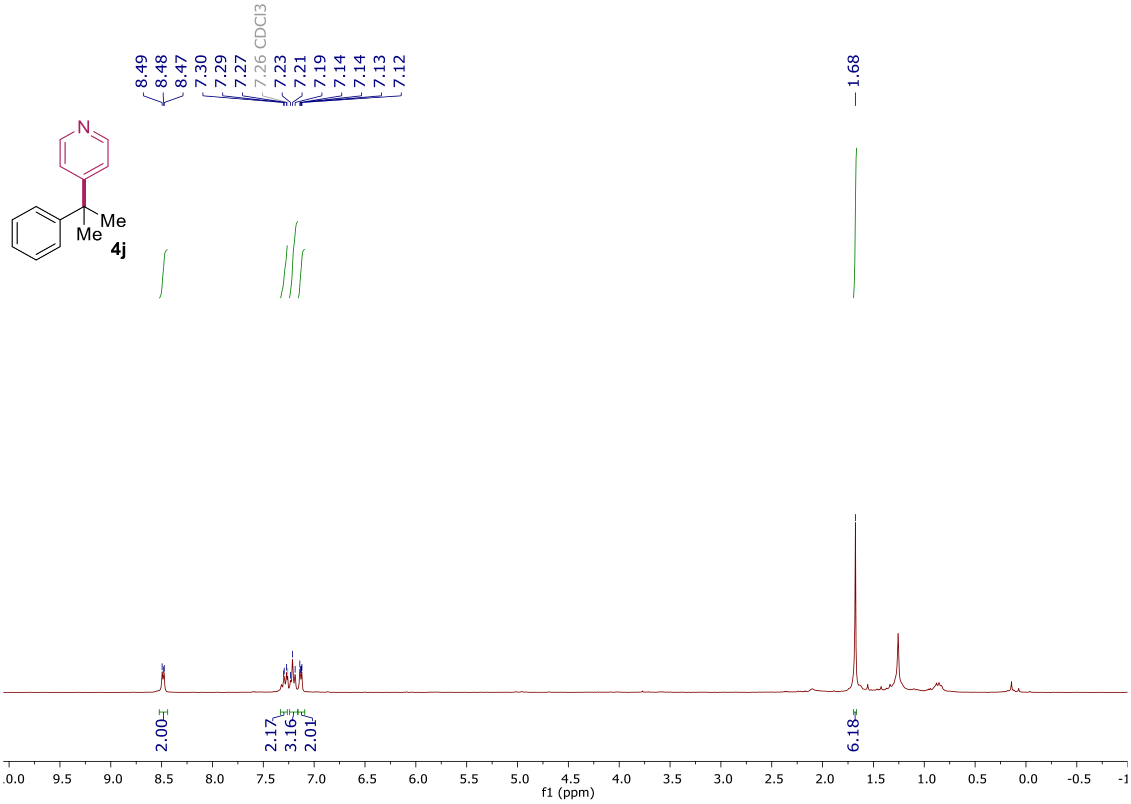

^{1}**H-NMR** (300 MHz, $CDCl_3$) of compound **4k**

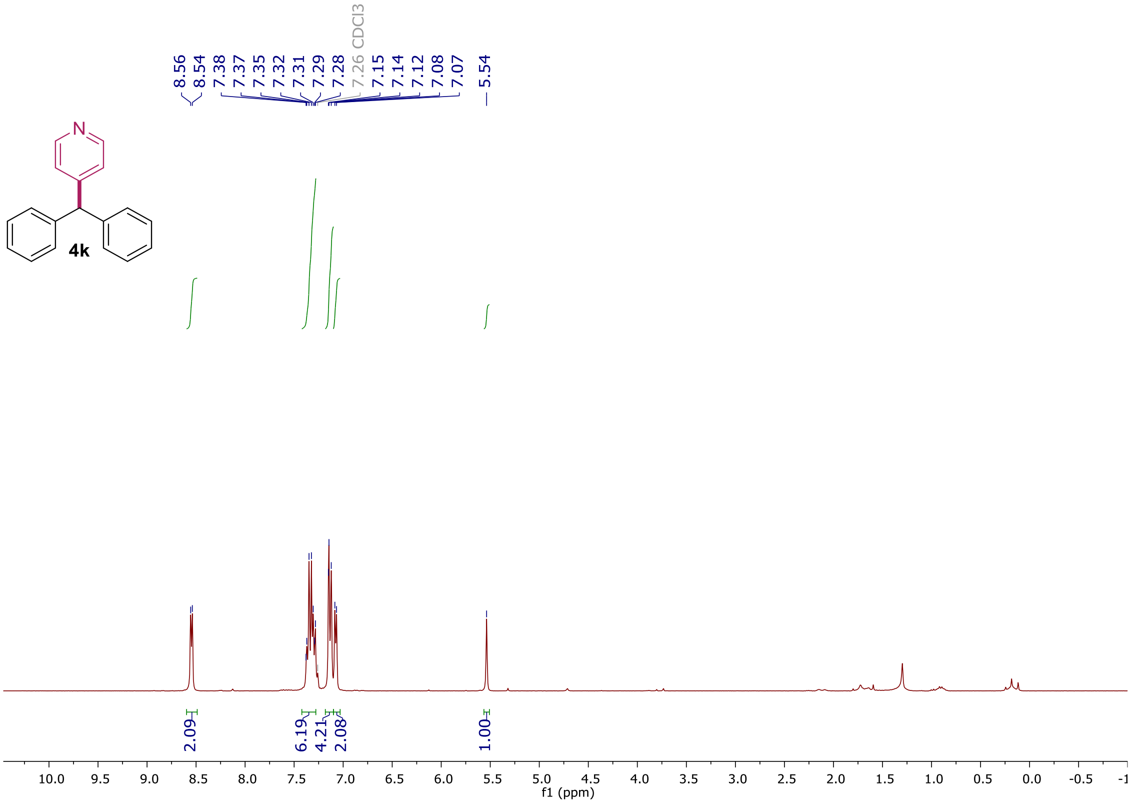

^{1}H-NMR (300 MHz, $CDCl_3$) of compound **4l**

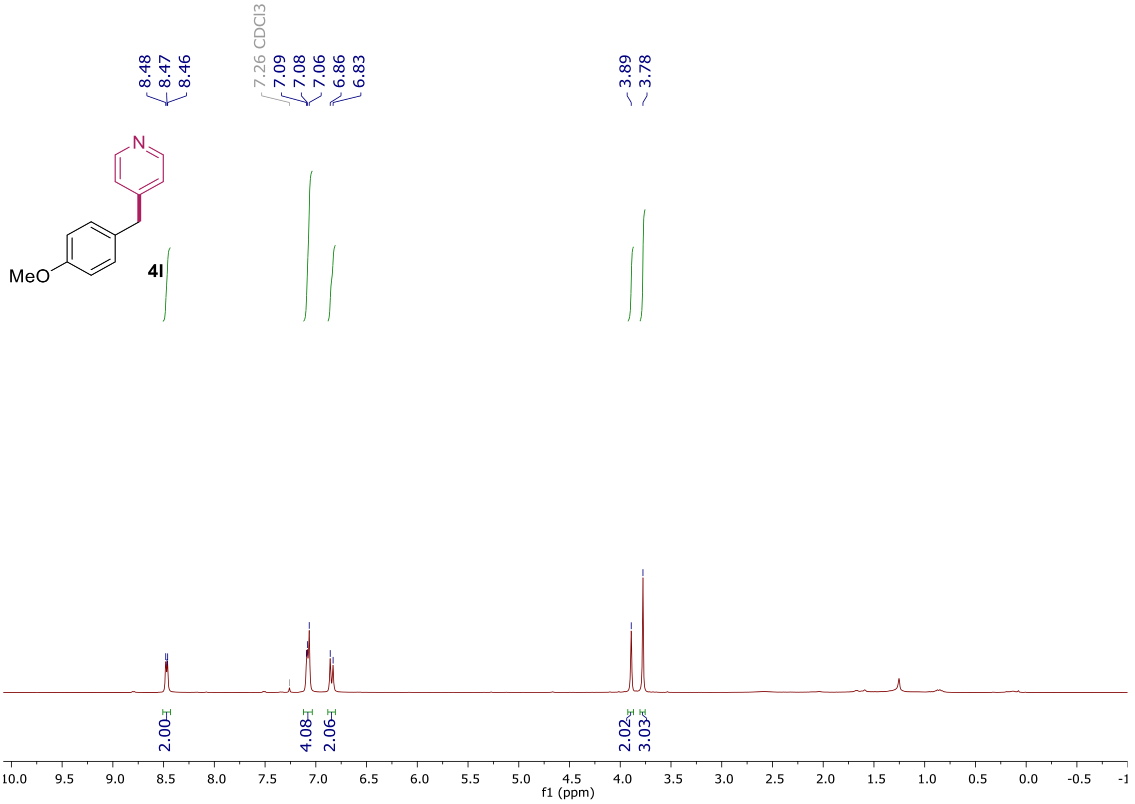

^{1}H-NMR (300 MHz, $CDCl_3$) of compound **4m**

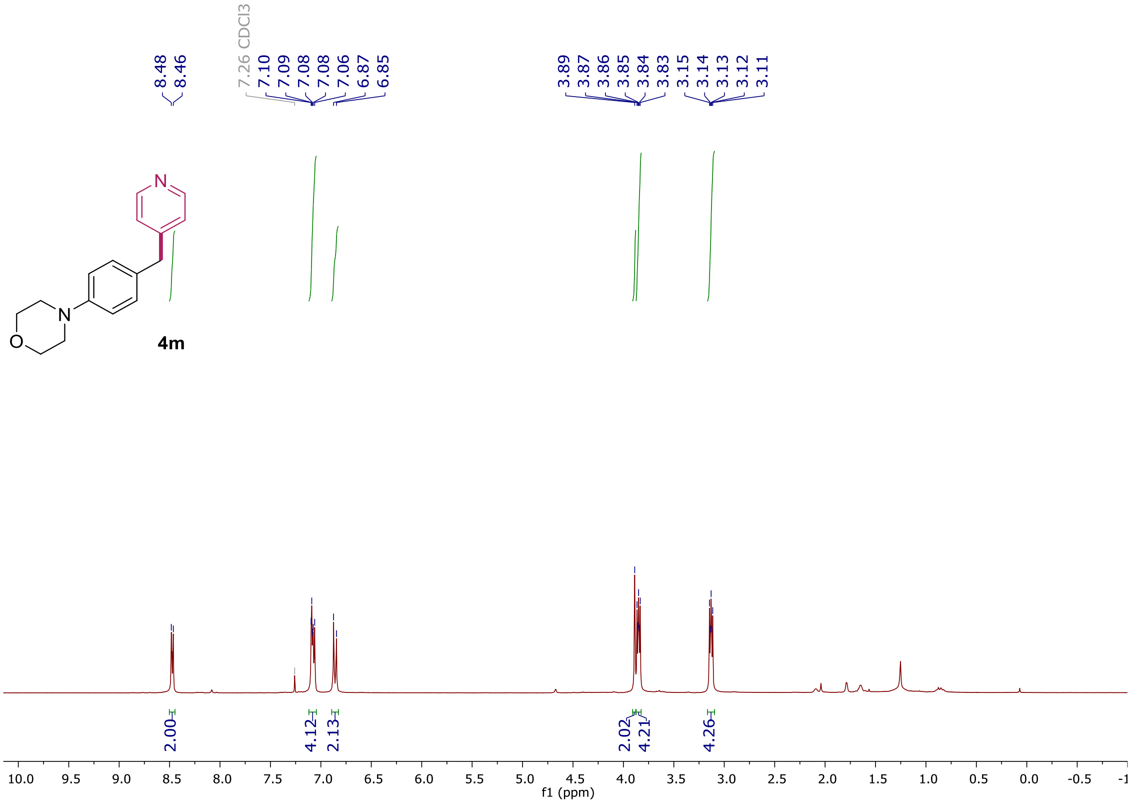

^{13}C-NMR (75 MHz, $CDCl_3$) of compound **4m**

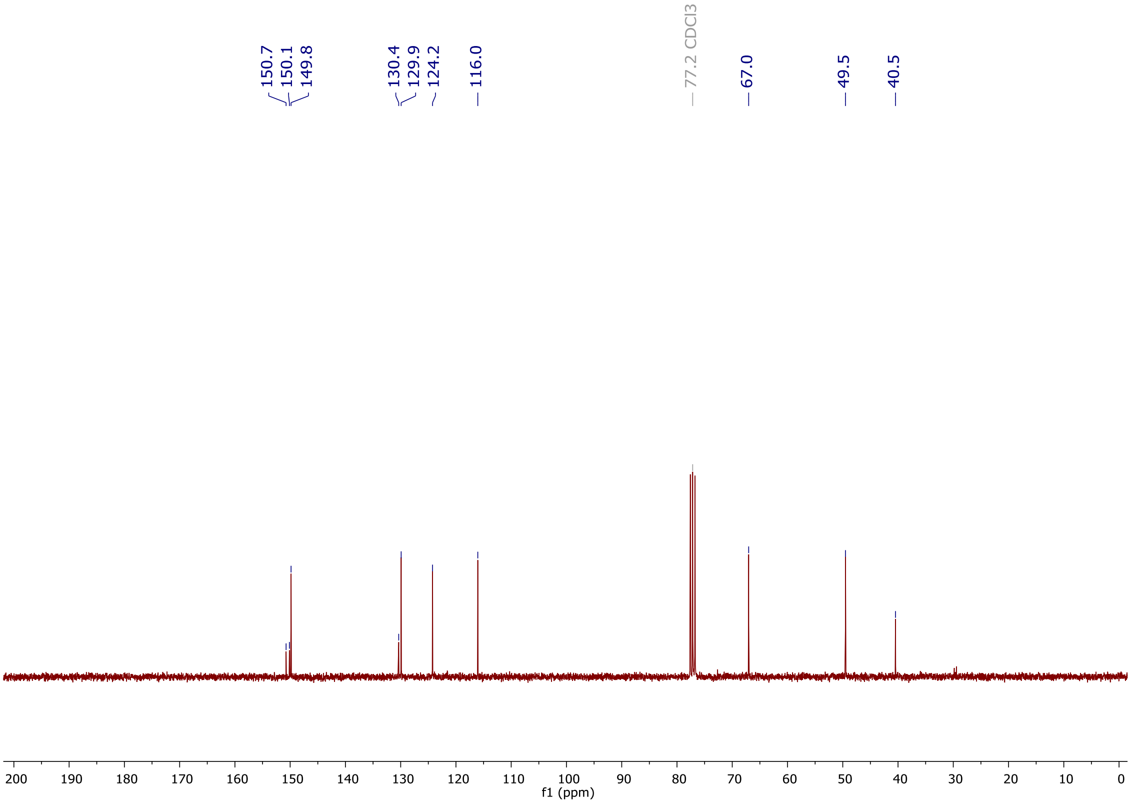

[1]**H-NMR** (300 MHz, $CDCl_3$) of compound **4n**

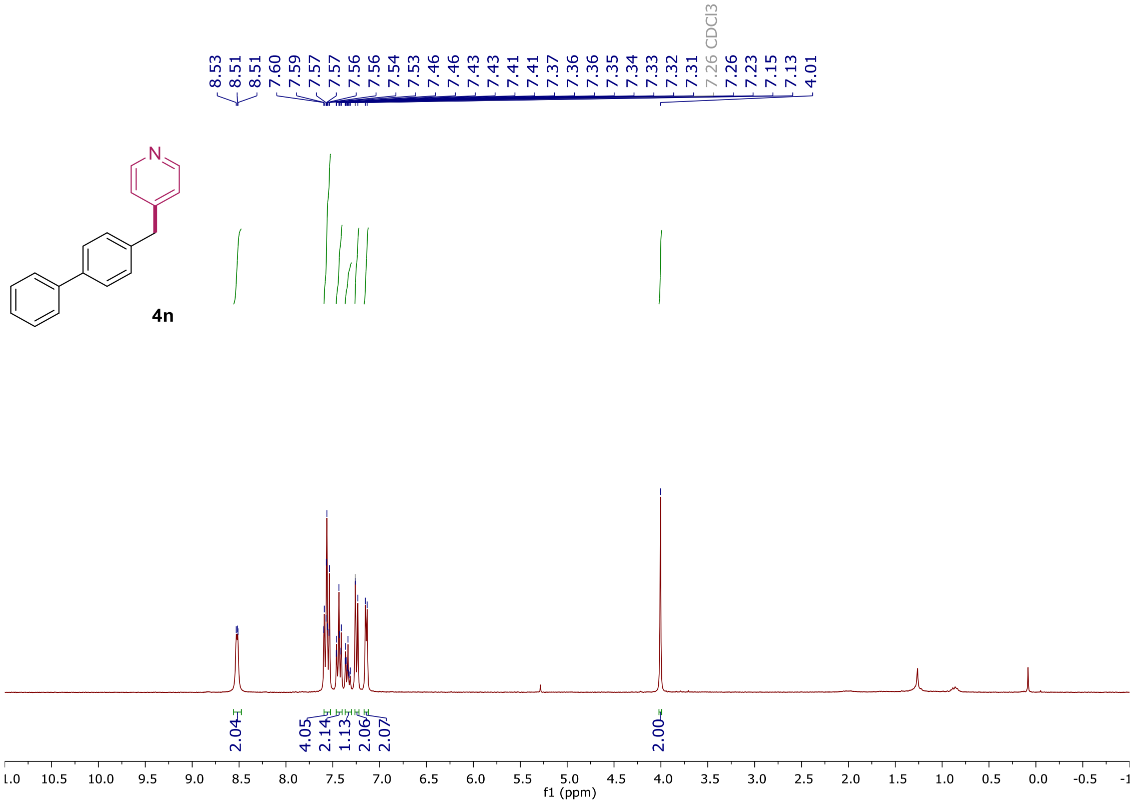

^{1}H-NMR (300 MHz, $CDCl_3$) of compound **4o**

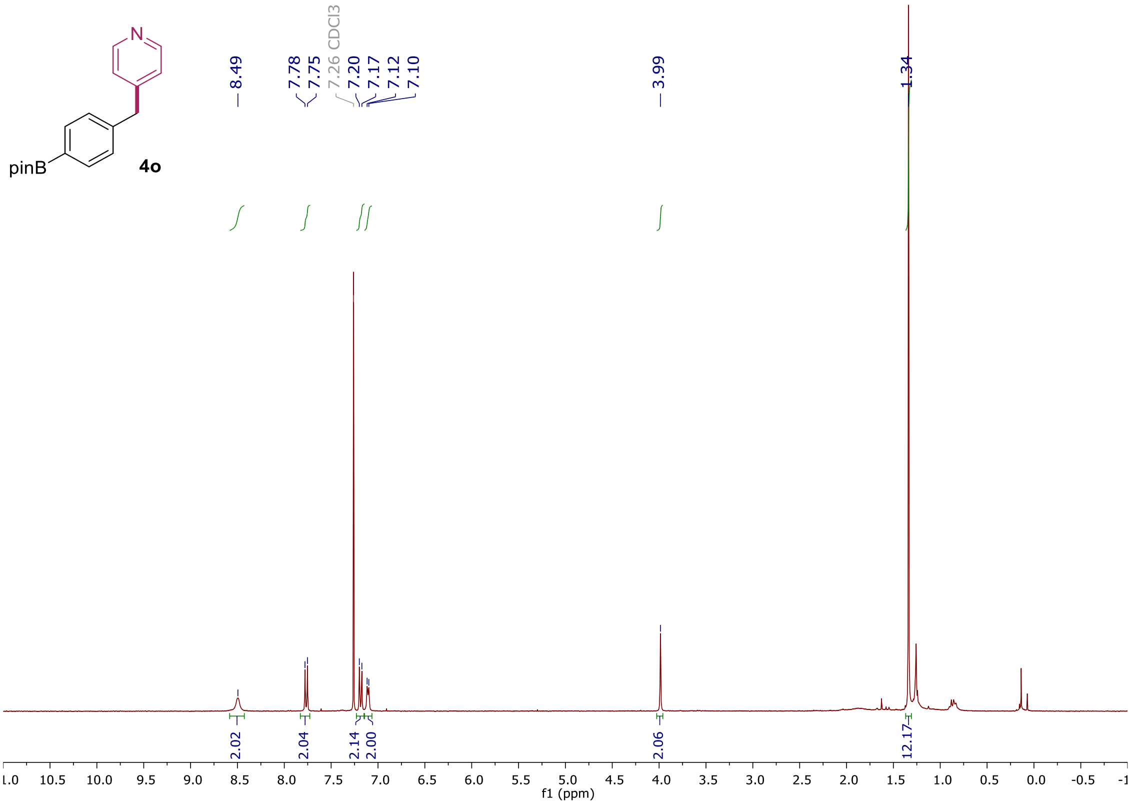

^{13}C-NMR (75 MHz, $CDCl_3$) of compound **4o**

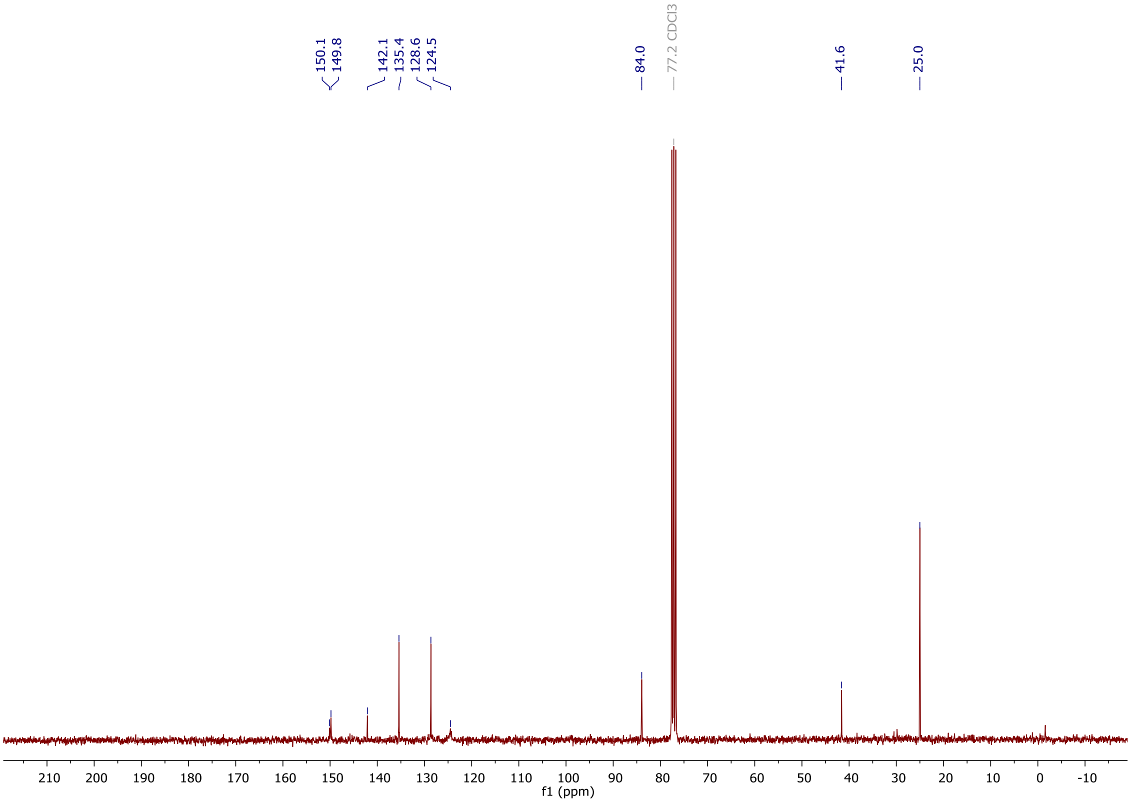

^{11}B-NMR (160 MHz, $CDCl_3$) of compound **4o**

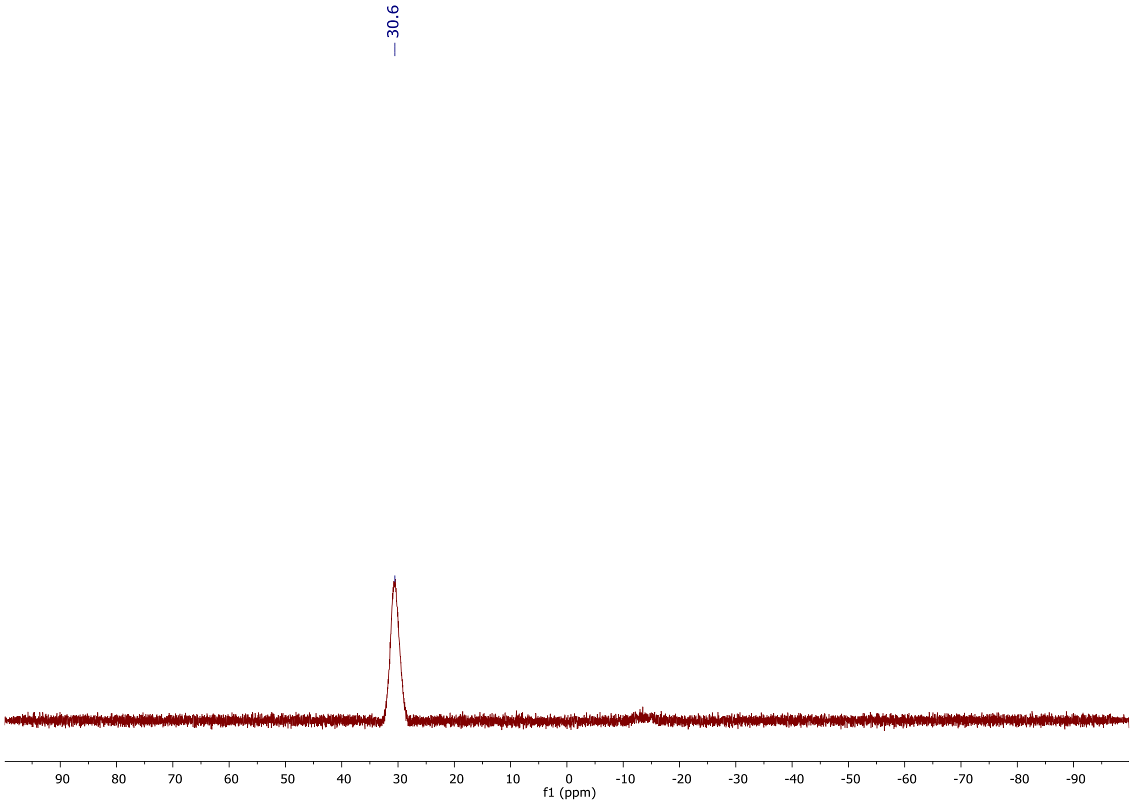

^{1}H-NMR (300 MHz, $CDCl_3$) of compound **4p**

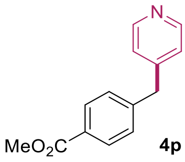

4p

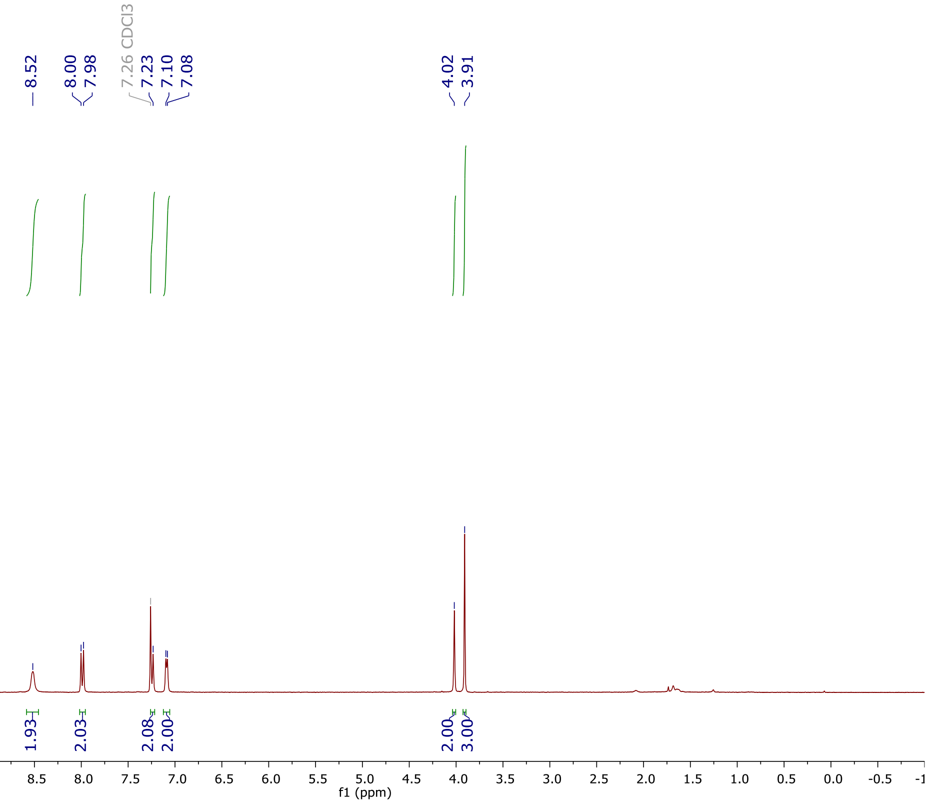

^{13}C-NMR (75 MHz, $CDCl_3$) of compound **4p**

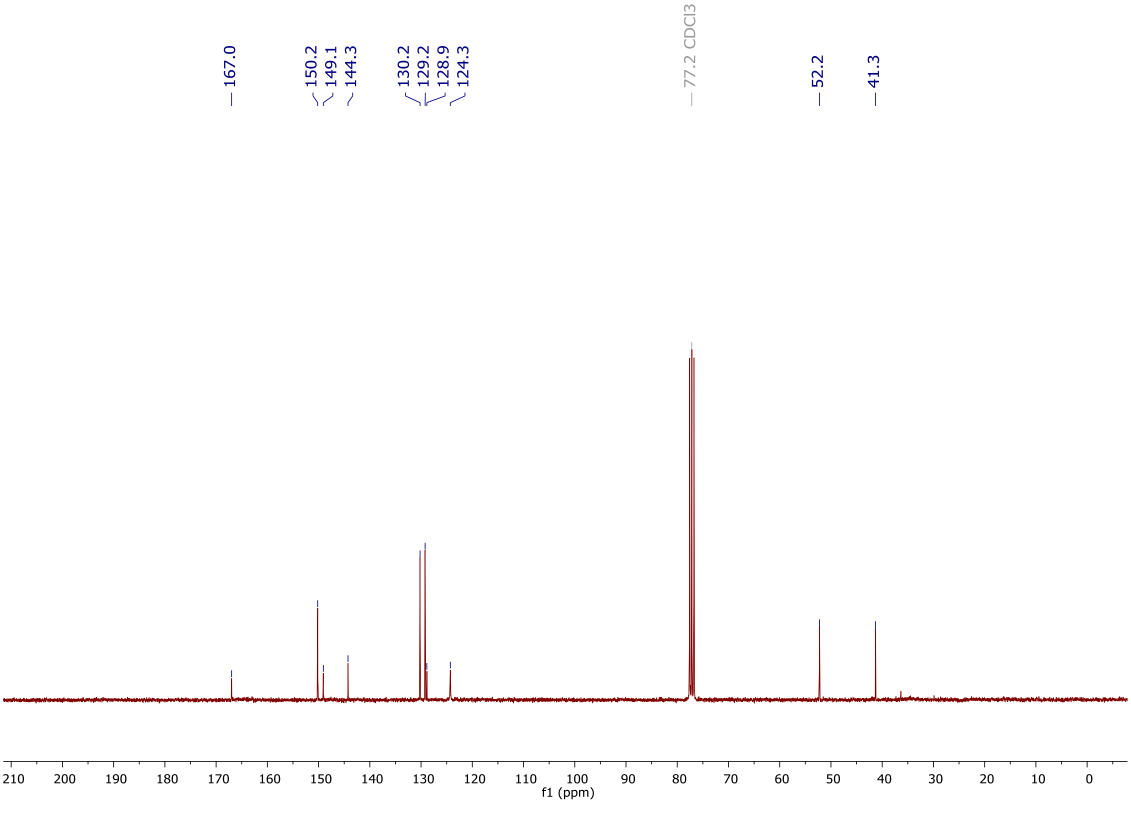

^{1}H-NMR (300 MHz, $CDCl_3$) of compound **4q**

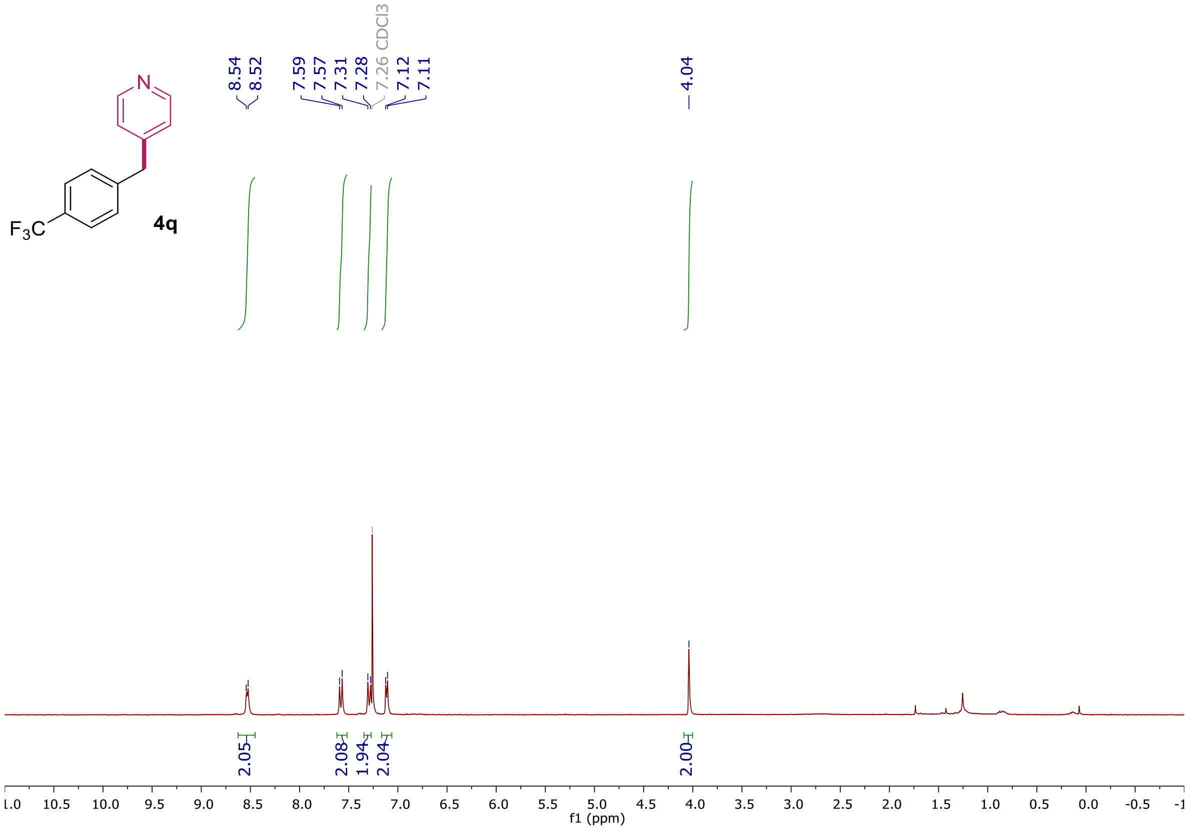

^{1}H-NMR (300 MHz, $CDCl_3$) of compound **4r**

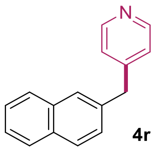
4r

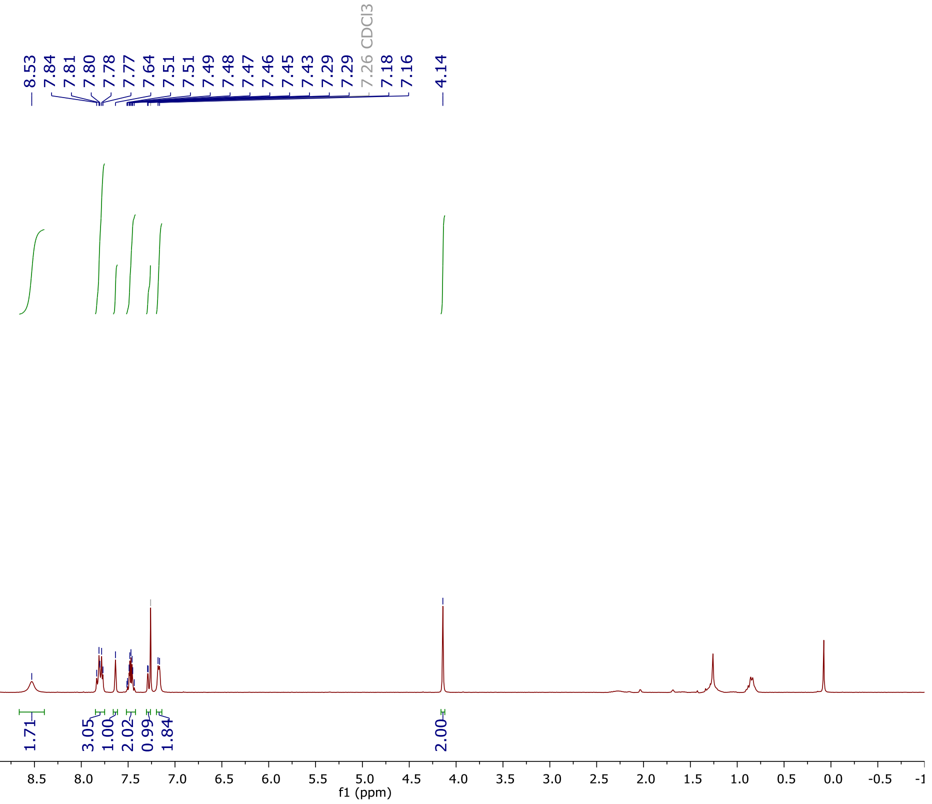

^{1}H-NMR (300 MHz, $CDCl_3$) of compound **4s**

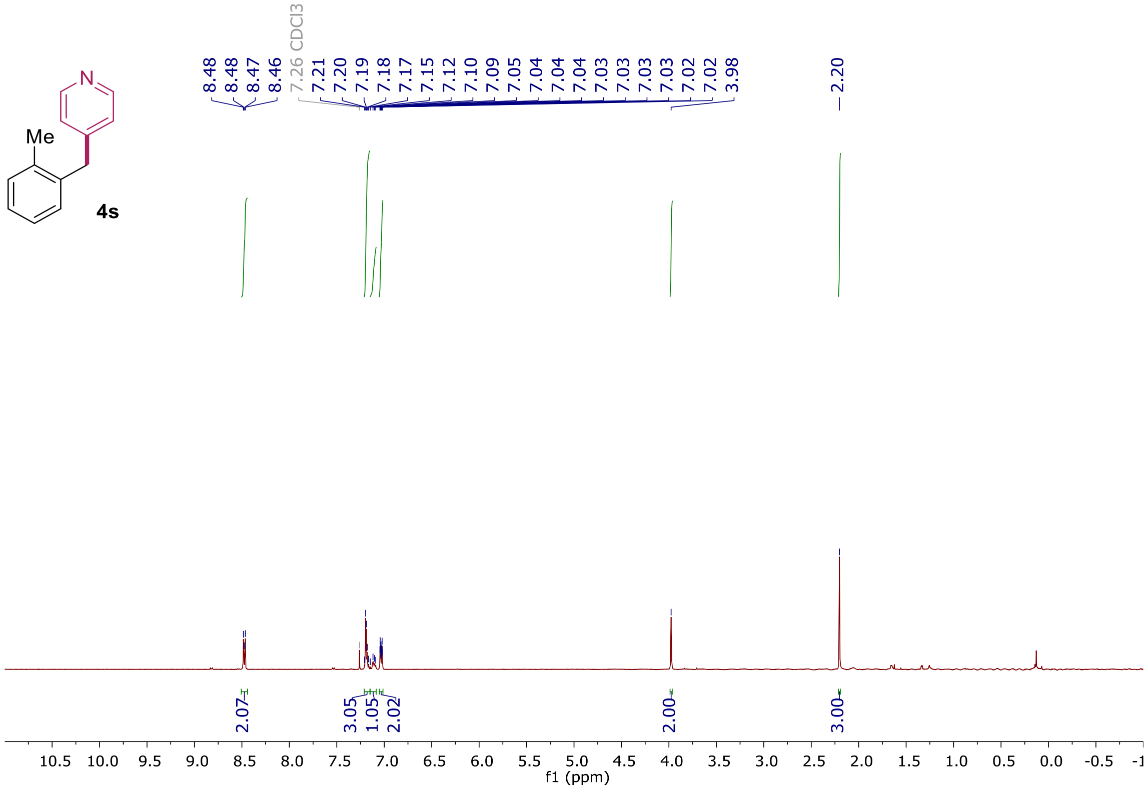

[1]**H-NMR** (300 MHz, $CDCl_3$) of compound **4t**

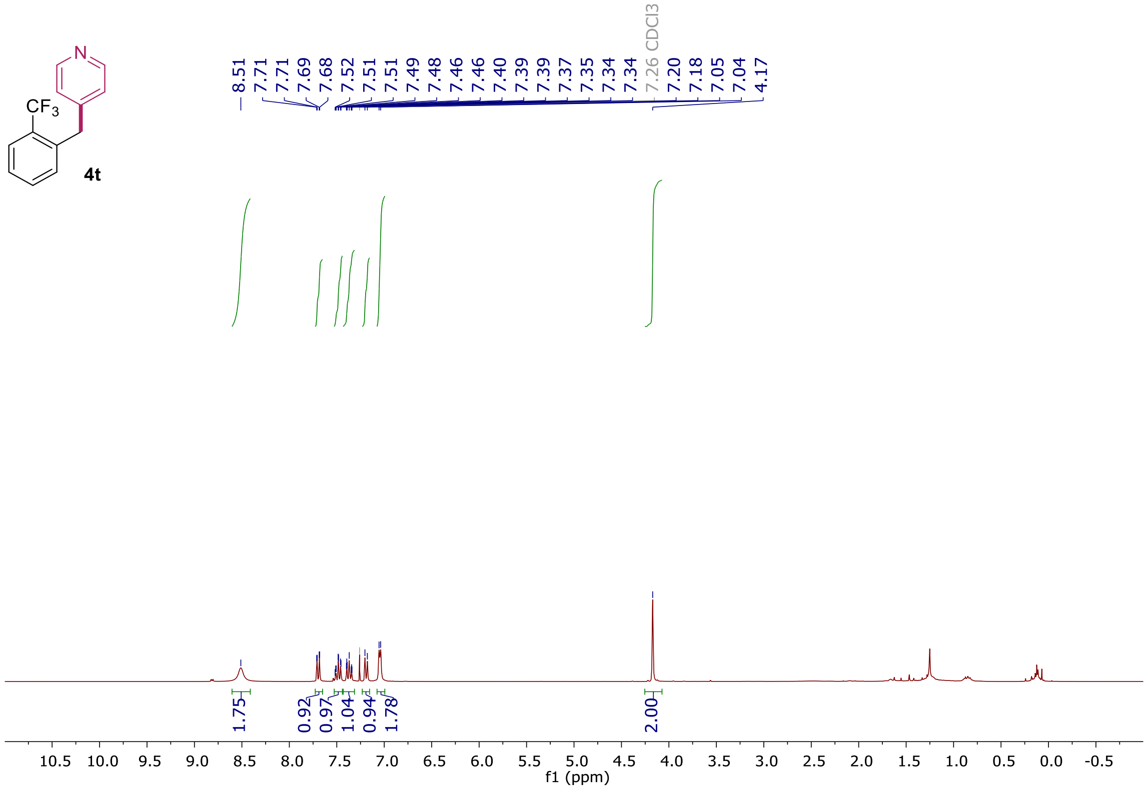

[13]**C-NMR** (75 MHz, $CDCl_3$) of compound **4t**

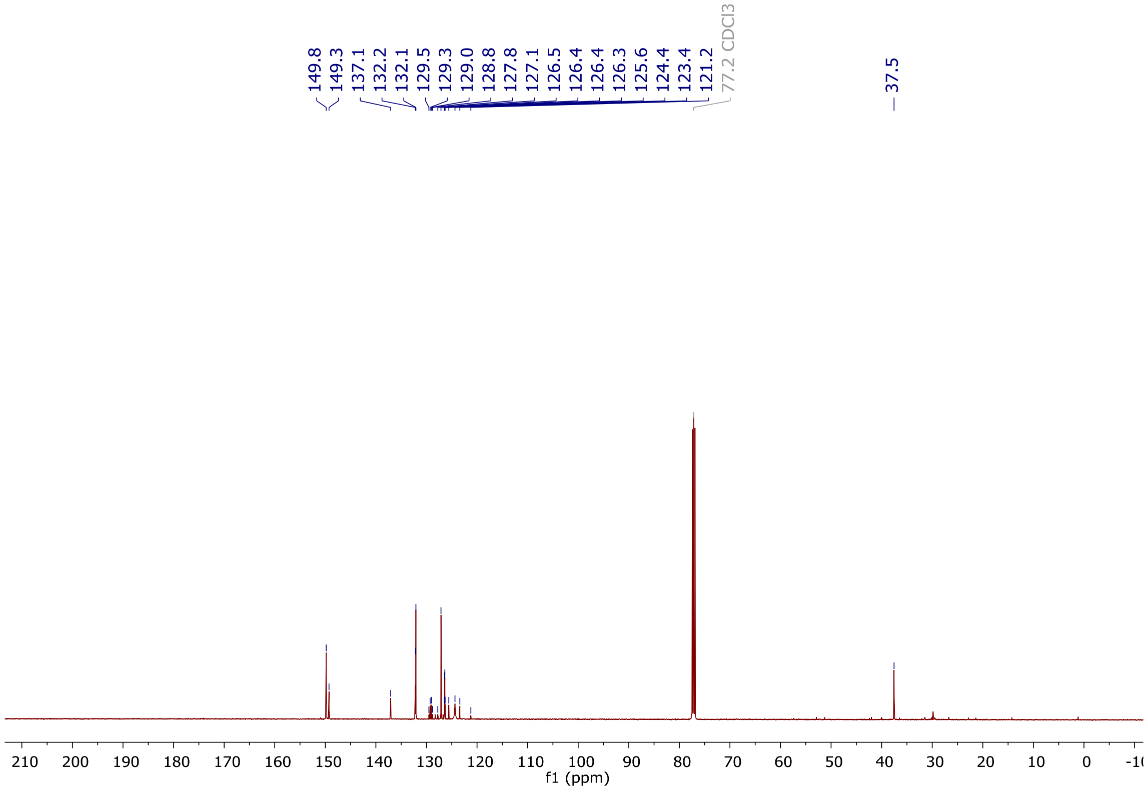

^{19}F-NMR (470 MHz, $CDCl_3$) of compound **4t**

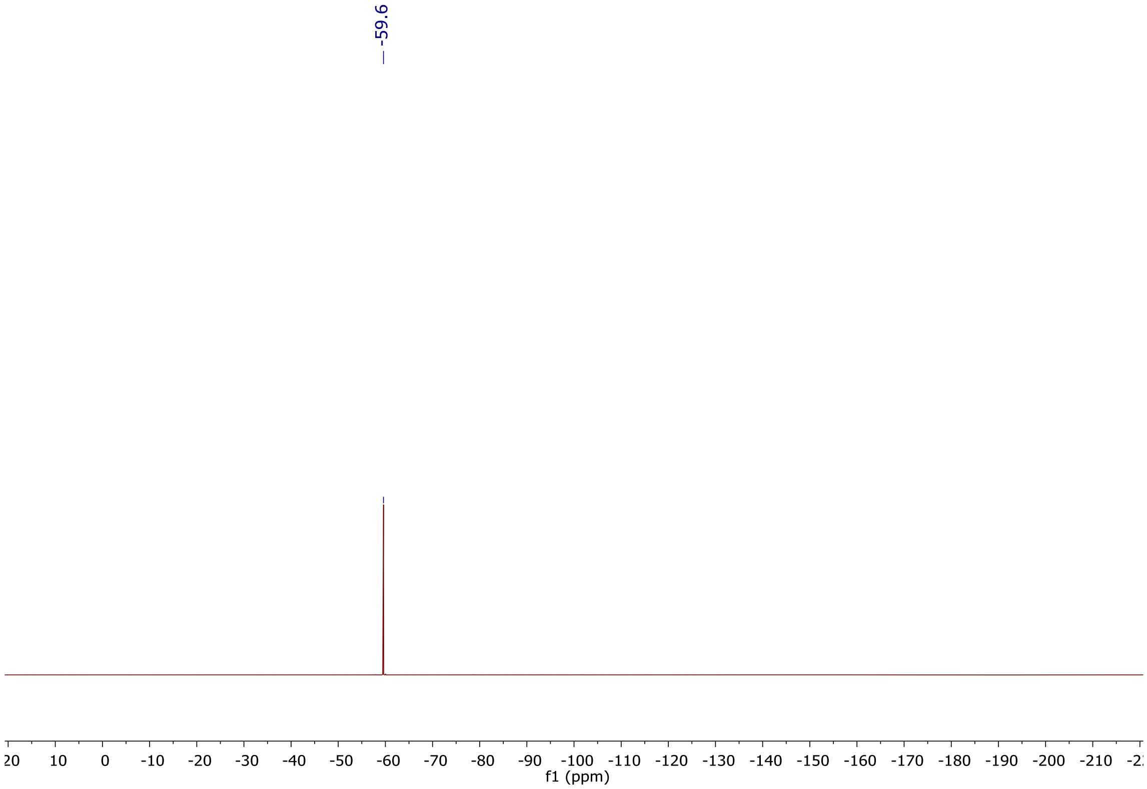

^{1}H-NMR (300 MHz, $CDCl_3$) of compound **4u**

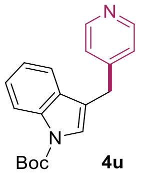

4u

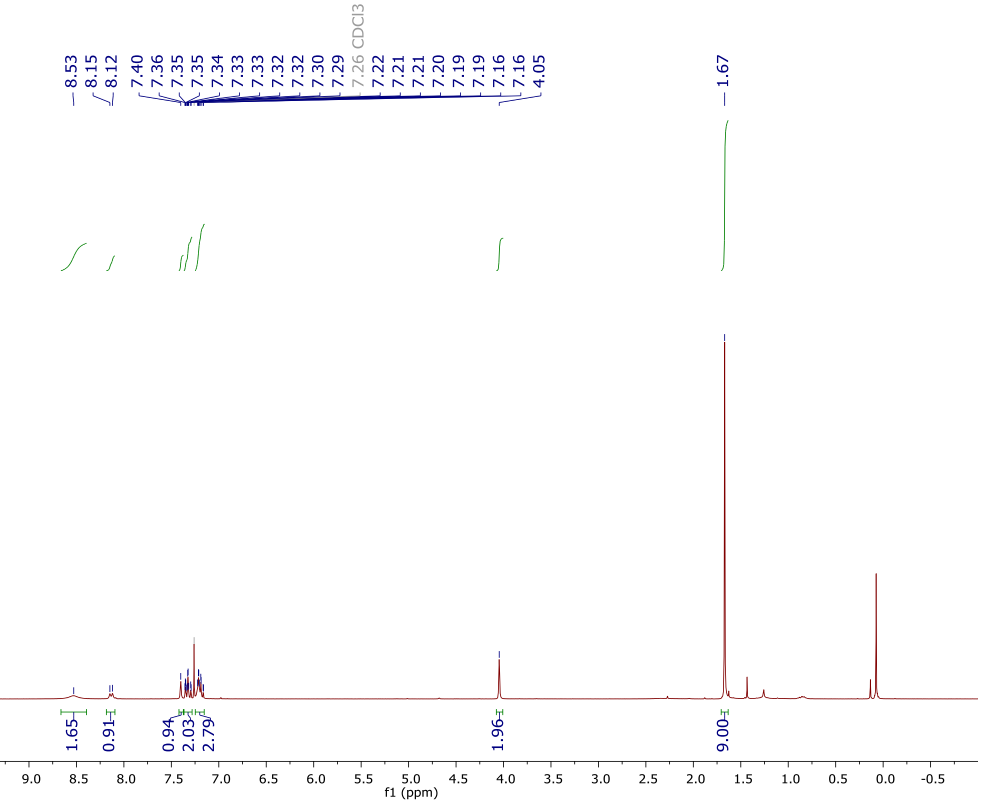

^{1}H-NMR (300 MHz, $CDCl_3$) of compound **4v**

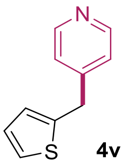

4v

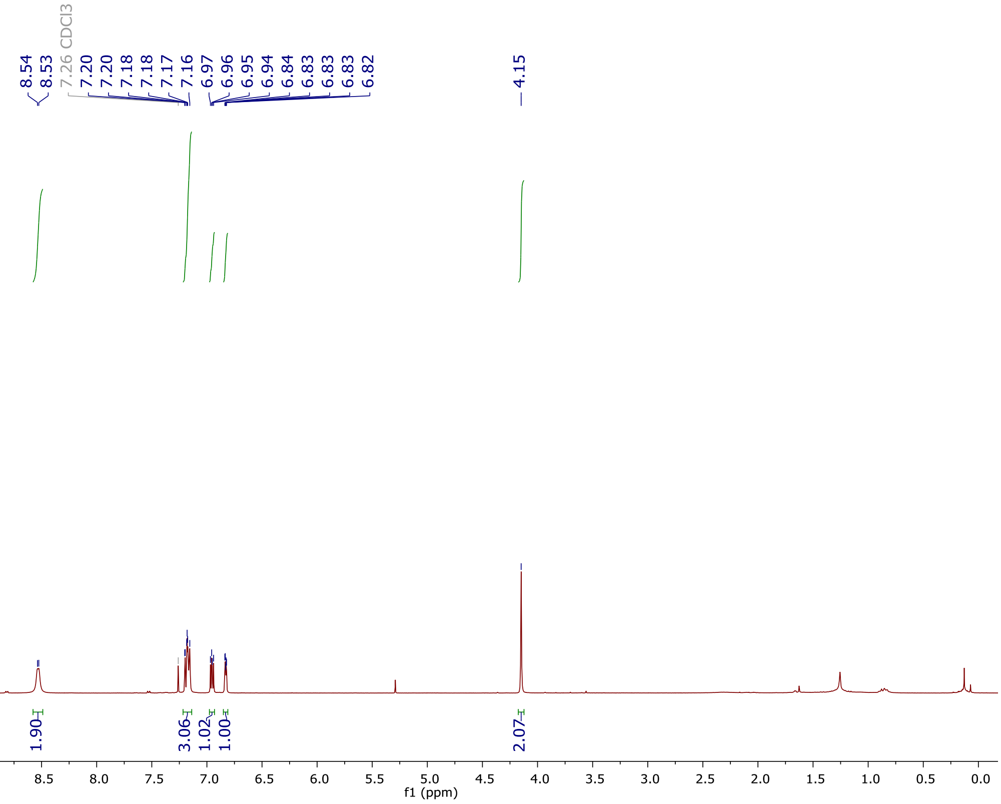

^{13}C-NMR (75 MHz, $CDCl_3$) of compound **4v**

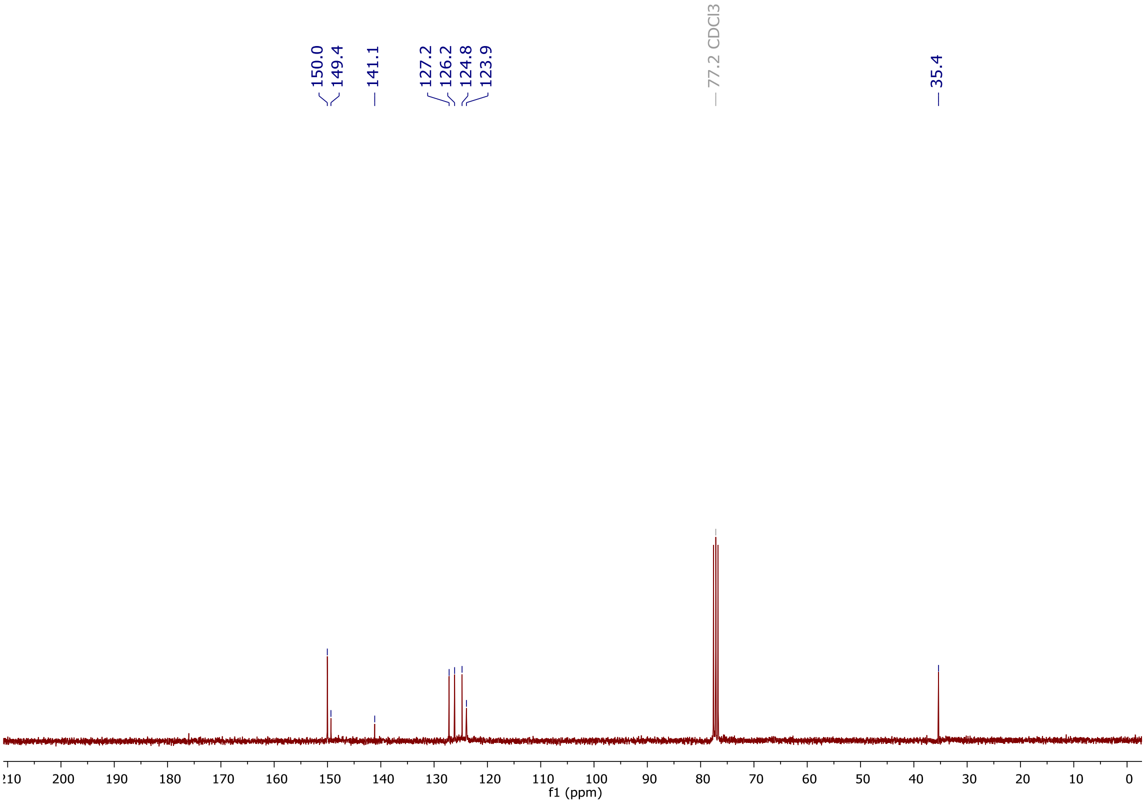

^{1}H-NMR (300 MHz, $CDCl_3$) of compound **4w**

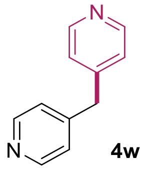

4w

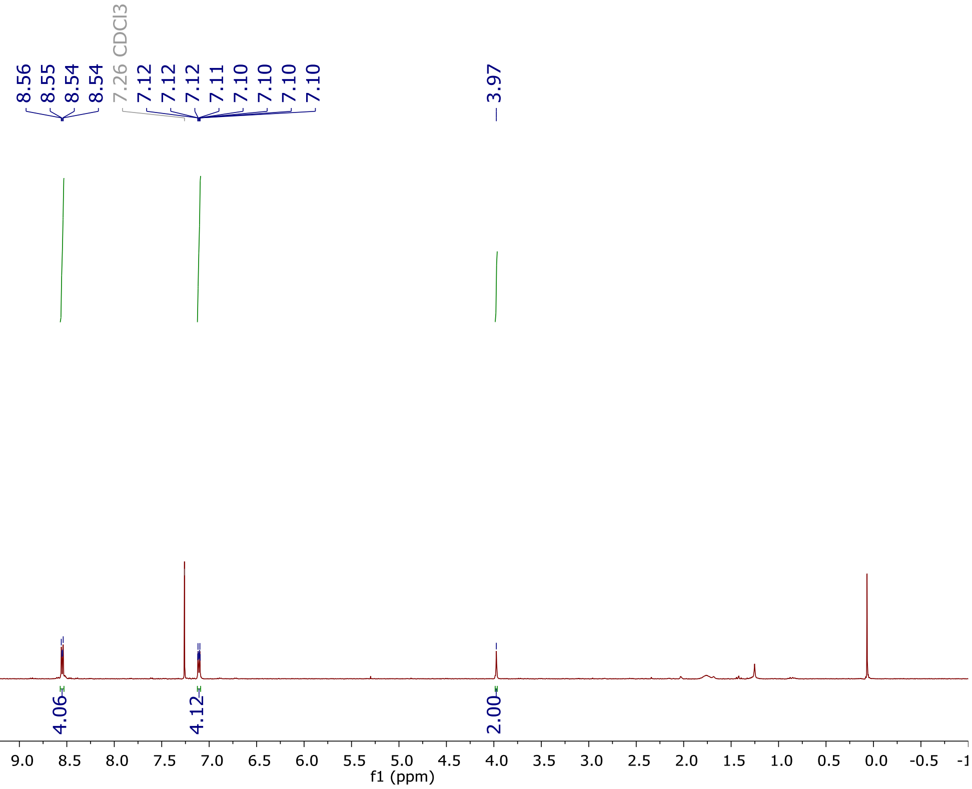

¹H-NMR (300 MHz, $CDCl_3$) of compound **4x**

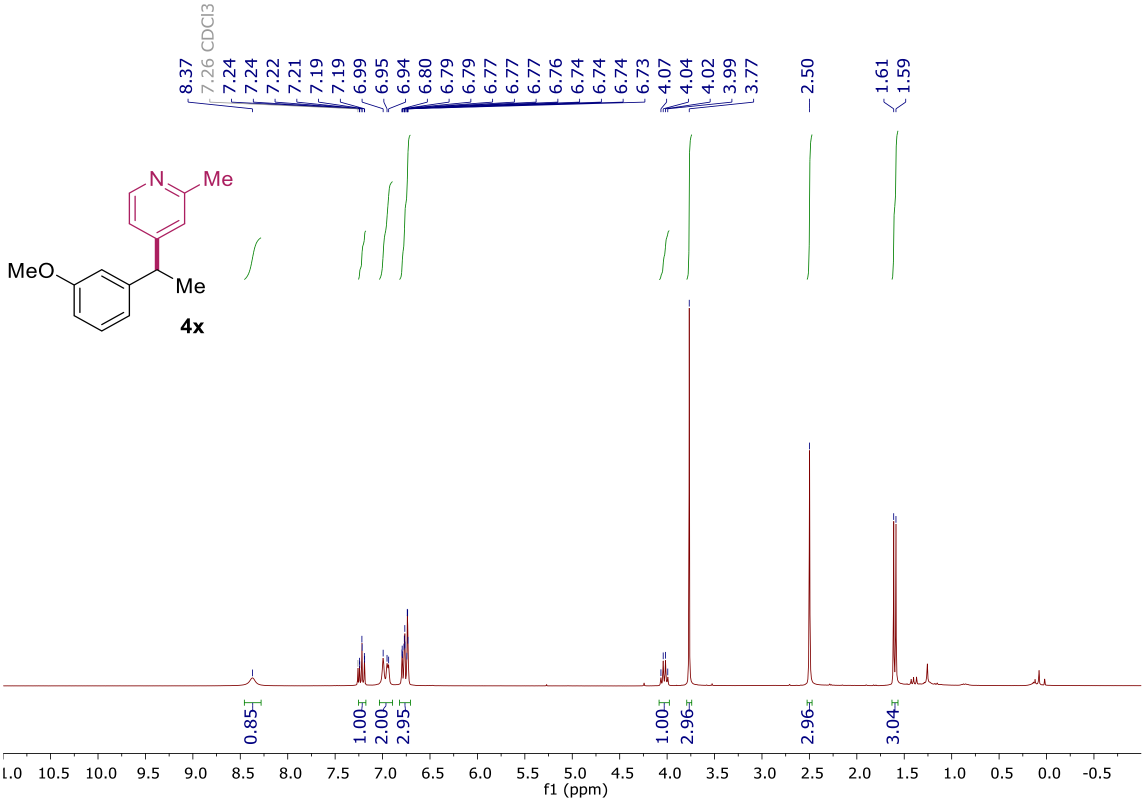

¹³C-NMR (75 MHz, $CDCl_3$) of compound **4x**

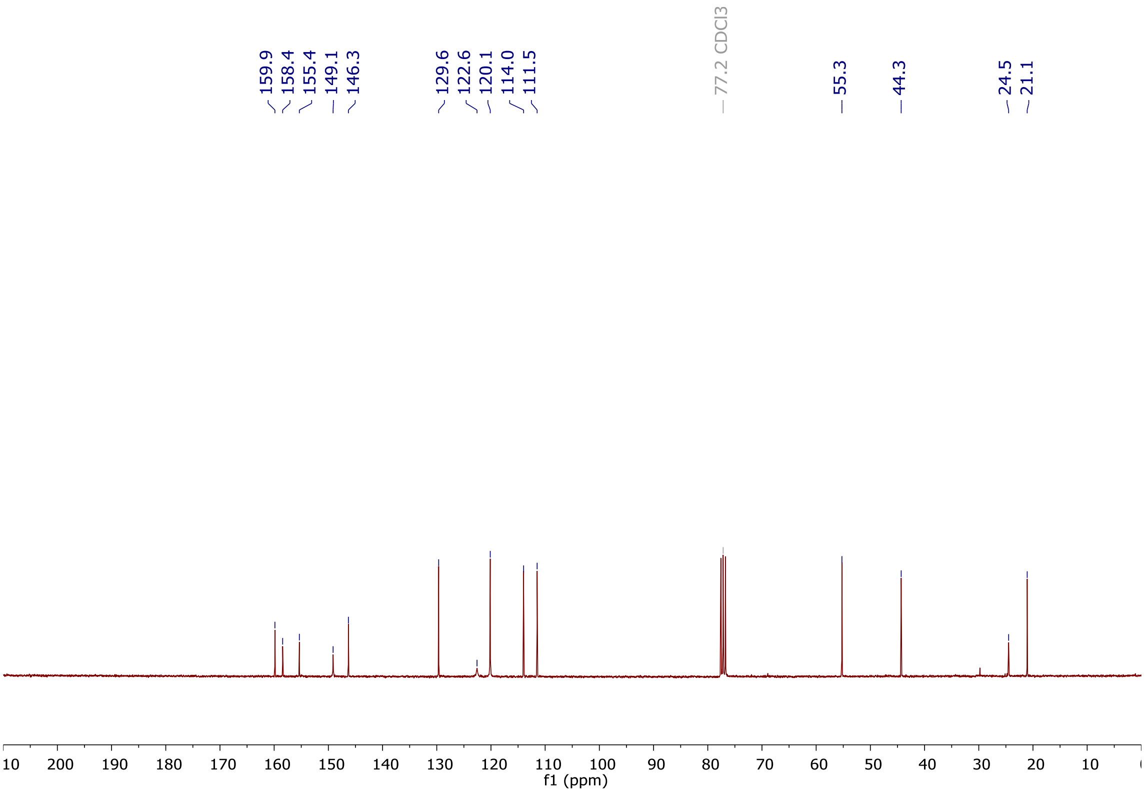

^{1}H-NMR (300 MHz, $CDCl_3$) of compound **4y**

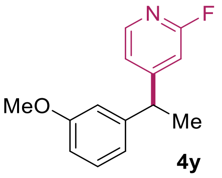

4y

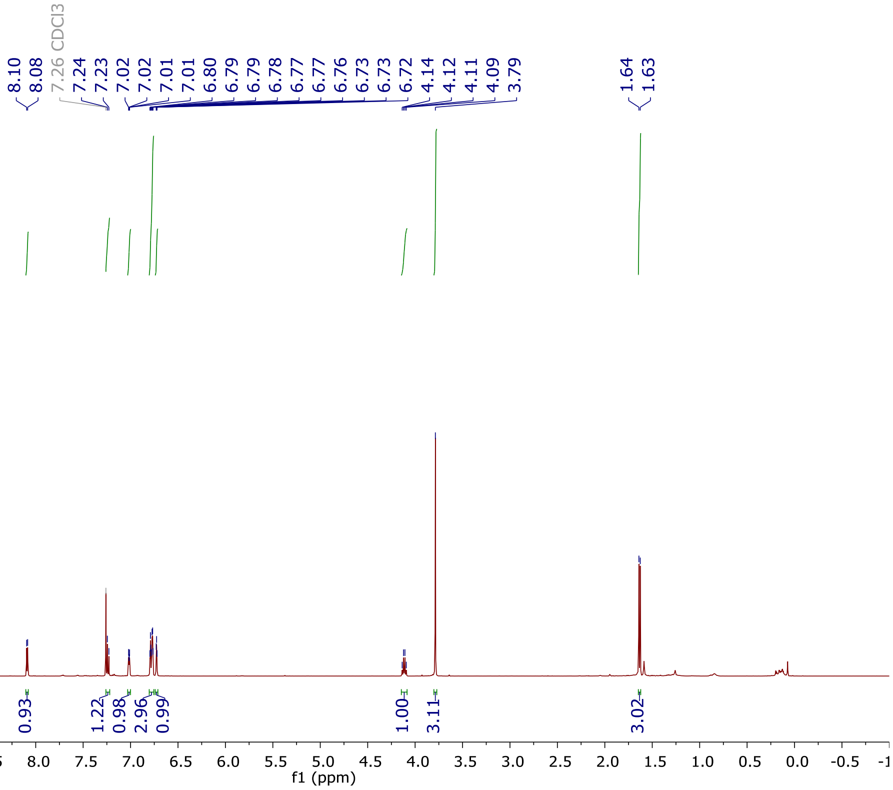

^{13}C-NMR (75 MHz, $CDCl_3$) of compound **4y**

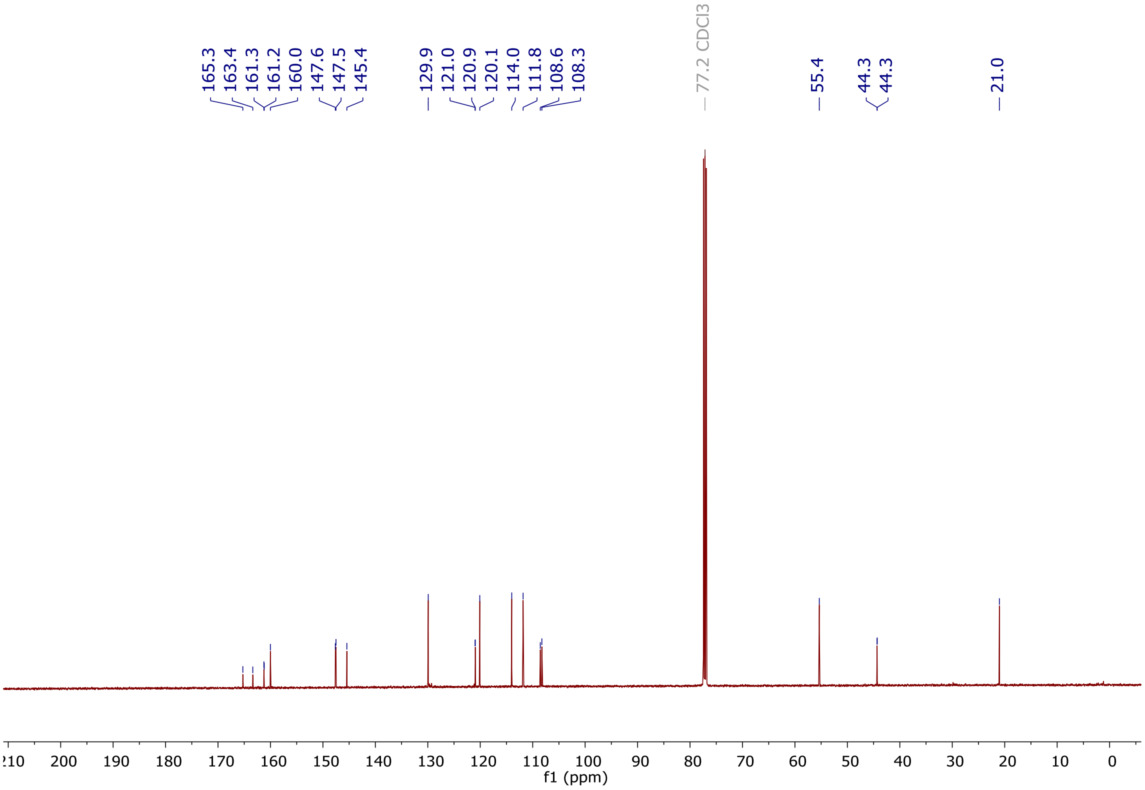

^{19}**F-NMR** (470 MHz, $CDCl_3$) of compound **4y**

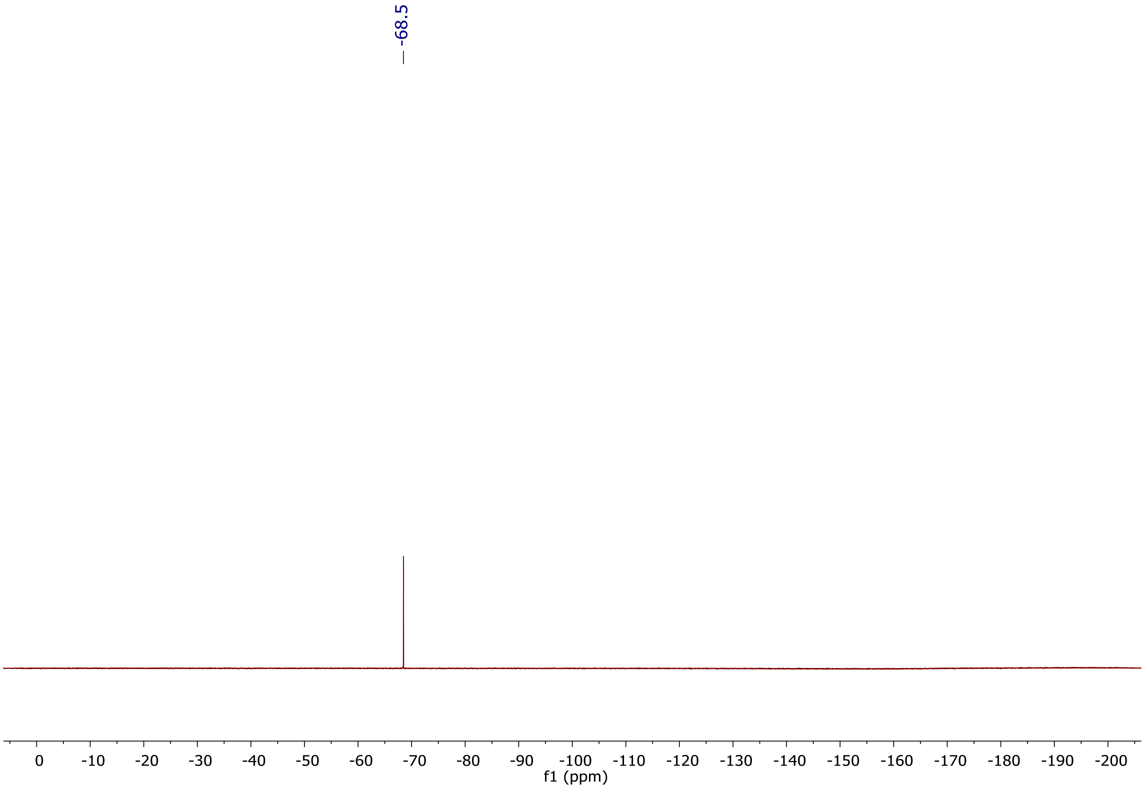

^{1}H-NMR (300 MHz, $CDCl_3$) of compound **4z**

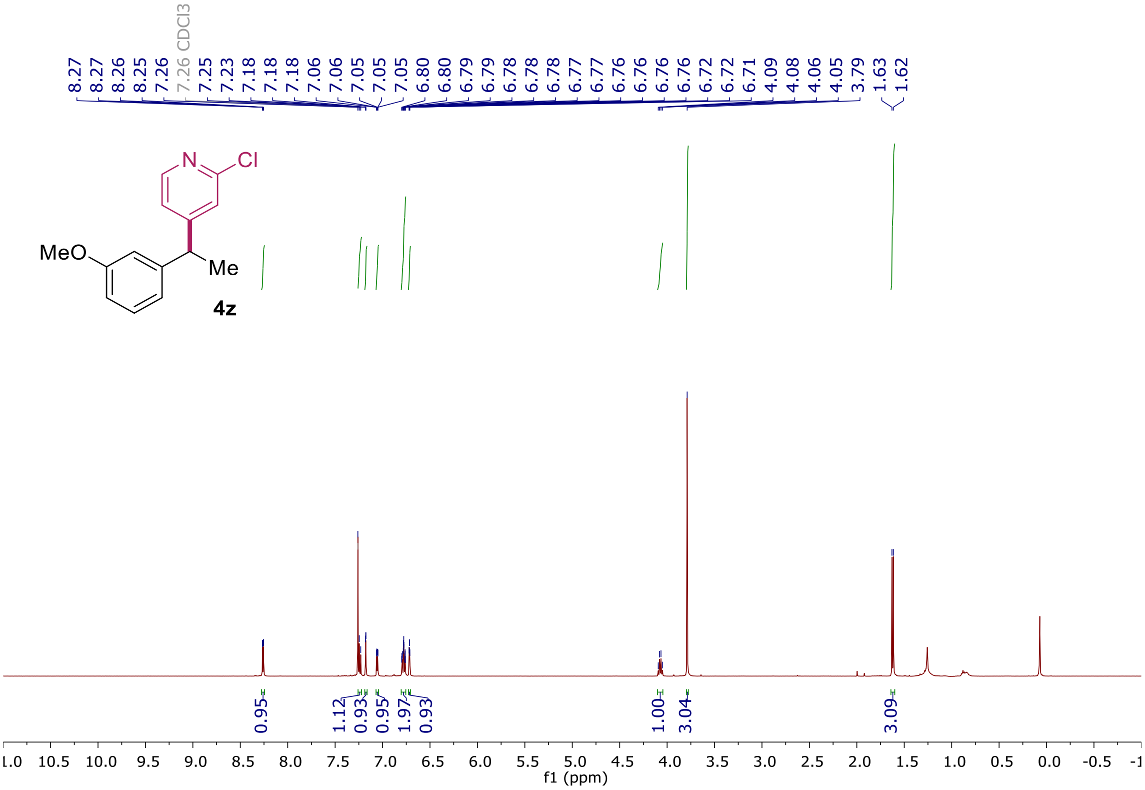

^{13}C-NMR (75 MHz, $CDCl_3$) of compound **4z**

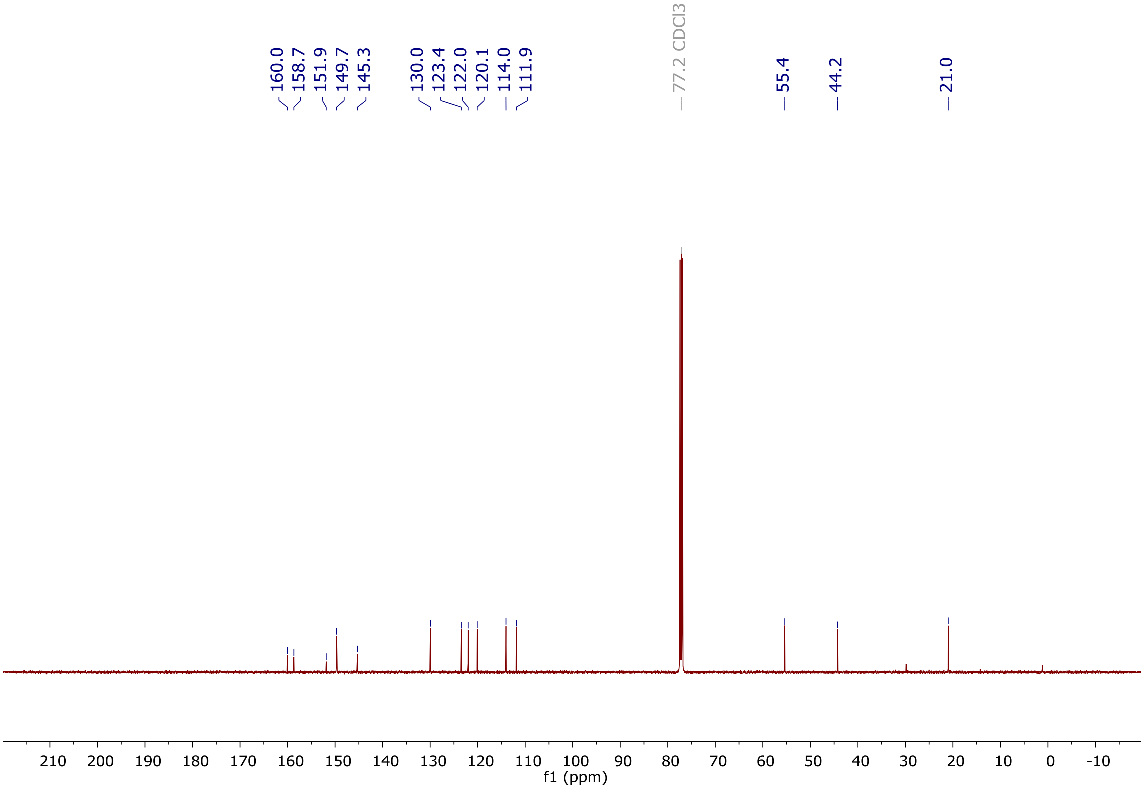

[1]**H-NMR** (300 MHz, $CDCl_3$) of compound **4aa**

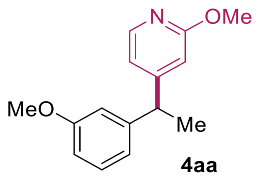

4aa

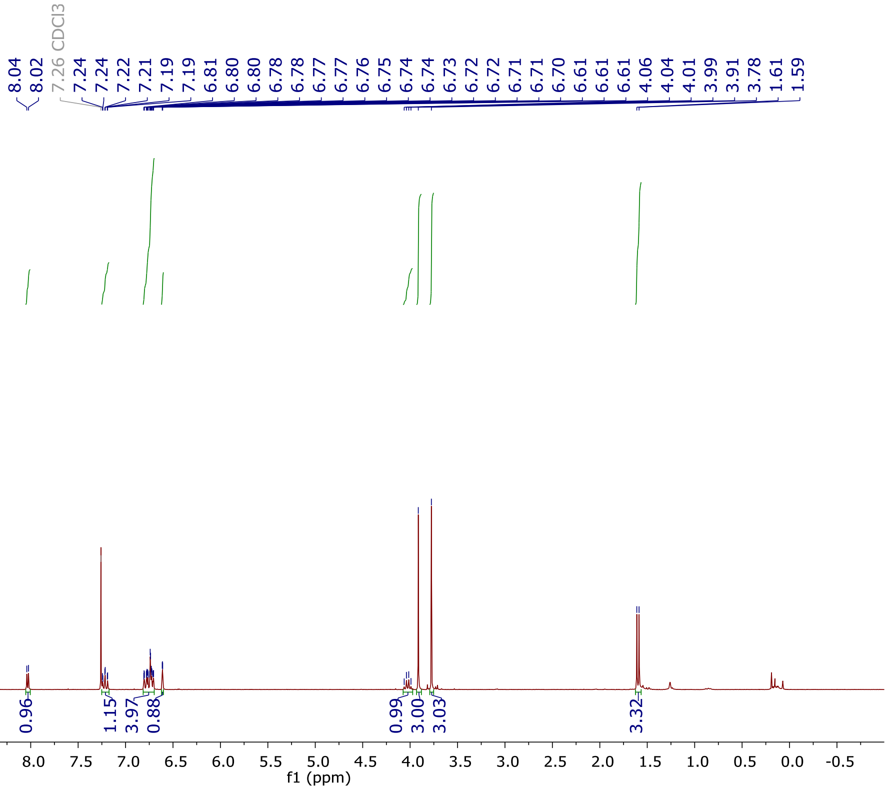

[13]**C-NMR** (75 MHz, $CDCl_3$) of compound **4aa**

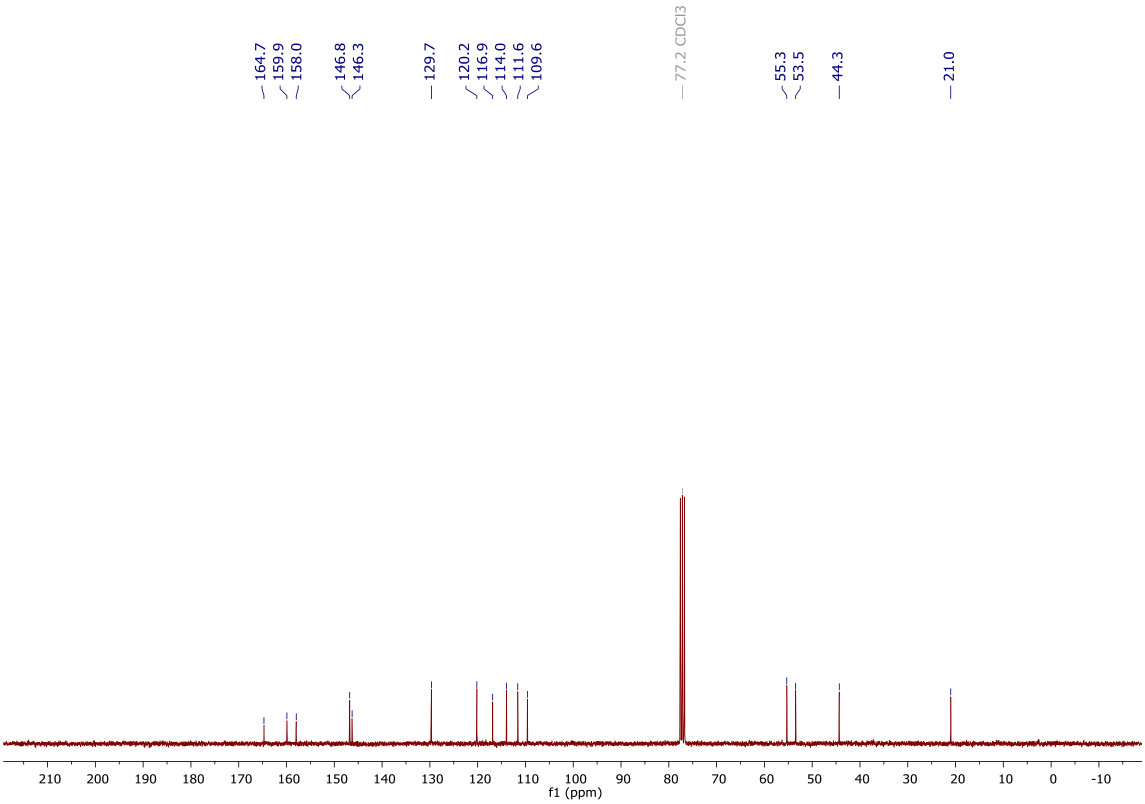

^{1}H-NMR (300 MHz, $CDCl_3$) of compound **4ab**

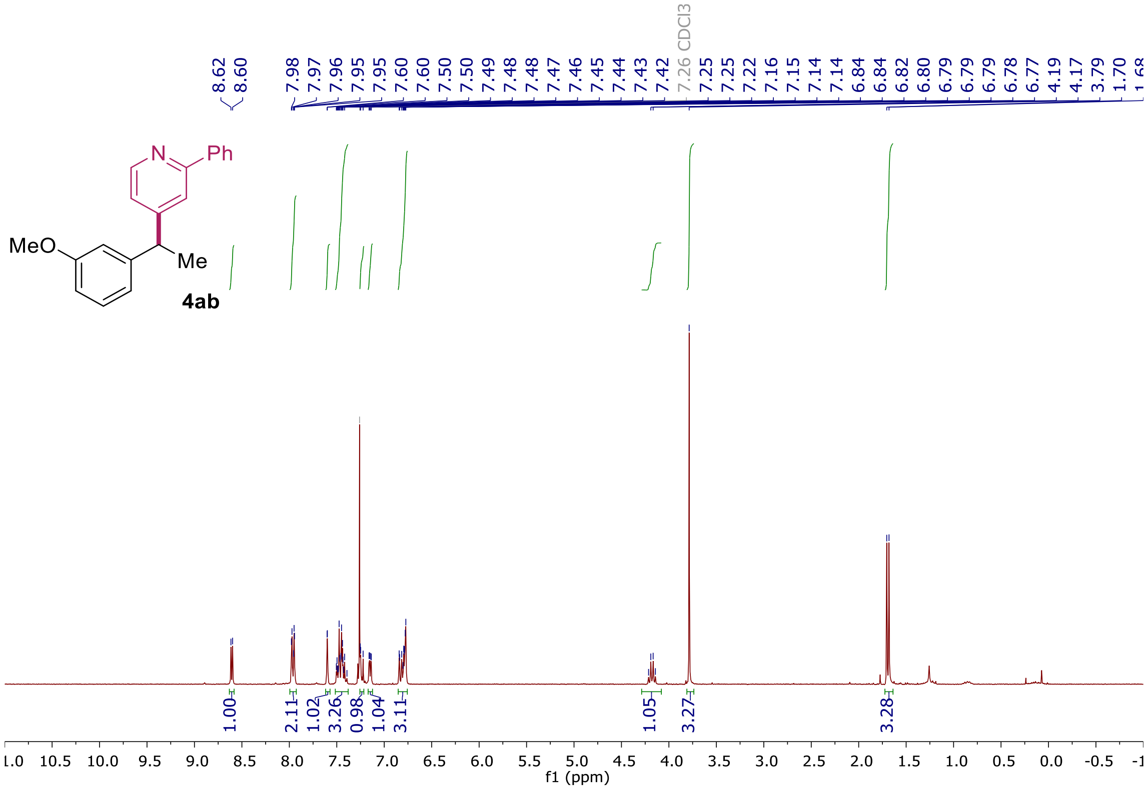

^{13}C-NMR (75 MHz, $CDCl_3$) of compound **4ab**

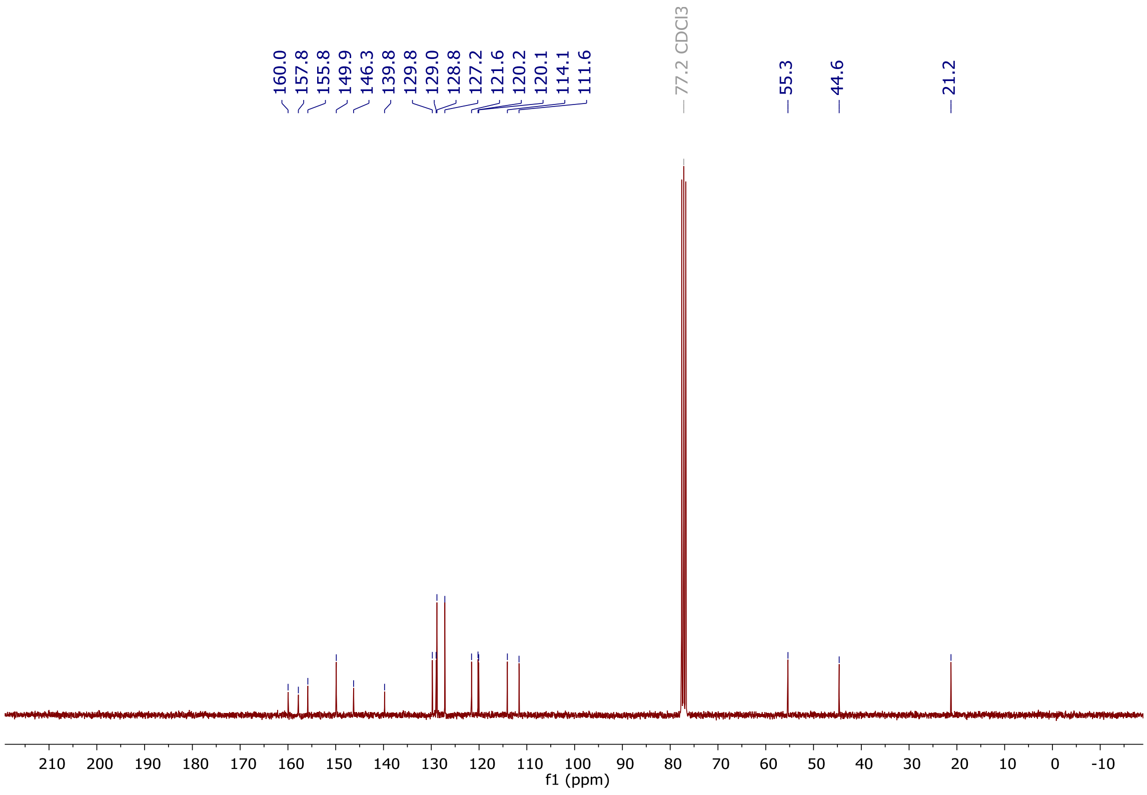

^{1}H-NMR (300 MHz, $CDCl_3$) of compound **4ac**

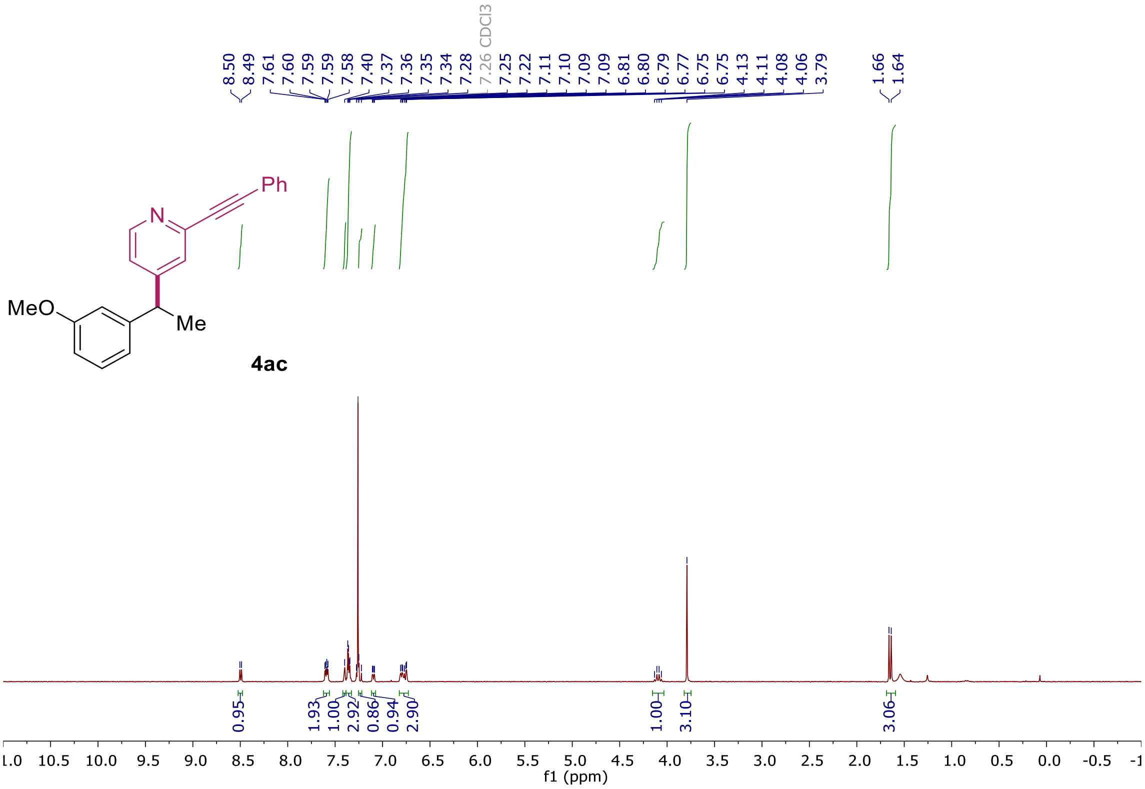

^{13}C-NMR (75 MHz, $CDCl_3$) of compound **4ac**

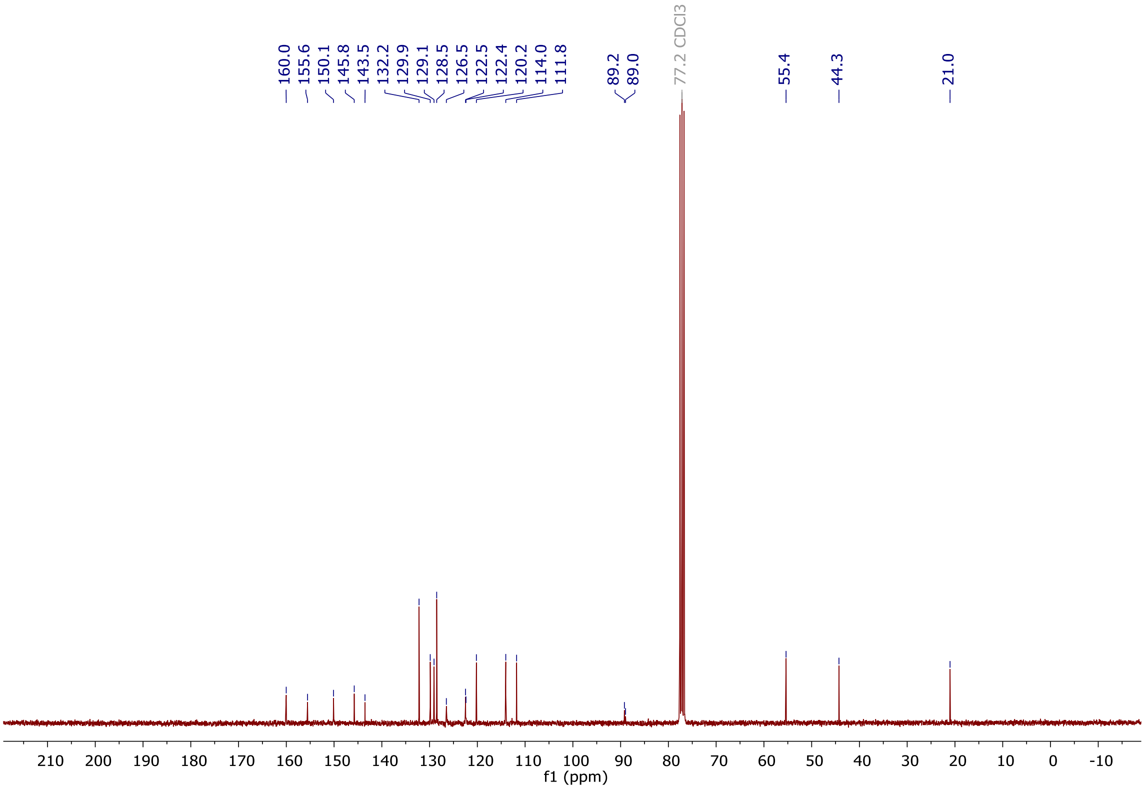

^{1}H-NMR (300 MHz, $CDCl_3$) of compound **4ad**

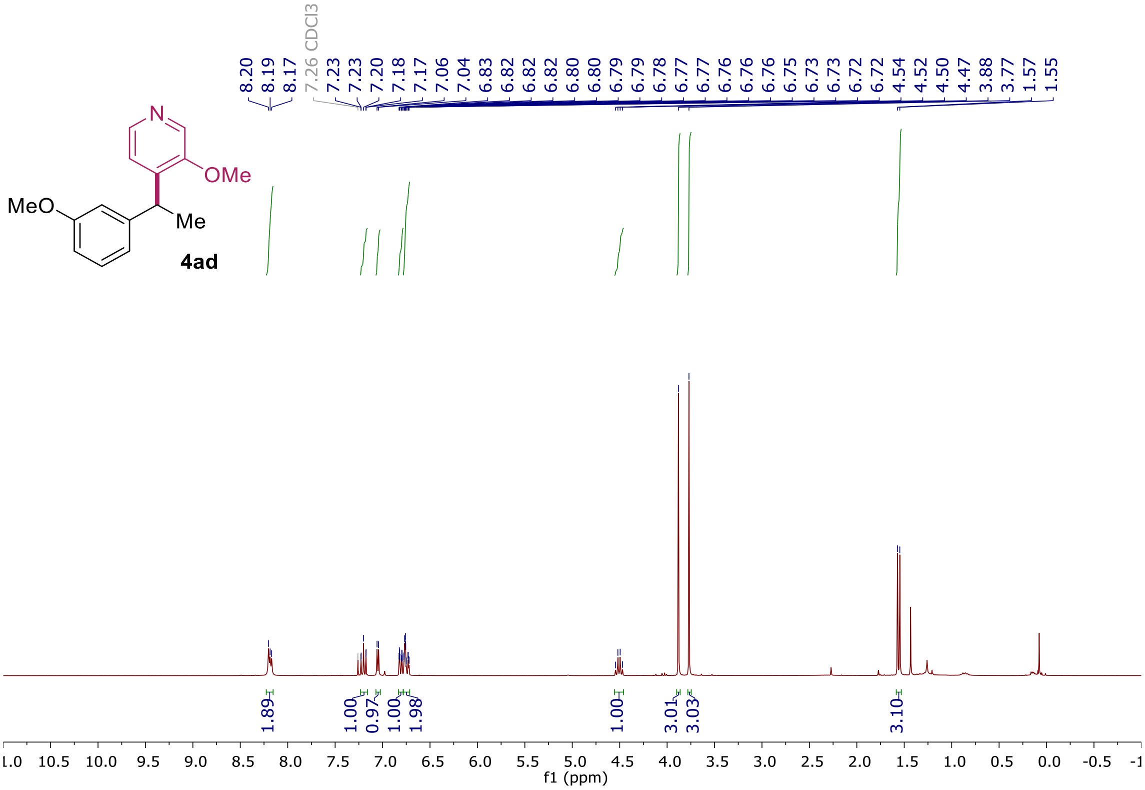

^{13}C-NMR (75 MHz, $CDCl_3$) of compound **4ad**

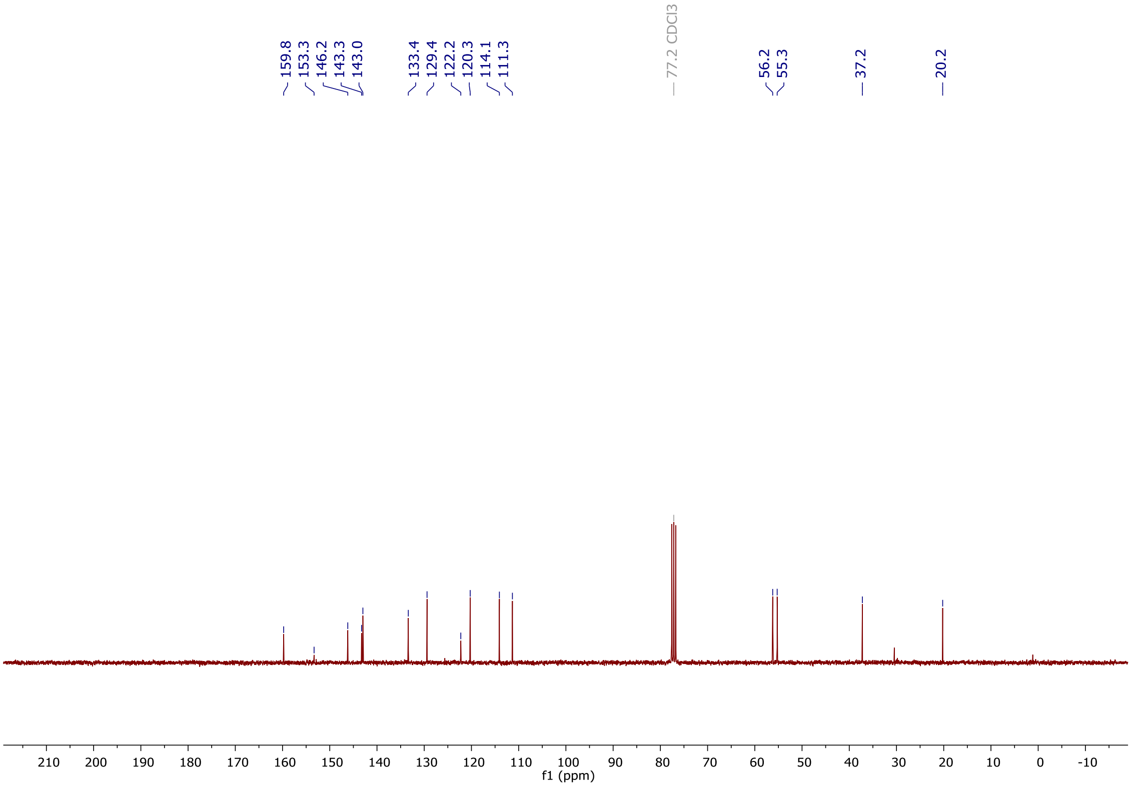

[1]**H-NMR** (300 MHz, $CDCl_3$) of compound **4ae**

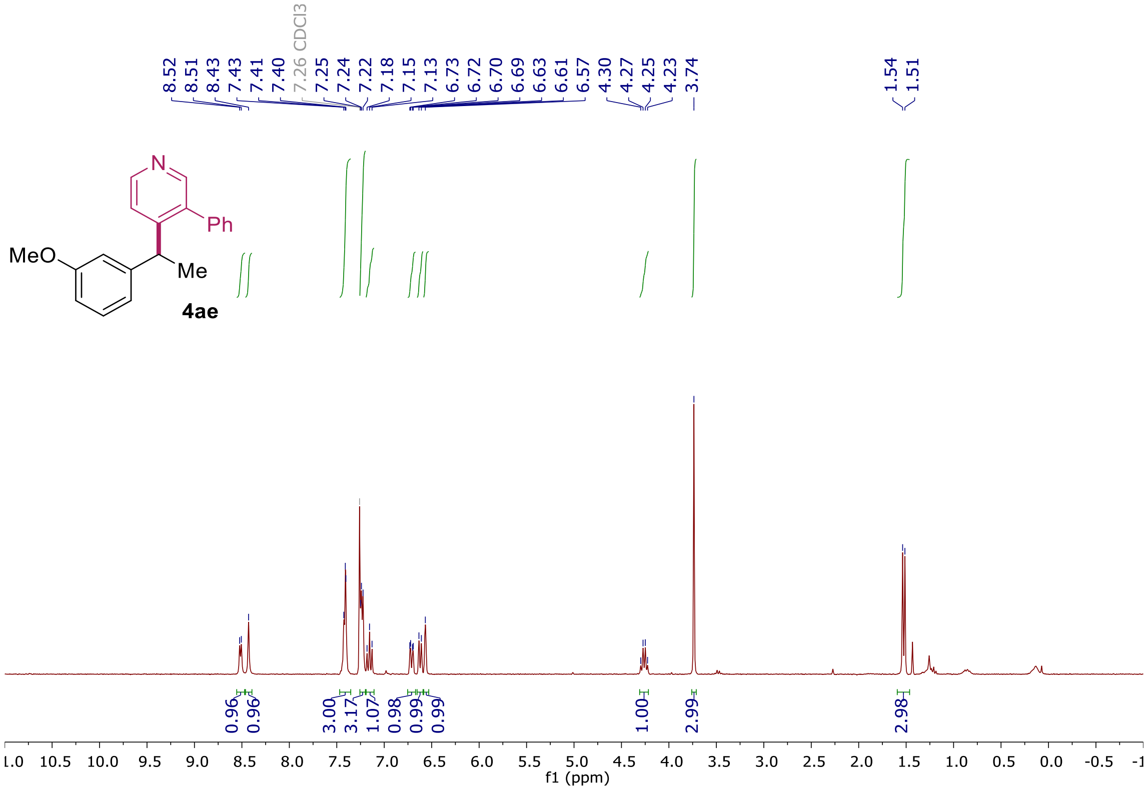

[13]**C-NMR** (75 MHz, $CDCl_3$) of compound **4ae**

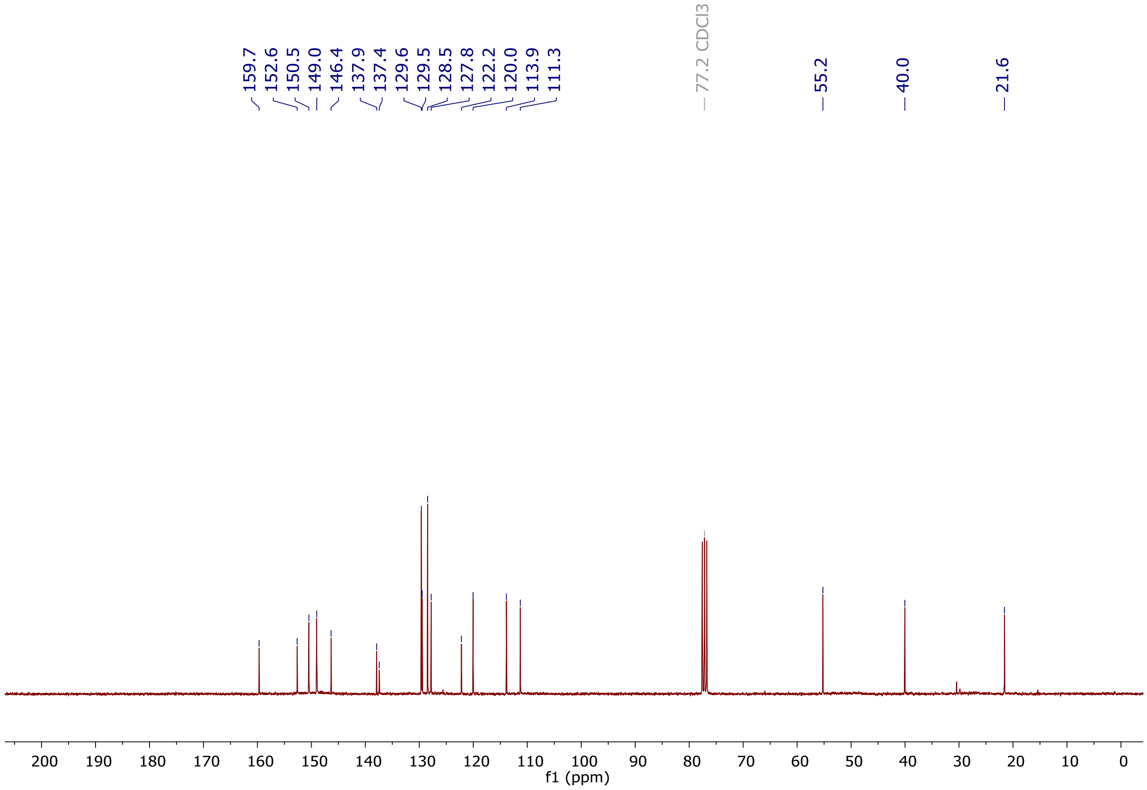

[1]**H-NMR** (300 MHz, $CDCl_3$) of compound **4af**

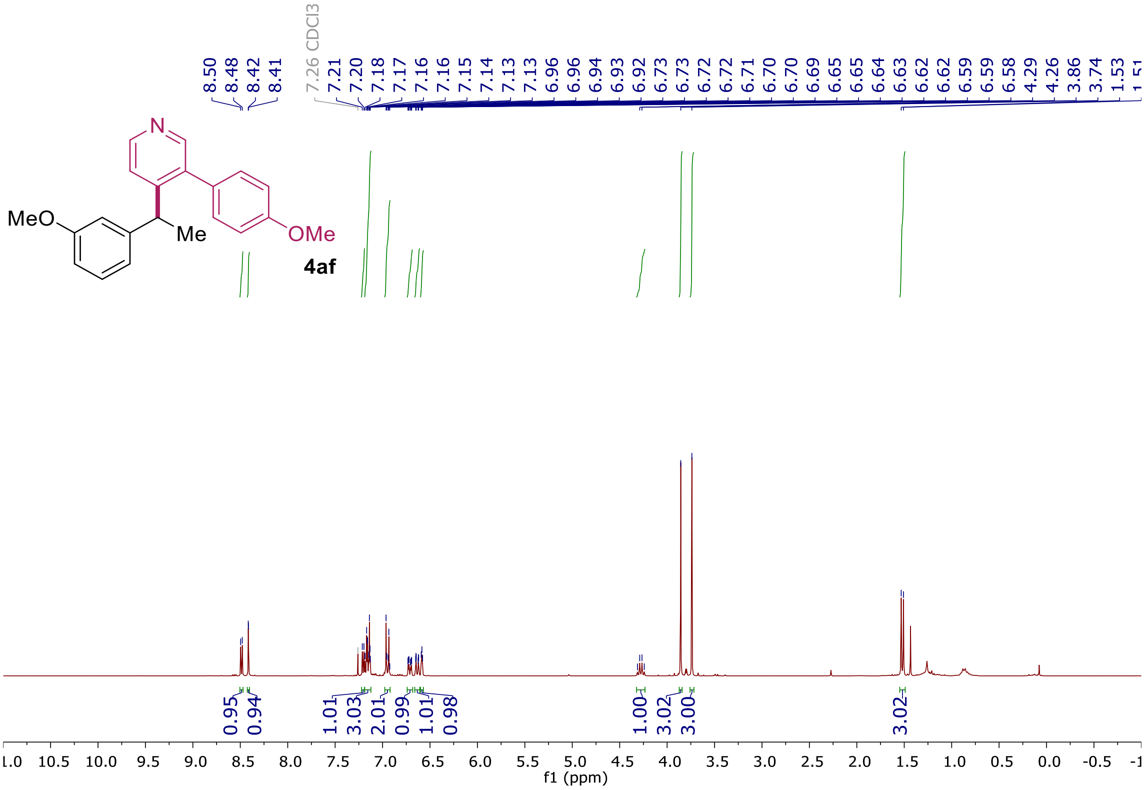

[13]**C-NMR** (75 MHz, $CDCl_3$) of compound **4af**

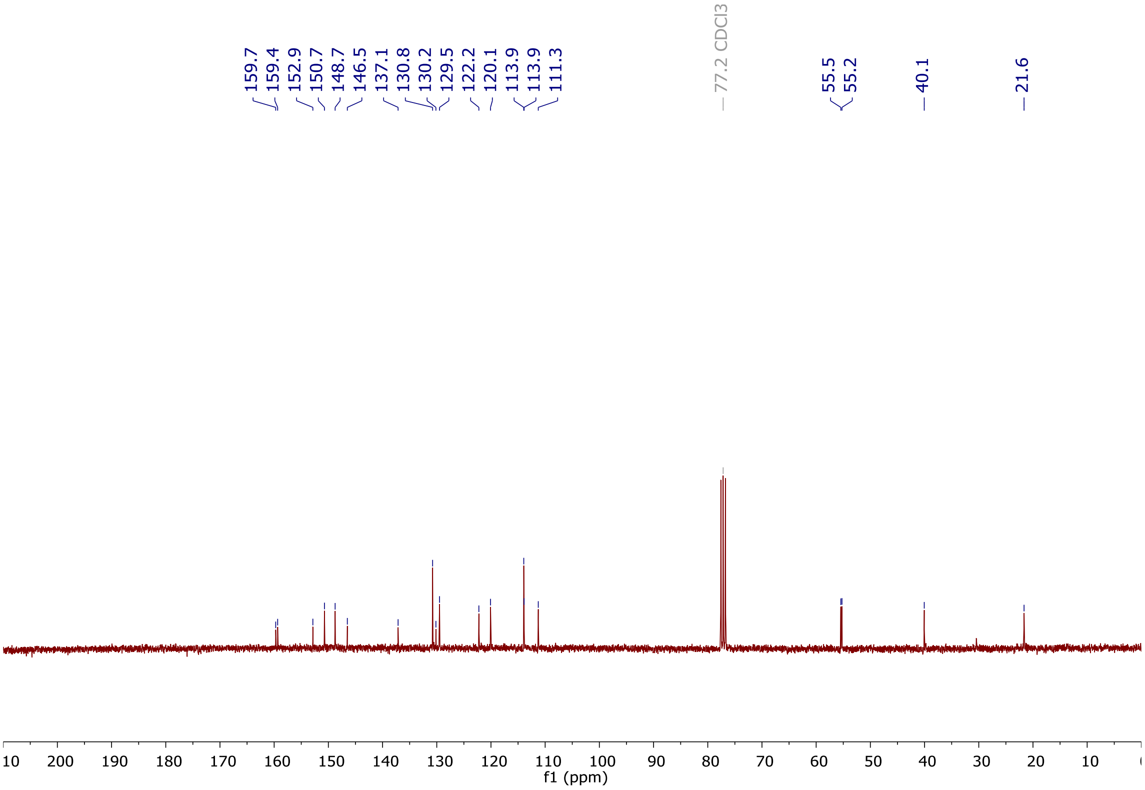

^{1}H-NMR (300 MHz, $CDCl_3$) of compound **4ag**

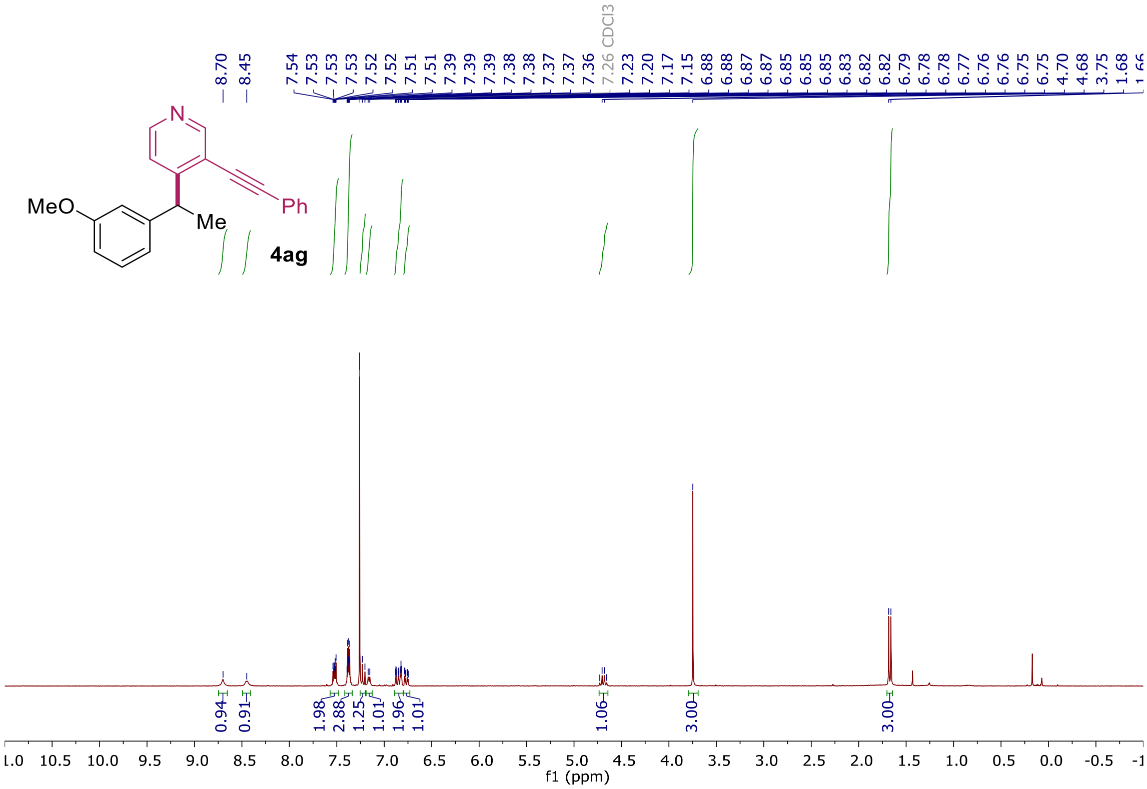

^{13}C-NMR (75 MHz, $CDCl_3$) of compound **4ag**

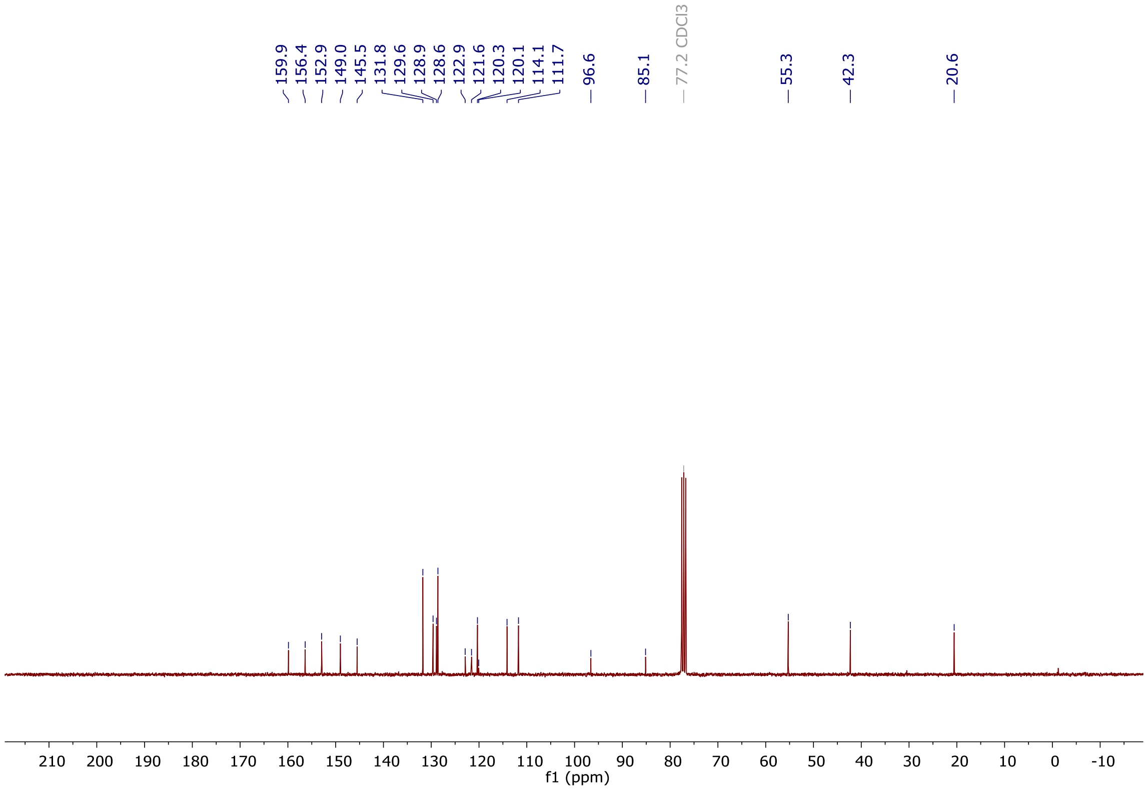

^{1}H-NMR (300 MHz, DMSO-d_6) of compound **4ah, T = 27 ºC**

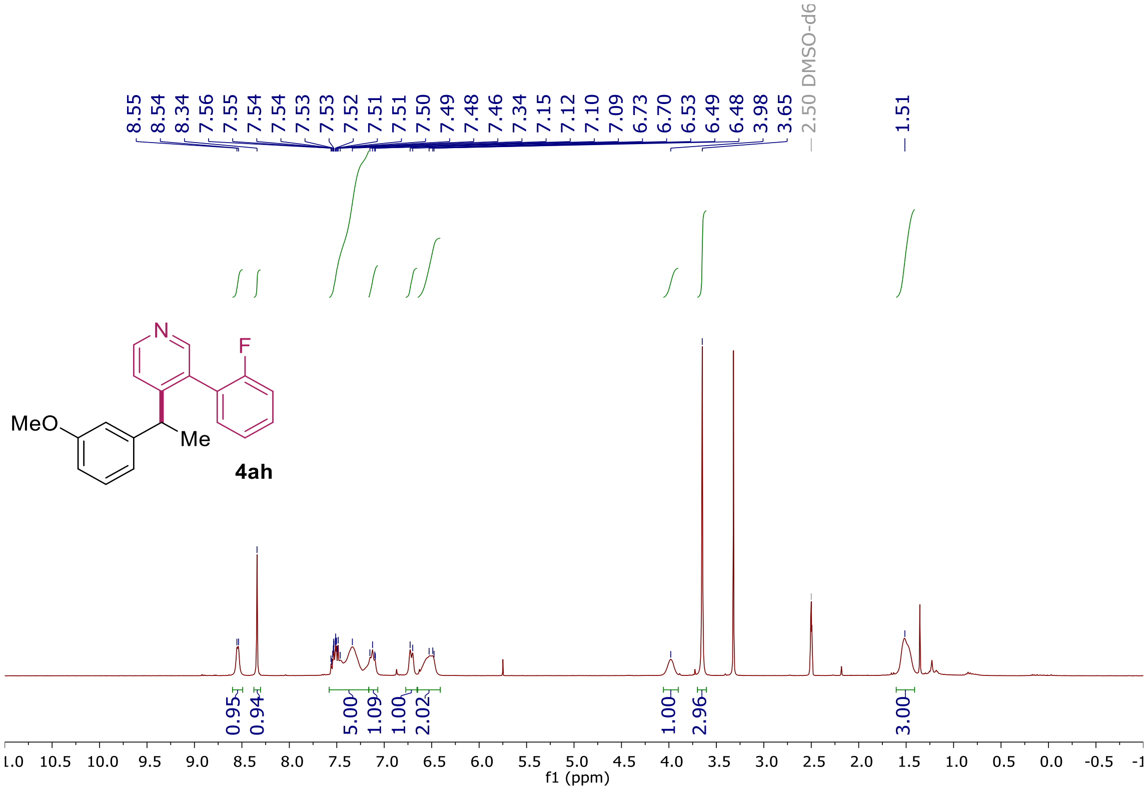

^{1}H-NMR (300 MHz, DMSO-d_6) of compound **4ah, T = 80 ºC**

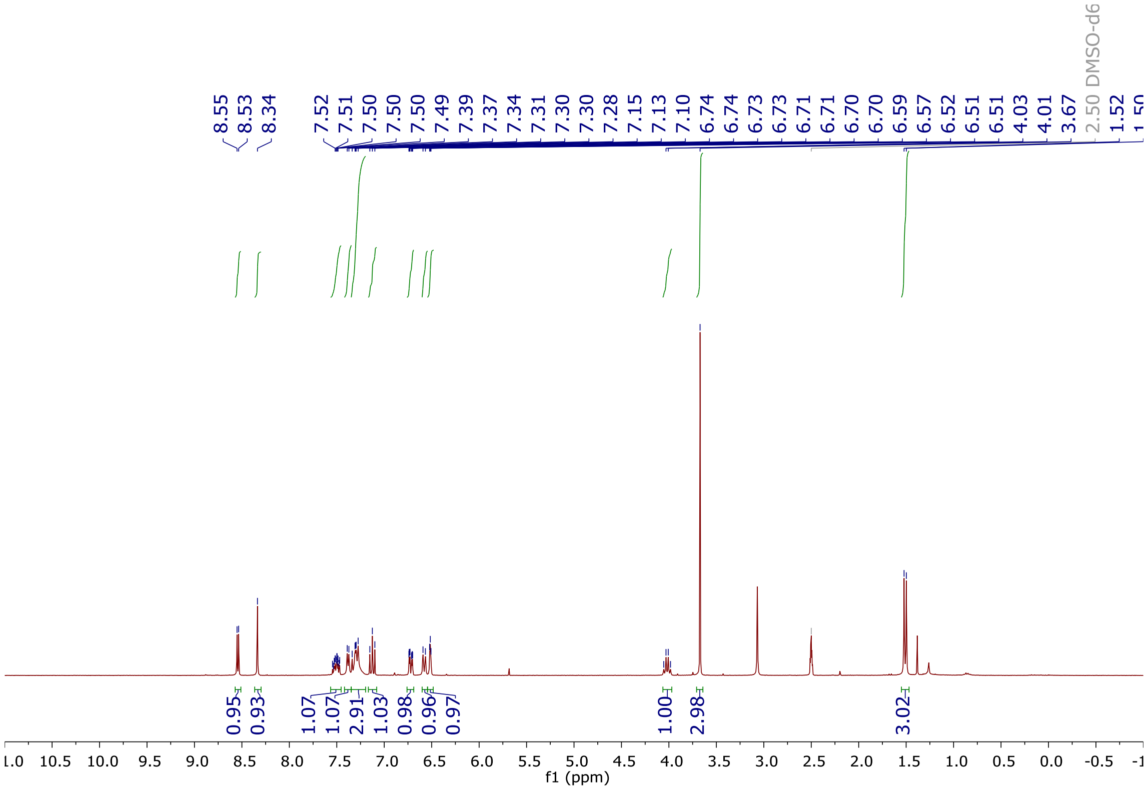

^{13}C-NMR (75 MHz, DMSO-d_6) of compound **4ah, T = 80 ºC**

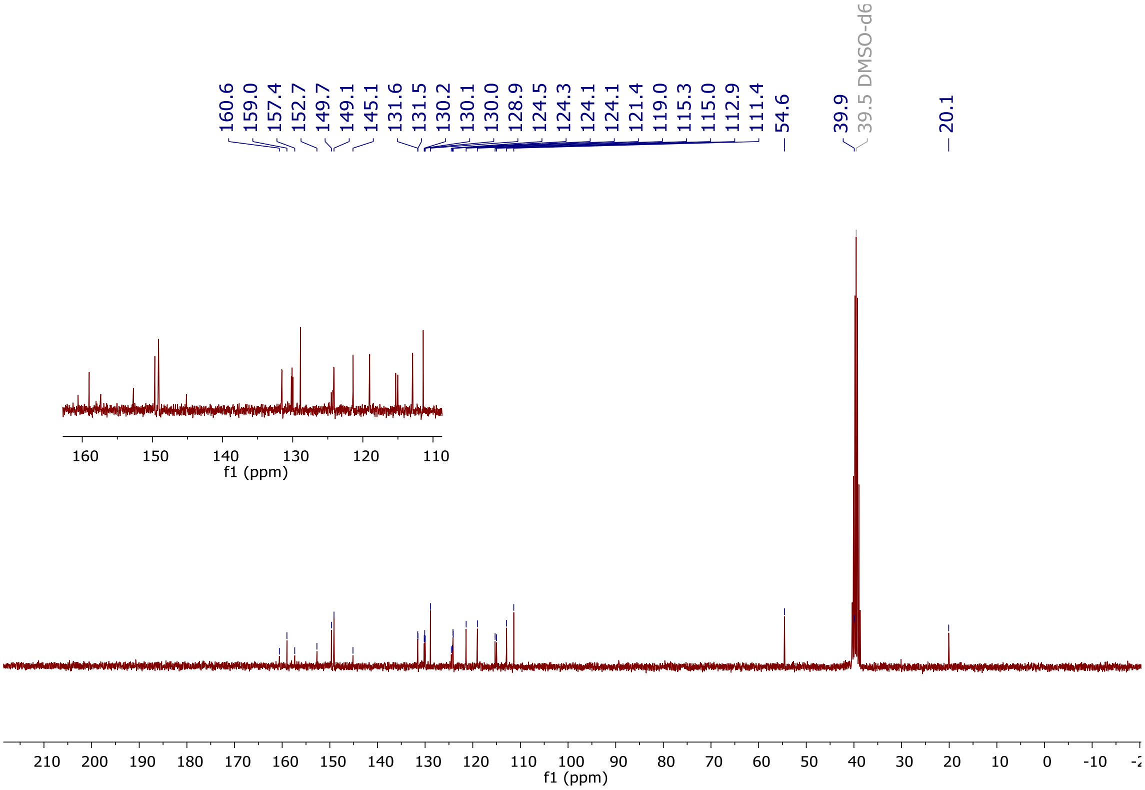

^{19}F-NMR (470 MHz, $CDCl_3$) of compound **4ah, T = 27 ºC**

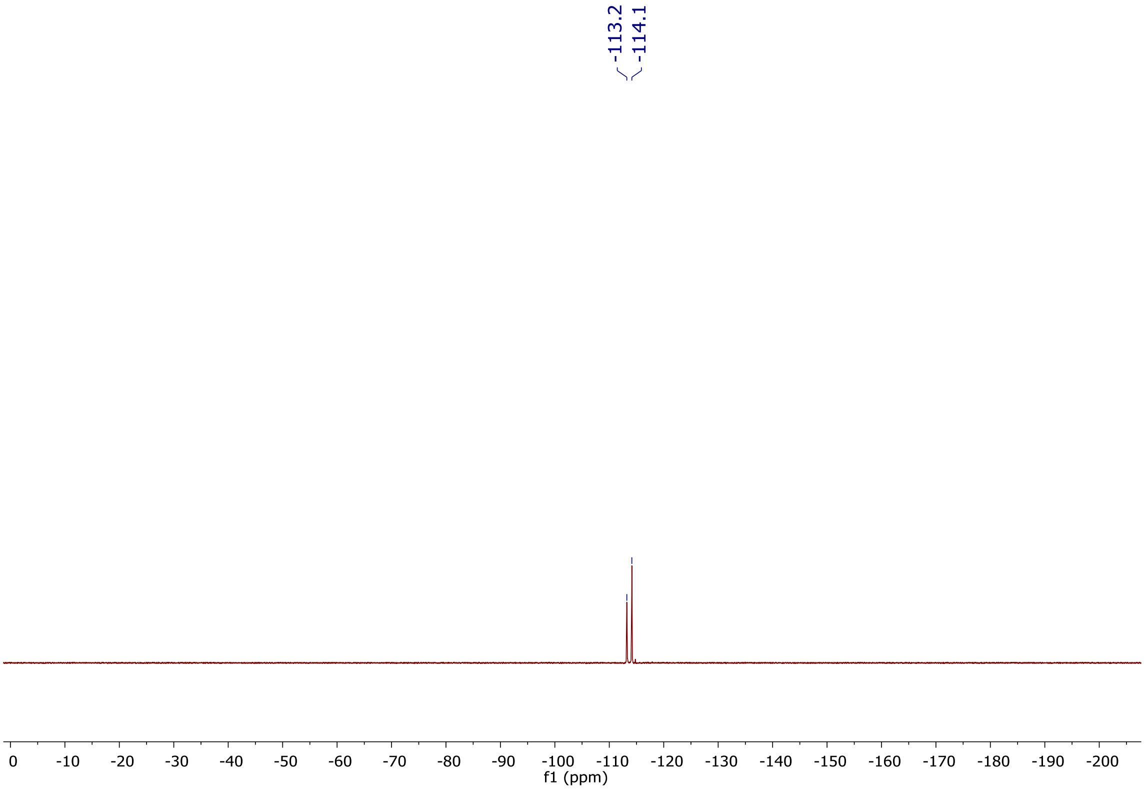

^{19}F-NMR (300 MHz, DMSO-d_6) of compound **4ah, T = 80 ºC**

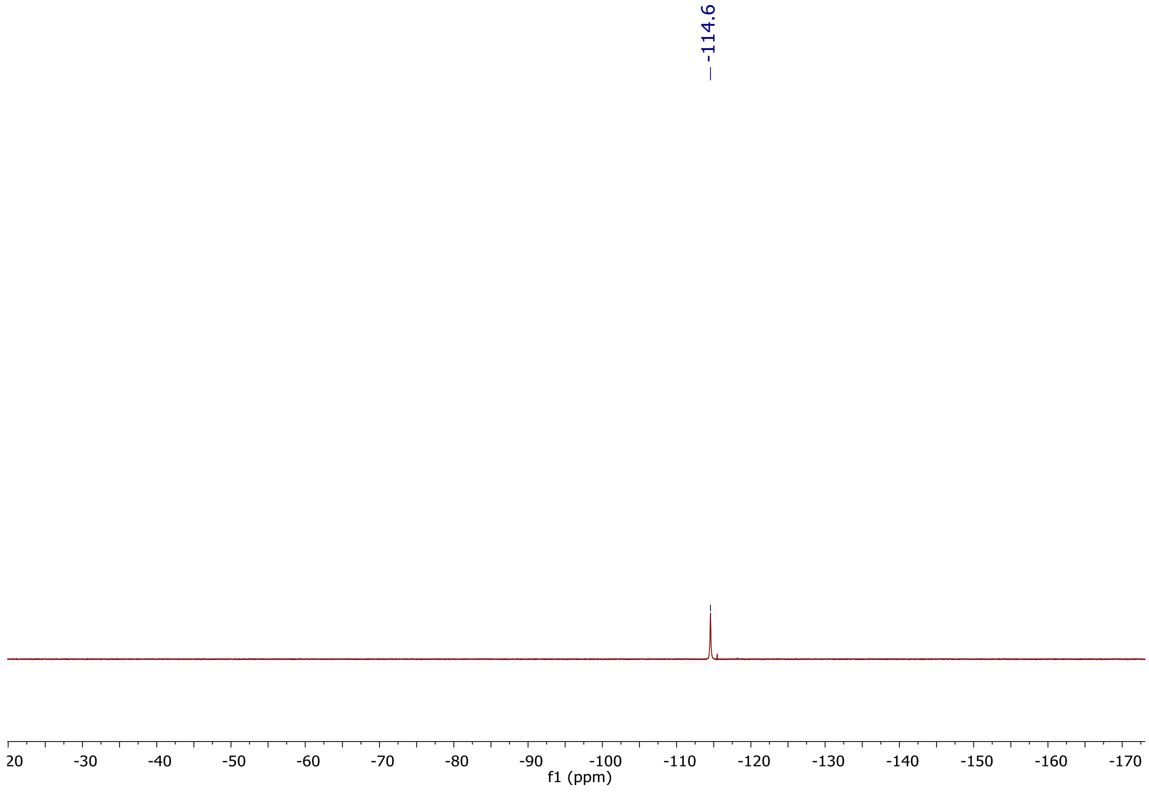

^{1}H-NMR (300 MHz, DMSO-d_6) of compound **4ai, T = 27 ºC**

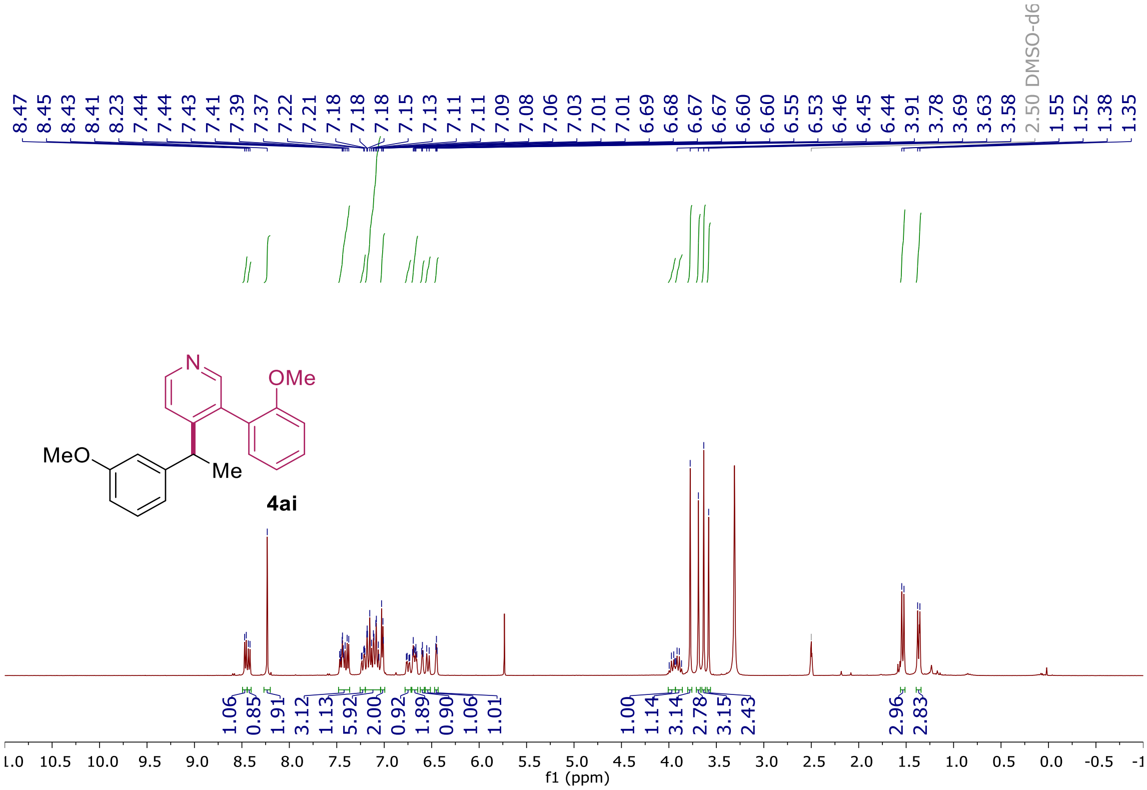

^{1}H-NMR (300 MHz, DMSO-d_6) of compound **4ai, T = 120 ºC**

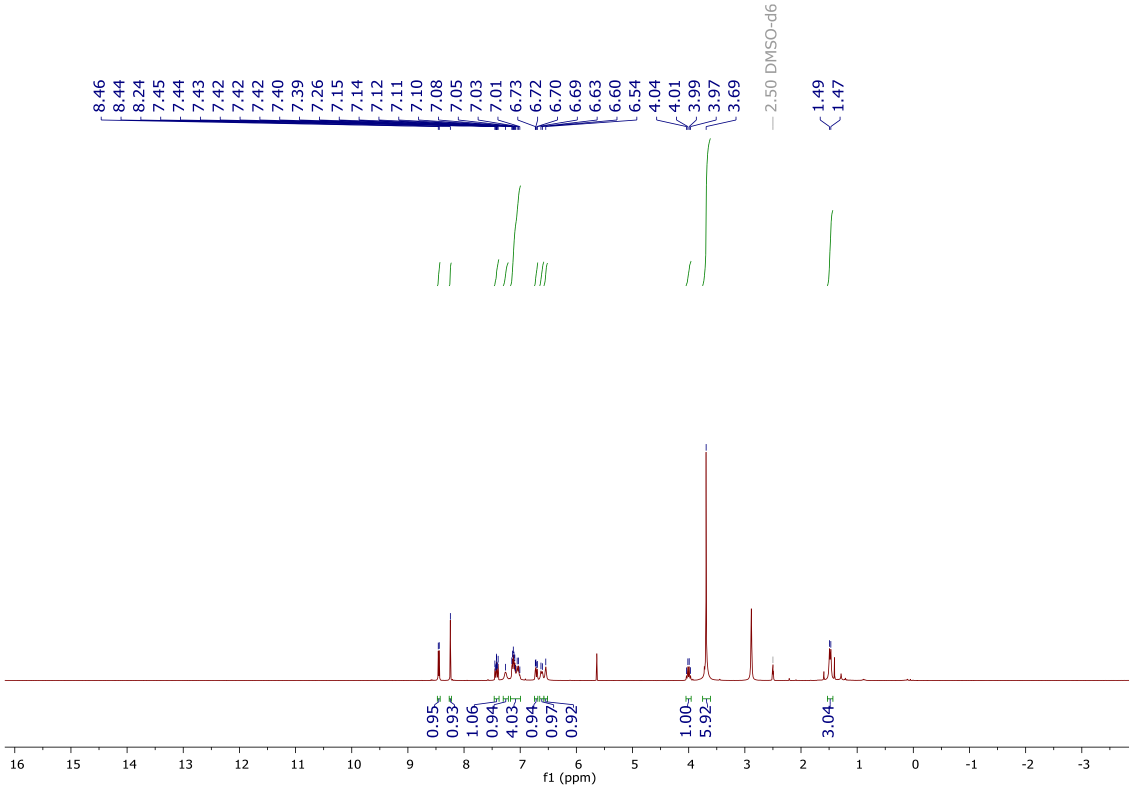

^{13}C-NMR (75 MHz, DMSO-d_6) of compound **4ai, T = 120 ºC**

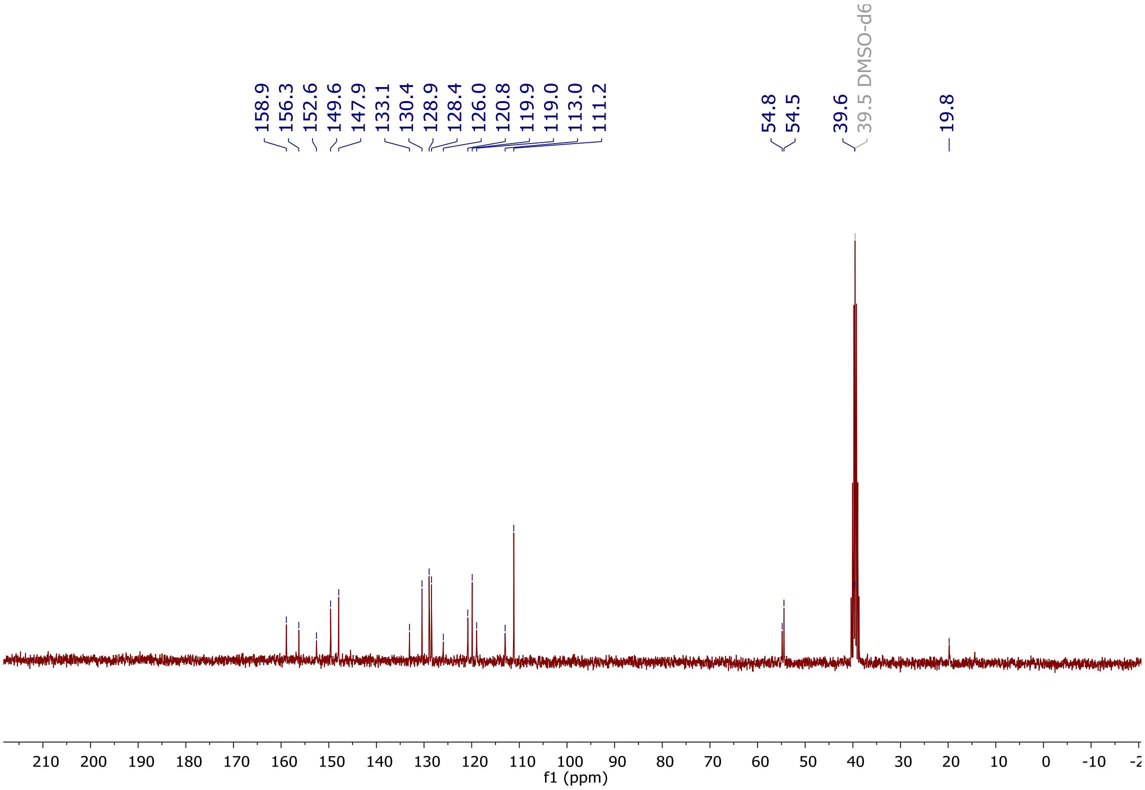

^{1}H-NMR (300 MHz, $CDCl_3$) of compound **4aj**

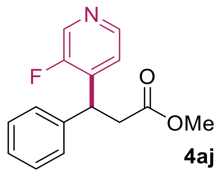

4aj

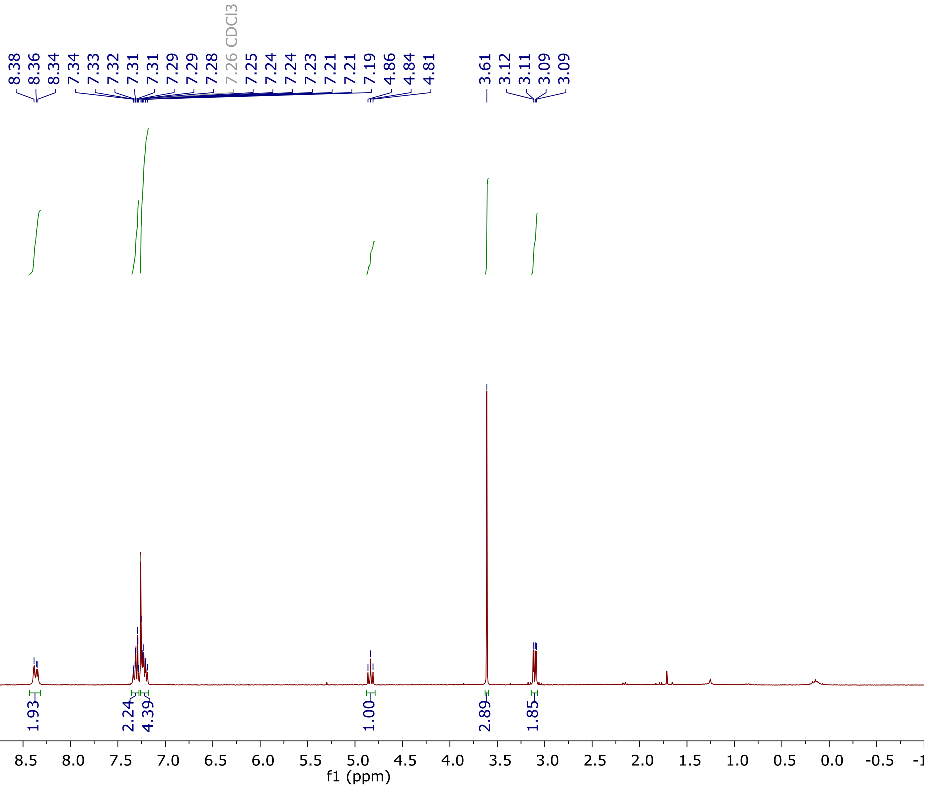

^{13}C-NMR (75 MHz, $CDCl_3$) of compound **4aj**

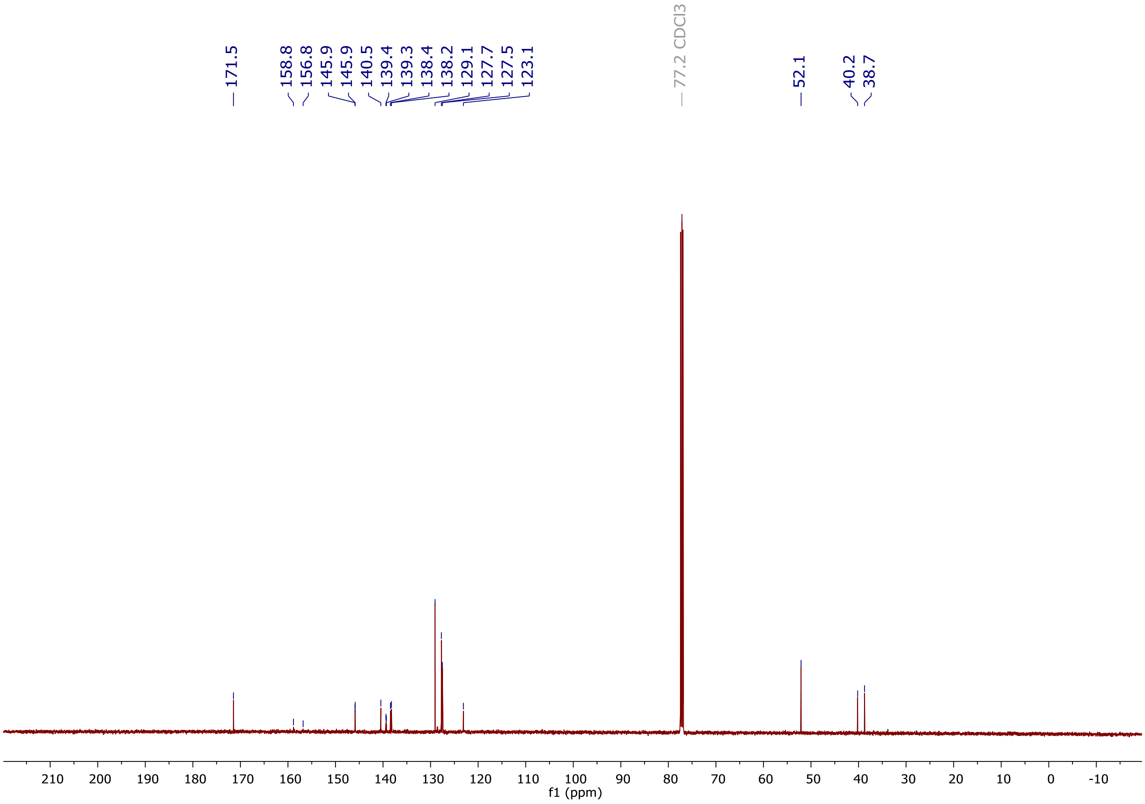

^{19}F-NMR (470 MHz, $CDCl_3$) of compound **4aj**

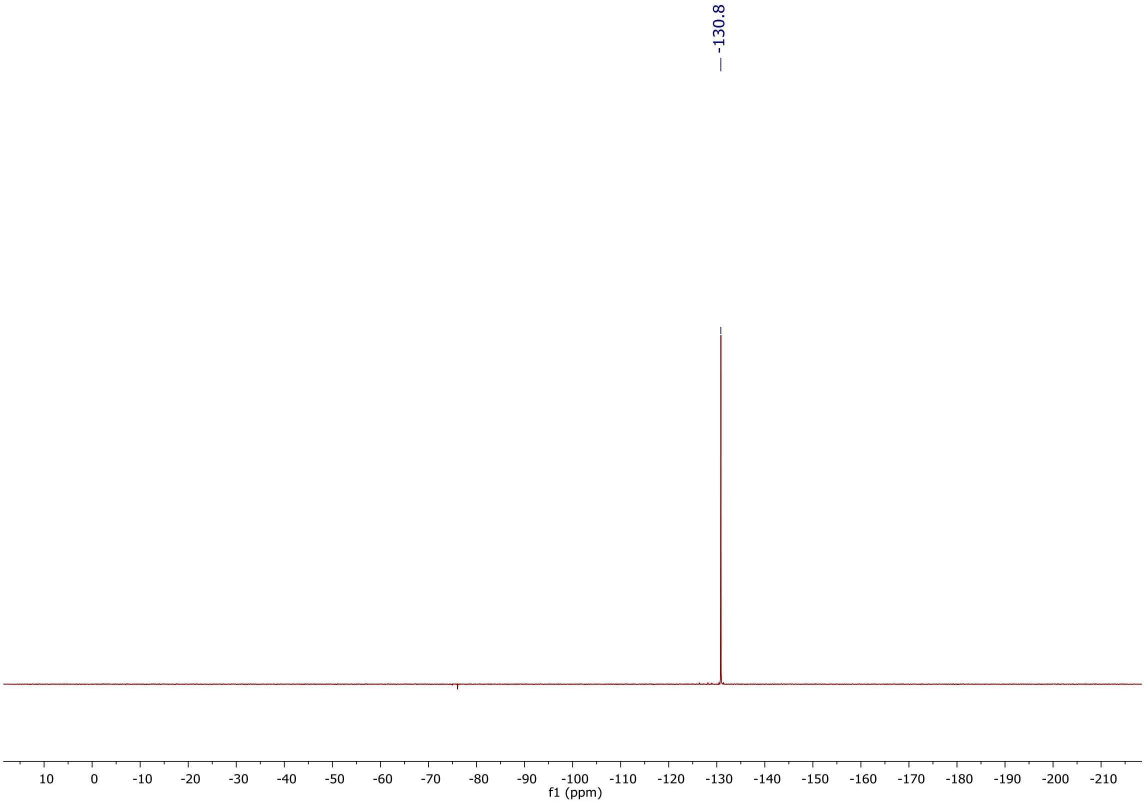

^{1}H-NMR (300 MHz, $CDCl_3$) of compound **4ak**

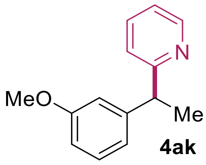

4ak

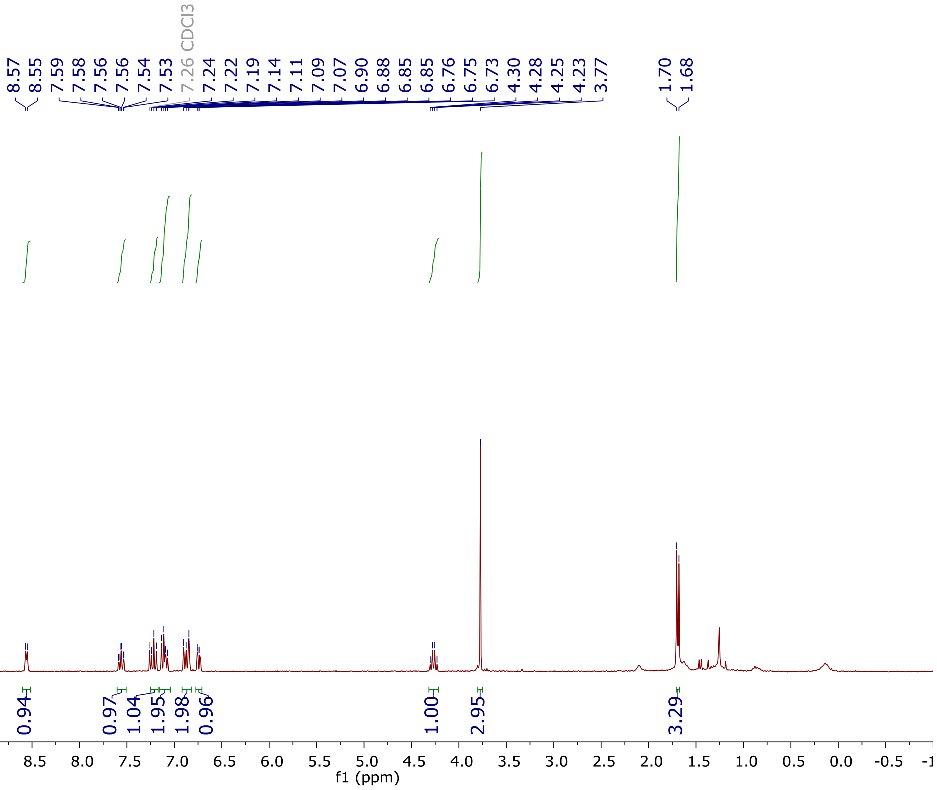

^{1}H-NMR (300 MHz, $CDCl_3$) of compound **4al**

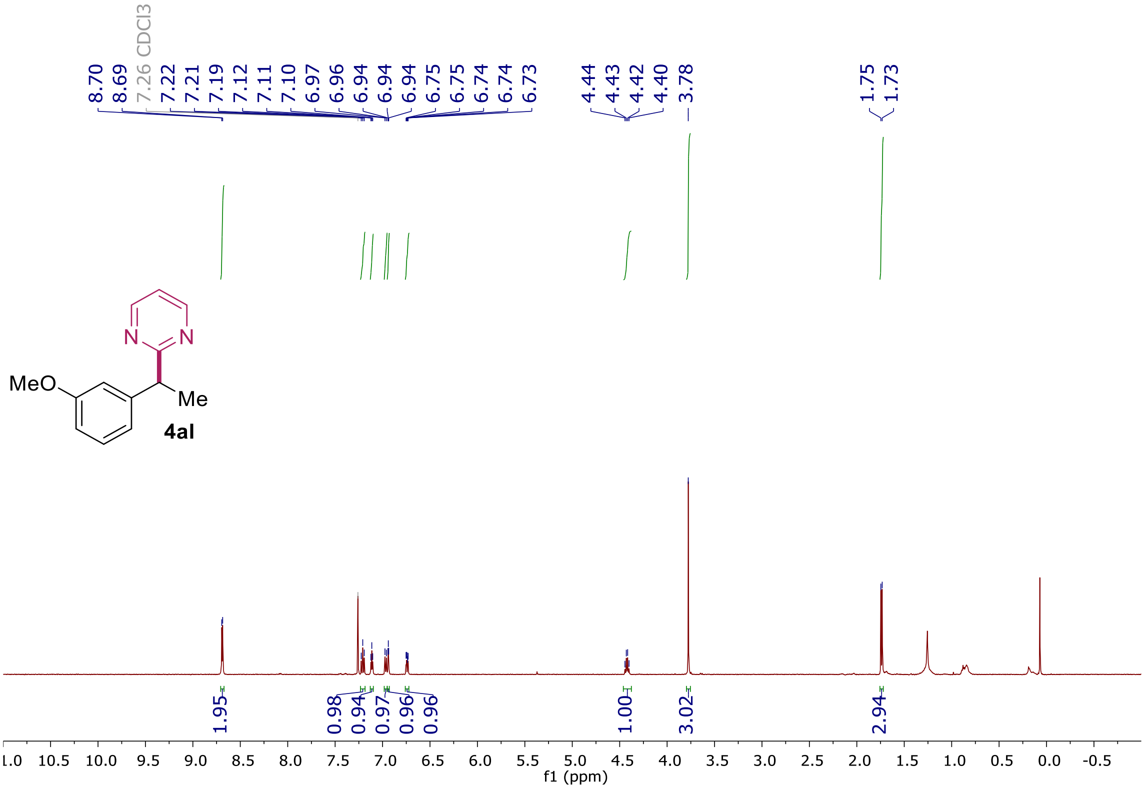

^{13}C-NMR (75 MHz, $CDCl_3$) of compound **4al**

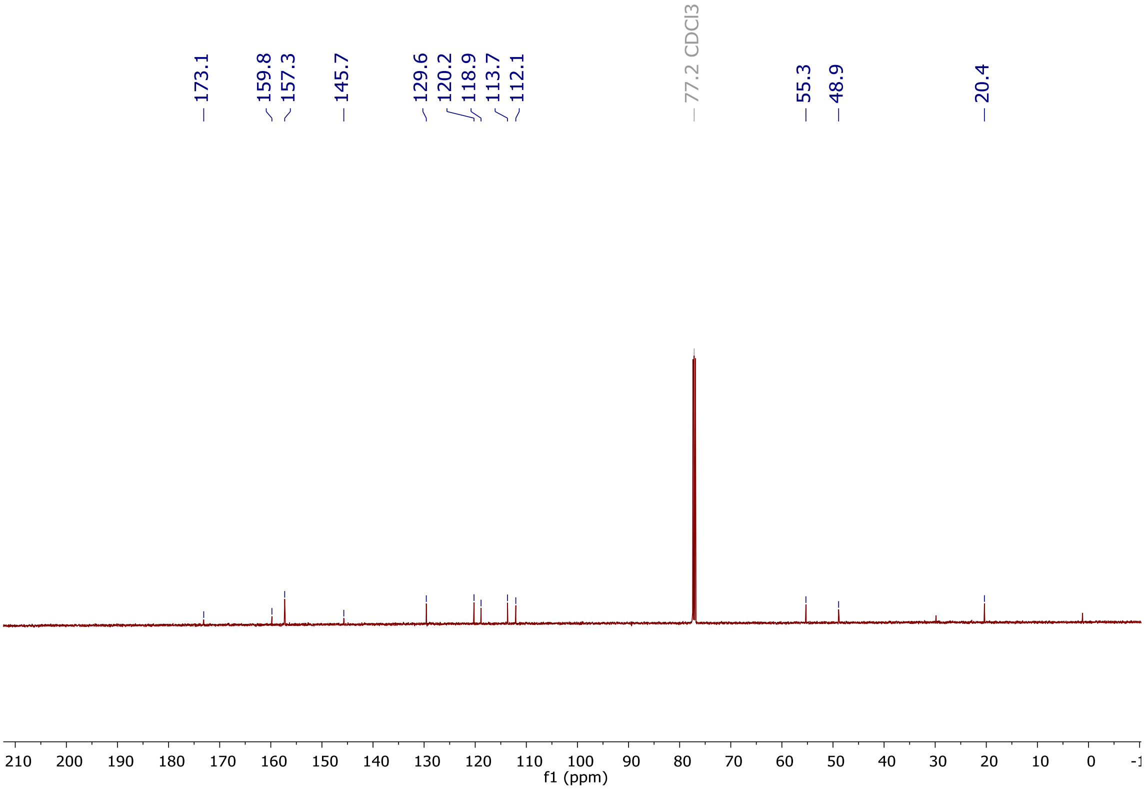

^{1}H-NMR (300 MHz, $CDCl_3$) of compound **4am**

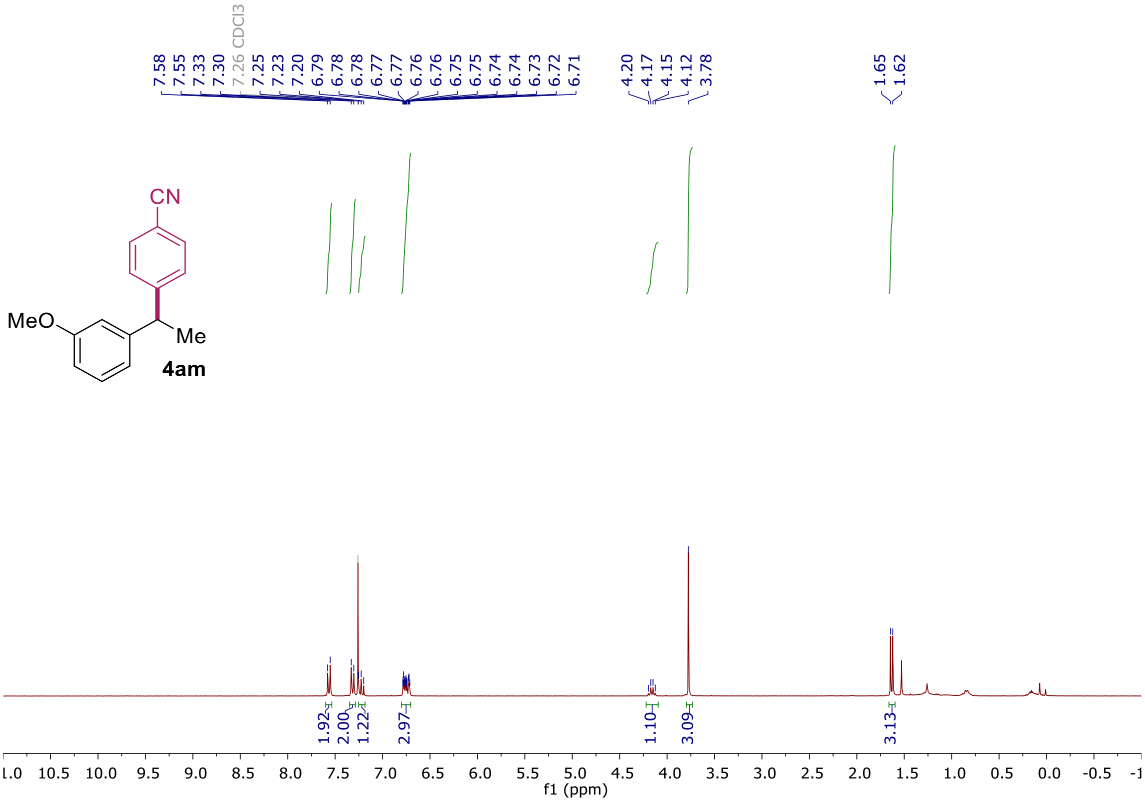

^{13}C-NMR (75 MHz, $CDCl_3$) of compound **4am**

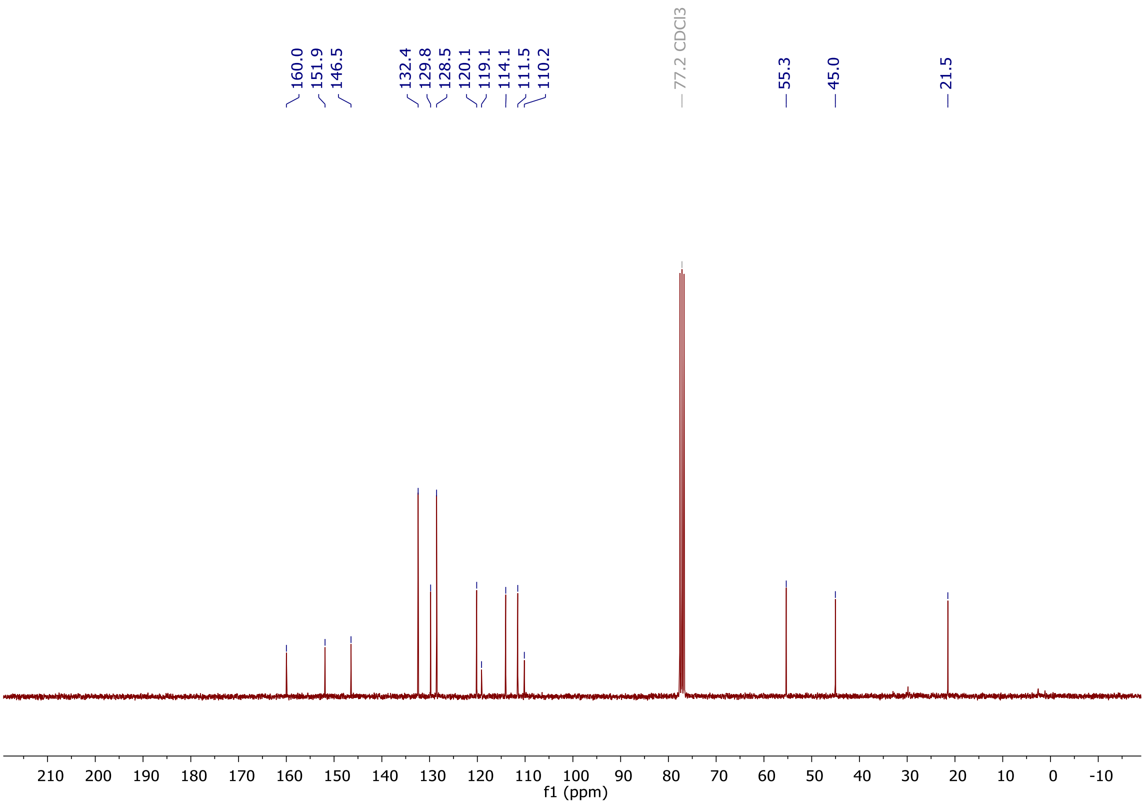

[1]**H-NMR** (300 MHz, $CDCl_3$) of compound **4an**

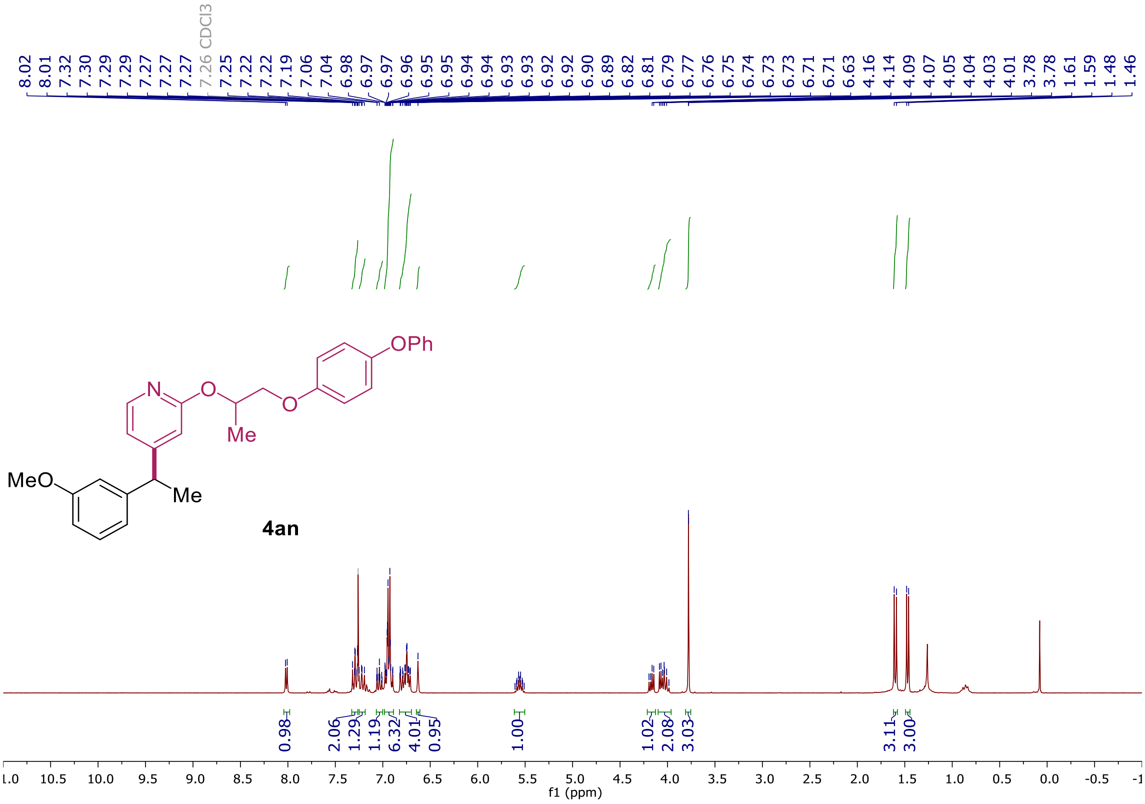

[13]**C-NMR** (75 MHz, $CDCl_3$) of compound **4an**

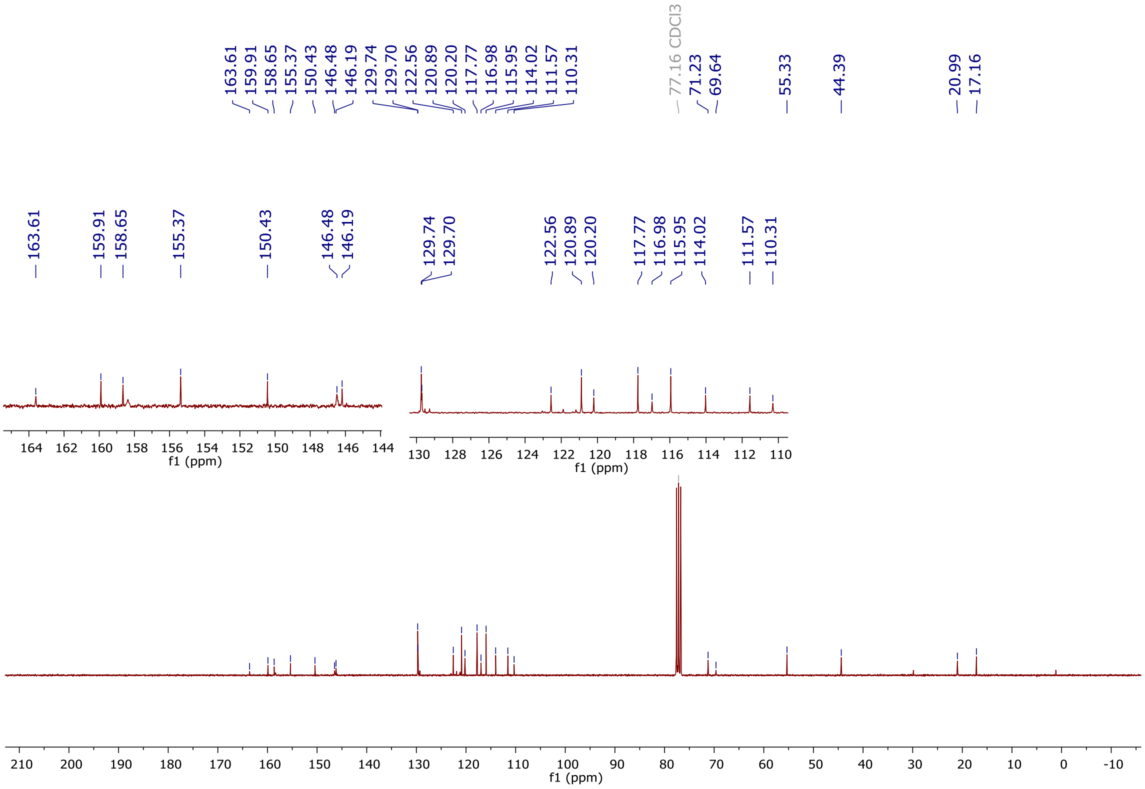

^{1}H-NMR (300 MHz, CDCl$_3$) of compound **4ao**

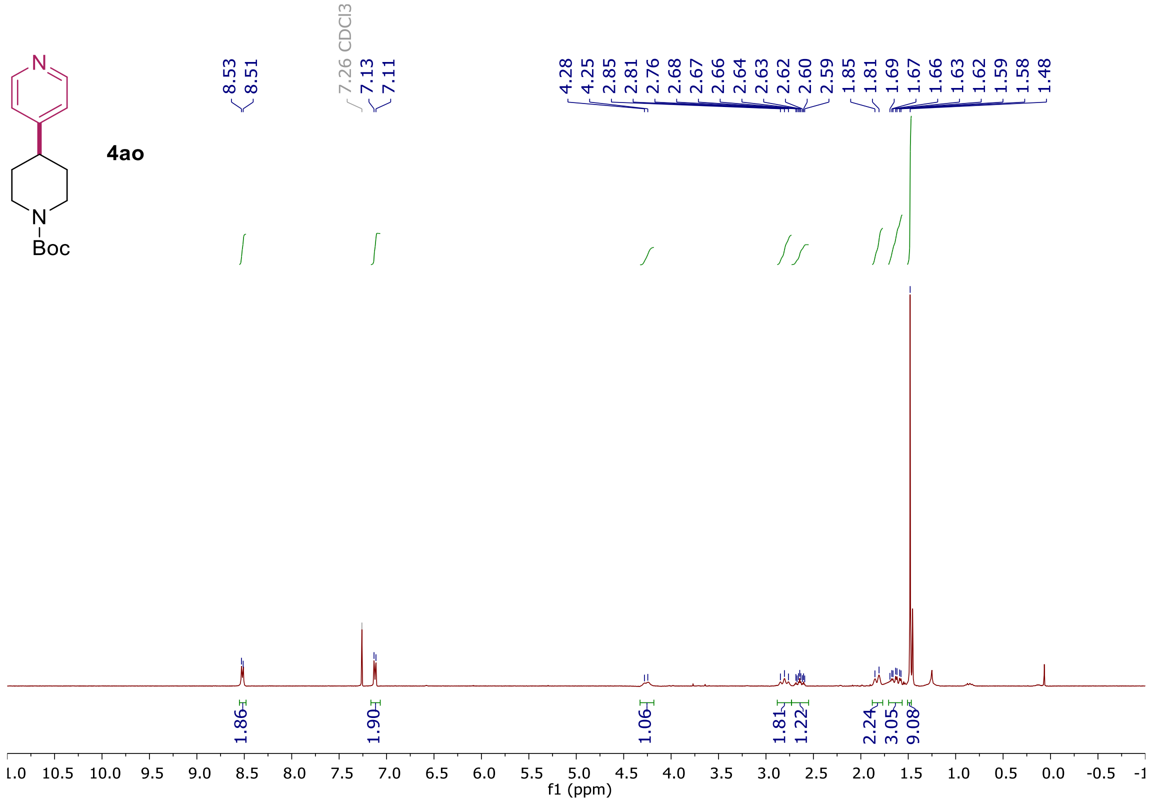

^{1}H-NMR (300 MHz, $CDCl_3$) of compound **4ap**

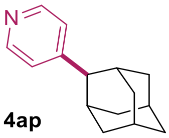

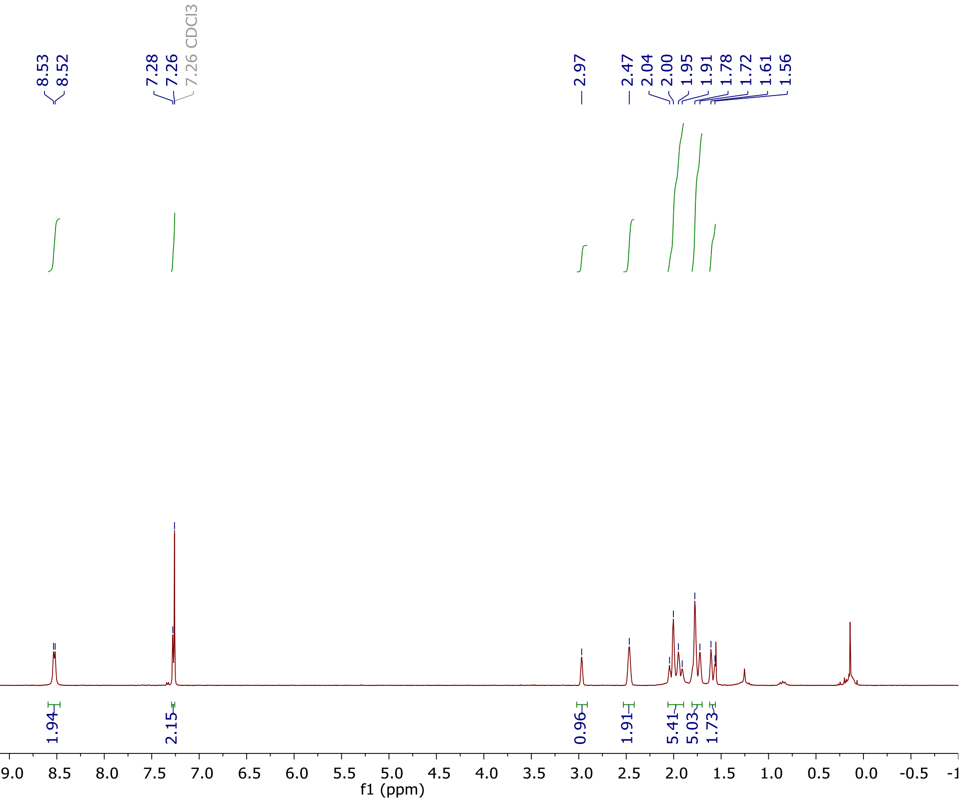

^{13}C-NMR (75 MHz, $CDCl_3$) of compound **4ap**

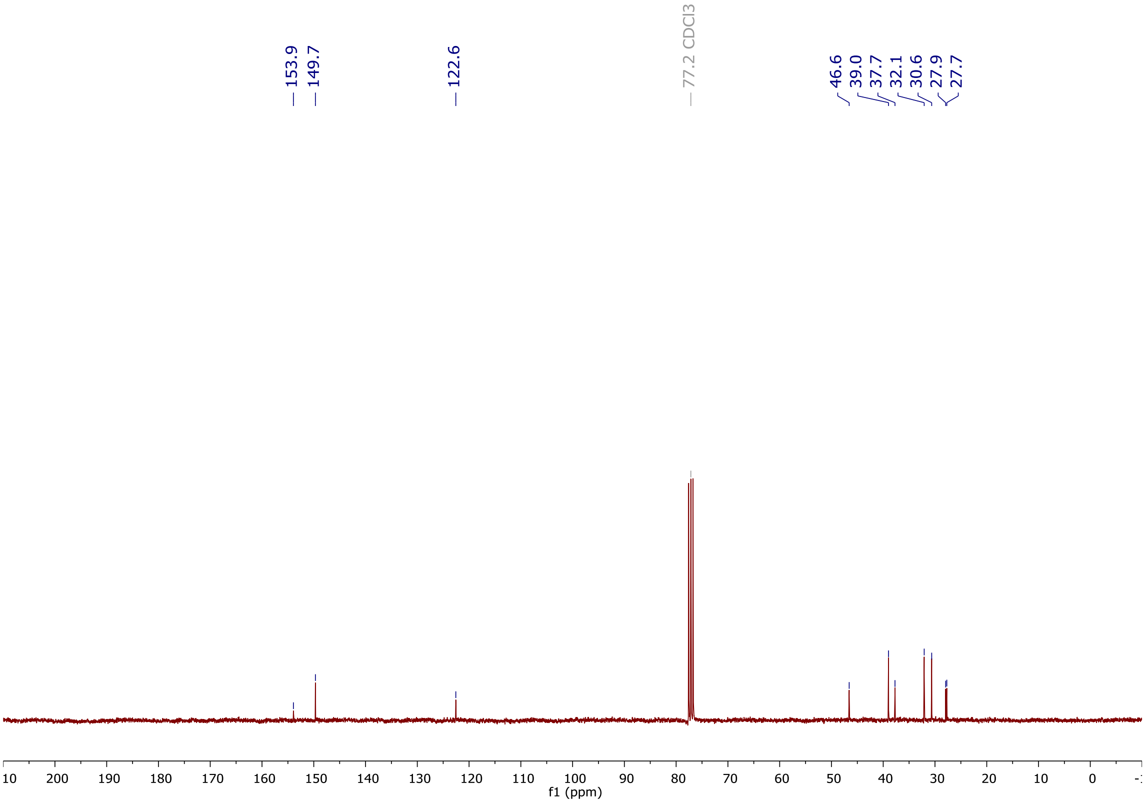

^{1}H-NMR (300 MHz, $CDCl_3$) of compound **4aq**

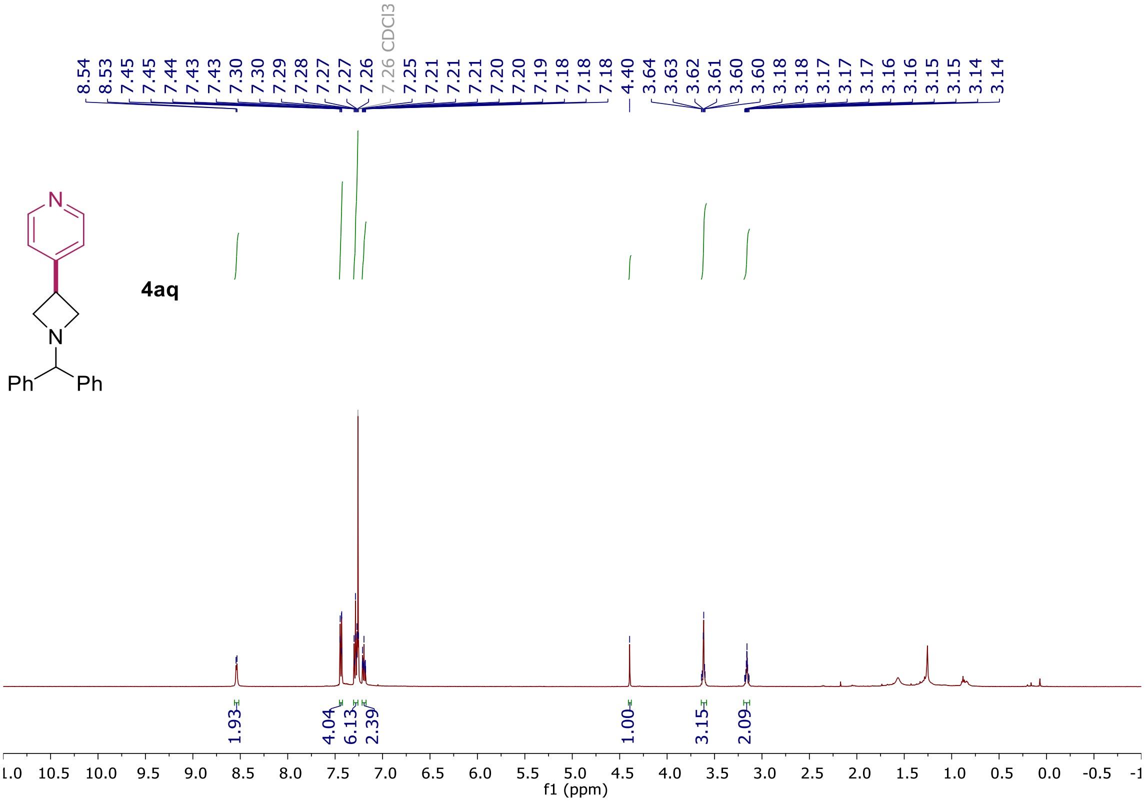

^{13}C-NMR (75 MHz, $CDCl_3$) of compound **4aq**

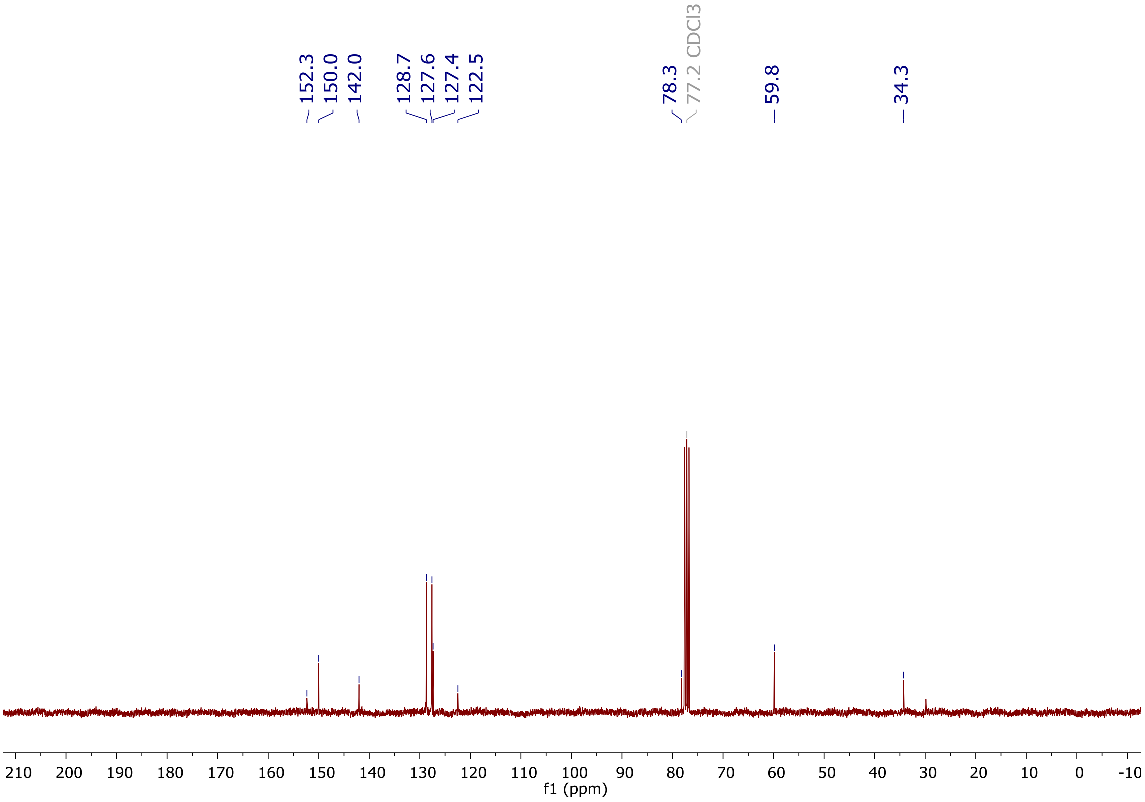

^{1}H-NMR (300 MHz, $CDCl_3$) of compound **4ar**

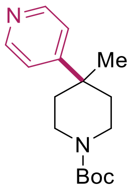

4ar

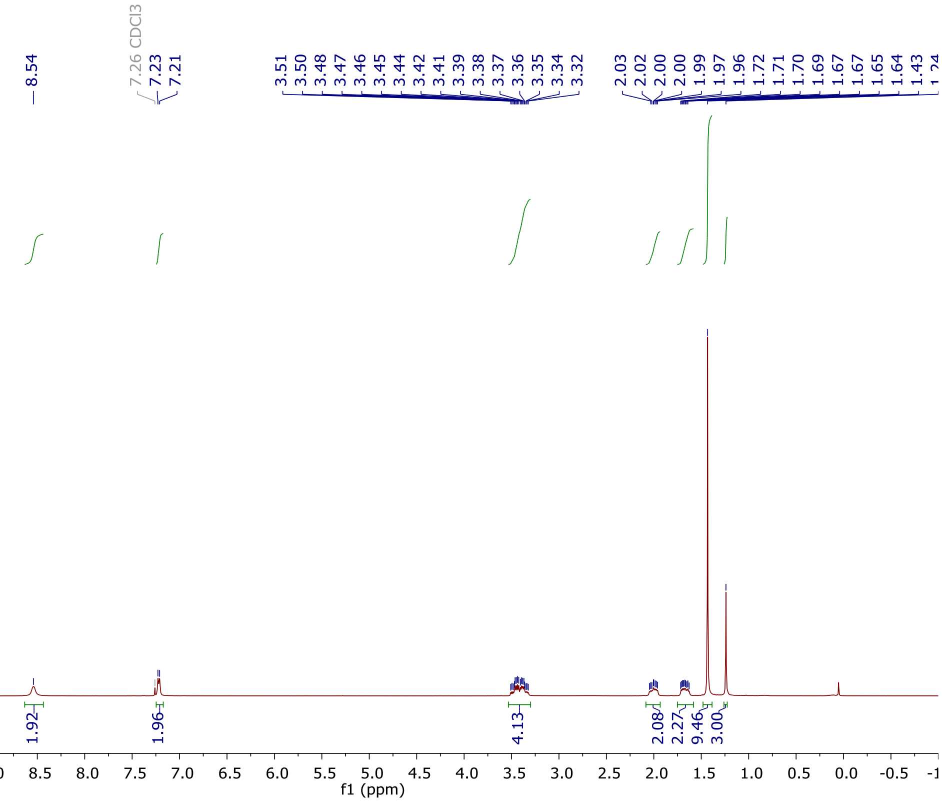

^{13}C-NMR (75 MHz, $CDCl_3$) of compound **4ar**

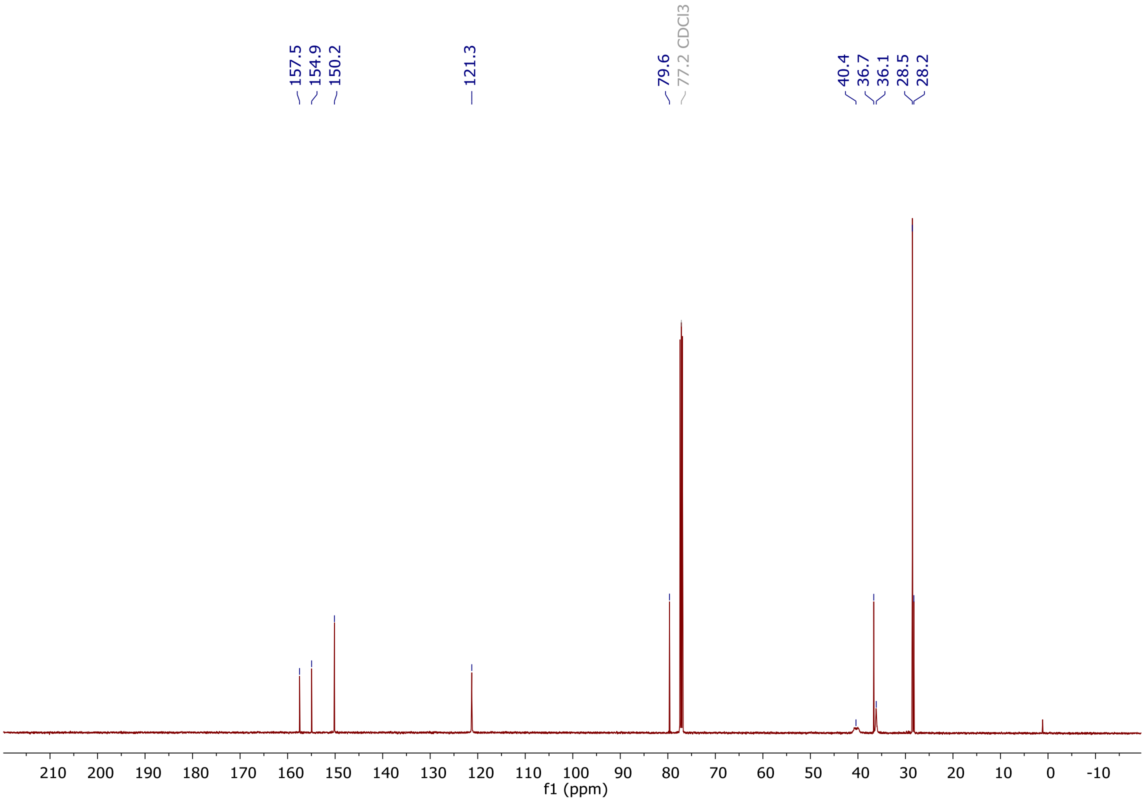

[1]**H-NMR** (300 MHz, $CDCl_3$) of compound **4as**

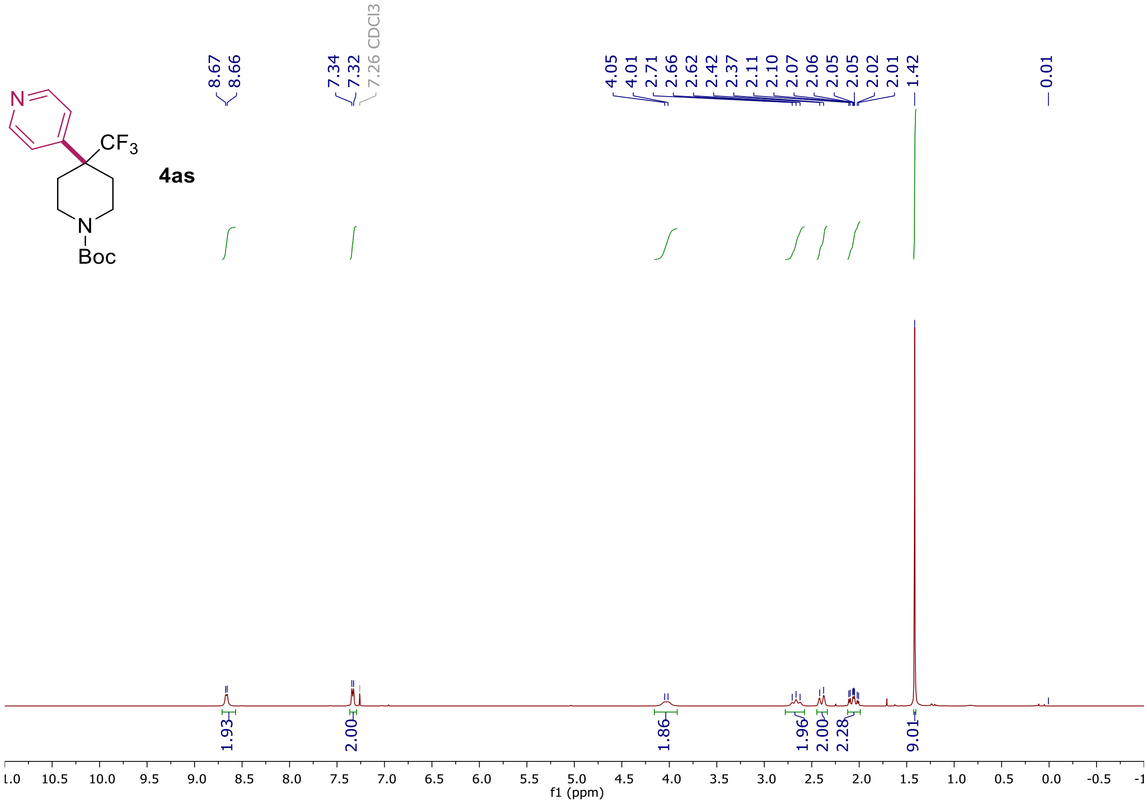

[13]**C-NMR** (75 MHz, $CDCl_3$) of compound **4as**

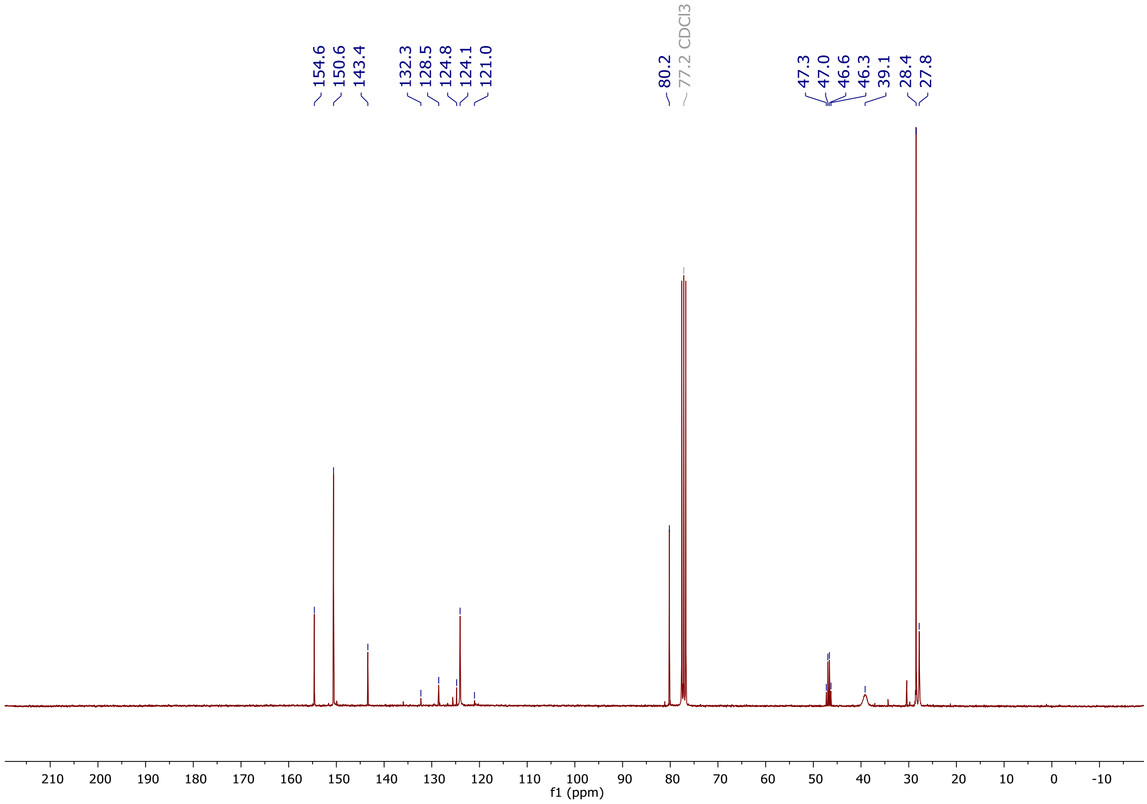

^{19}F-NMR (470 MHz, $CDCl_3$) of compound **4as**

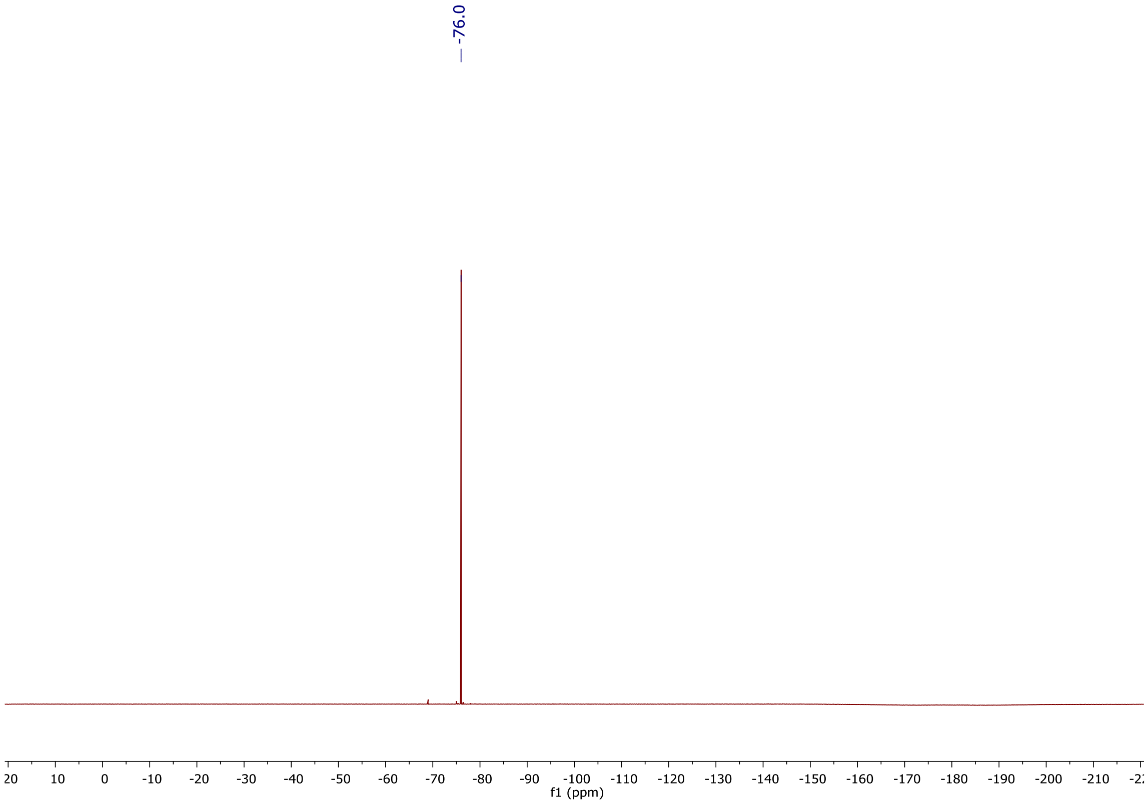

¹H-NMR (300 MHz, $CDCl_3$) of compound **4at**

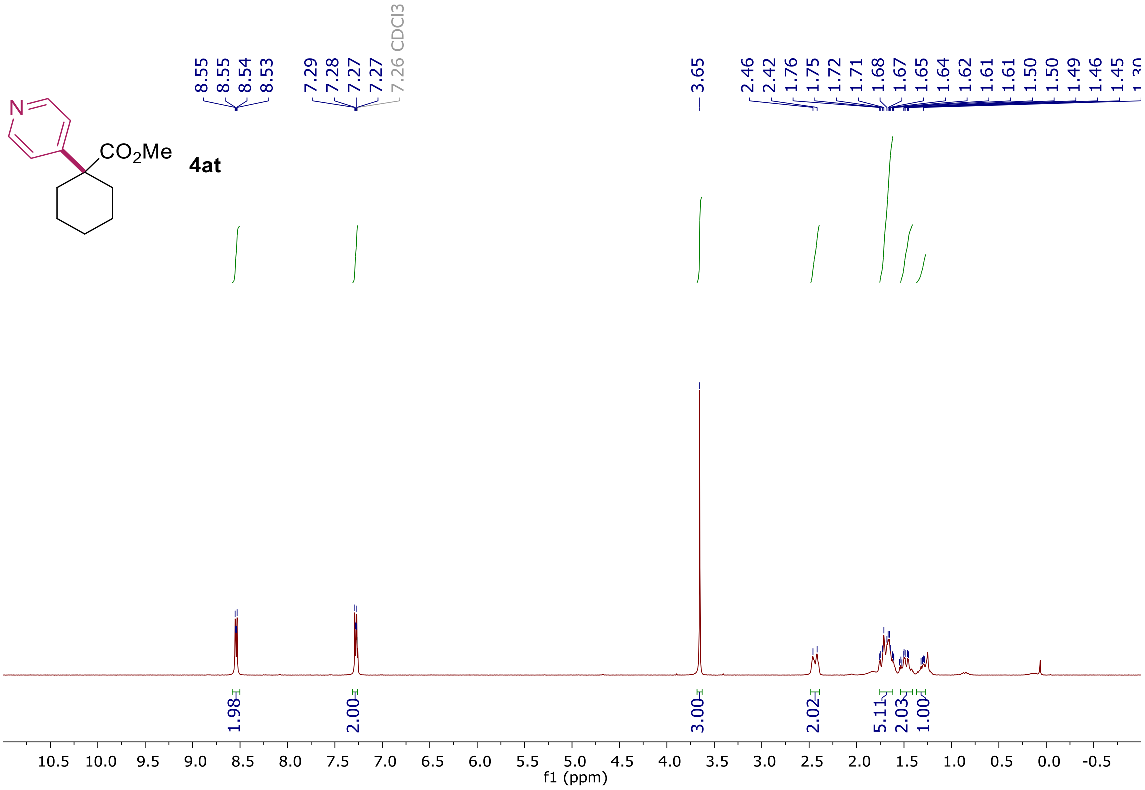

¹³C-NMR (75 MHz, $CDCl_3$) of compound **4at**

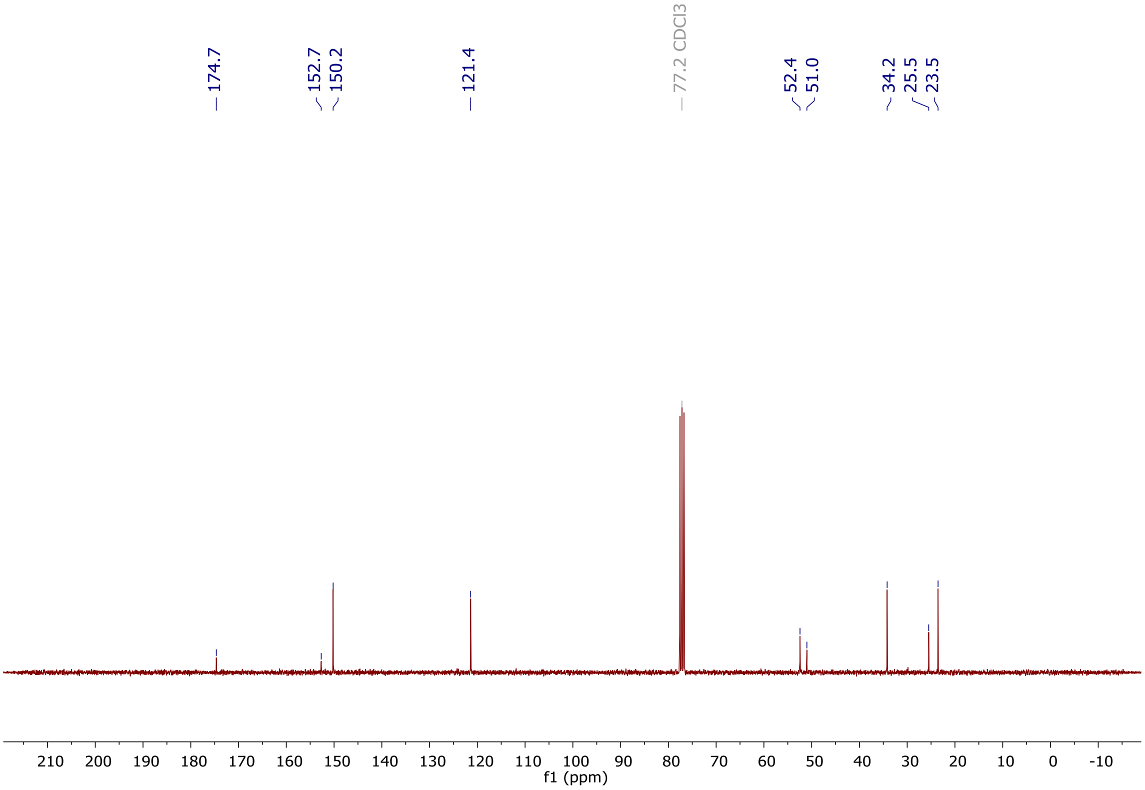

^{1}H-NMR (300 MHz, $CDCl_3$) of compound **4au**

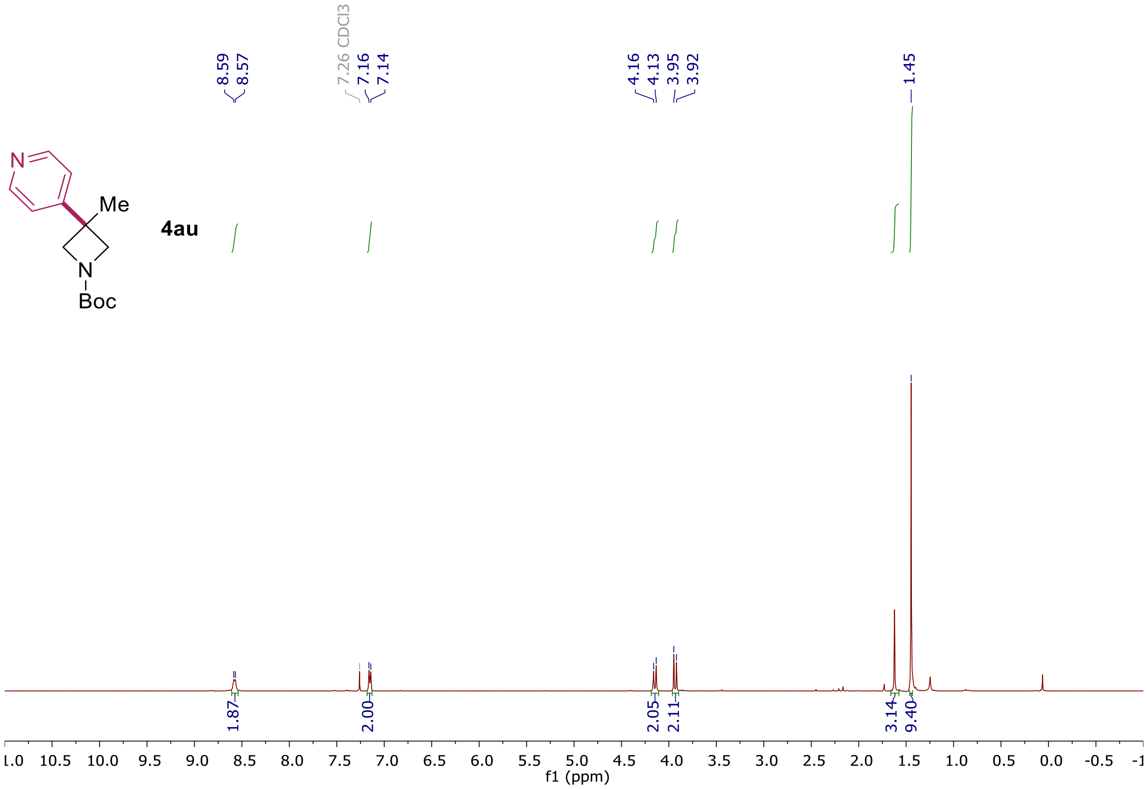

^{13}C-NMR (75 MHz, $CDCl_3$) of compound **4au**

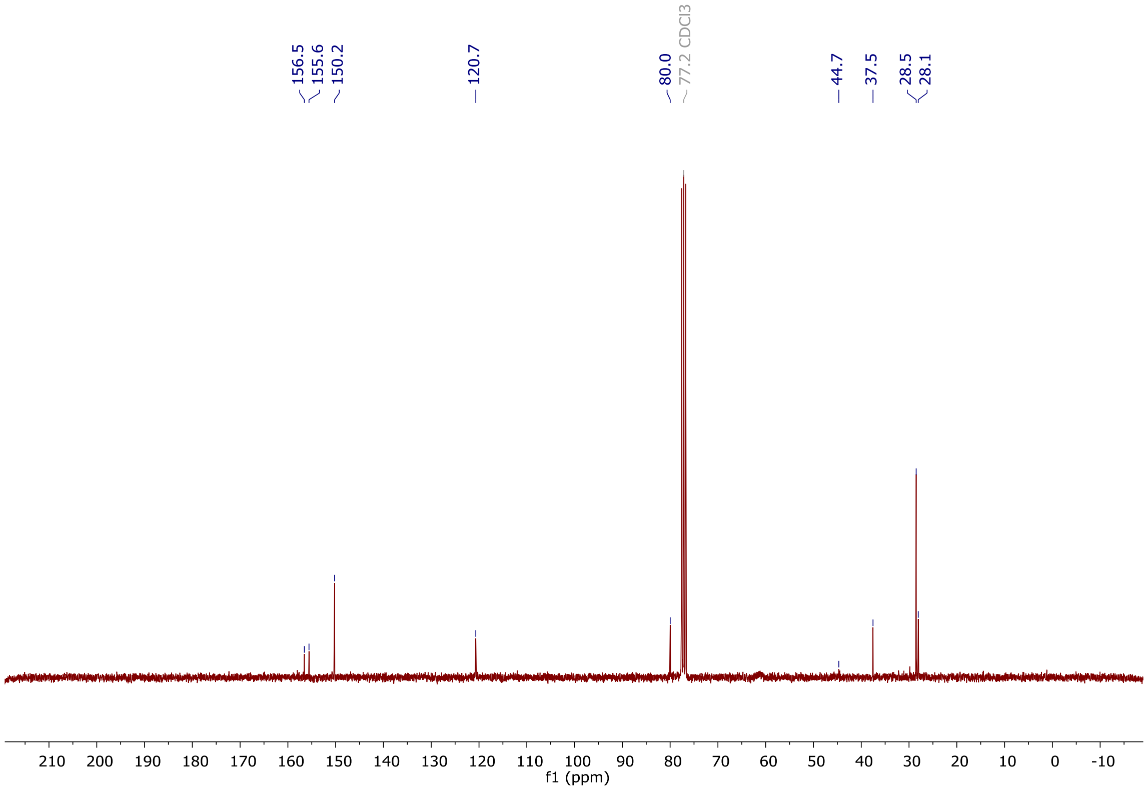

^{1}H-NMR (300 MHz, CDCl3) of compound **4av**

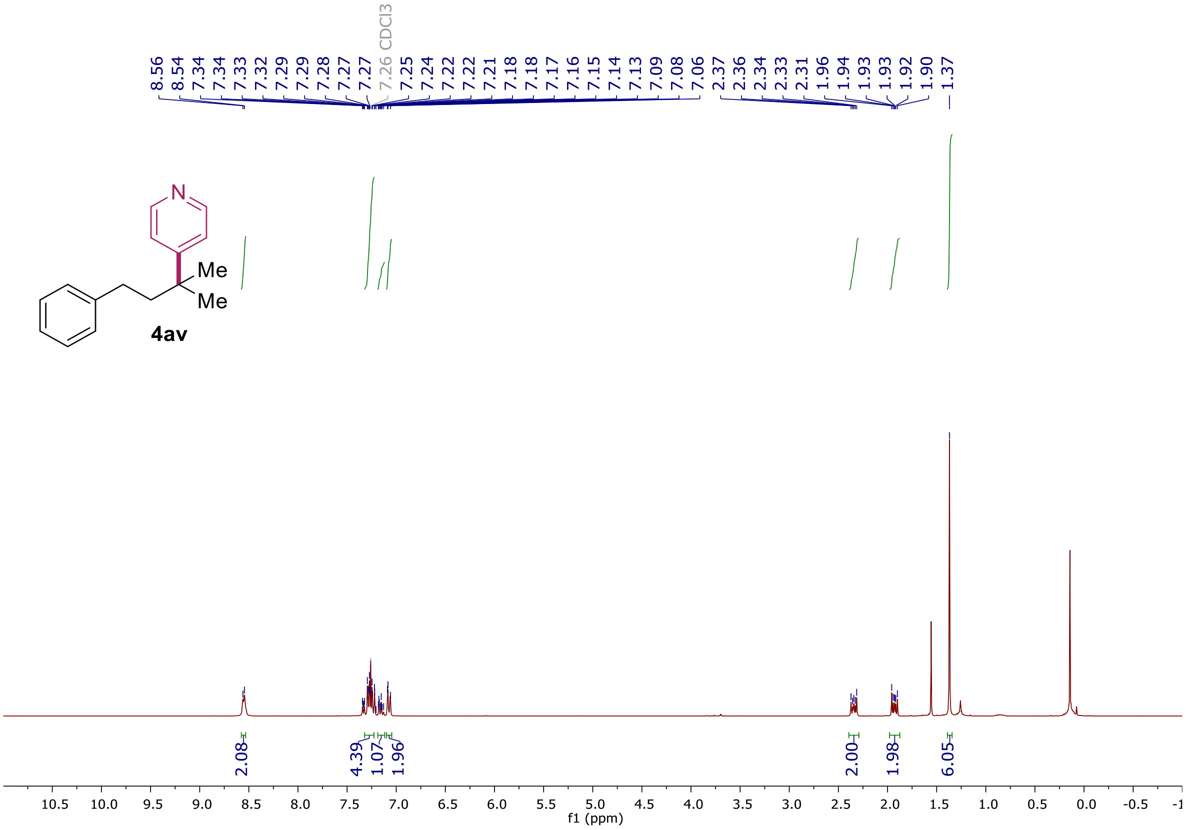

^{1}H-NMR (300 MHz, $CDCl_3$) of compound **4aw**

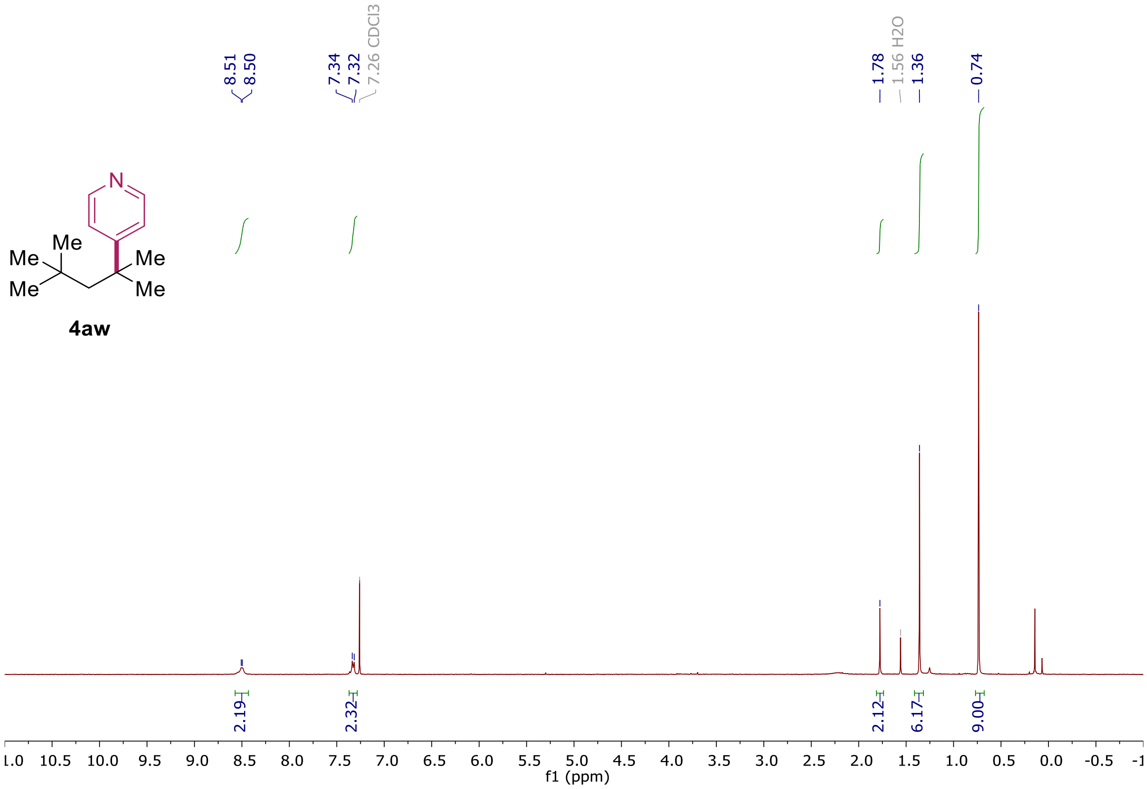

^{13}C-NMR (75 MHz, $CDCl_3$) of compound **4aw**

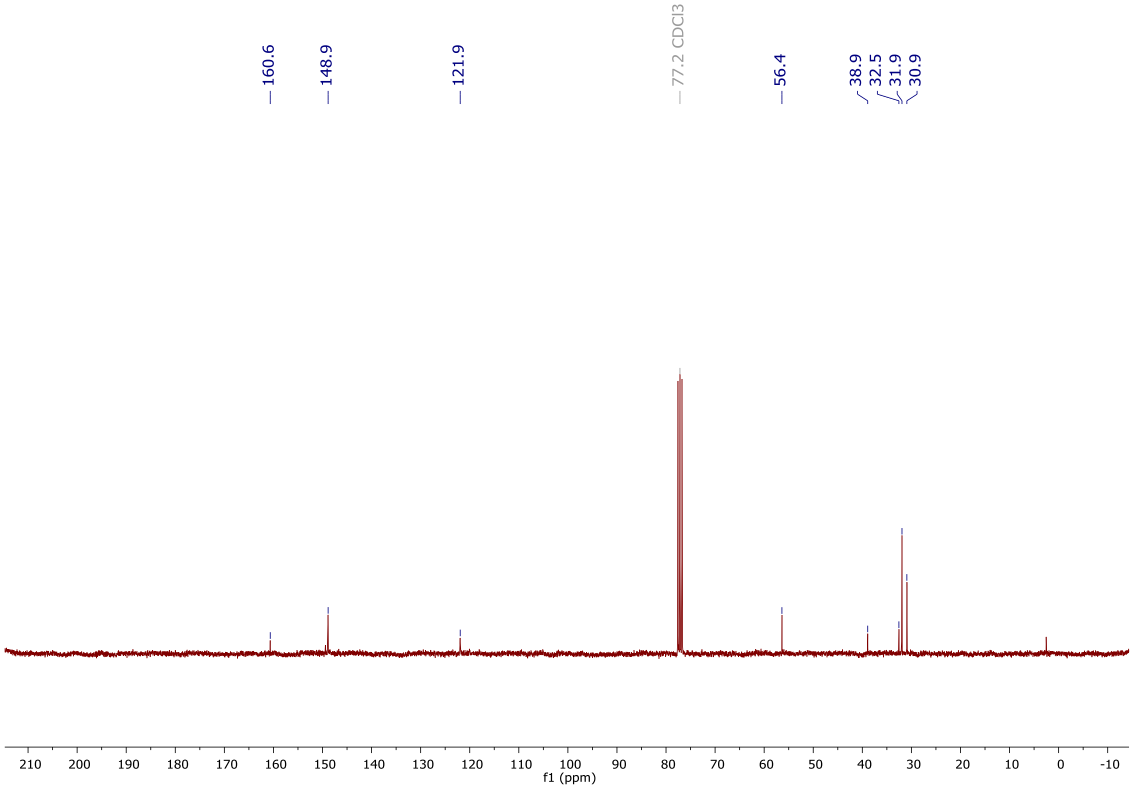

[1]**H-NMR** (300 MHz, $CDCl_3$) of compound **4ax**

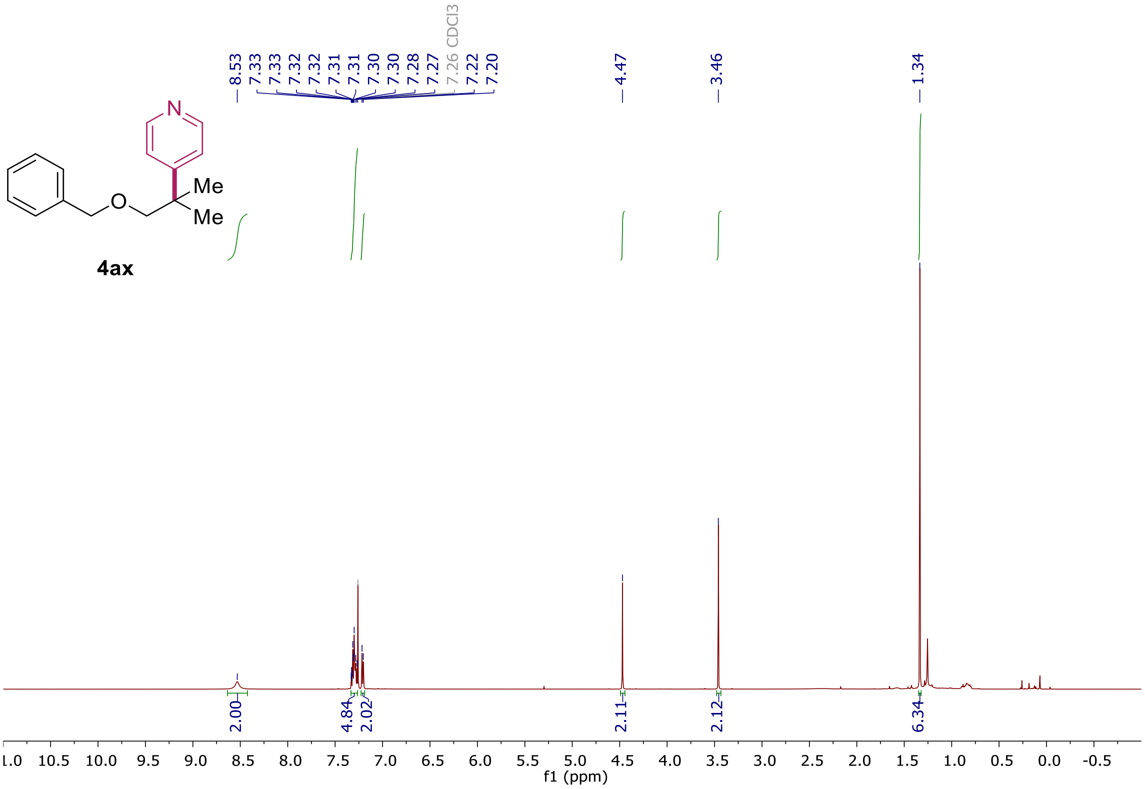

[13]**C-NMR** (75 MHz, $CDCl_3$) of compound **4ax**

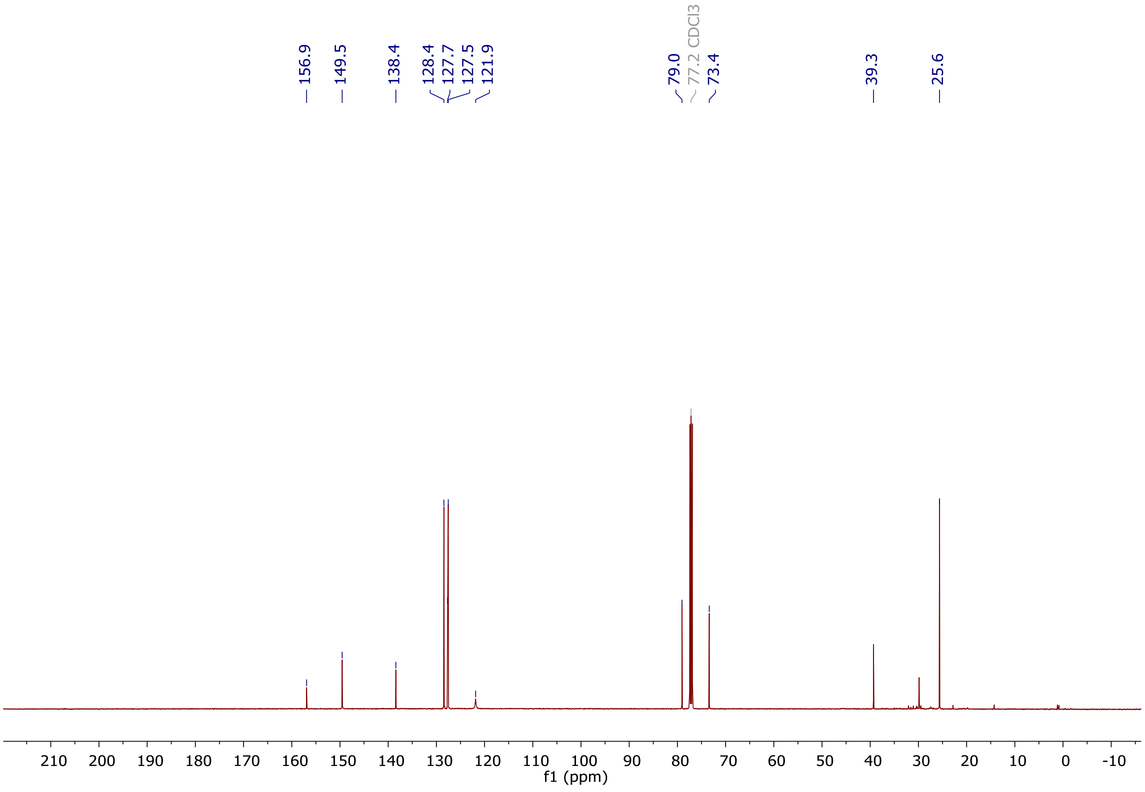

¹H-NMR (300 MHz, $CDCl_3$) of compound **4ay**

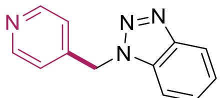

4ay

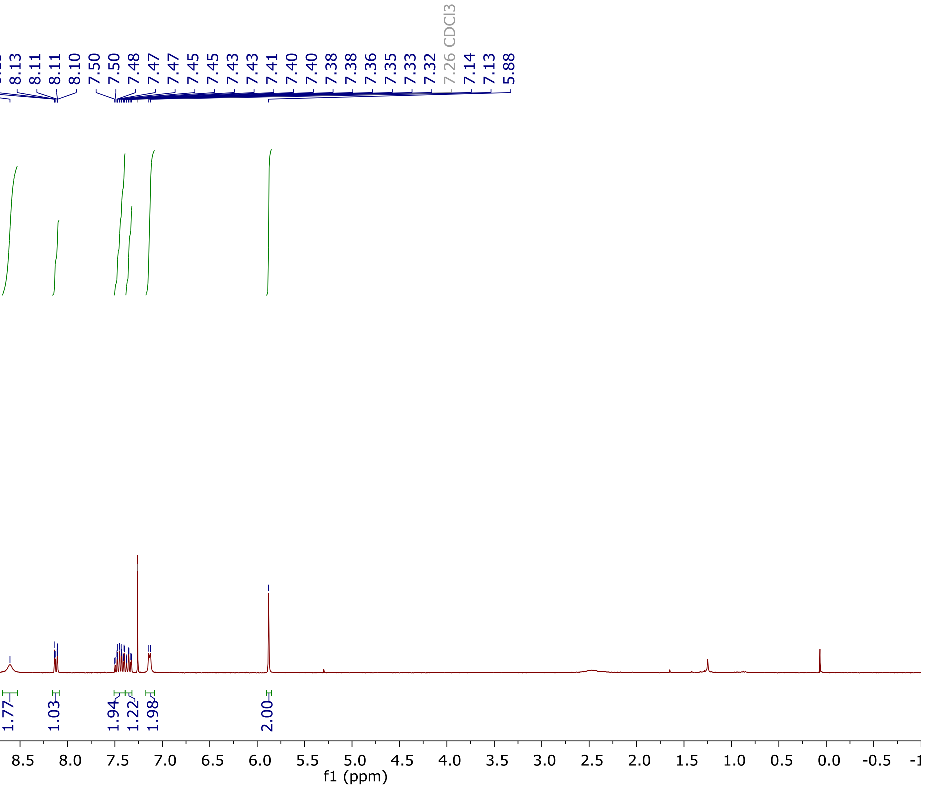

[1]**H-NMR** (300 MHz, CDCl$_3$) of compound **4az**

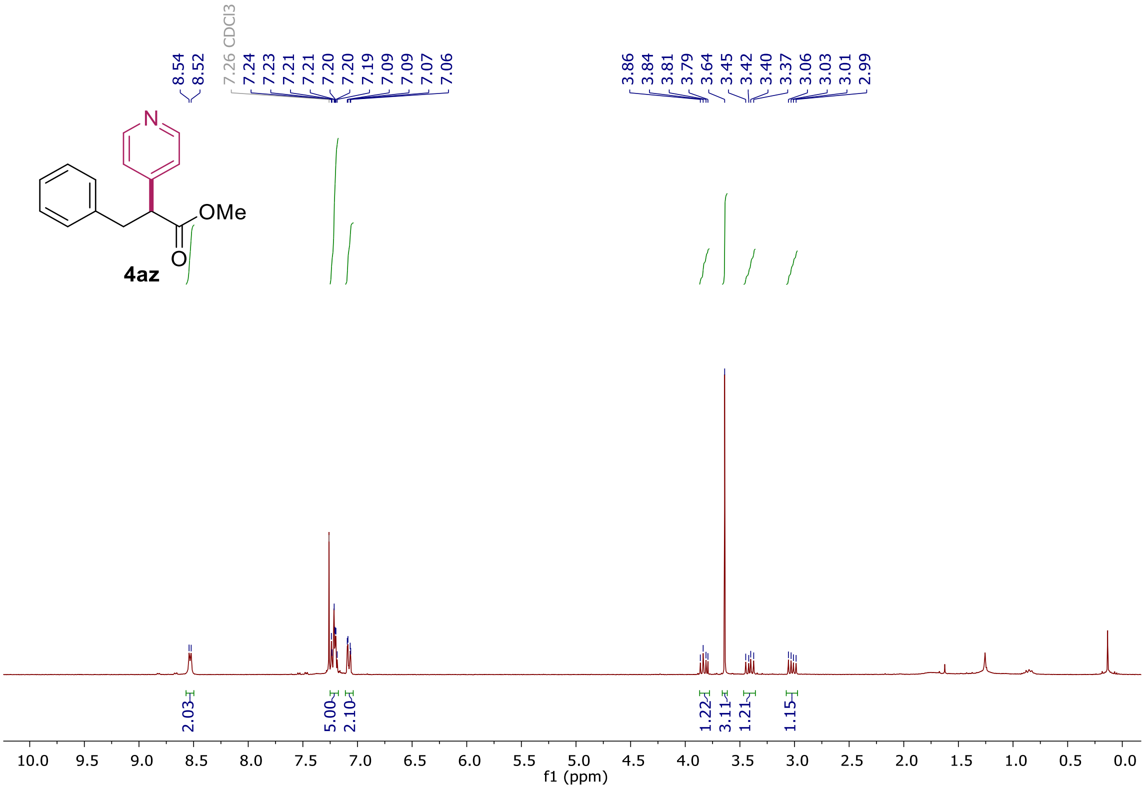

[13]**C-NMR** (75 MHz, CDCl$_3$) of compound **4az**

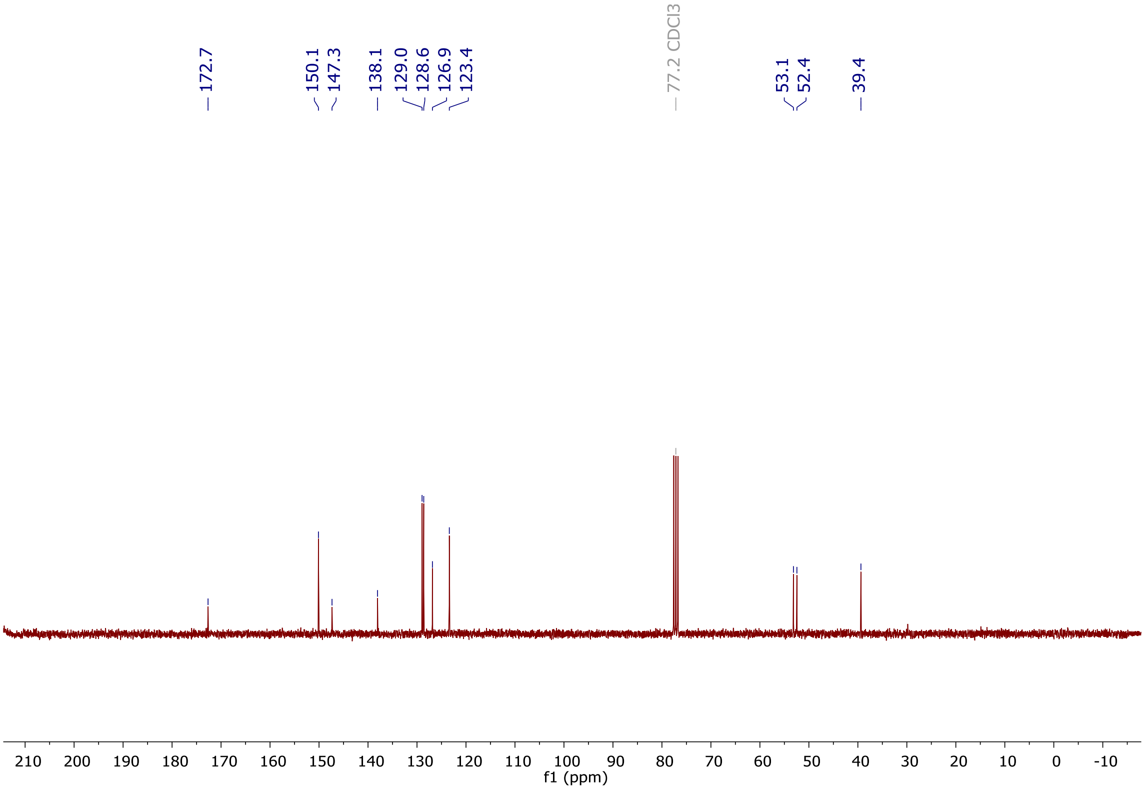

^{1}H-NMR (300 MHz, $CDCl_3$) of compound **4ba**

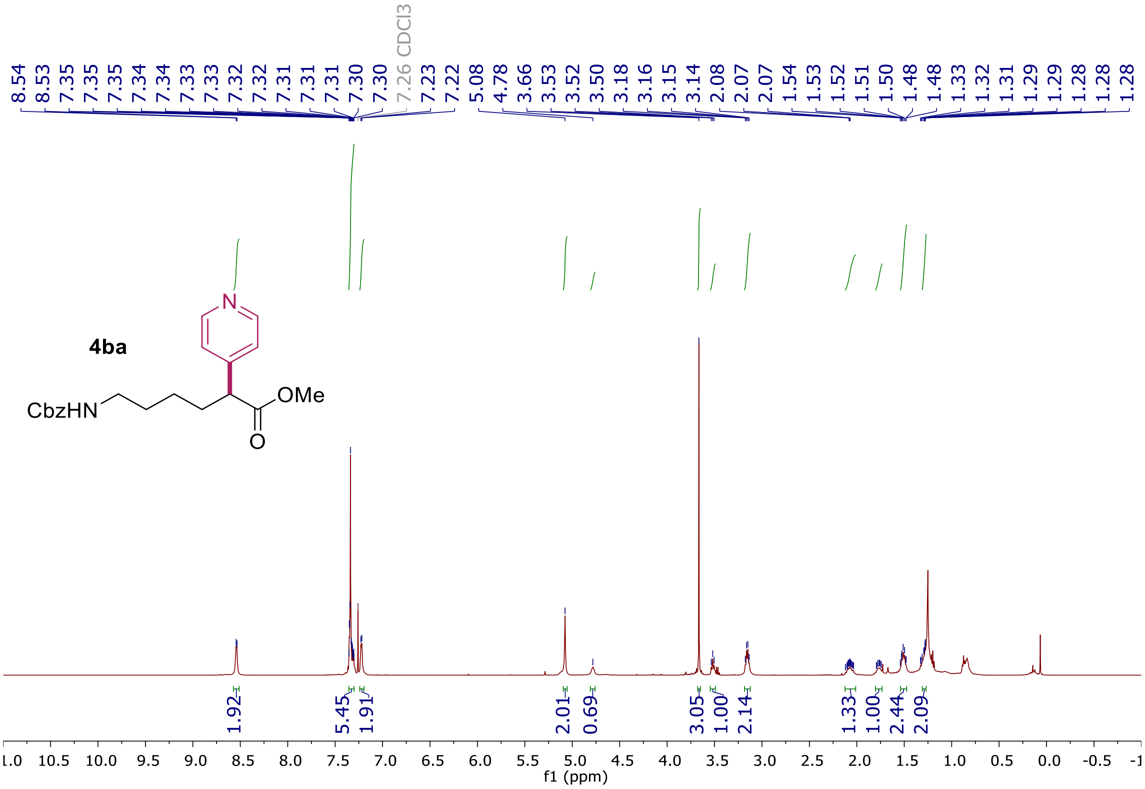

^{13}C-NMR (75 MHz, $CDCl_3$) of compound **4ba**

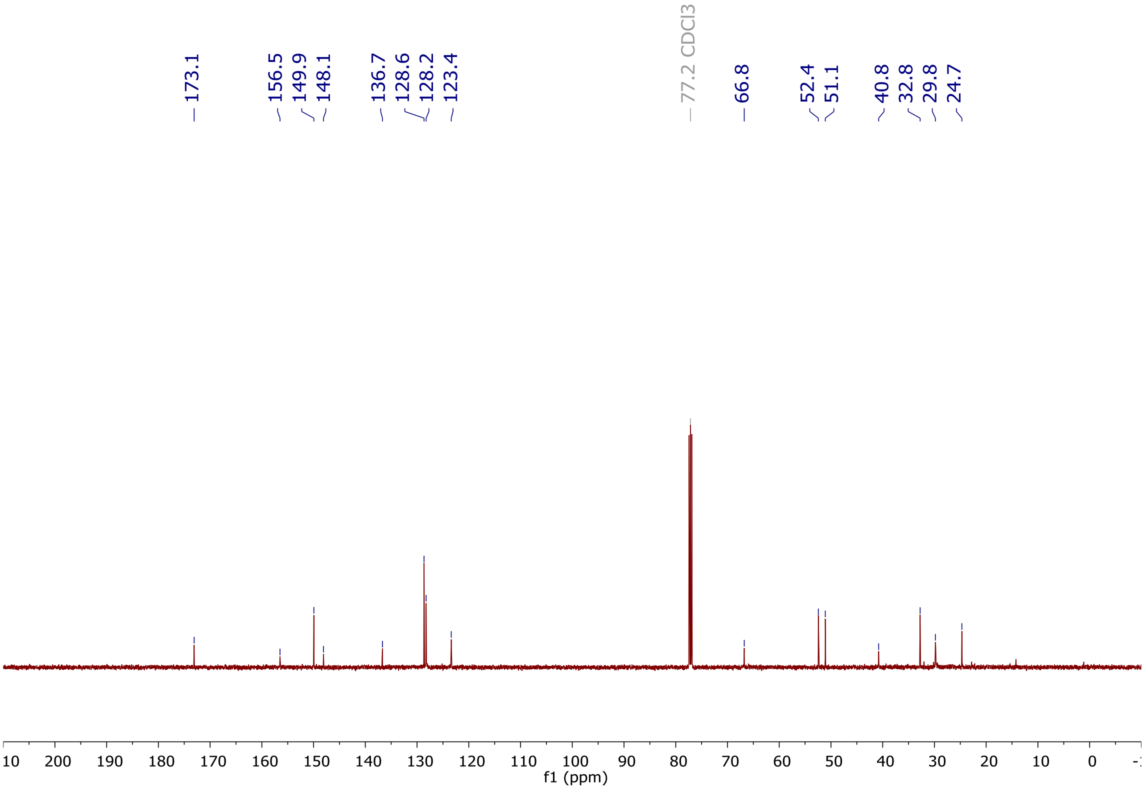

¹H-NMR (300 MHz, $CDCl_3$) of compound **4bb**

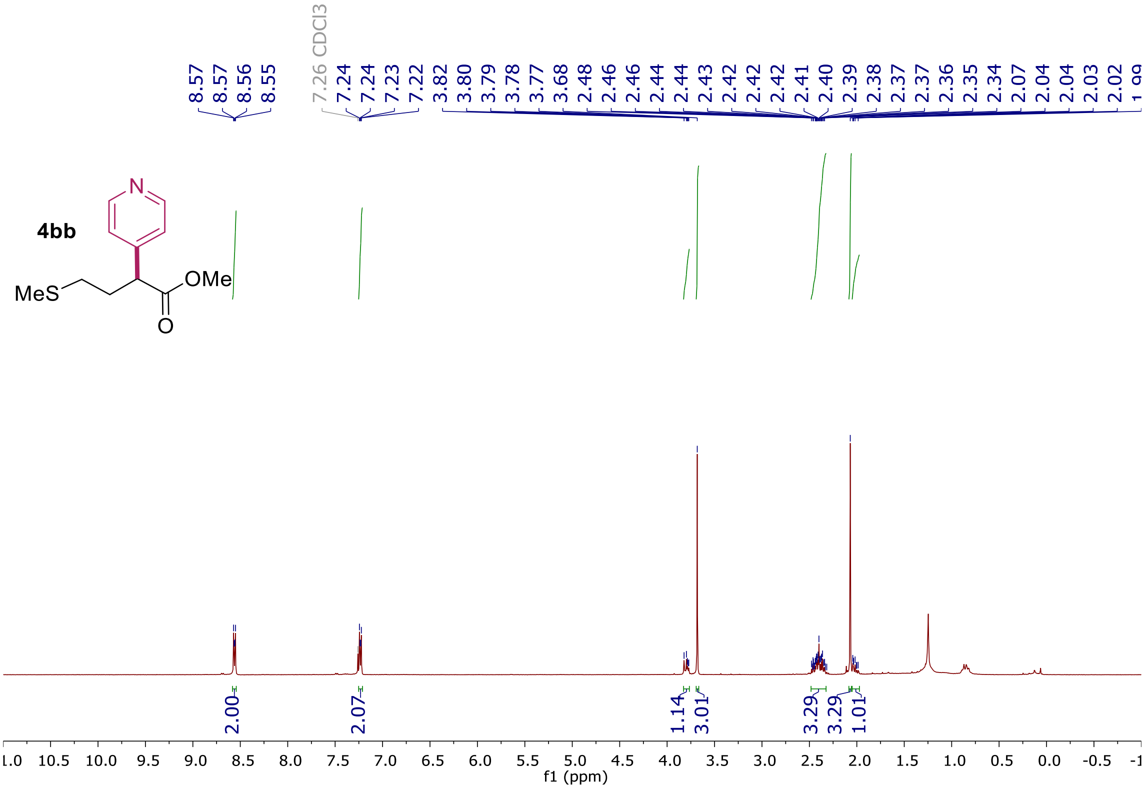

¹³C-NMR (75 MHz, $CDCl_3$) of compound **4bb**

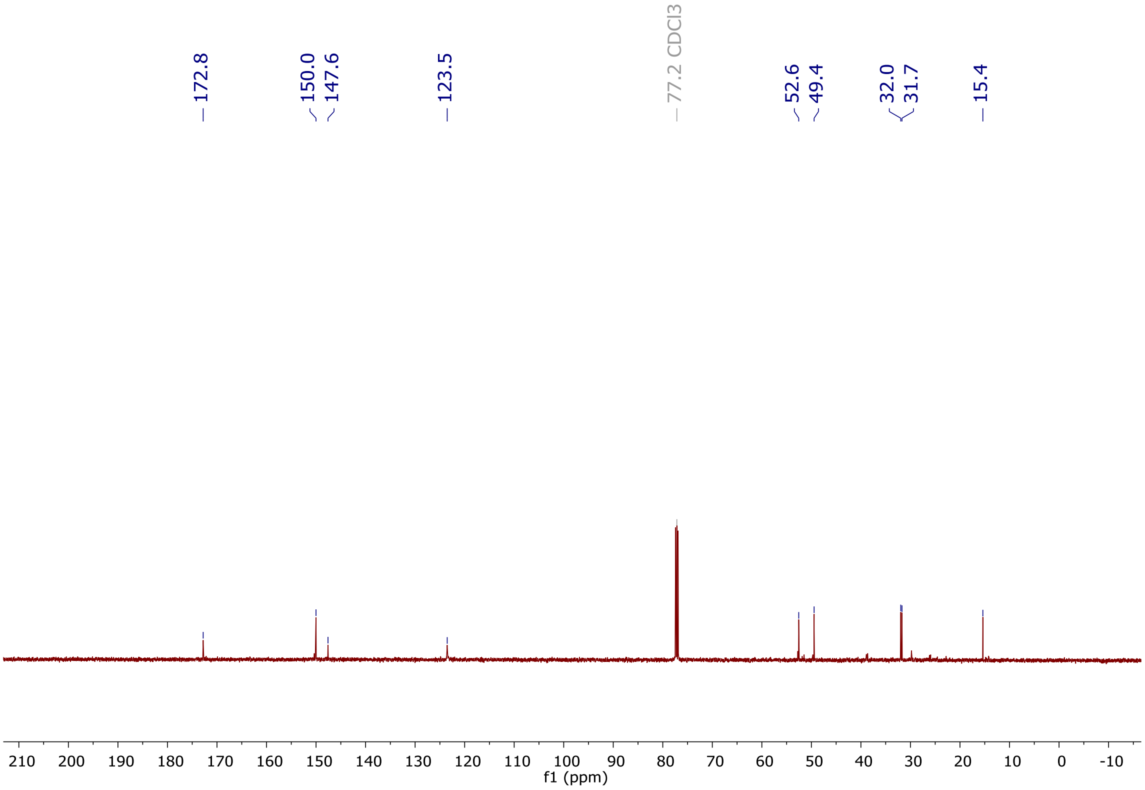

[1]**H-NMR** (300 MHz, $CDCl_3$) of compound **4bc**

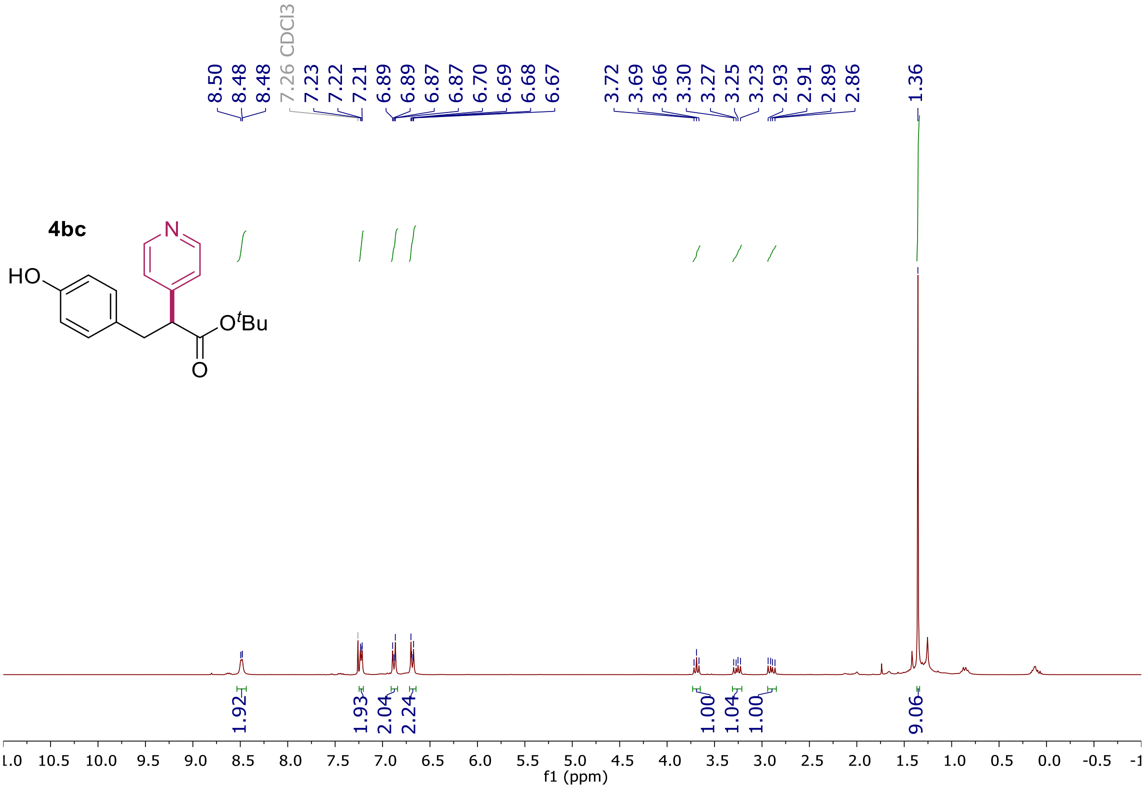

[13]**C-NMR** (75 MHz, $CDCl_3$) of compound **4bc**

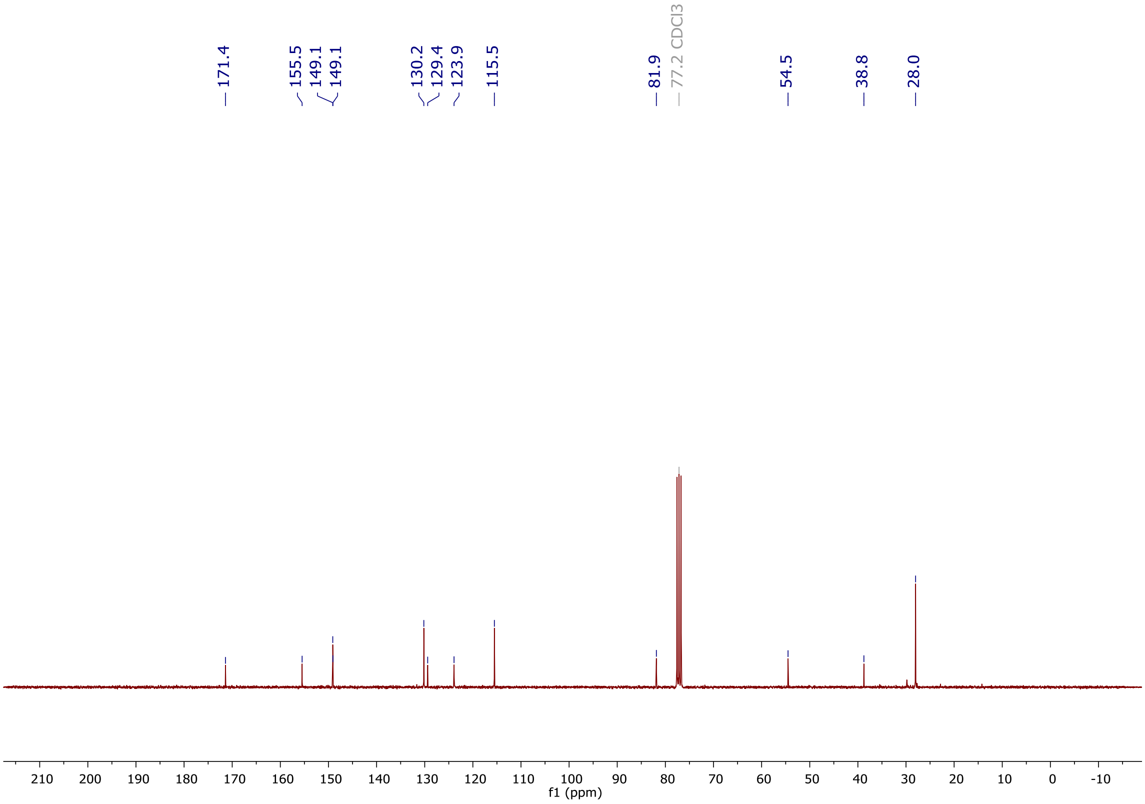

[1]**H-NMR** (300 MHz, $CDCl_3$) of compound **4bd**

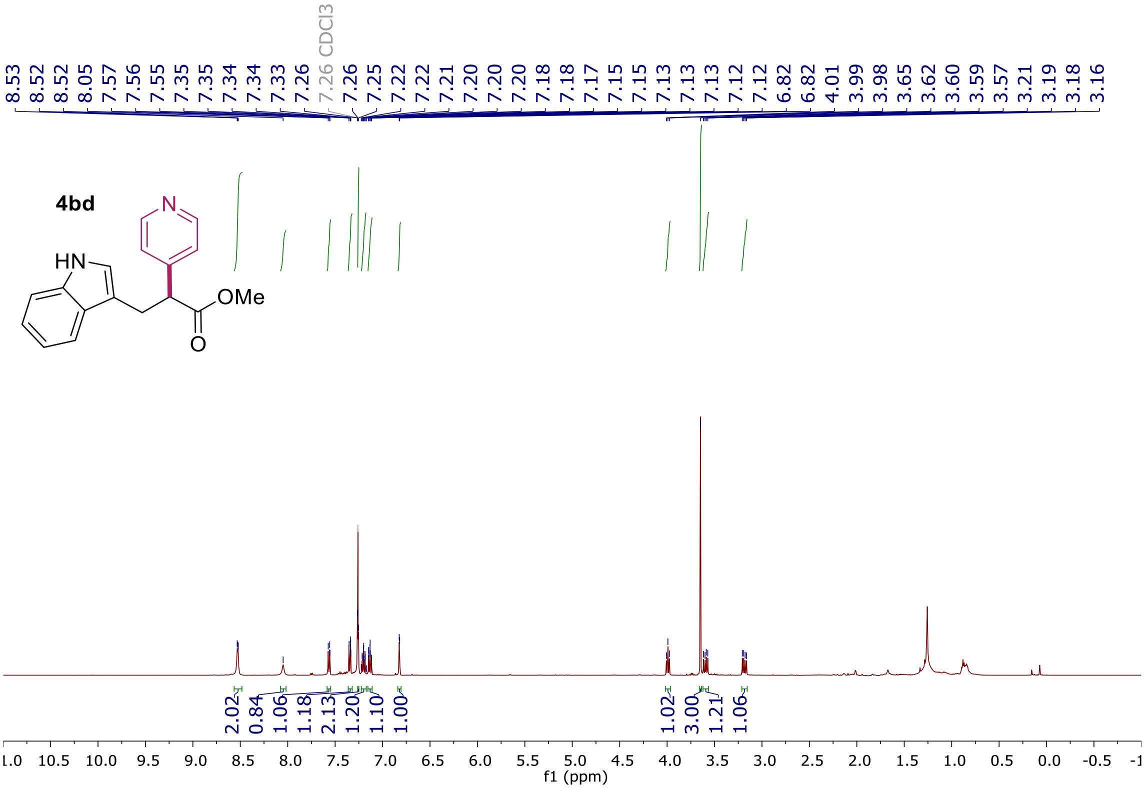

[13]**C-NMR** (75 MHz, $CDCl_3$) of compound **4bd**

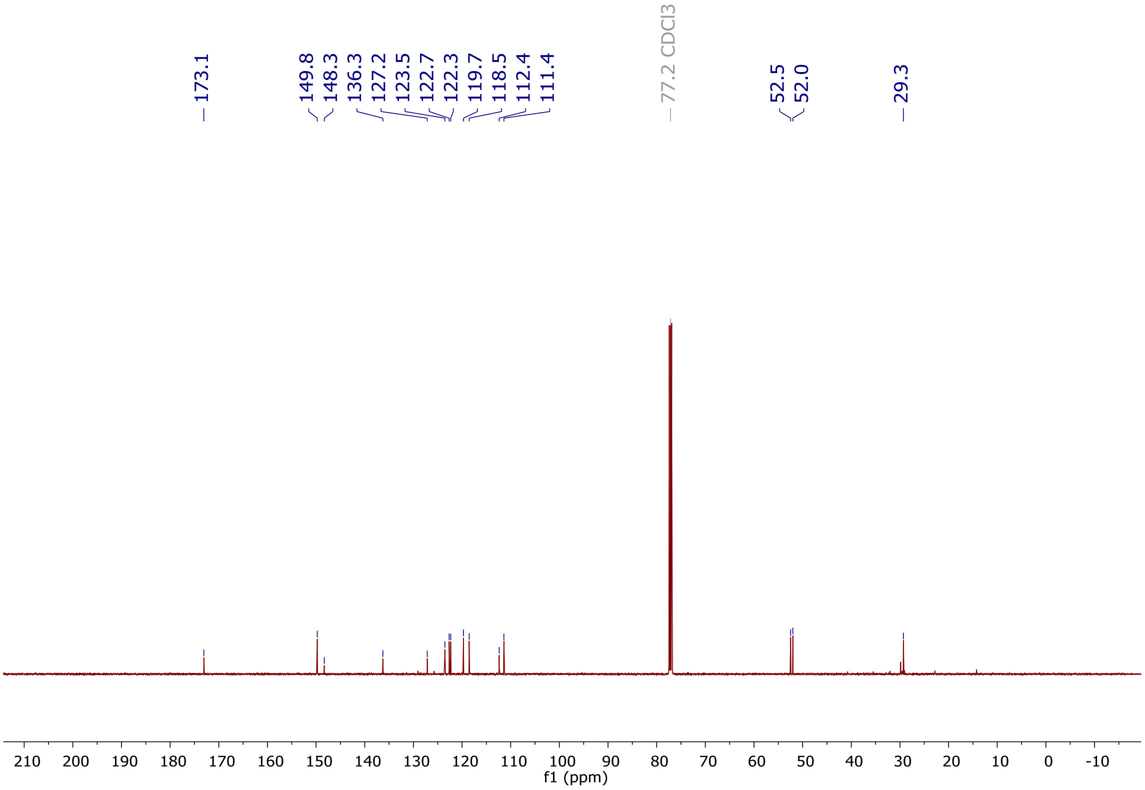

¹H-NMR (300 MHz, $CDCl_3$) of compound **4be**

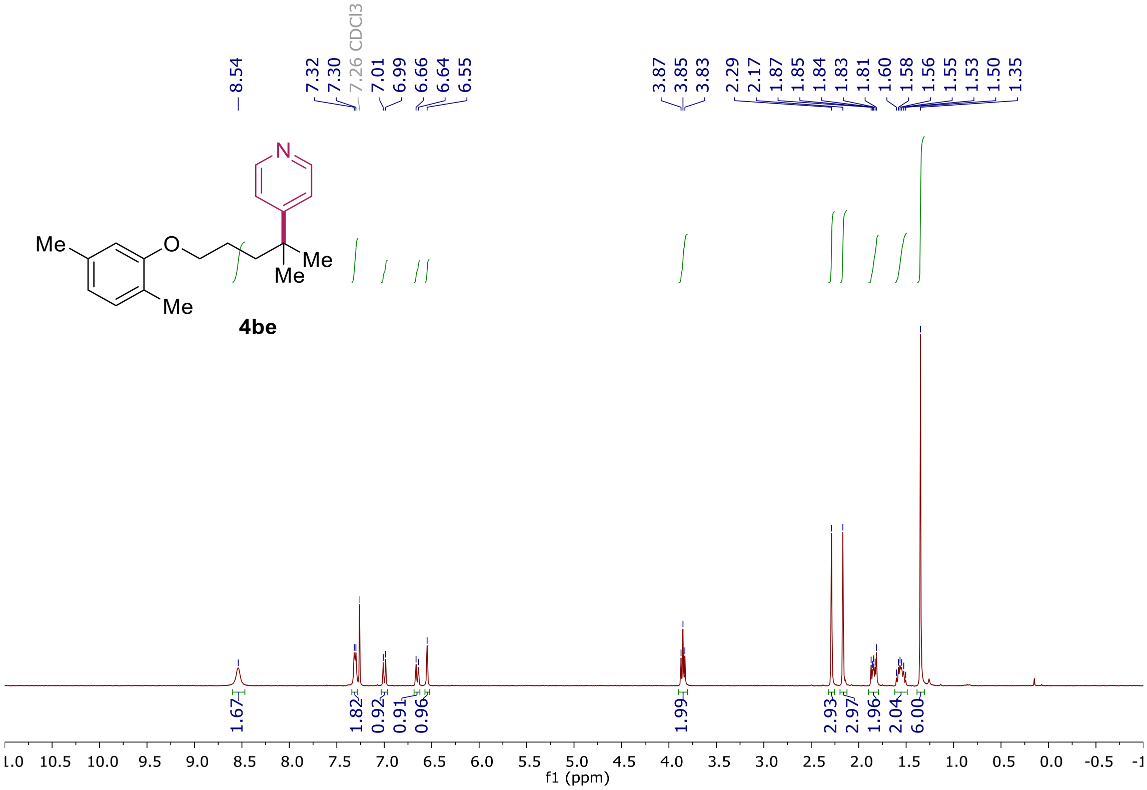

¹H-NMR (300 MHz, $CDCl_3$) of compound **4bf**

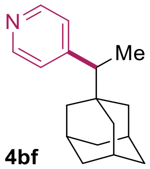

4bf

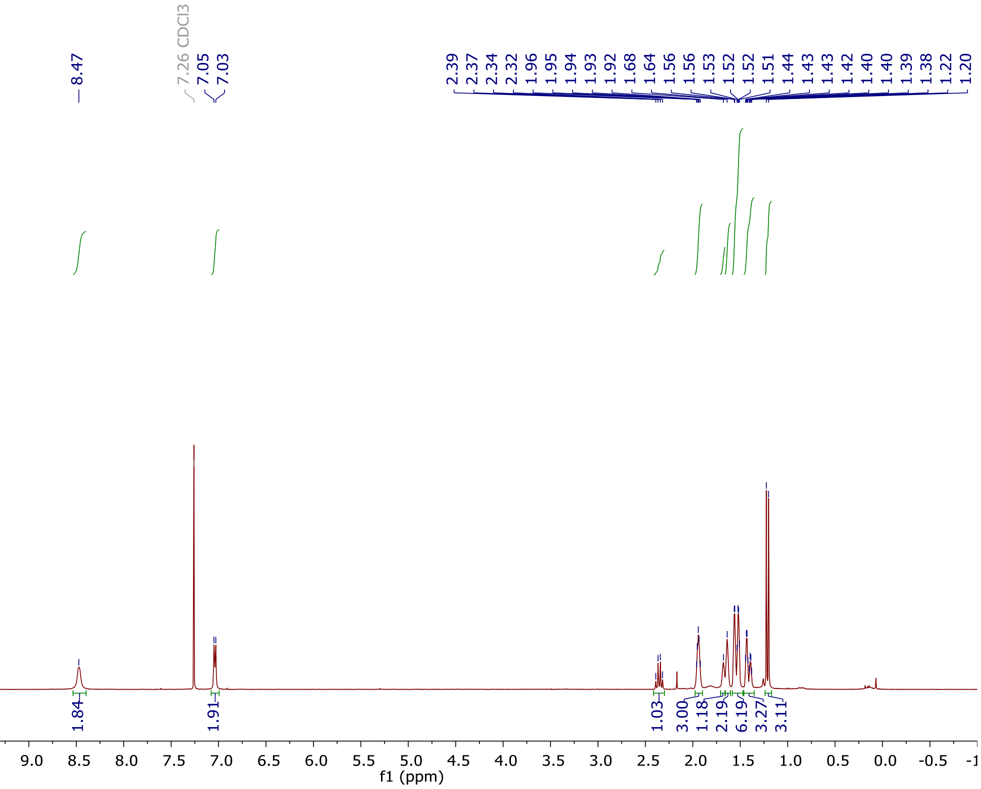

¹³C-NMR (75 MHz, $CDCl_3$) of compound **4bf**

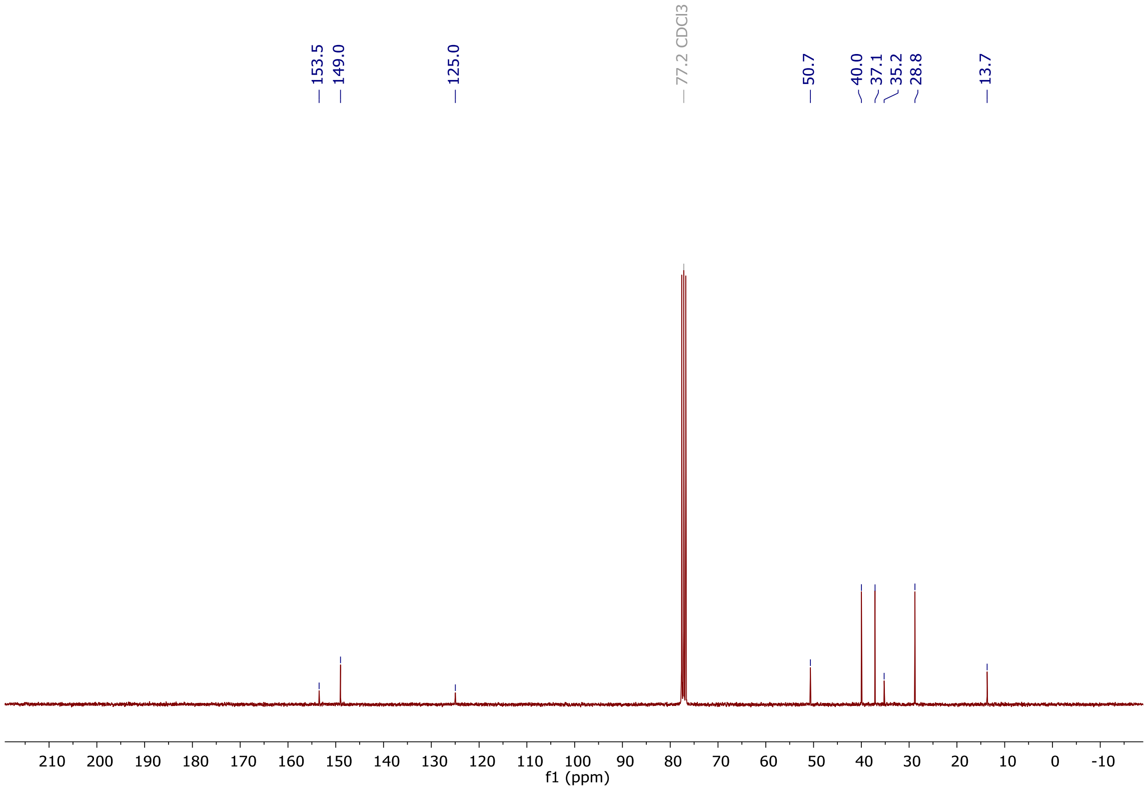

^{1}H-NMR (300 MHz, $CDCl_3$) of compound **4bg**

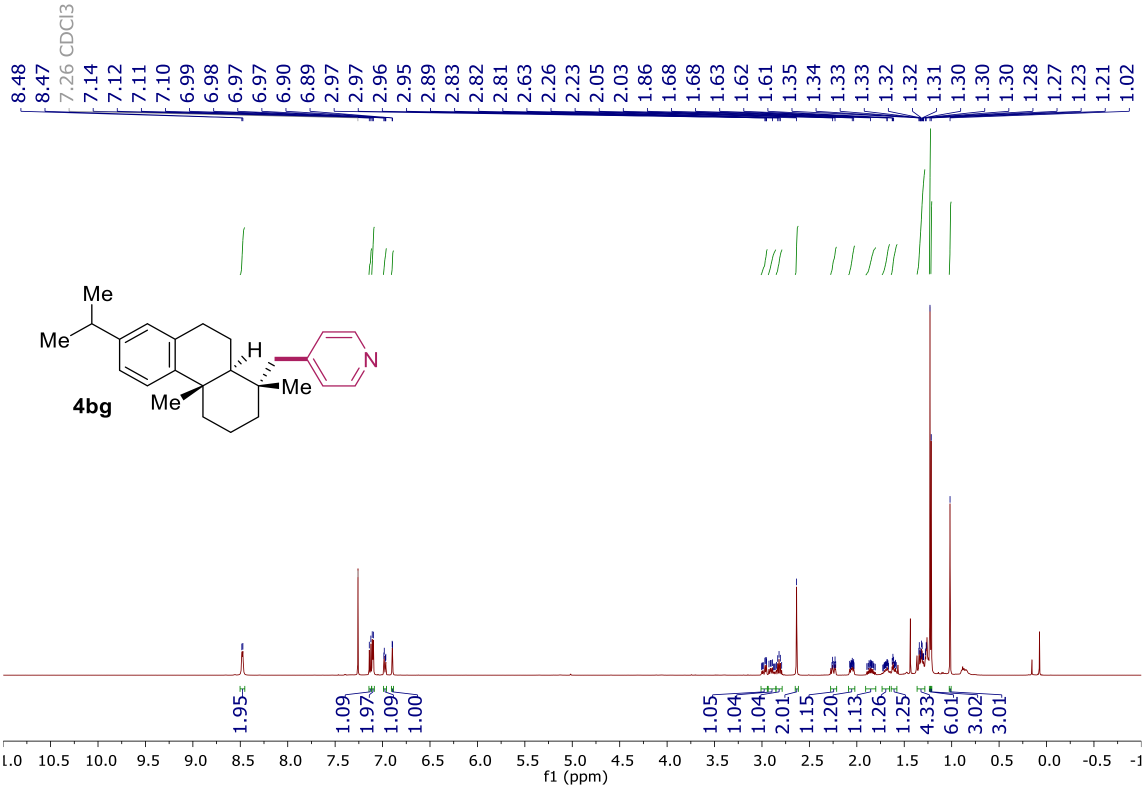

^{13}C-NMR (75 MHz, $CDCl_3$) of compound **4bg**

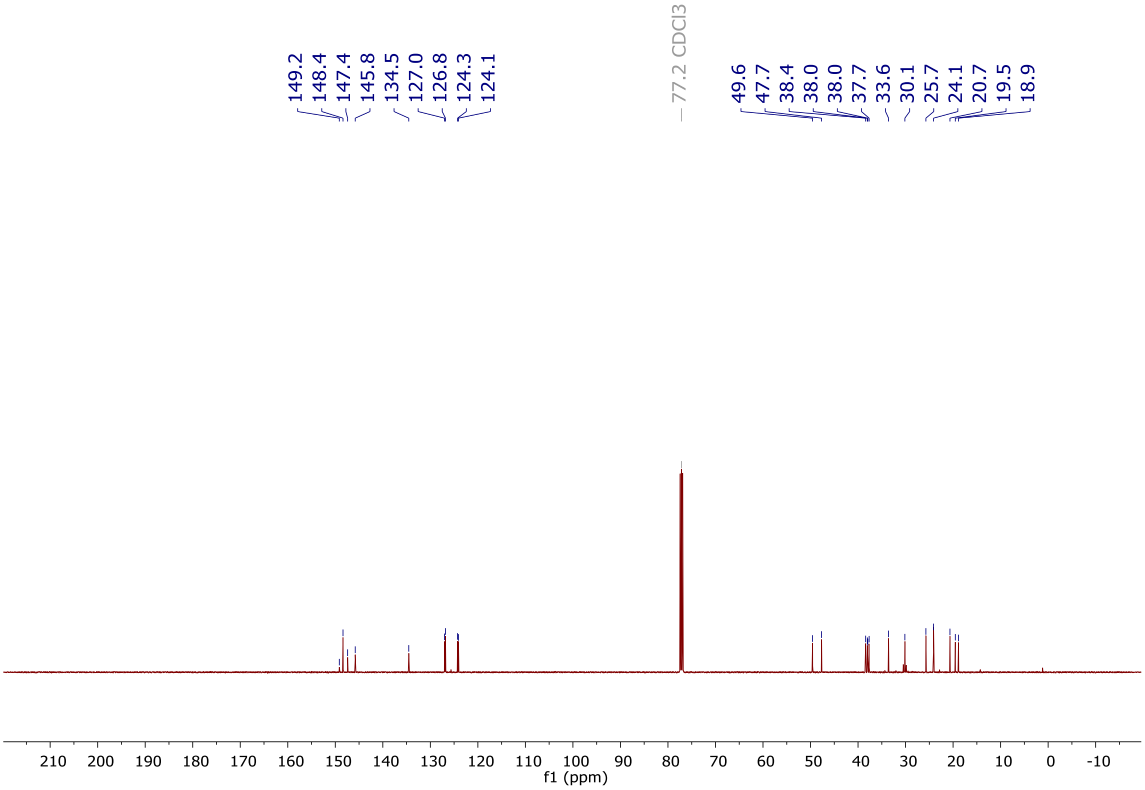

^{1}H-NMR (300 MHz, $C_2D_2Cl_4$) of compound **4bh, T = 27 ºC**

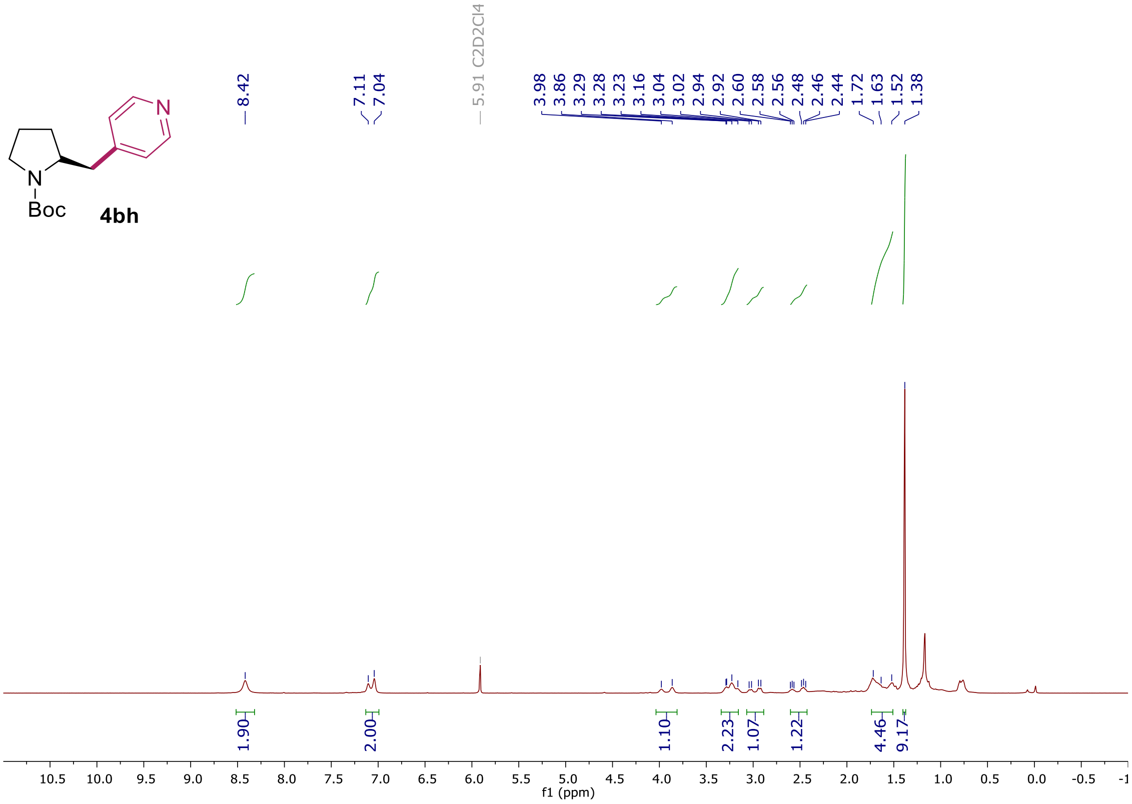

^{1}H-NMR (300 MHz, $C_2D_2Cl_4$) of compound **4bh, T = 90 ºC**

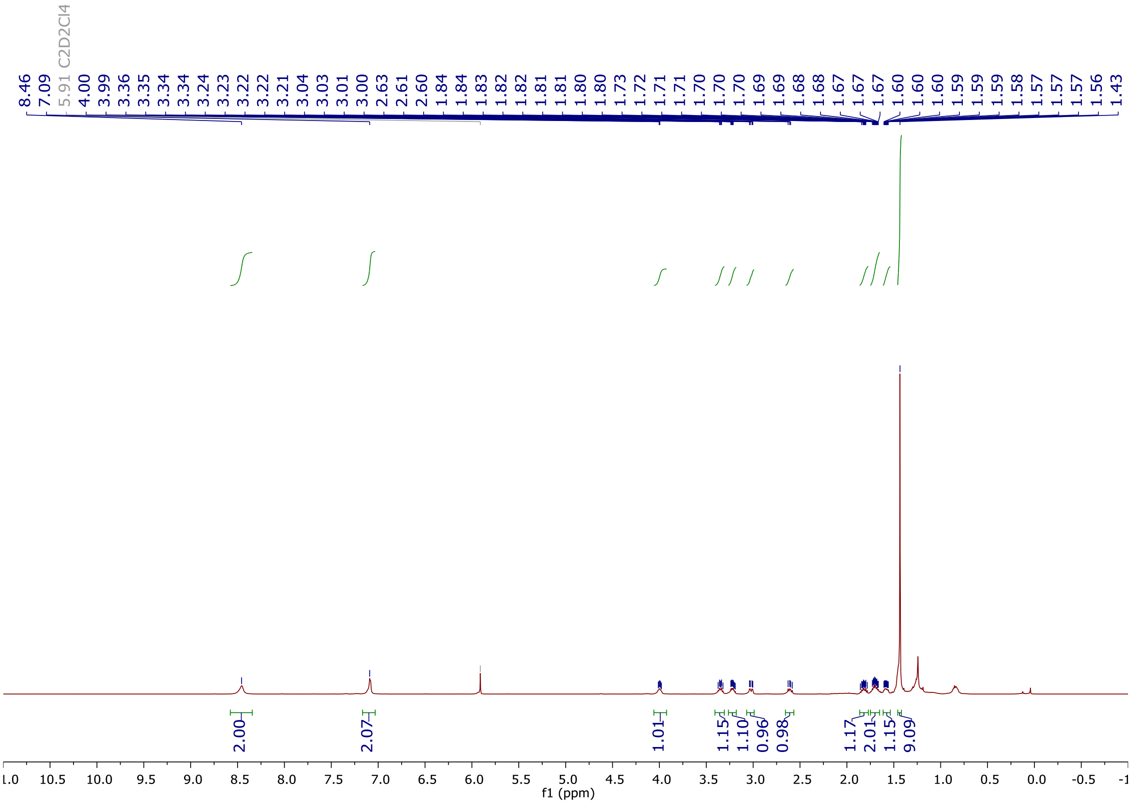

^{13}C-NMR (75 MHz, $C_2D_2Cl_4$) of compound **4bh, T = 90 ºC**

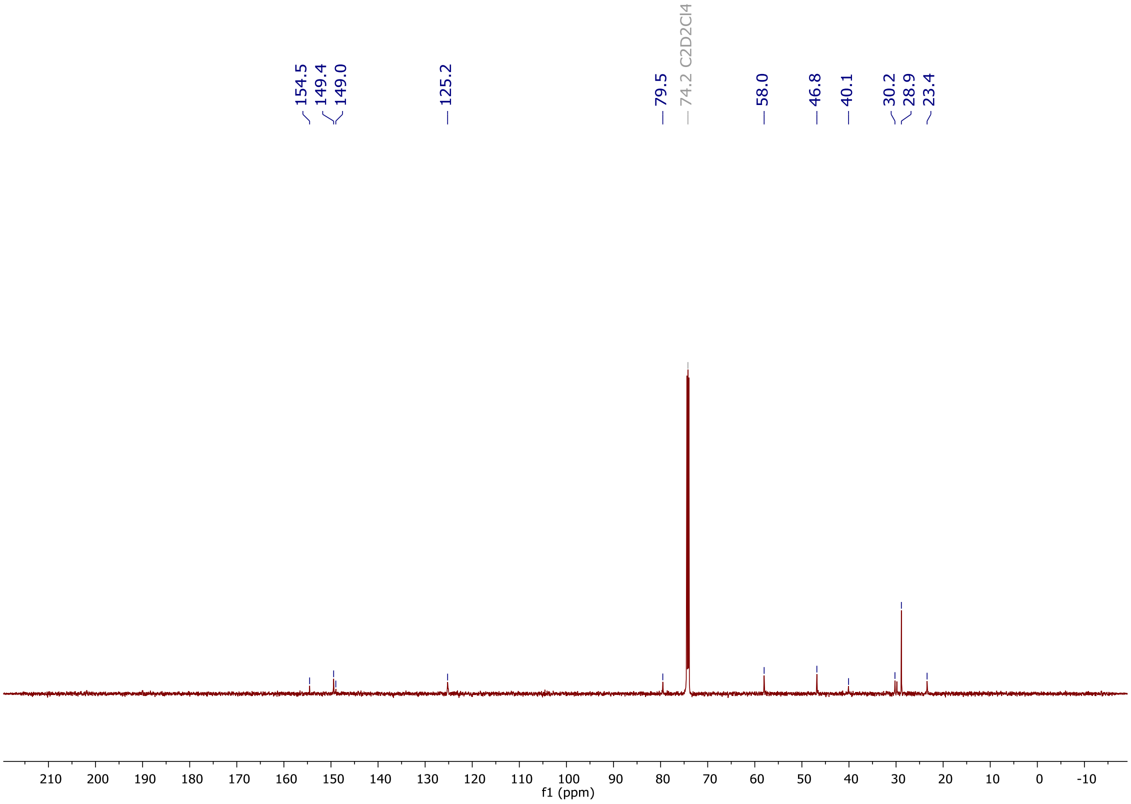

[1]**H-NMR** (300 MHz, $CDCl_3$) of compound **4bi**

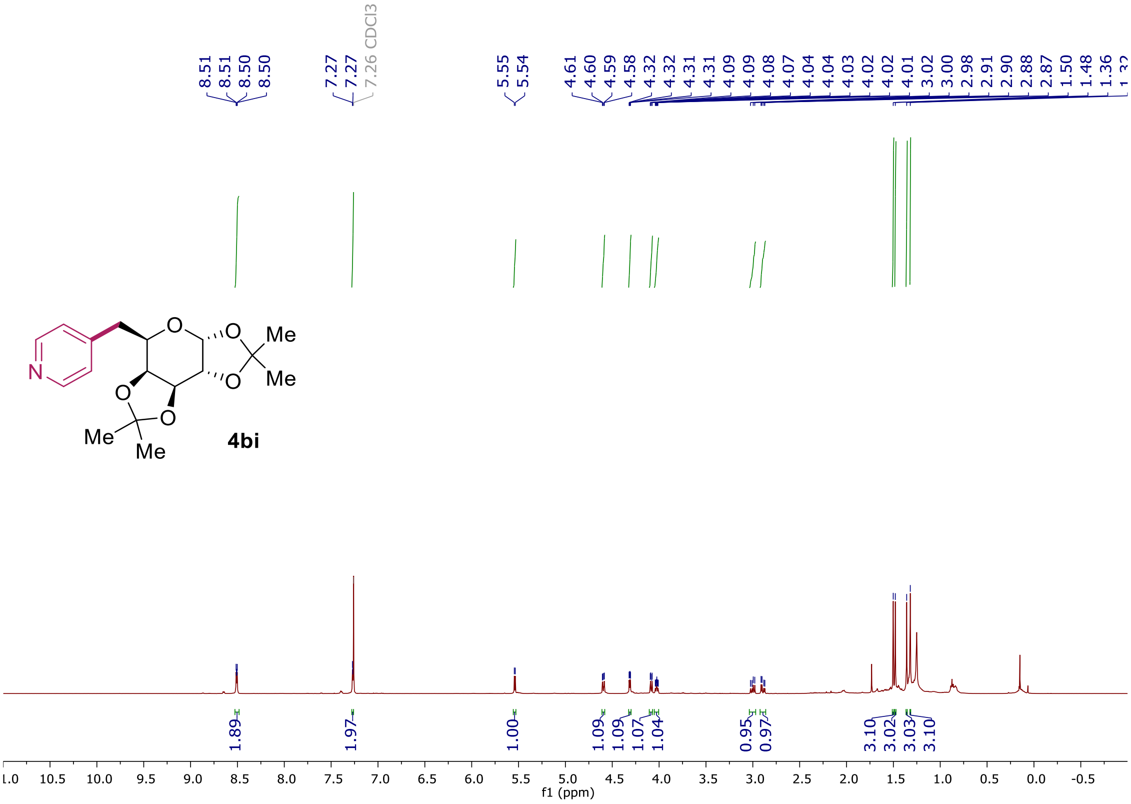

[13]**C-NMR** (75 MHz, $CDCl_3$) of compound **4bi**

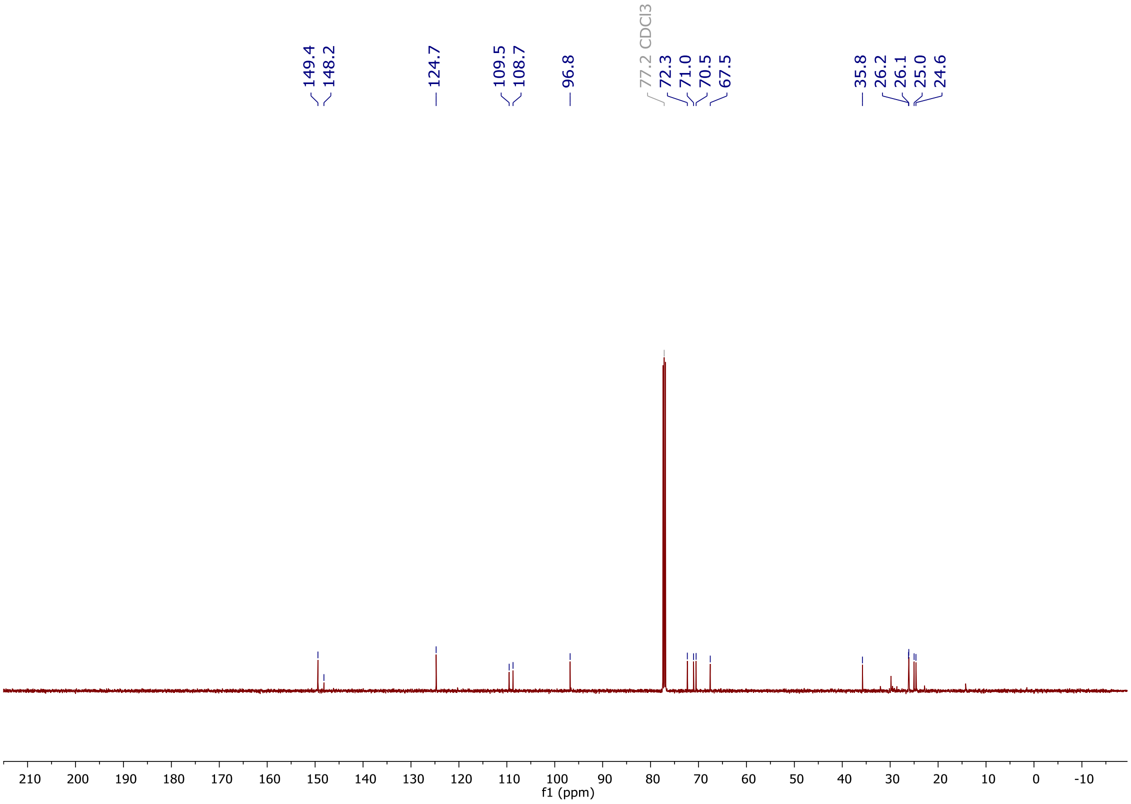

[1]**H-NMR** (300 MHz, $CDCl_3$) of compound **4bj**

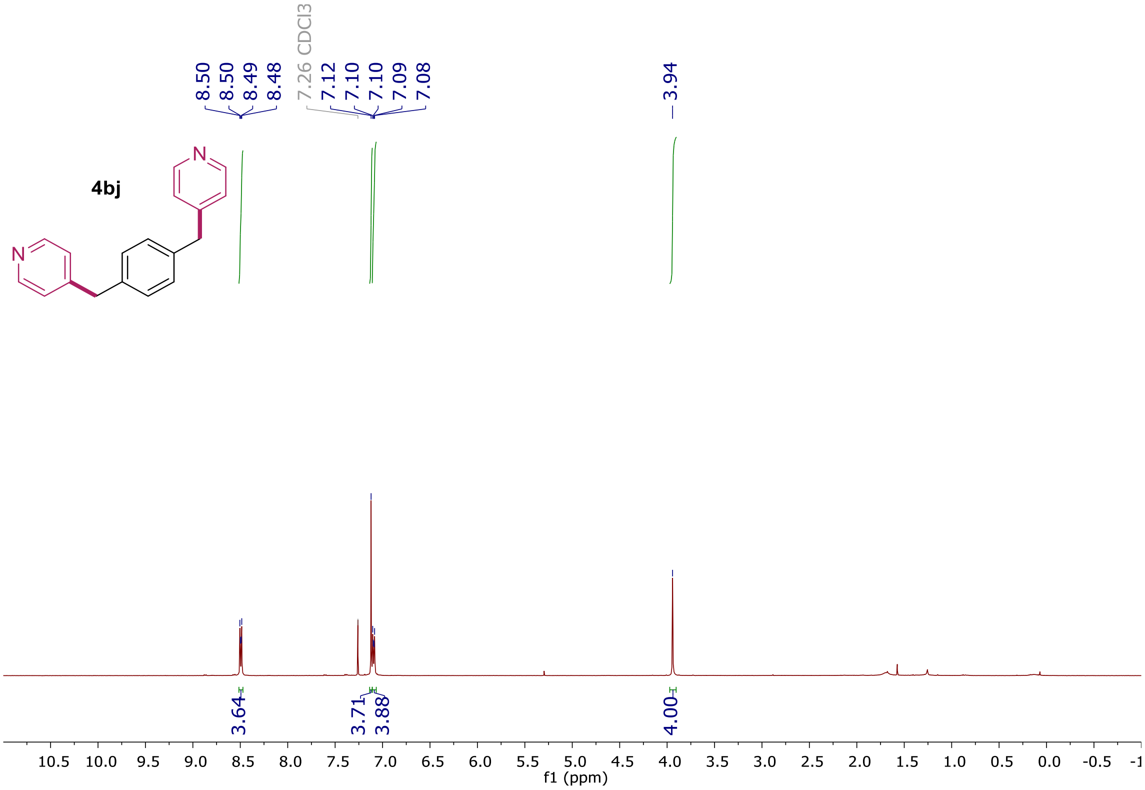

[13]**C-NMR** (75 MHz, $CDCl_3$) of compound **4bj**

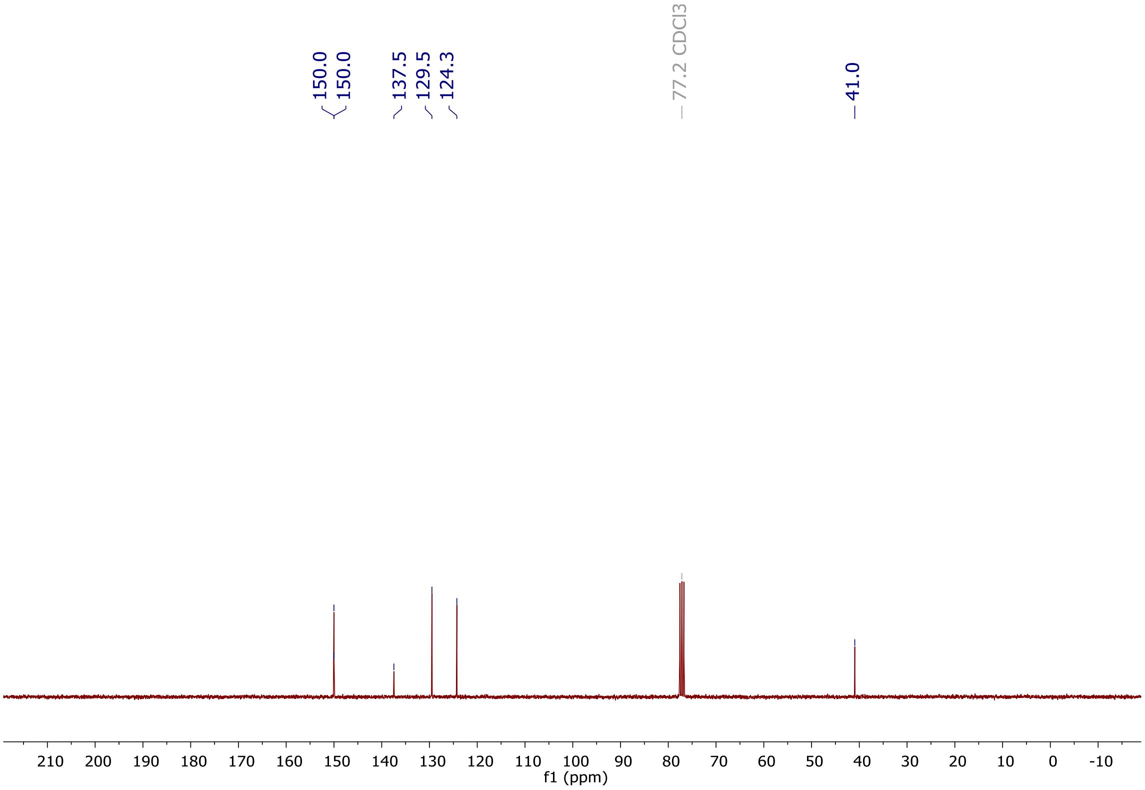

^{1}H-NMR (300 MHz, $CDCl_3$) of compound **4bk**

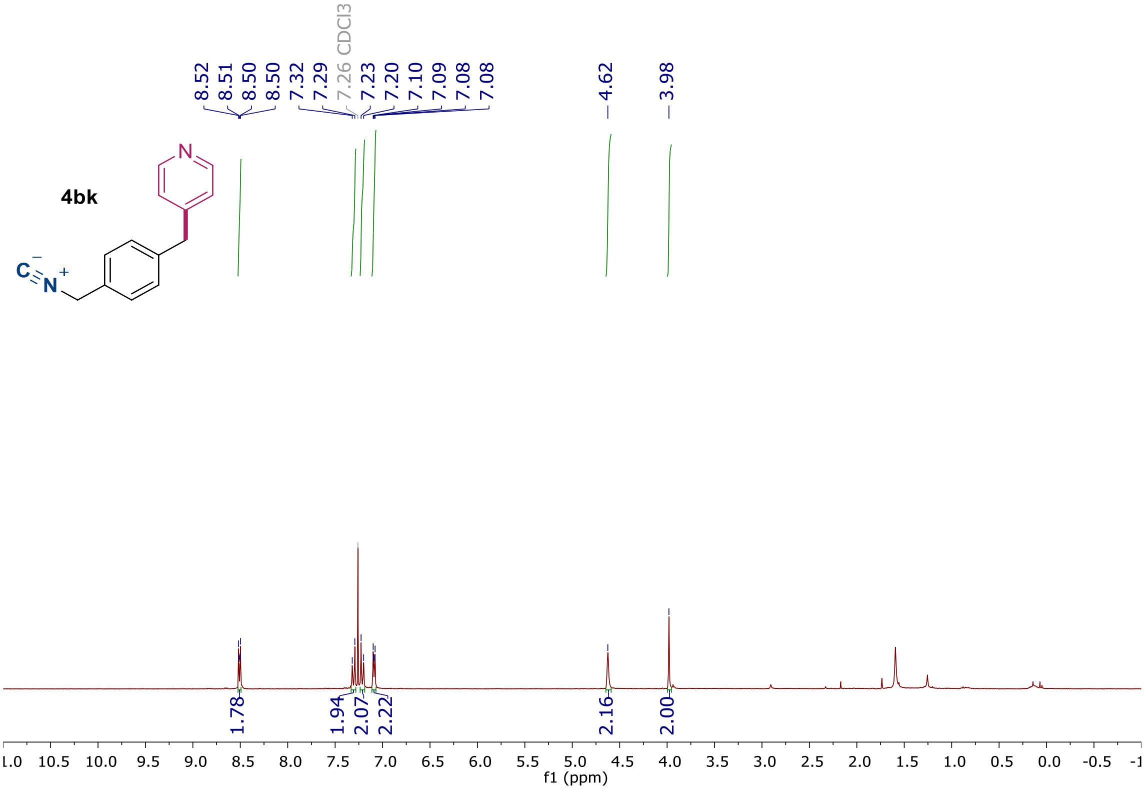

^{13}C-NMR (75 MHz, $CDCl_3$) of compound **4bk**

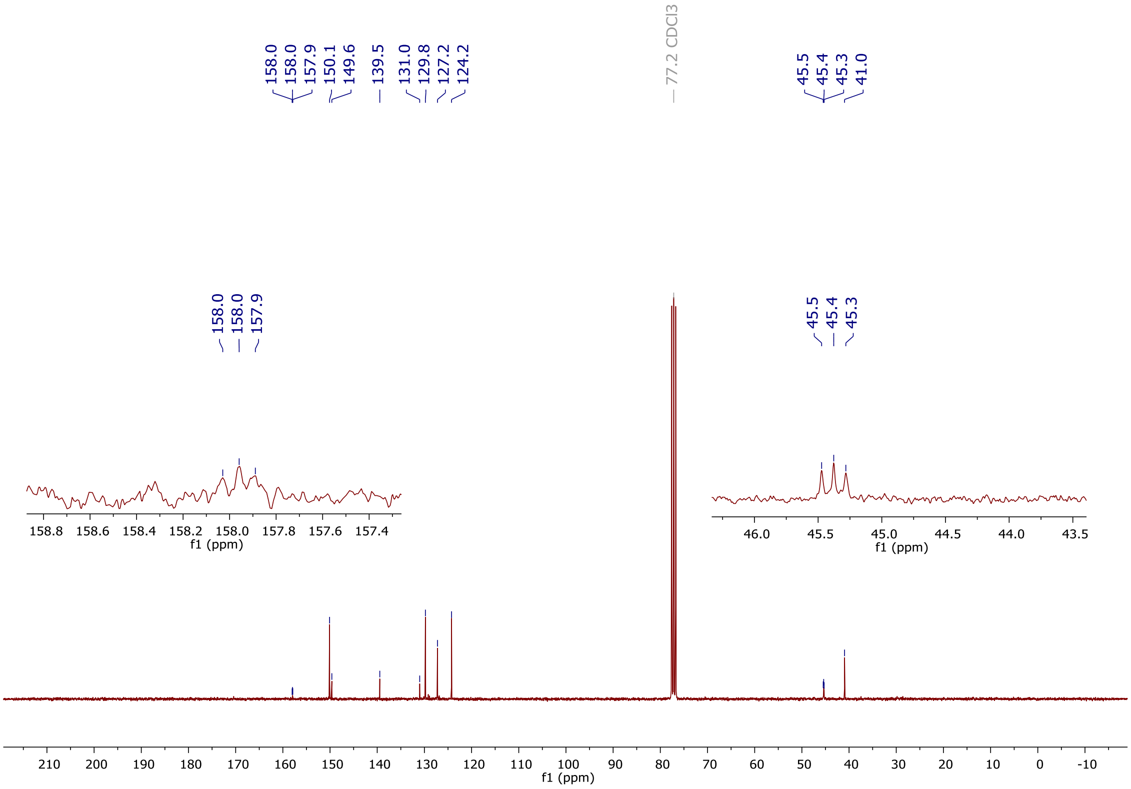

^{1}H-NMR (300 MHz, $CDCl_3$) of compound **4bl**

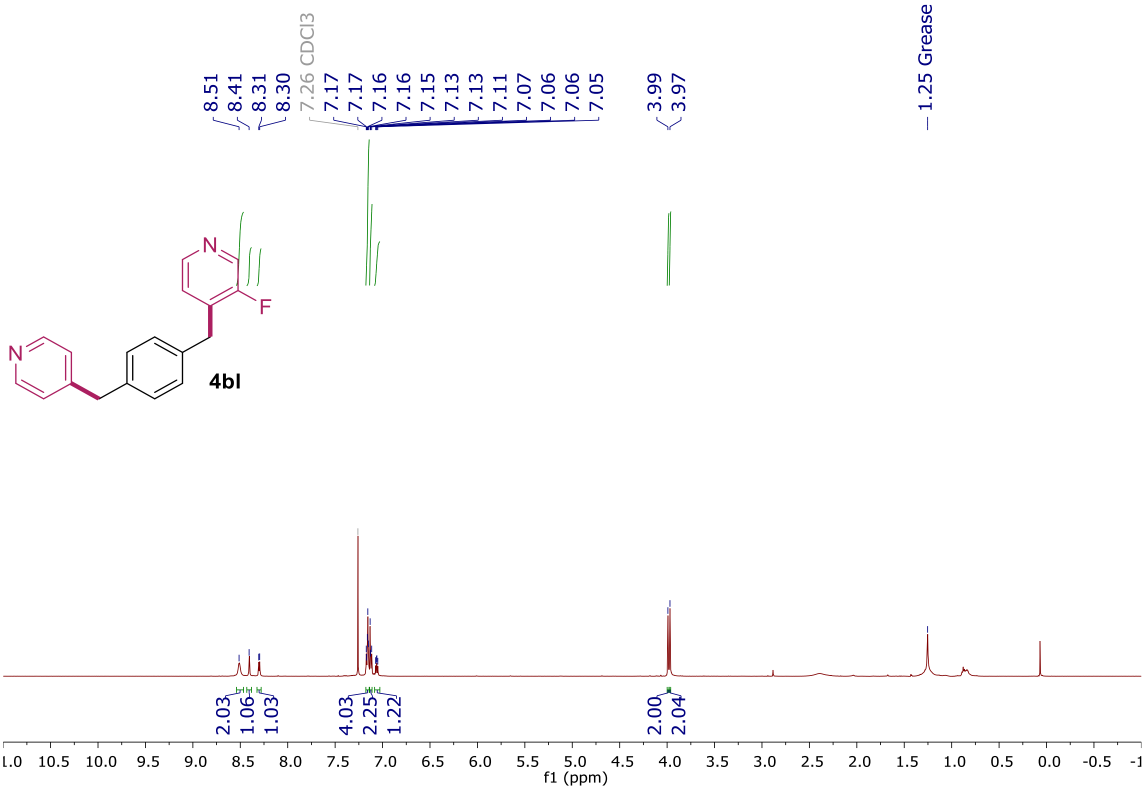

^{13}C-NMR (75 MHz, $CDCl_3$) of compound **4bl**

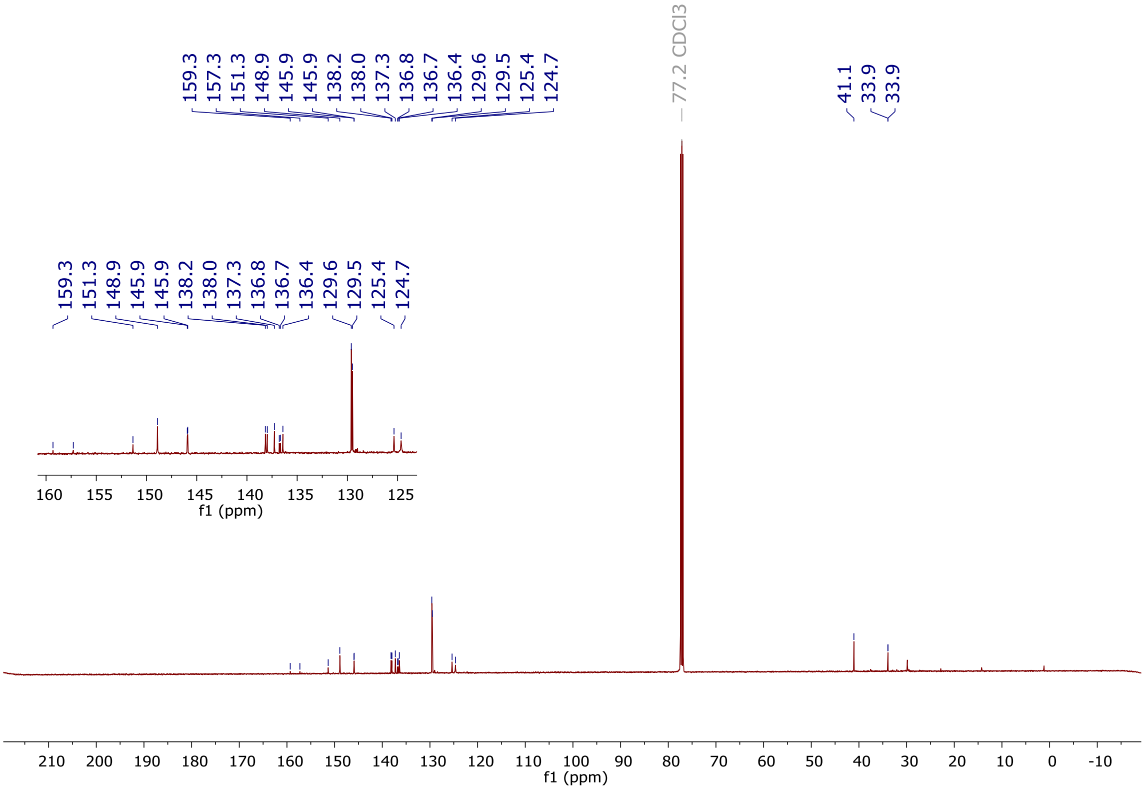

^{19}**F-NMR** (470 MHz, $CDCl_3$) of compound **4bl**

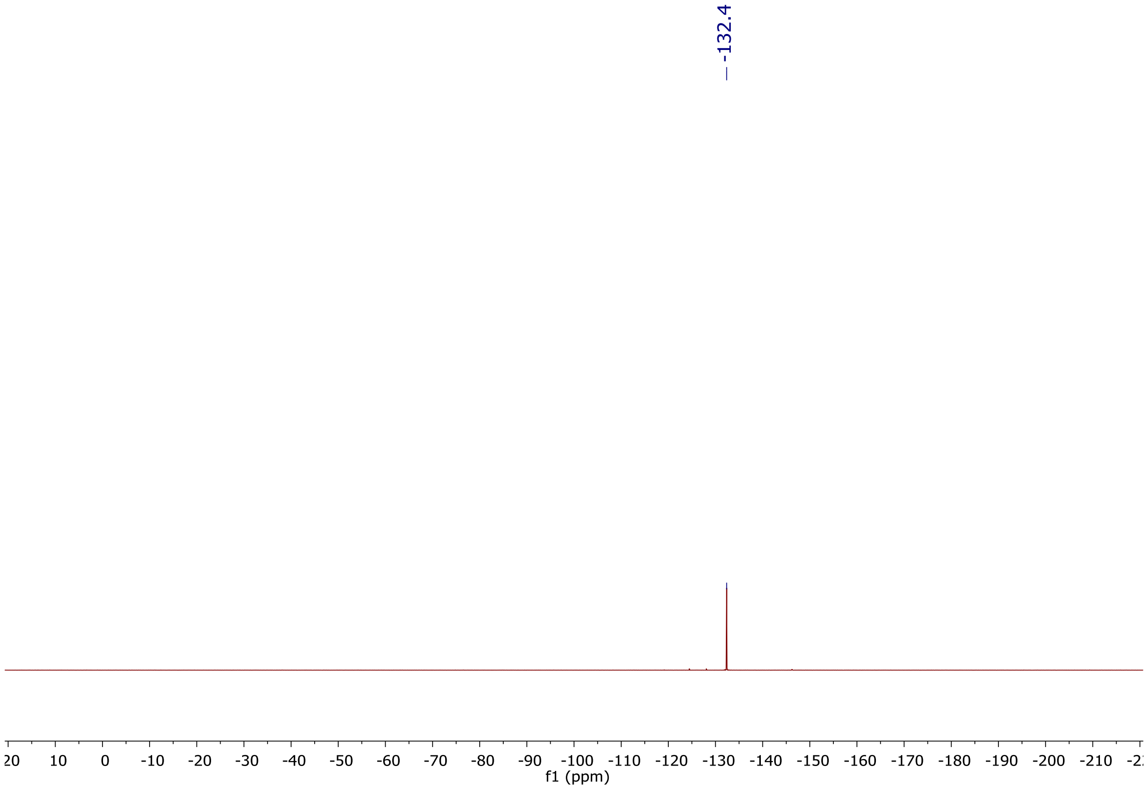

^{1}H-NMR (300 MHz, DMSO-d_6) of compound **7**

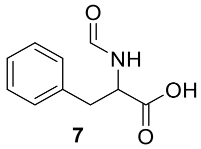

7

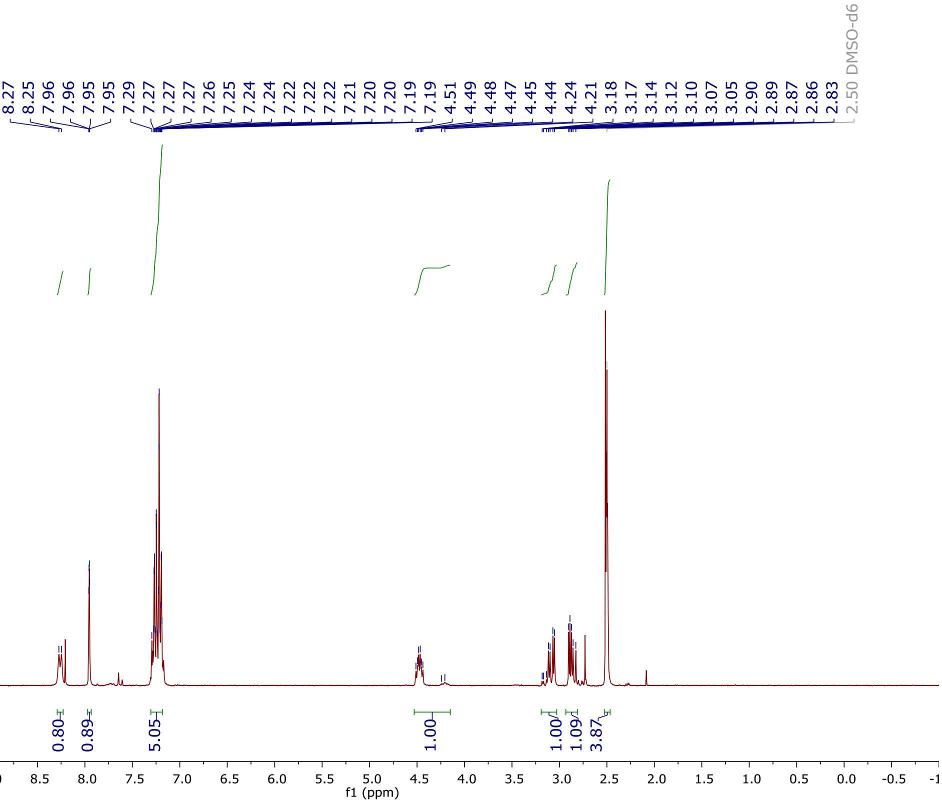

^{1}H-NMR (500 MHz, $CDCl_3$) of compound **8**

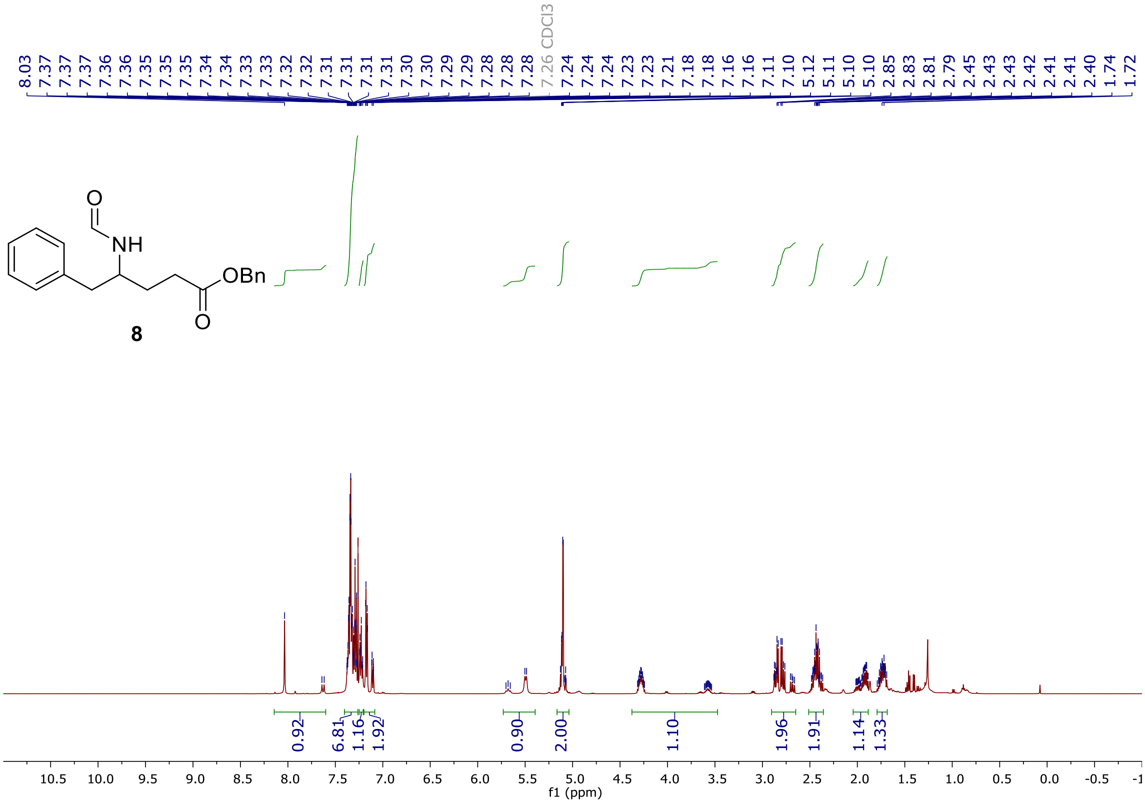

^{13}C-NMR (125 MHz, $CDCl_3$) of compound **8**

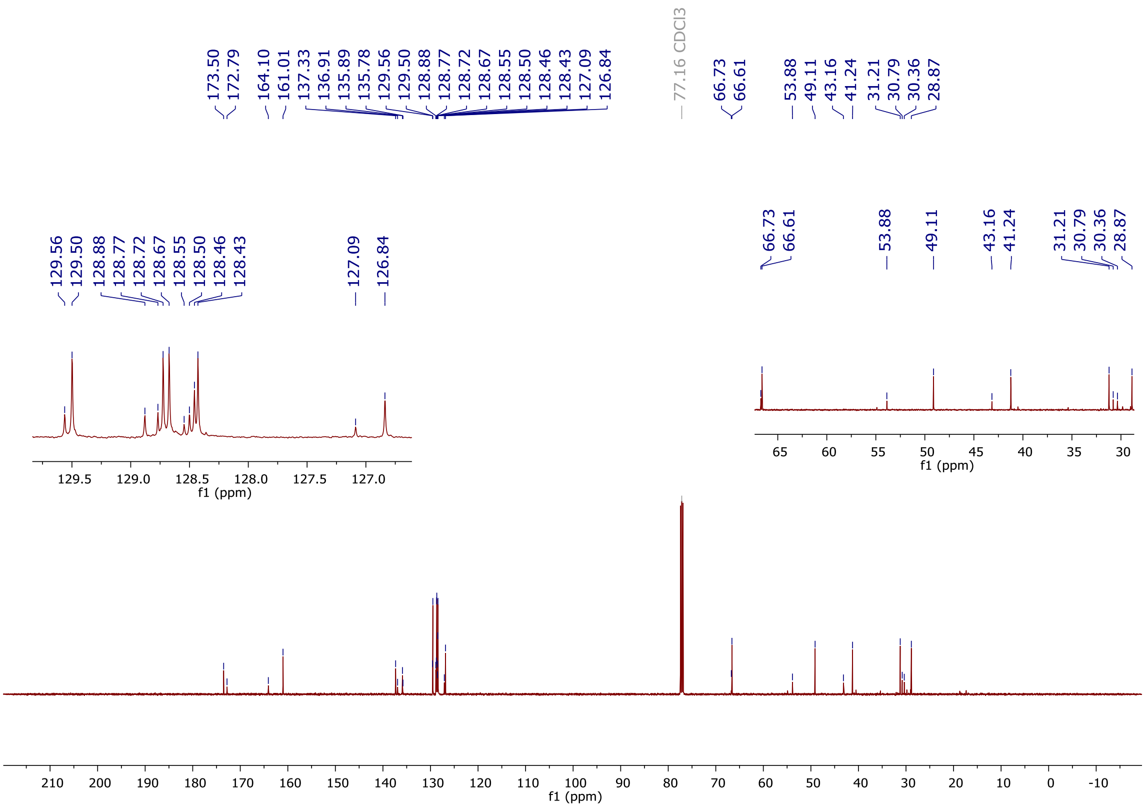

^{1}H-NMR (300 MHz, $CDCl_3$) of compound **SI-20**

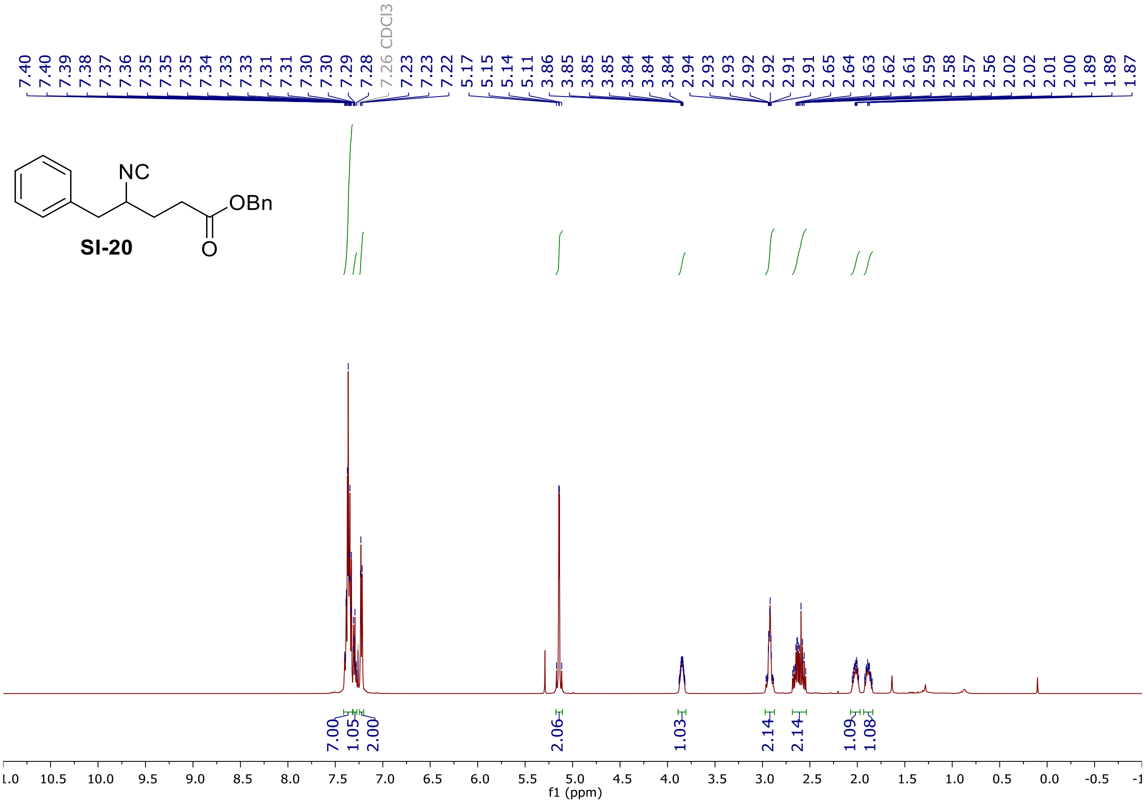

^{13}C-NMR (75 MHz, $CDCl_3$) of compound **SI-20**

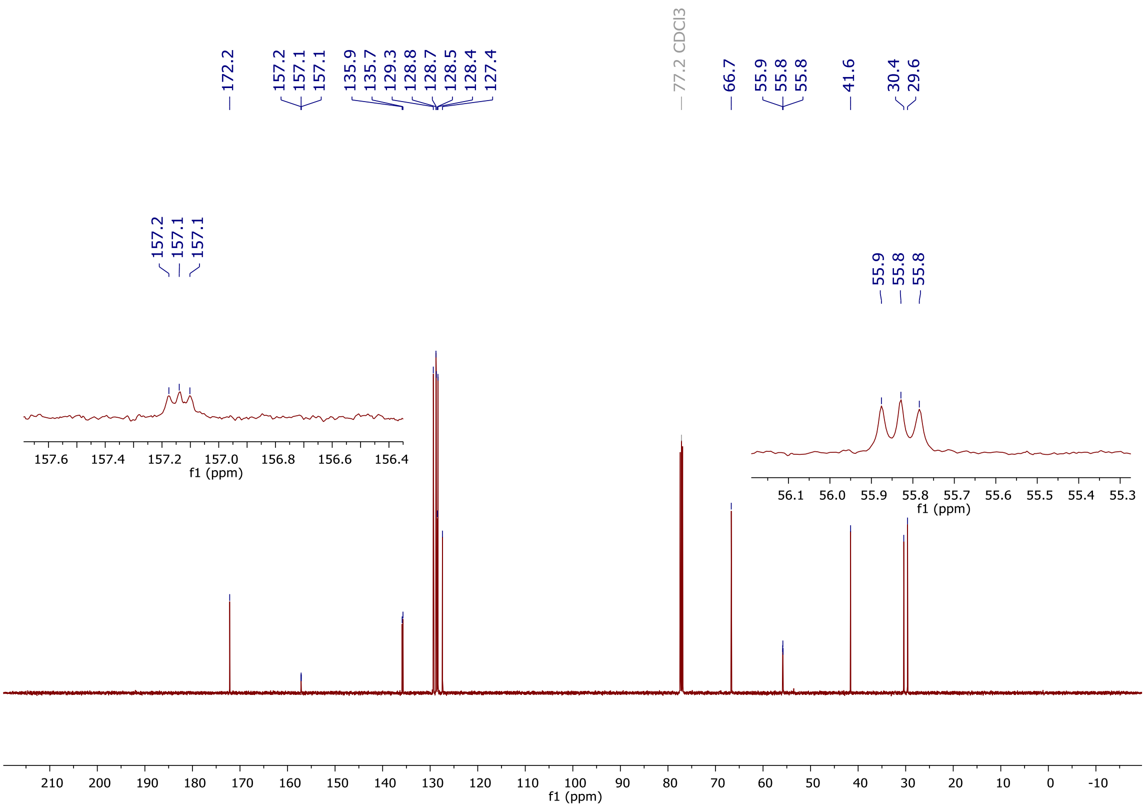

^{1}H-NMR (300 MHz, $CDCl_3$) of compound **9**

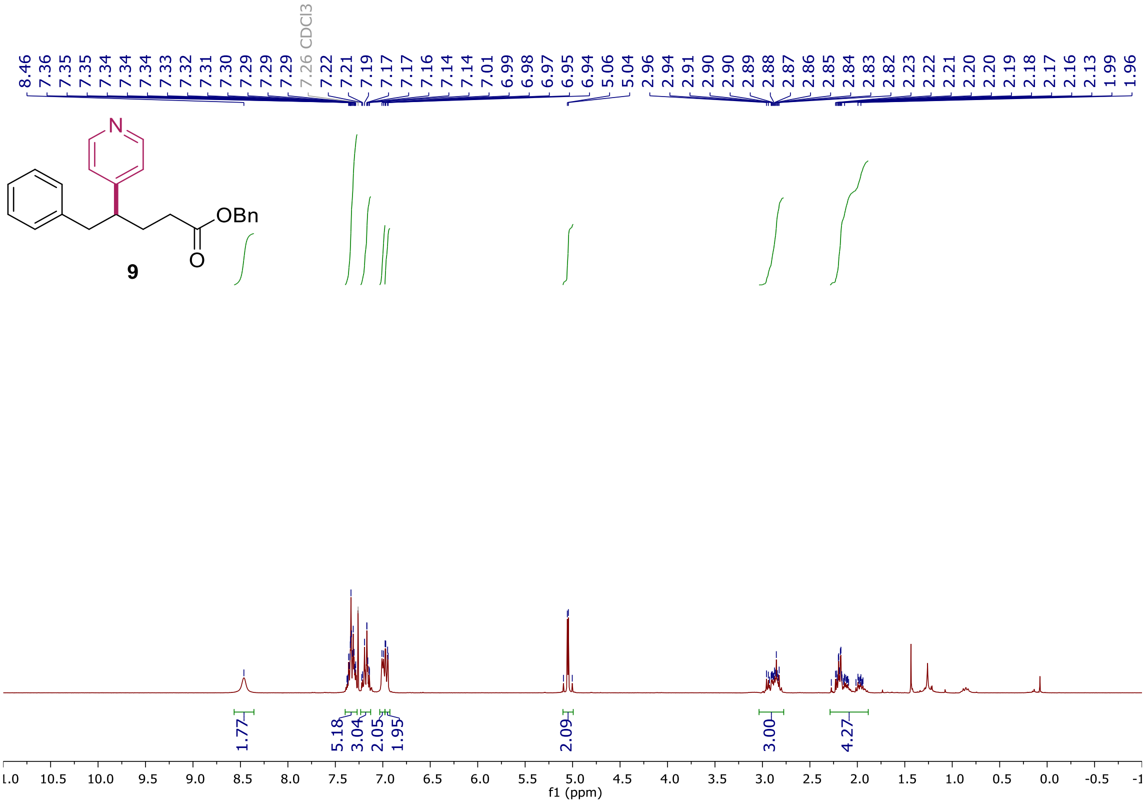

^{13}C-NMR (75 MHz, $CDCl_3$) of compound **9**

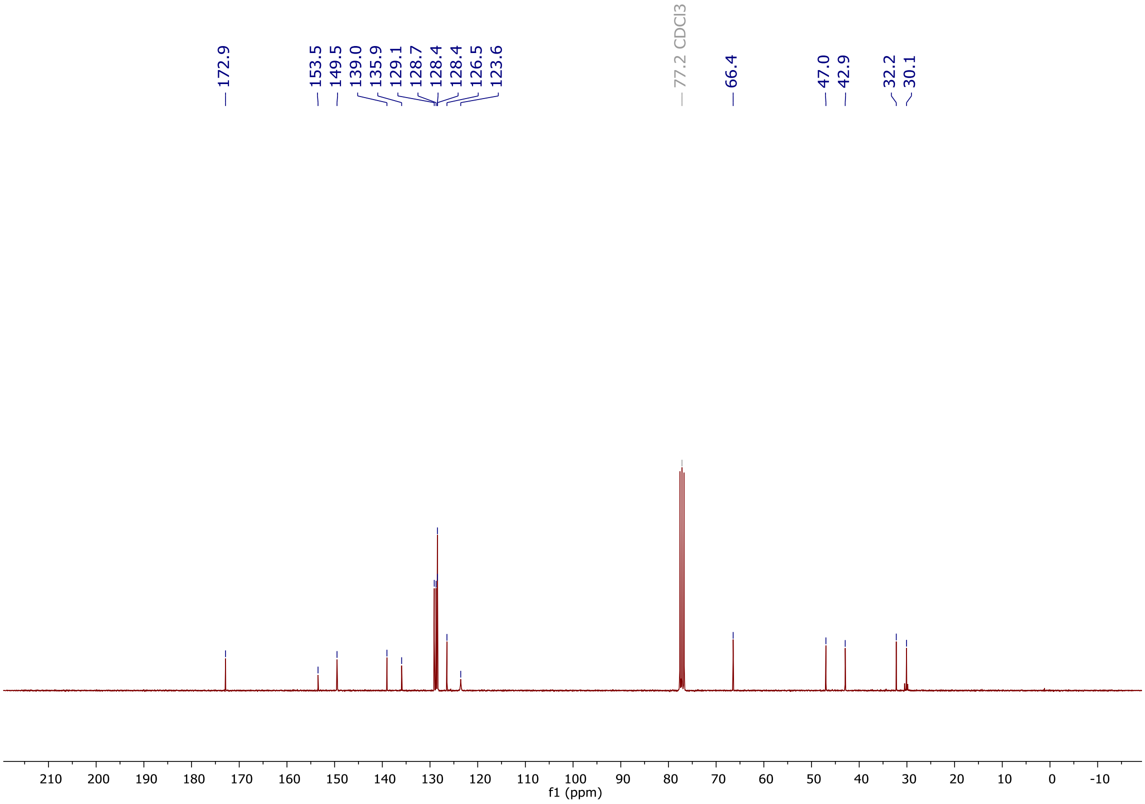

^{1}H-NMR (300 MHz, $CDCl_3$) of compound **10**

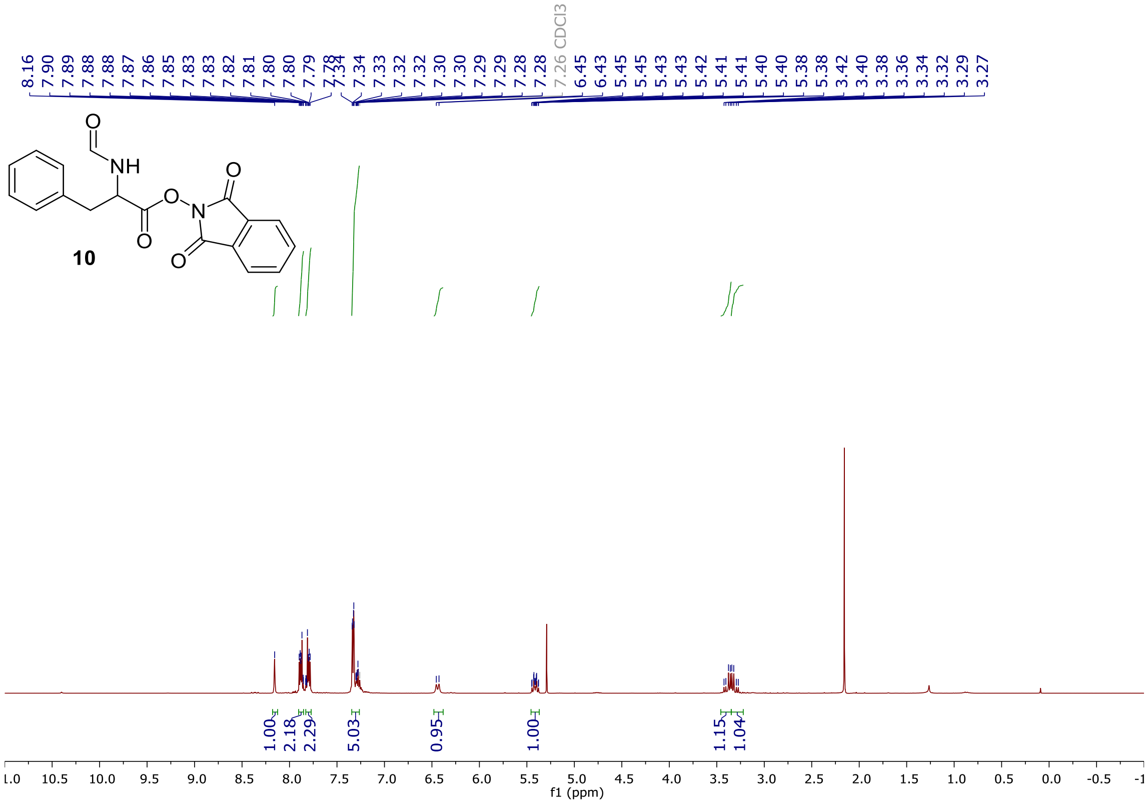

^{13}C-NMR (125 MHz, $CDCl_3$) of compound **10**

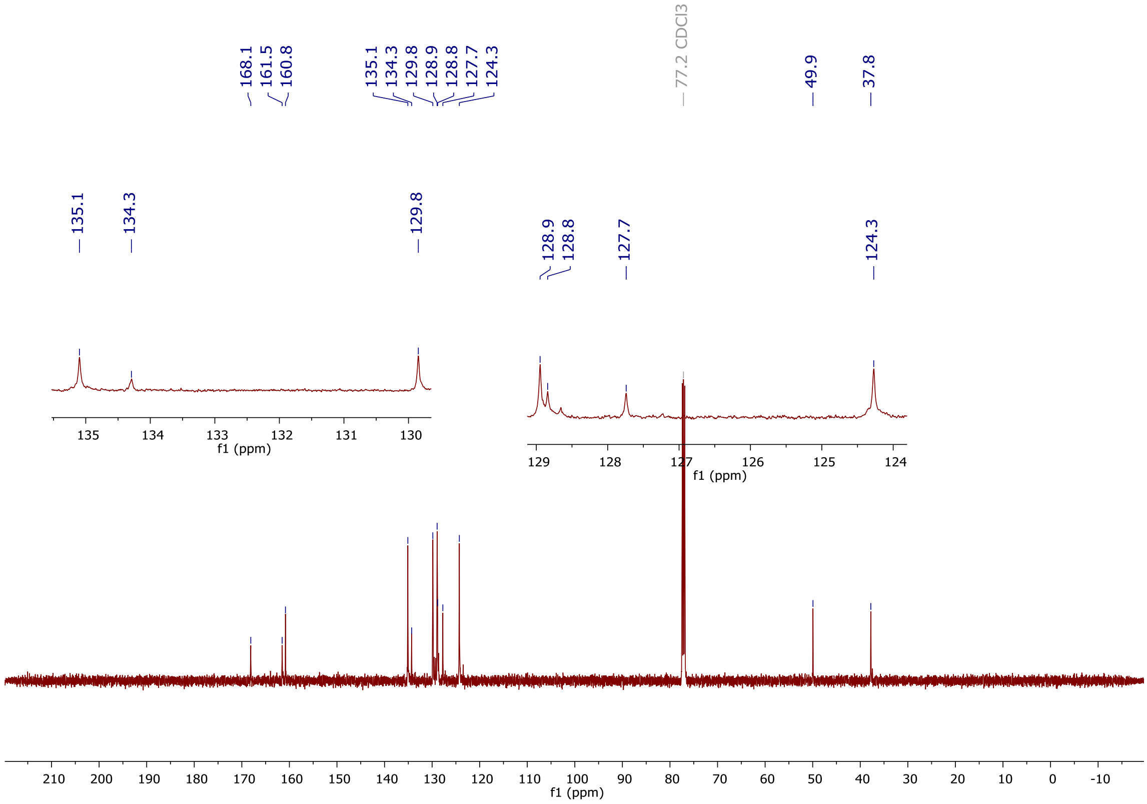

^{1}H-NMR (300 MHz, $CDCl_3$) of compound **11**

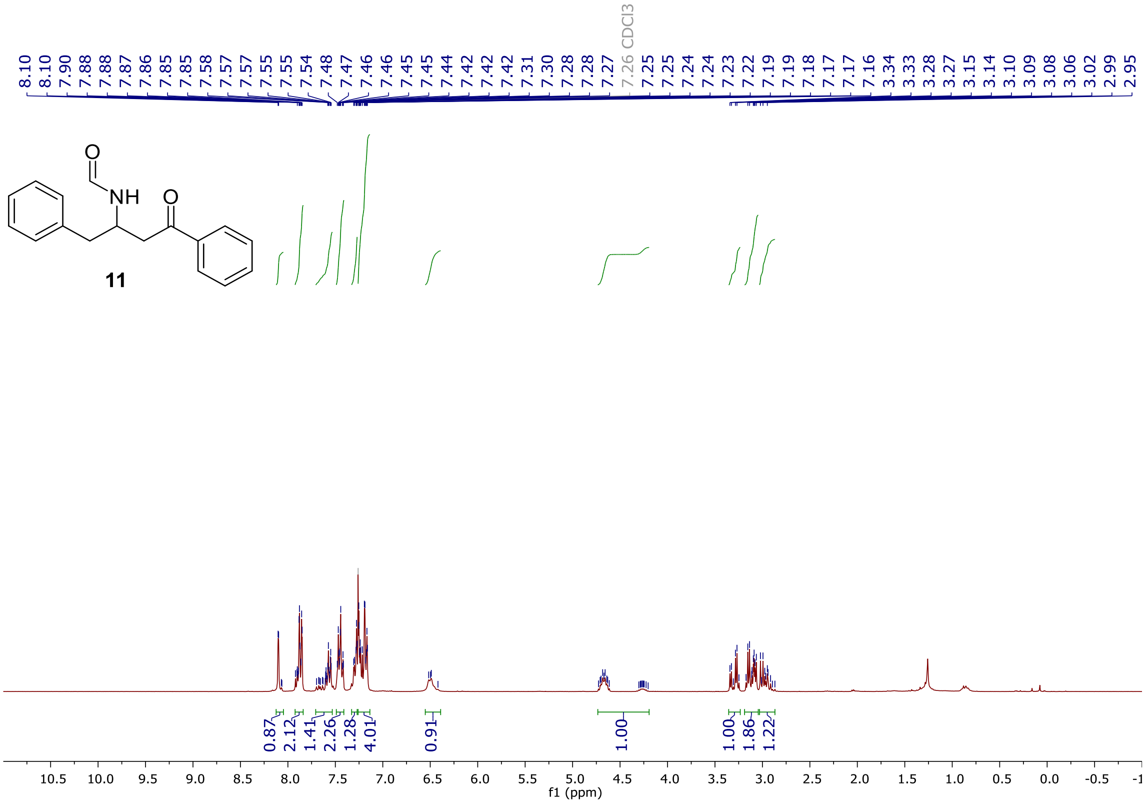

^{13}C-NMR (75 MHz, $CDCl_3$) of compound **11**

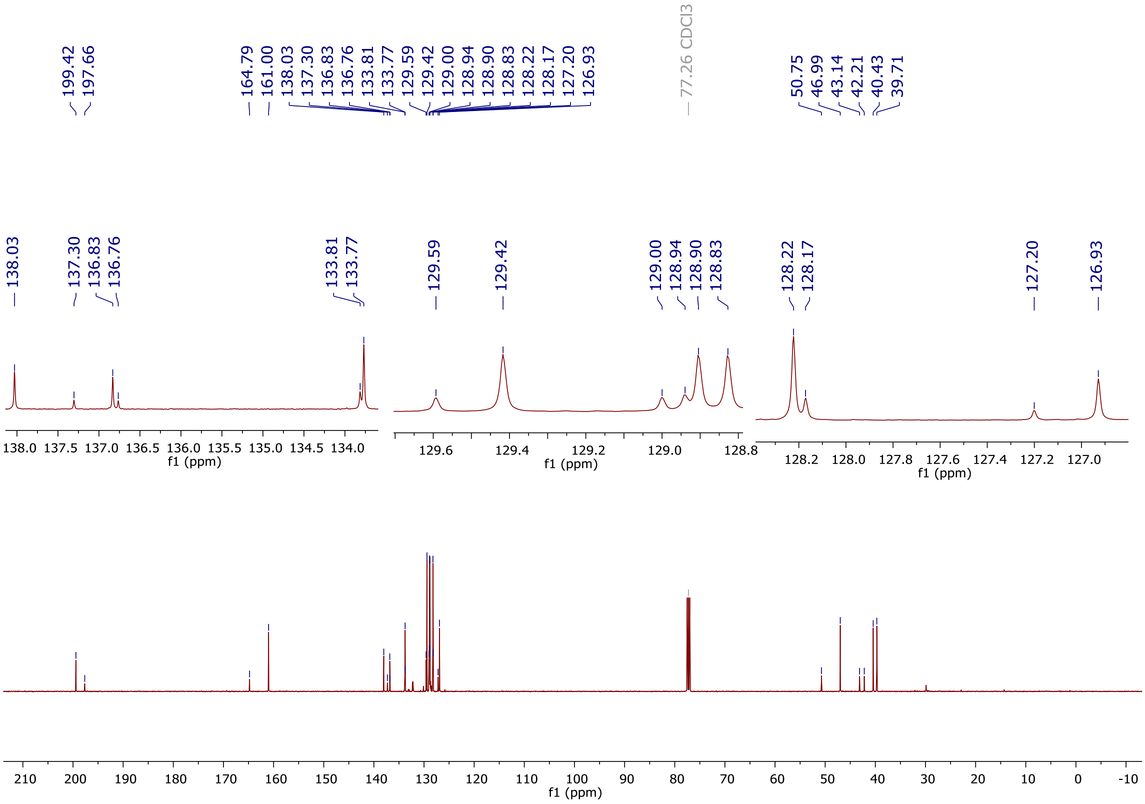

^{1}H-NMR (300 MHz, $CDCl_3$) of compound **SI-21**

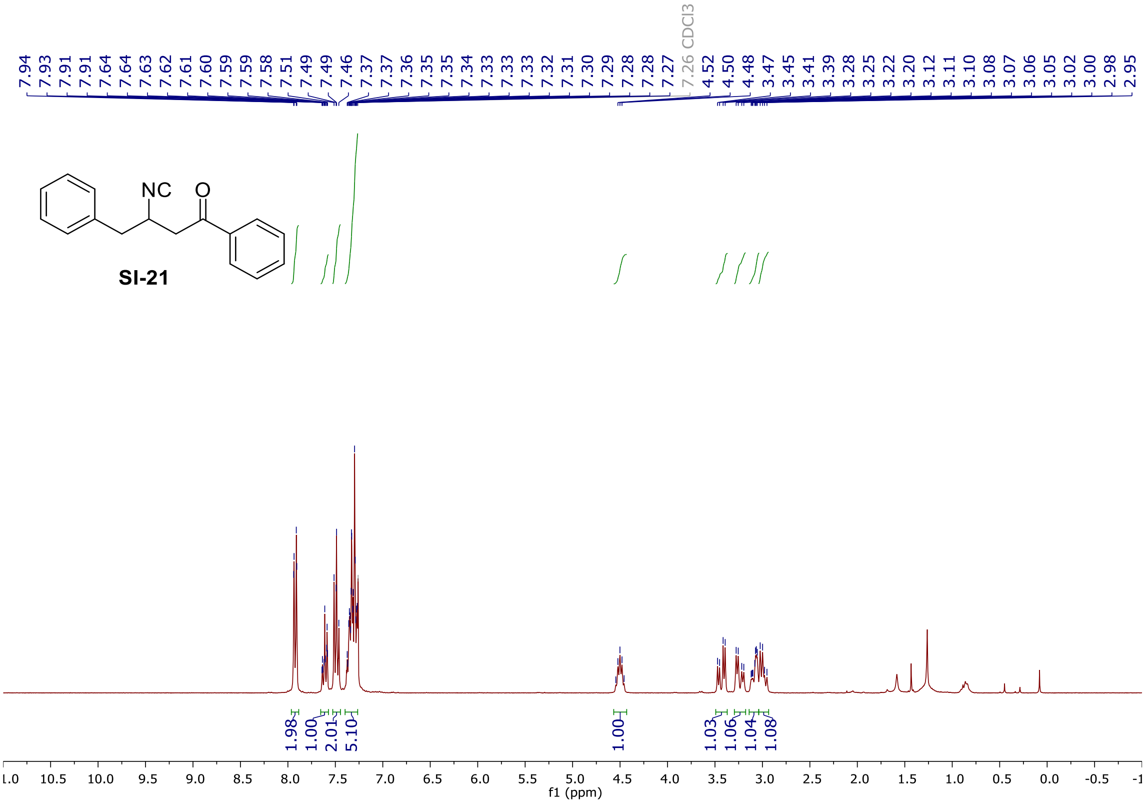

^{13}C-NMR (75 MHz, $CDCl_3$) of compound **SI-21**

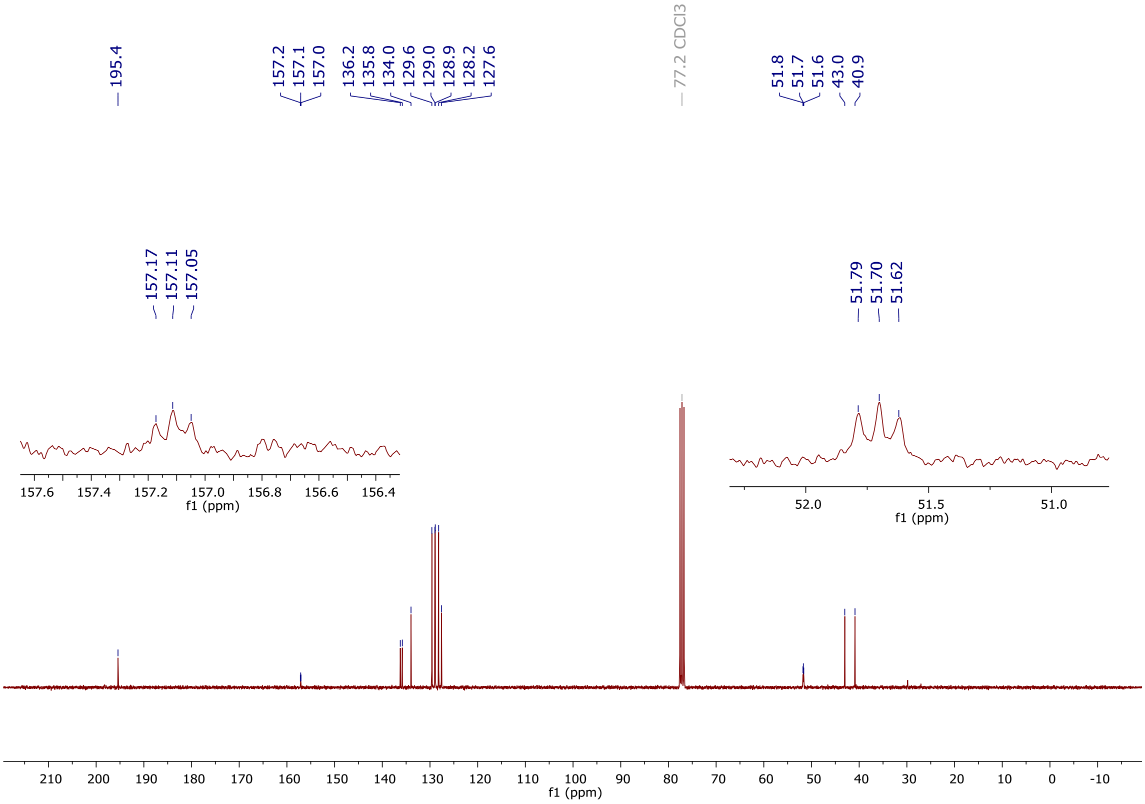

^{1}H-NMR (500 MHz, $CDCl_3$) of compound **12**

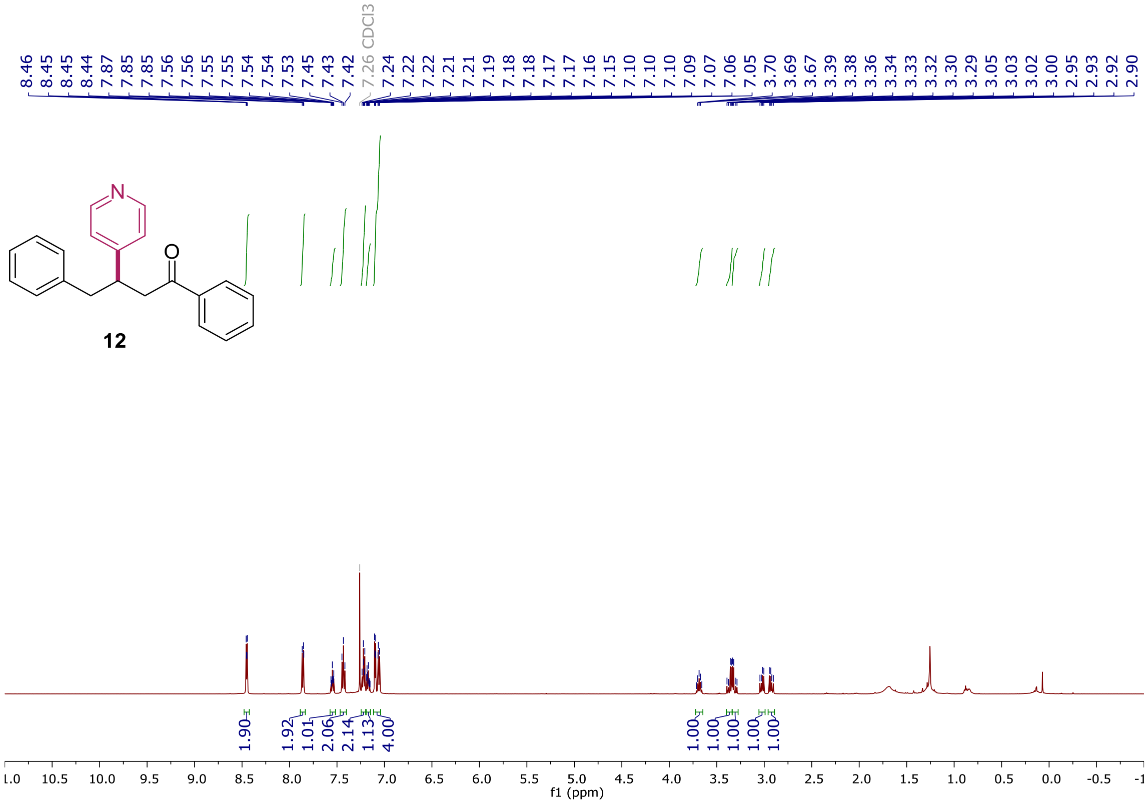

^{13}C-NMR (125 MHz, $CDCl_3$) of compound **12**

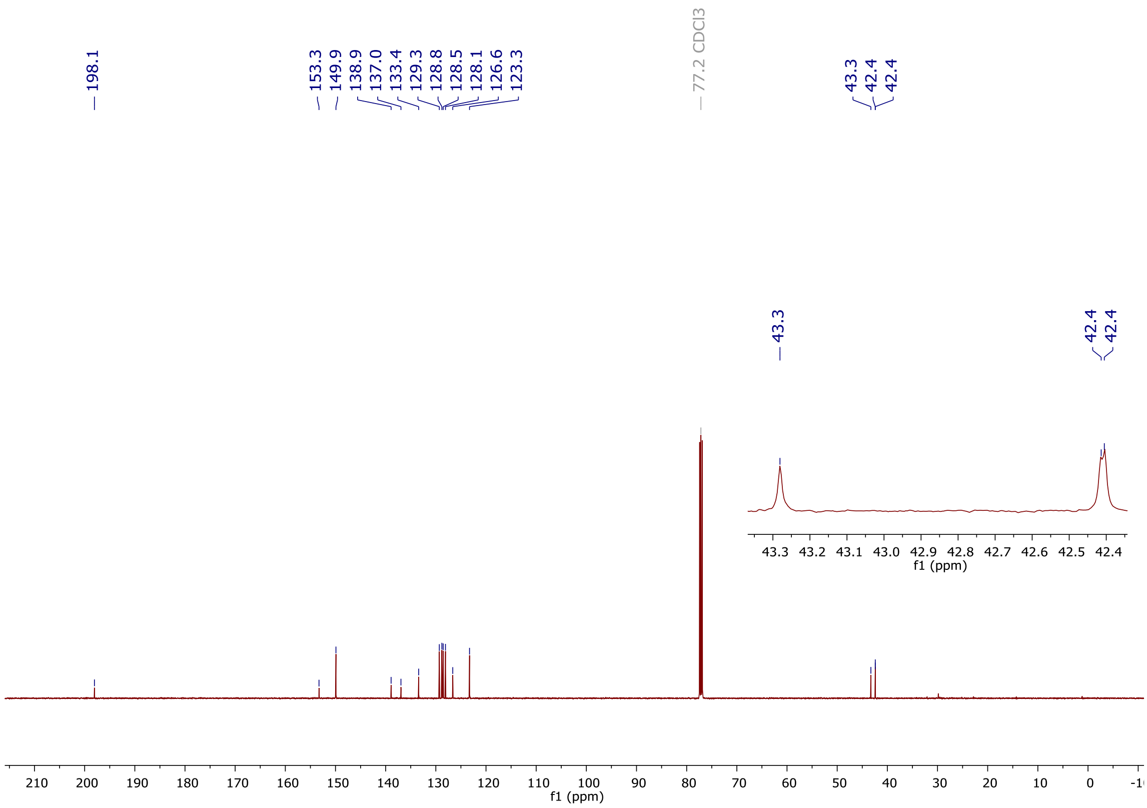

Supplement: Supplementary file 1 — cs4c06269_si_001.pdf [file cs4c06269_si_001.pdf]
